# Mechanism exploration and model construction for small cell transformation in *EGFR*-mutant lung adenocarcinomas

**DOI:** 10.1038/s41392-024-01981-3

**Published:** 2024-10-02

**Authors:** Yan Li, Tongji Xie, Shouzheng Wang, Lin Yang, Xuezhi Hao, Yan Wang, Xingsheng Hu, Lin Wang, Junling Li, Jianming Ying, Puyuan Xing

**Affiliations:** 1 State Key Laboratory of Molecular Oncology, Department of Pathology, National Cancer Center/National Clinical Research Center for Cancer/Cancer Hospital, Chinese Academy of Medical Sciences and Peking Union Medical College, Beijing, 100021 China; 2 Department of Medical Oncology, National Cancer Center/National Clinical Research Center for Cancer/Cancer Hospital, Chinese Academy of Medical Sciences and Peking Union Medical College, Beijing, 100021 China; 3 Department of Pulmonary and Critical Care Medicine, Beijing Hospital, National Centre of Gerontology, Institute of Geriatric Medicine, Chinese Academy of Medical Sciences, Beijing, 100730 China; 4 Department of Medical Oncology, Beijing Chest Hospital, Capital Medical University, Beijing Tuberculosis and Thoracic Tumor Research Institute, Beijing, 101149 China

**Keywords:** Lung cancer, Translational research

## Abstract

Small-cell lung cancer (SCLC) transformation accounts for 3–14% of resistance in *EGFR*-TKI relapsed lung adenocarcinomas (LUADs), with unknown molecular mechanisms and optimal treatment strategies. We performed transcriptomic analyses (including bulk and spatial transcriptomics) and multiplex immunofluorescence on pre-treated samples from LUADs without transformation after *EGFR*-TKI treatment (LUAD-NT), primary SCLCs (SCLC-P) and LUADs with transformation after *EGFR*-TKI treatment (before transformation: LUAD-BT; after transformation: SCLC-AT). Our study found that LUAD-BT exhibited potential transcriptomic characteristics for transformation compared with LUAD-NT. We identified several pathways that shifted during transformation, and the transformation might be promoted by epigenetic alterations (such as *HDAC10*, *HDAC1*, *DNMT3A*) within the tumor cells instead of within the tumor microenvironment. For druggable pathways, transformed-SCLC were proved to be less dependent on *EGF* signaling but more relied on *FGF* signaling, while *VEGF*-*VEGFR* pathway remained active, indicating potential treatments after transformation. We also found transformed-SCLC showed an immuno-exhausted status which was associated with the duration of *EGFR*-TKI before transformation. Besides, SCLC-AT exhibited distinct molecular subtypes from SCLC-P. Moreover, we constructed an ideal 4-marker model based on transcriptomic and IHC data to predict SCLC transformation, which obtained a sensitivity of 100% and 87.5%, a specificity of 95.7% and 100% in the training and test cohorts, respectively. We provided insights into the molecular mechanisms of SCLC transformation and the differences between SCLC-AT and SCLC-P, which might shed light on prevention strategies and subsequent therapeutic strategies for SCLC transformation in the future.

## Introduction

Lung cancer is the leading cause of cancer-related deaths,^1^ and lung adenocarcinoma (LUAD) accounts for more than 40% of lung cancer. Epidermal growth factor receptor (*EGFR*) mutations are the most frequent driver genetic events in Asian LUADs.^2^ In recent decades, the treatment of *EGFR*-mutated LUAD has been revolutionized by *EGFR* tyrosine kinase inhibitors (TKIs). However, acquired resistance eventually occurs after 10–15 months.^3^ The histological transformation to small-cell lung cancer (SCLC), accounts for ~3–14% of resistance mechanisms to *EGFR-*TKIs.^3,4^ With the front-line usage of third-generation *EGFR-*TKIs and increased practice of tumor re-biopsy, more patients were identified SCLC transformation.^5^ Transformed-SCLC is more aggressive and associated with poor prognosis.^6^ Thus, the prediction of optimal treatment for transformed-SCLC has been a major challenge for clinicians and patients.

Many studies have explored the mechanisms of SCLC transformation at the genomic level and supported the same origins of LUAD and transformed-SCLC.^7,8^ Experiments on mice revealed that loss of *Trp53* and *Rb1* in alveolar type II cells, which are the origination of adenocarcinoma, could also lead to the development of SCLC, indicating the shared ancestry of some SCLC and LUAD.^9^ In patient-derived cancer cells, Pros et al. also reported that *EGFR*-mutated LUAD with *RB1* inactivation might favor SCLC transformation under TKI selection pressure.^10^ Other studies in small number of patients also suggested that concomitant inactivation of *TP53* and *RB1* is necessary but not sufficient for SCLC transformation.^11–14^ Copy number variation (CNV) events might also played an important role in transformation.^15^ However, it has been reported recently that distinct histologic features were associated with transcriptomic features as late molecular events, rather than the genomic profiles in multiple types of cancers.^16,17^ Thus, investigating the shifts in RNA expression patterns during SCLC transformation is of great clinical significance. Recently, Quintanal-Villalonga et al. have made good attempts to explain the mechanisms of SCLC transformation by performing multi-omics analysis.^18^ However, due to the difficulty of accessing the paired pre-/post-transformation clinical samples, Quintanal-Villalonga et al. chose spatial mixed LUAD/SCLC samples instead.^18^ Nevertheless, whether the spatial mixed samples could reflect the mechanisms of transformation was still worth discussing, as the SCLC components in spatial mixed samples were not formed under anti-cancer drug pressure. Transcriptomic analyses of neuroendocrine transformation in prostate cancer have also been reported, but only on post-transformation samples.^19^ Besides, several studies have made great effort to explore the mechanisms of histological transformation from LUAD to SCLC, or to squamous cell carcinoma, using in vitro experimental methods such as lentivirus infection cell lines^20,21^ or mouse models,^22,23^ and received some important results. However, exploratory study using tumor tissues from the real-world patients can reflect the actual mechanisms of transformation in human body, and can not be replaced by in vitro or mouse model studies.

In addition, the optimal clinical treatment strategies for transformed-SCLCs remain unclear. Some studies have reported that transformed-SCLCs could benefit from platinum-etoposide chemotherapy^24,25^ alone or in combination with anti-angiogenic therapies.^26^ Whether *EGFR*-TKIs should be continued is controversial. Previous study reported that *EGFR* pathways still existed in *EGFR*-mutant transformed-SCLC using whole exome sequencing, and using three patient-derived organoids (PDOs) they reported that *EGFR*-TKI plus chemotherapy had better anti-cancer activity for transformed-SCLC than chemotherapy or TKI alone.^27^ However, case reports revealed that transformed-SCLC received limited benefit from *EGFR*-TKI plus chemotherapy.^4^ Our previous study explored the treatment modes and outcomes after SCLC transformation, and proved that the addition of *EGFR*-TKI to chemotherapy could significantly improve the progression-free survival (PFS) after transformation, but no significant difference in overall survival (OS) were observed between transformed-SCLCs received chemotherapy with or without *EGFR*-TKIs.^26^ New druggable molecular targets need to be identified by exploring the characteristics of transformed-SCLCs. Previous study described primary SCLCs as immune cold tumors and only a minority of SCLCs demonstrated benefit from immuno-monotherapy.^28^ However, a recent study indicated that immunotherapy plus chemotherapy could increase clinical survival in extensive SCLCs,^4,29^ offering hope for immunotherapy plus chemotherapy in transformed-SCLCs. Besides, previous study also reported that the combination of immunotherapy and chemotherapy had significantly improved the clinical outcomes for advanced *EGFR*-mutant lung cancers after TKI failure,^30^ offering hope for immunotherapy plus chemotherapy in transformed-SCLCs. However, the response of transformed-SCLC to immunotherapy is also controversial.^24,26,31^ Thus, investigation of tumor immuno-microenvironment (TIME) characteristics of transformed-SCLC is also of great clinical importance. Moreover, four molecular subtypes of SCLC have been described, and different molecular subtypes might have different optimal treatments.^32^ However, few studies have investigated molecular subtypes of transformed-SCLC.

In this study, we performed integrated transcriptomic analysis, including bulk and spatial, on pre/post-transformed tissue from *EGFR*-mutated LUAD patients, and explored the mechanism of transformation and the key driver. Furthermore, we compared the transcriptomic features and molecular subtypes of transformed and primary SCLC to explore the subsequent therapeutic strategies after transformation. In addition, TIME characteristics were explored using multiplex immunofluorescence (mIF) among pre/post-transformed tissues and primary SCLC samples. A model to predict SCLC transformation was also constructed by comparing LUADs with and without transformation, and was validated in an expanded cohort.

## Results

### Gene expression patterns of both bulk RNA and spatial transcriptomic (ST) data reveal the relationships among four groups

**Fig. 1:**
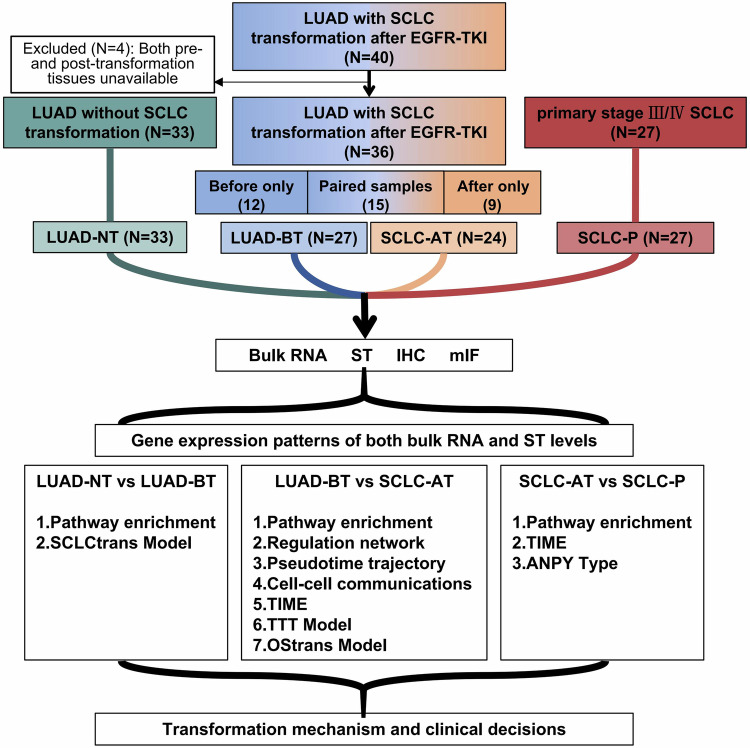
Study flow chart. LUAD-NT never-transformed lung adenocarcinoma, LUAD-BT lung adenocarcinoma before transformation, SCLC-AT small-cell lung cancer after transformation, SCLC-P primary small-cell lung cancer, TTT time to transformation, TIME tumor immuno-microenvironment

Study flow chart is shown in Fig. 1. Patients’ clinicopathologic characteristics of *EGFR*-mutant LUADs were never transformed after *EGFR*-TKI treatment (pre-treated samples were collected as LUAD-NT) (*N* = 33), LUADs with SCLC transformation after *EGFR*-TKI treatment (*N* = 40, including before-transformation LUAD samples [LUAD-BT] and after-transformation SCLC samples [SCLC-AT]) and primary SCLCs (SCLC-P) (*N* = 27) are summarized (Supplementary Table 1, details of each patient in Supplementary Table 2). Progression-free survival (PFS) analysis of LUAD patients enrolled in our study were summarized in Supplementary Fig. 1. The median time to transformation (TTT) of 40 LUADs with transformation was 28.4 months.

**Fig. 2:**
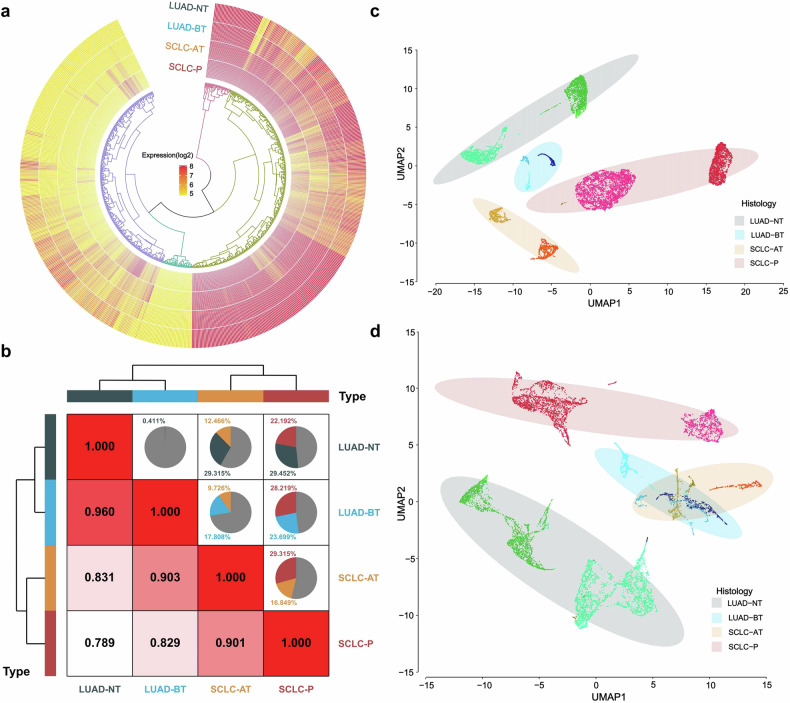
Gene expression patterns of both bulk RNA and ST data revealed the relationships of four groups. **a** Annular heatmap of bulk RNA expression. **b** Spearman’s correlation coefficients and percentage of DEGs between each two groups using bulk RNA data. **c** UMAP analysis based on ST data of tumor spots. **d** UMAP analysis based on ST data of non-tumor spots

Bulk RNA expression analysis was performed on 12, 18, 21, and 20 samples of four groups, respectively (including paired before-transformation LUAD samples [LUAD-BT] and after-transformation SCLC samples [SCLC-AT] from 12 patients, Supplementary Table 2). After normalization of 730 RNAs’ expression, annular heatmap (Fig. 2a) showed that expression pattern of SCLC-AT represents intermediate between LUAD-BT and SCLC-P. To further compare the relationships of four groups, we calculated the Spearman’s correlation and percentage of differential expressed genes (DEGs) between each two groups (Fig. 2b). As expected, the two LUAD groups had the highest correlation coefficient (r = 0.960) and smallest percentage of DEGs. Notably, both the correlation coefficient of SCLC-AT with LUAD-BT (r = 0.903) and that with SCLC-P (r = 0.901) were relatively high, indicating SCLC-AT clustered adjacent to both LUAD-BT and SCLC-P. Besides, SCLC-P seemed to have a greater correlation coefficient with LUAD-BT than with LUAD-NT (r = 0.829 versus 0.789), indicating LUAD-BT already showed potential characteristics for transformation.

ST data were generated on 2, 3, 3, and 2 samples of four groups, respectively (including paired LUAD-BT and SCLC-AT samples from 3 patients, Supplementary Table 2). Tumor spots were identified using Loupe Browser 6 and “estimate” algorithm.^33^

To accurately analyze the transcriptomic profile of tumor cells, only transcriptomic data of tumor spots were submitted to principal component analysis (PCA) and uniform manifold approximation and projection (UMAP) analysis for dimensionality reduction, which revealed the same gradual relationships among these four groups (Fig. 2c). These observations suggested that LUAD-BT was distinctly primed to transform compared to LUAD-NT, while SCLC-AT remained features of both LUAD-BT and SCLC-P with an intermediate phenotype. Besides, ST data of non-tumor spots were also submitted to PCA and UMAP analysis. Unlike tumor spots, non-tumor spots from LUAD-BT and SCLC-AT overlapped (Fig. 2d), indicating the changes of tumor microenvironment during transformation were not as dramatic as tumor cells.

To investigate the heterogeneity of tumor cells during transformation in the same patient, tumor spots from paired LUAD-BT and SCLC-AT samples were submitted together for clustering, and the optimal cluster number for each patient was determined separately (Supplementary Fig. 2). All the three patients showed the same trend that the tumor cells of SCLC-AT were evolved from subgroups of LUAD tumor cells before transformation (Supplementary Fig. 2), indicating the existence of certain tumor subgroups before transformation with transcriptomic characteristics that have the potential of SCLC transformation under TKI pressure.

### Mechanisms of SCLC transformation

**Fig. 3:**
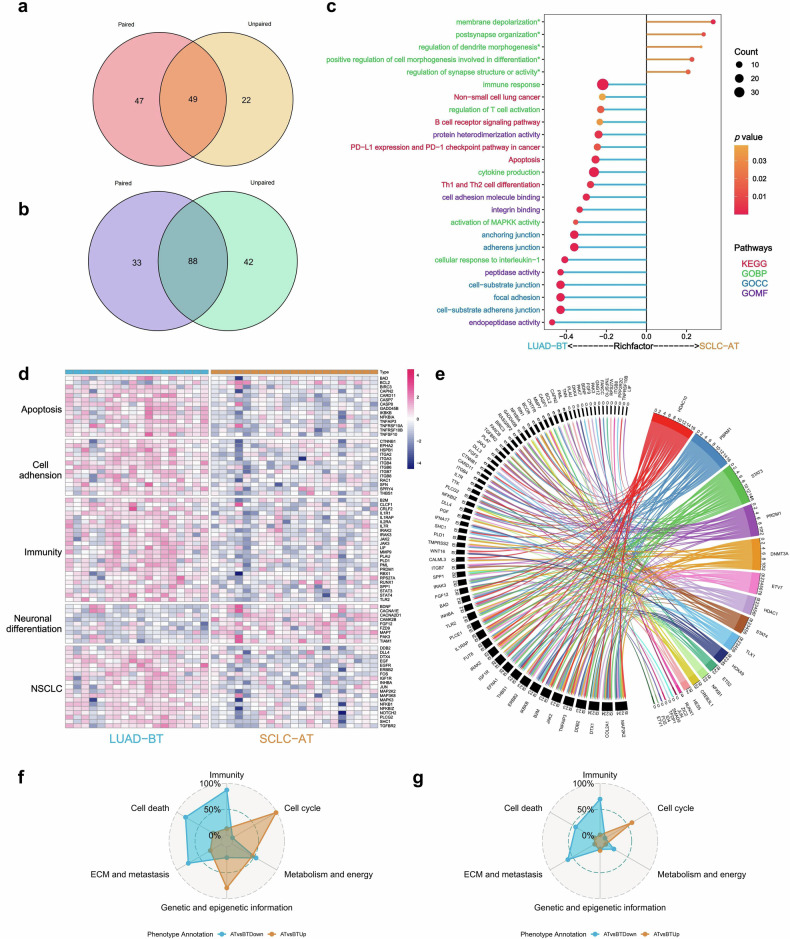
Transcriptomic characteristics during SCLC transformation according to comparison of LUAD-BT and SCLC-AT samples. **a**, **b** Significantly up- and down-regulated genes (both in paired test with samples from 12 patients and in unpaired test with 18 LUAD-BT and 21 SCLC-AT samples) which enrolled in further analysis. **c** Pathway enrichment analyses on the DEGs of LUAD-BT vs SCLC-AT. **d** Heatmap highlighting DEGs of interest grouped by associated pathways. **e** Weighted regulation network according to Spearman’s correlation coefficients. **f** GSEA utilizing MSigDB. Radar plot illustrates all 1350 pathways in SCLC-AT vs LUAD-BT. **g** GSEA utilizing MSigDB. Radar plot illustrates pathways statistically significant in SCLC-AT vs LUAD-BT

To analyze the transcriptomic changes during transformation, we performed DEGs and pathway enrichment analyses with bulk RNA data of LUAD-BT and SCLC-AT. One hundred thirty-seven DEGs (49 up-regulated and 88 down-regulated genes, Supplementary Table 3), which were statistically significant (raw *P* < 0.05 & false discovery rate [FDR] < 0.25) both in paired test with samples from 12 patients and in unpaired test with 18 LUAD-BT and 21 SCLC-AT samples, were included (Fig. 3a, b). Pathway enrichment analyses showed up-regulated genes during transformation involved in neural differentiation (including pathways related to membrane depolarization, postsynapse organization, regulation of dendrite morphogenesis, regulation of synapse structure or activity etc.), as well as down-regulation genes involved in (1) non-small cell lung cancer (NSCLC); (2) apoptosis; (3) cell adhesion (including pathways related to cell adhesion molecular binding, integrin binding, anchoring junction etc.); (4) immunity (regulation of T-cell activation, B-cell receptor signaling pathway, Th1 and Th2 cell differentiation etc.). Figure 3c showed the pathway enrichment analyses on the DEGs of LUAD-BT versus SCLC-AT; Fig. 3d showed heatmap highlighting DEGs of interest grouped by associated pathways.

We also calculated the DEGs’ expression pattern of all 71 samples with bulk RNA data in four groups. The scatter plot, which displayed the weighted expression of up-regulated and down-regulated DEGs (Supplementary Fig. 3a, Supplementary Table 4), echoed the results of the gradual relationships among four groups. Besides, we also investigated whether transformation mechanisms were associated with *EGFR*-sensitizing mutation type (19del and L858R) (Supplementary material 1). It seemed that patients harboring L858R were more relied on the up-regulation of neural differentiation, while patients harboring 19del were regulated by both up-regulation of neural differentiation and down-regulation of other mechanisms such as NSCLC, apoptosis, cell adhesion, and immunity (Supplementary Fig. 3b).

We noticed that among 137 DEGs, 17.5% (24/137) were transcriptional regulation factors. To further investigate the key regulation factors during transformation, we analyzed the relationships between regulation factors and regulated genes during transformation and constructed the weighted regulation network according to Spearman’s correlation coefficients. The weighted regulation network was shown in Fig. 3e. *HDAC10* was found to be the key regulation factor which was down-regulated during transformation. Other important regulation factors involved in (1) transcription factors: up-regulation of *PBRM1*, *TLX1*, *HOXA9*, *HES5*; and down-regulation of *STAT3*, *PRDM1*, *ETV7*, *STAT4*, *ETS2*; (2) DNA methyltransferases: up-regulation of *DNMT3A*; (3) histone deacetylases: down-regulation of *HDAC1* (Fig. 3e, Supplementary Fig. 3c). The relationships of representative genes and the associated transcriptional regulation factors were listed in Supplementary Fig. 4. These results highlighted that transformation to SCLC phenotype might be promoted by epigenetic alterations.

As described above, ST data revealed different tumor spot clusters in paired LUAD-BT and SCLC-AT samples from the same patient, implying the intratumoral heterogeneity and evolution of different tumor subclones. Thus, pseudotime analysis was performed on tumor spots from paired LUAD-BT and SCLC-AT samples for temporal information of differentiation status during transformation. The pseudotime trajectory was obtained for each patient after calculating the pseudotime value of each tumor spot (Supplementary Fig. 5), and the spatial distribution of tumor spots according to pseudotime values were also shown (Supplementary Fig. 5). The expressions of 137 DEGs identified by bulk RNA data were validated within the pseudotime trajectory, and showed similar results with bulk RNA data: up-regulated DEGs were also up-regulated, and down-regulated DEGs were also down-regulated within the pseudotime trajectory during the differentiation of SCLC cells (Supplementary Fig. 5). The down expression of key regulation factor *HDAC10* was also validated within the pseudotime trajectory (Supplementary Fig. 5). Besides, the expression of *HDAC10* in tumor spots and non-tumor spots were analyzed separately in LUAD-BT and SCLC-AT based on ST data. The median expression of *HDAC10* was down-regulated in tumor spot of SCLC-AT than LUAD-BT (8.58 vs 5.25), not in non-tumor spots (0 vs 0) (Supplementary Fig. 6).

ST data were also used to perform gene set enrichment analysis (GSEA). A total of 1350 genesets associated tumor and TIME were analyzed and summarized into six classes according to the information of these genesets provided on the molecular signatures database (MSigDB), including 421 “Immunity”, 127 “Cell cycle”, 315 “Metabolism & energy”, 286 “Genetic and epigenetic information”, 129 “extracellular matrix (ECM) & metastasis” and 72 “Cell death” genesets. The details of 1350 genesets and their classification according to MSigDB were listed in Supplementary Table 5. The genesets with raw *P* < 0.05 & FDR < 0.25 were considered as statistically significant. Cell cycle-related pathways were more enriched in SCLC-AT, while pathways relating to immunity, cell death, ECM and metastasis were more enriched in LUAD-BT (Fig. 3f, g).

### Changes of pathways related to *EGFR*-TKI resistance during SCLC transformation

Considering SCLC transformation is one of the resistance mechanisms to *EGFR*-TKIs, we investigated the change in genes involved in pathways related to *EGFR*-TKI resistance during transformation. As expected (Supplementary Fig. 7), key genes in *EGF*-*EGFR* pathway showed significant down-regulation after transformation, as well as its downstream signaling pathways (such as *RAS*-*MAPK*, *JAK*-*STAT*). Thus, SCLC-AT no longer depended on *EGFR*-related pathways, indicating *EGFR*-TKI on transformed-SCLC might not be as effective as it was on LUAD. Besides, SCLC-AT demonstrated decreased activity of apoptosis pathway. This observation is consistent with the highly aggressive feature of SCLC-AT. Notably, genes in *FGF*-*FGFR* pathway were significantly up-regulated after transformation, indicating potential therapeutic target for SCLC-AT. Besides, no significant change of *VEGF*-*VEGFR* pathway was observed, suggesting SCLC-AT might still benefit from anti-angiogenic therapy, similar to LUAD. Our results indicated that transcriptomic alterations for evading *EGFR*-TKI occurred not only on the cytomembrane, but also on the intracellular pathways.

### Cell–cell communications between different clusters based on ST data

In the above results, the spatial distribution of tumor spot clusters during transformation in the same patient had been analyzed, and the transcriptomic heterogeneity of tumor spots in LUAD-BT and SCLC-AT samples had been proved. In order to better understanding the changes of multiple signals during transformation, cell–cell communications between different clusters were performed in each sample. Significant ligand-receptor pairs between different clusters in each patient’s LUAD-BT and SCLC-AT samples were shown in Supplementary Fig. 8. Similar to the results of bulk RNA data, it seemed that transformed-SCLCs were less dependent on *EGF* signaling but more relied on *FGF* signaling. Thus, the incoming and outgoing signaling patterns of *EGF* and *FGF* pathways between different clusters in each sample were also shown (Supplementary Fig. 8). We noticed that the tumor spots were mainly contacted through *EGF* signaling before transformation, then switched to *FGF* signaling after transformation.

### Construction and validation of the model to predict SCLC transformation

**Fig. 4:**
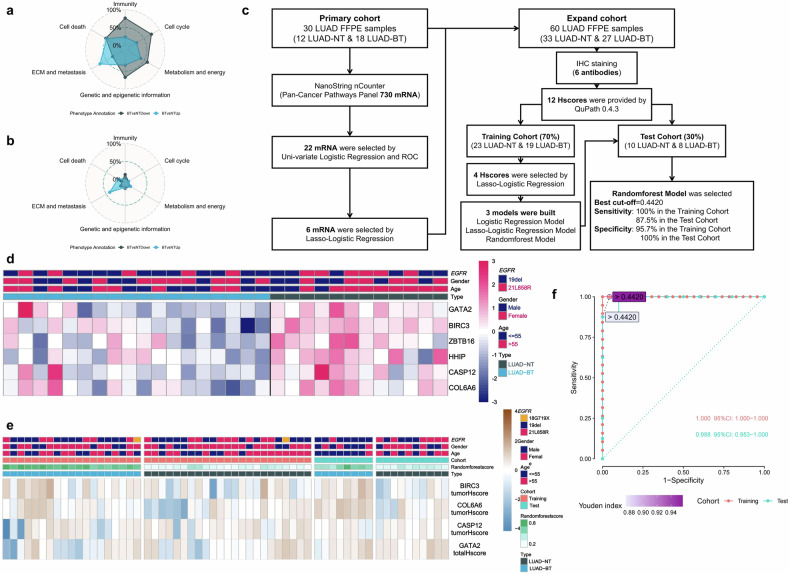
Construction and validation of SCLCtrans Model. **a** GSEA utilizing MSigDB. Radar plot illustrates all 1350 pathways in LUAD-NT vs LUAD-BT. **b** GSEA utilizing MSigDB. Radar plot illustrates pathways statistically significant in LUAD-NT vs LUAD-BT. **c** workflow chart of building SCLCtrans Model. **d** heatmap of 6 RNAs which involved in the model construction showed distinct expression pattern between LUAD-NT and LUAD-BT. **e** heatmap of IHC H-scores for IHC markers in the final SCLCtrans Model in the expanded cohort. **f** ROC curve measuring the performance of SCLCtrans Model in both training and test cohort using Randomforest Model

To reveal the characteristics of enriched pathways in LUAD primed to SCLC transformation, GSEA was performed using ST data of LUAD-NT and LUAD-BT. Immunity-related pathways were more enriched in LUAD-NT, while ECM and metastasis-related pathways were more enriched in LUAD-BT (Fig. 4a, b).

To better screen *EGFR*-mutant LUAD patients with potential SCLC transformation after receiving *EGFR*-TKI, we constructed a model “SCLCtrans Model” (Fig. 4c). RNA expression was binary classified as high-expression and low-expression by each best cut-off value according to receiver operating characteristic (ROC), and 22 RNAs statistically significant in both ROC and univariate Logistic Regression were enrolled in model construction. Using the least absolute shrinkage and selection operator (LASSO)-penalized Logistic Regression analysis, six candidate RNAs were identified based on the optimal value of λ (Supplementary Fig. 9a). The heatmap of these six RNAs also showed distinct expression pattern between LUAD-NT and LUAD-BT (Fig. 4d). Then immunohistochemistry (IHC) of these six candidate RNAs was performed in an expand cohort (*N* = 60, consisting of 33 LUAD-NT and 27 LUAD-BT samples) and H-score of the tumor region and all regions were analyzed for each marker using QuPath-0.4.3, generating 12 H-scores. We applied LASSO-penalized Logistic Regression to select four H-scores (tumor H-score of *BIRC3*, *COL6A6*, *CASP12* and total H-score of *GATA2*, heatmap of the 4 H-scores were shown in Fig. 4e) and trained models using three algorithms (Fig. 4c) in the training cohort (*N* = 42). The Randomforest Model was selected with best performance in training cohort (area under the curve [AUC] = 1.000 [95%CI = 100–100], sensitivity = 100% and specificity = 95.7%), and achieved a sensitivity of 87.5% and a specificity of 100% with an AUC of 0.988 (95%CI = 95.3–100) in the independent test cohort (*N* = 18) (Fig. 4f). The other two models built by Logistic Regression (AUC = 0.817 [95%CI = 68.5–94.9] and 0.938 [95%CI = 81.0–100] in training and test cohort, respectively, Supplementary Fig. 9b) and LASSO-Logistic Regression Model (AUC = 0.803 [95%CI = 66.6–94.1] and 0.938 [95%CI = 81.0–100] in training and test cohort, respectively, Supplementary Fig. 9c) also achieved relatively accurate results.

### Difference between transformed and primary SCLC

**Fig. 5:**
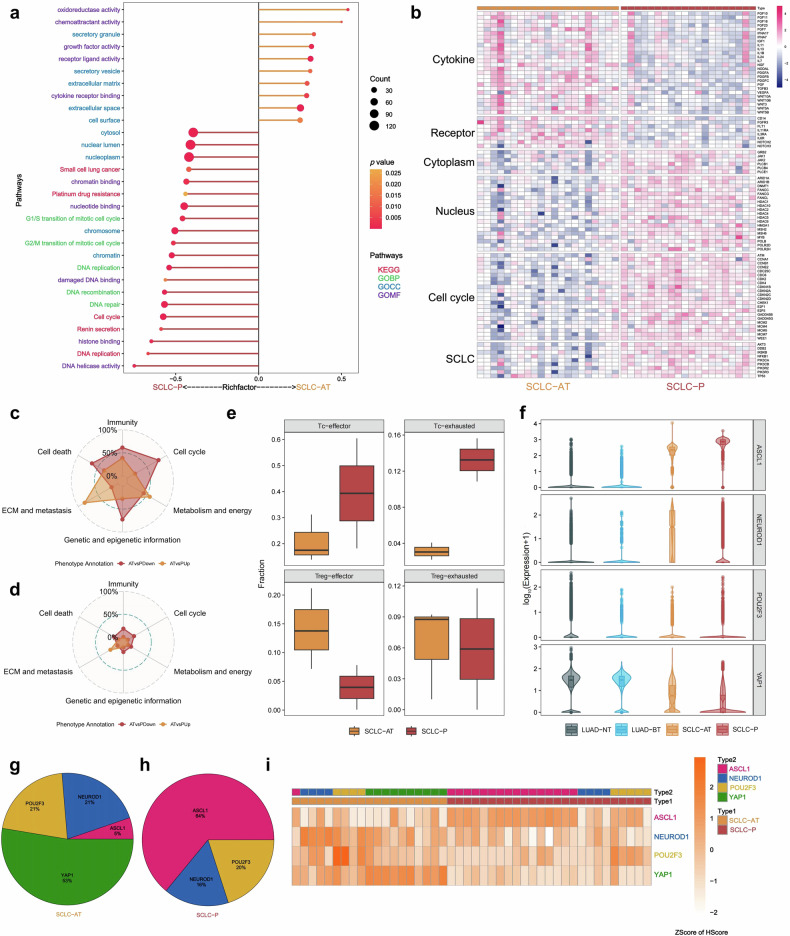
The different molecular characteristics between SCLC-AT and SCLC-P. **a** Pathway enrichment analyses on the DEGs from bulk RNA data of SCLC-AT versus SCLC-P. **b** Heatmap highlighting DEGs of interest grouped by associated pathways (bulk RNA data). **c** GSEA utilizing MSigDB. Radar plot illustrates all 1350 pathways in SCLC-AT vs SCLC-P. **d** GSEA utilizing MSigDB. Radar plot illustrates pathways statistically significant in SCLC-AT vs SCLC-P. **e** Comparison of Tc and Treg infiltration fractions between SCLC-AT and SCLC-P according to mIF. **f** The expression of 4 transcription factors (*ASCL1*, *NEUROD1*, *POU2F3*, and *YAP1*) in four groups of samples according to ST data. **g**, **h** The proportion of different molecular subtypes in SCLC-AT and SCLC-P. **i** IHC heatmap of four molecular subtype makers’ expressions (*ASCL1*, *NEUROD1*, *POU2F3*, and *YAP1*) after z-score normalization

To explore molecular differences between SCLC-AT and SCLC-P, firstly, we performed pathway enrichment analyses. Comparison of the bulk transcriptomic data revealed that SCLC-AT showed more activation of cytomembranous and extracellular pathways than SCLC-P, such as cytokine and receptor-related pathways resembling LUADs (Fig. 5a, b). Besides, SCLC-AT also showed down-regulations in genes associated with cell cycle, SCLC and DNA repair (Fig. 5a, b). Secondly, GSEA was performed using ST data showed that pathways related to ECM and metastasis were enriched in SCLC-AT, while cell cycle, immunity, cell death-related pathways were enriched in SCLC-P (Fig. 5c, d), in an echo of the results from bulk RNA analysis.

To further investigate the difference of TIME between SCLC-AT and SCLC-P, different immune-related cell types were calculated using mIF. Compared with SCLC-P, SCLC-AT had lower infiltration fraction of Tc (including Tc-effector and Tc-exhausted), and higher infiltration fraction of Treg-effector (Fig. 5e), indicating a relatively unfavorable TIME for immune checkpoint inhibitor (ICI) than SCLC-P. Moreover, we further investigated the association between TIME and four transcription factors in SCLCs.

Previous studies have highlighted molecular subtypes of primary SCLC based on the expression of four transcription factors: *ASCL1* (Subtype-A), *NEUROD1* (Subtype-N), *POU2F3* (Subtype-P), and *YAP1* (Subtype-Y).^34^ Thus, we analyzed the difference of molecular subtypes between SCLC-AT and SCLC-P. The comparison of RNA expression of four transcription factors according to ST data revealed that *ASCL1* was high expressed in two SCLC groups, and *YAP1* was high expressed in two LUAD groups. To be noticed, *ASCL1* expression in SCLC-AT ranged in between LUAD-BT and SCLC-P, as well as *YAP1* (Fig. 5f). Furthermore, the expressions of *ASCL1*, *NEUROD1*, *POU2F3*, and *YAP1* were evaluated in 21 SCLC-AT and 25 SCLC-P at protein levels by IHC (representative IHC staining in Supplementary Fig. 10). The proportion of molecular subtypes in SCLC-AT and SCLC-P were shown in Fig. 5g, h, and Fig. 5i showed the heatmap of four makers expressions after z-score normalization (2 SCLC-AT samples were insufficient for IHC, thus 19 SCLC-AT samples had the molecular subtype results). Similar to previous reported, most SCLC-P were Subtype-A (64%, 16/25), followed by Subtype-P (20%, 5/25) and Subtype-N (16%, 4/25). Interestingly, the subtype proportion of SCLC-AT was markedly different: Subtype-Y was most dominant with 53% (10/19), followed by Subtype-P (21%, 4/19) and Subtype-N (21%, 4/19). Subtype-A, which was most frequently seen in SCLC-P, accounted for only 5% (1/19) of SCLC-AT. Supplementary Fig. 11 showed the heatmap according to the Spearman’s correlation coefficients between fractions of different immune-related cell types and expressions of transcription factors. Although the relationship was not significant, it seemed that higher expression of *ASCL1* was associated with higher infiltration fraction of Tc-exhausted; while higher expression of *POU2F3* was associated with higher infiltration fraction of Tc-effector.

### Changes of TIME characteristic during transformation

**Fig. 6:**
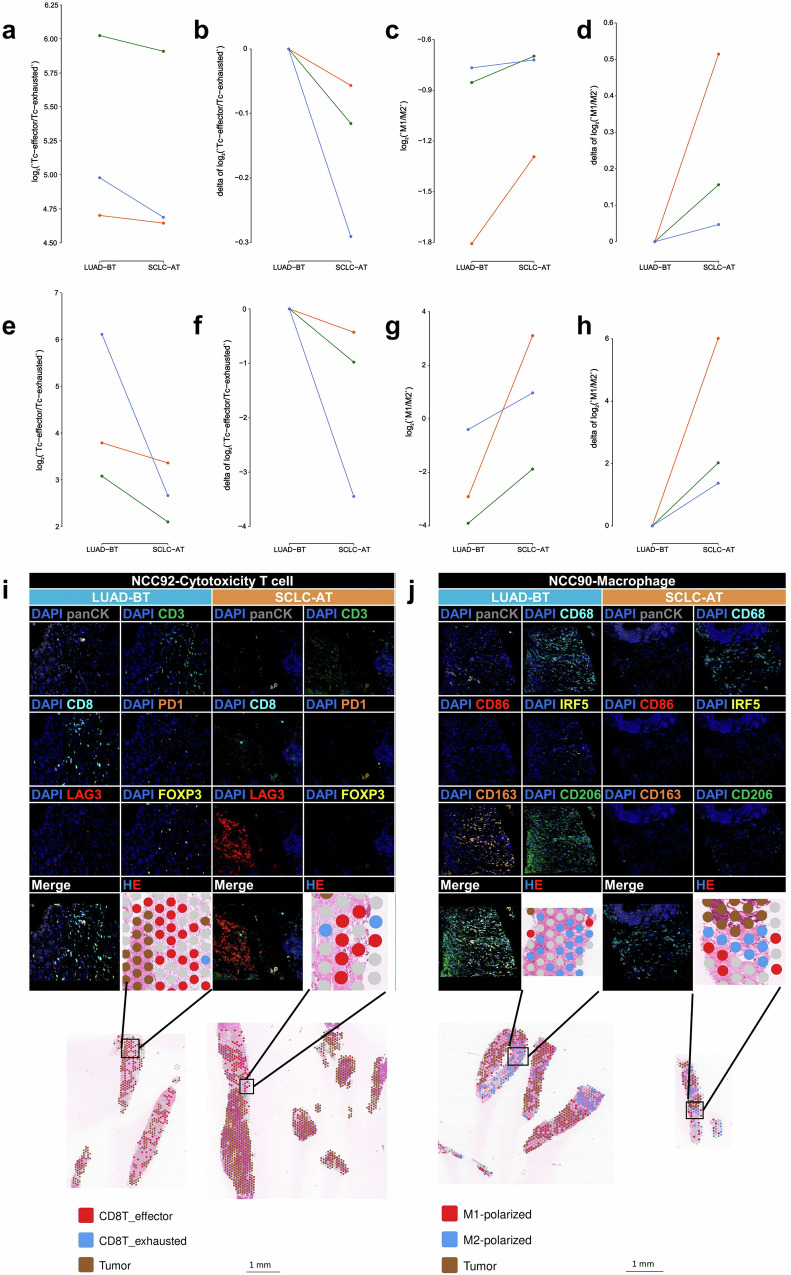
Changes of T cells and macrophages during transformation. **a**, **b** Absolute values and relative changes of Tc-effector to Tc-exhausted ratio based on the algorithm-simulated TIME. **c**, **d** Absolute values and relative changes of M1 to M2 ratio based on the algorithm-simulated TIME. **e**, **f** Absolute values and relative changes of Tc-effector to Tc-exhausted ratio based on the mIF results. **g**, **h** Absolute values and relative changes of M1 to M2 ratio based on the mIF results (**a**–**h**: orange line, TTT = 10.75 months; green line, TTT = 30.23 months; blue line, TTT = 58.98 months, corresponded to Patient No. NCC90, NCC91, NCC92, respectively). **i** Examples of multi-channel mIF images from the T cell panel and their corresponding algorithm-simulated results annotated HE images in paired LUAD-BT and SCLC-AT samples (Patient No. NCC92). **j** Examples of multi-channel mIF images from the macrophage panel and their corresponding algorithm-simulated results annotated HE images in paired LUAD-BT and SCLC-AT samples (Patient No. NCC90)

To explore the changes of TIME characteristic during transformation, the algorithm-simulated TIME based on ST data were performed. The Tc-effector/Tc-exhausted ratio was decreased during transformation in three patients with ST data, and the changes had positive correlation with the TTT (Fig. 6a, b). In terms of the M1/M2 ratio, increasing changes had negatively correlation with the TTT (Fig. 6c, d). To validate above observation of TIME during transformation, mIF was performed and the tendency of the two ratios were in consist with that algorithm-simulated (Fig. 6e–h). Examples of multi-channel mIF images and their corresponding algorithm-simulated results annotated hematoxylin-eosin (HE) images from the T cell panel (Fig. 6i) and from the macrophage panel (Fig. 6j) in paired LUAD-BT and SCLC-AT samples were provided.

### Clinical decision system

To better understand the prognosis of transformed-SCLCs, two models to predict TTT and OStrans of transformed-SCLCs were constructed using bulk RNA data. For TTT prognostic model, eight genes were selected (Supplementary Fig. 12a). TTT model showed independent predictive value when the gender and *EGFR*-sensitizing mutation type were controlled (Supplementary Table 6, Supplementary Fig. 12b–d). In terms of OS after transformation (OStrans), nine genes were selected (Supplementary Fig. 13a). OStrans model showed independent predictive value in transformed-SCLCs (Supplementary Table 7, Supplementary Fig. 13b–e). The predictive efficacy of OStrans Model was also verified in SCLC-P patients (Supplementary Fig. 13f).

**Fig. 7:**
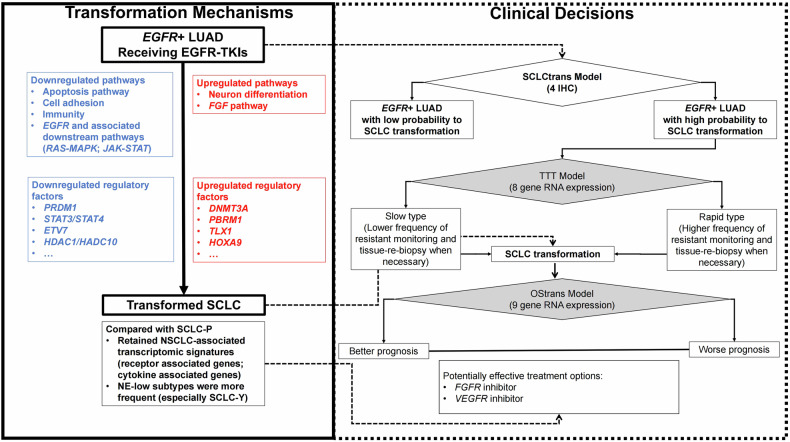
Molecular mechanism for SCLC transformation and whole-course seamless management of *EGFR*-mutant LUADs with potential transformation. *EGFR* epidermal growth factor receptor, *FGFR* fibroblast growth factor receptor, LUAD lung adenocarcinoma, NSCLC non-small cell lung cancer, OS overall survival, SCLC small cell lung cancer, TTT time to transformation, *VEGFR* vascular endothelial growth factor receptor

Thus, based on the molecular mechanisms of SCLC transformation and three models (SCLCtrans Model, TTT Model, and OStrans Model), the whole-course seamless management of *EGFR*-mutant LUADs with potential transformation was concluded in Fig. 7.

## Discussion

SCLC transformation was mainly reported in *EGFR*-TKI-relapsed patients. Identification of the molecular mechanisms, exploration of optimal treatment choices and prognostic implications for SCLC transformation are of great importance. Most studies of SCLC transformation focused on the genetic alterations, but transcriptomic features have been proven to associate with histologic differences as late molecular events rather than genomic alterations in lung cancers.^16^ Thus investigation of transcriptomic characteristics might be the key to exploration for histological transformation, which was relatively lack of research in this field, mainly due to the difficulty of accessing the paired pre-/post-transformation clinical samples. Besides, several studies have made great efforts to explore the mechanisms of histological transformation from LUAD to SCLC, or to squamous cell carcinoma, using in vitro experimental methods such as lentivirus infection cell lines^20,21^ or mouse models,^22,23^ and received important results. However, exploratory study using tumor tissues from the real world patients can reflect the actual mechanisms of transformation in human body, and can not be replaced by in vitro or mouse model studies. Furthermore, with the usage of immunotherapy in primary SCLCs, it’s an urgent to investigate the TIME characteristics of transformed-SCLCs and clarify whether transformed-SCLCs can benefit from immunotherapy. The strengths of our study lie in the following aspects. Firstly, to our knowledge, this is the first study to elaborate the transformation mechanisms focused on transcriptomic characteristics, including bulk RNA and ST data; while we also compared the TIME characteristics during transformation. Secondly, our study has the largest sample size to date with 27 pre-transformed and 24 post-transformed samples (including 15 paired samples). Thirdly, our study innovatively enrolled never-transformed LUAD patients to construct a model to predict SCLC transformation. Besides, we also compared the difference of molecular characteristics, subtypes, and TIME between transformed-SCLCs and stage-matched primary SCLCs, which could indicate the choice of potential treatment strategies for transformed-SCLCs.

In our study, the gene expression pattern revealed that both LUAD-BT and SCLC-AT shared overlaps with their non-transforming histology, as well as substantial overlap with each other. Besides, spatial distribution of tumor spot clusters from the same patient revealed transformed-SCLC cells were evolved from subgroups of LUAD cells, indicating the existence of certain tumor subgroups with lineage plasticity before transformation. These observations indicated that LUAD-BT were primed to transformation before histological transformation appeared, while SCLC-AT showed transcriptomic features of both LUAD-BT and SCLC-P with an intermediate phenotype. This conclusion also can be proved by the comparison of transcriptomic features of SCLC-AT and SCLC-P: SCLC-AT retained RNA expression features of the LUAD from which they derived, such as activation on pathways associated with cytokine and receptor, resembling with LUAD. Same results were reported in spatial mixed histology LUAD/SCLC samples.^18^

In order to explore whether the drivers of SCLC transformation are within tumor cells or within the tumor microenvironment, the ST data of tumor spots and non-tumor spots were submitted to UMAP analysis separately. We noticed that tumor spots from LUAD-BT and SCLC-AT were separated in some extent, while non-tumor spots from LUAD-BT and SCLC-AT overlapped, indicating the changes of tumor cells were more dramatic than the changes of tumor microenvironment during transformation. Besides, weighted regulation network revealed that *HDAC10* was the key regulation factor which was down-regulated during transformation; while in ST analysis the expression of *HDAC10* was proved to be down-regulated within the pseudotime trajectory. Based on separately analysis of tumor spots and non-tumor spots with ST data, we also proved that the median expression of *HDAC10* in tumor spots was down-regulated in tumor spot of SCLC-AT than LUAD-BT, not in non-tumor spots. These results indicated that the key driver of transformation are within tumor cells instead of within the tumor microenvironment.

By comparison of LUAD-BT and SCLC-AT, our study pointed out a number of pathways shifted during transformation, including down-regulation of NSCLC-related pathways, apoptosis, cell adhesion, and up-regulation of neural differentiation pathways. This result consisted with clinical behavior of transformed-SCLC, such as high proliferative ability, small round-cell morphology, and neuroendocrine features. Previous studies reported SCLC transformation was related to down-regulation of Notch signaling.^18^ Our results also suggested significant up-regulation of genes associated to Notch signaling inhibition, such as *DLL3* (*P* < 0.001) and *HES5* (*P* = 0.022), indicating Notch signaling down-regulation during transformation. Notch signaling was known to play a suppressing role in neuroendocrine tumor growth,^35^ down-regulation of Notch signaling has been reported in neuroendocrine plasticity dynamics in both prostate cancer and NSCLC.^36^ WNT signaling has been reported to implicate in lineage plasticity of basal cell carcinoma.^37^ Previous study reported β-catenin was significantly increased in the SCLC components than LUAD components in spatial mixed samples.^18^ However, in 12 paired pre-/post-transformation samples in our study, the RNA expression level of β-catenin (*CTNNB1*) was up-regulated in only one patients. Consistent with our result, in neuroendocrine transformation of prostate cancer, WNT was activated without activating β-catenin.^38^ Thus, the role of WNT signaling and β-catenin in transformation need to be further investigated. Besides, PRC2 complex was proved to be up-regulated in SCLC components by *EZH2*,^18^ which was also up-regulated during transformation in our study. PRC2 complex was implicated with lineage plasticity in prostate cancer,^39^ and inhibiting *CREB*/*EZH2* axis could repress the growth of neuroendocrine differentiated prostate cancer.^39^ The up-regulation of *EZH2* provided potential treatment target to prevent SCLC transformation. Moreover, we found 17.5% of DEGs were transcriptional regulation factors, and pointed out several key regulation factors involved in transformation. Besides, weighted regulation network also showed that key regulation factors played important roles during transformation, such as *HDAC10*, *STAT3*, and *DNMT3A*. These results highlighted that SCLC transformation was mainly dependent on epigenetic shifts as previous study.^18^

For druggable pathways, transformed-SCLC were proved to be less dependent on *EGF* signaling but more relied on *FGF* signaling in both bulk RNA and ST data. These results indicated that *EGFR*-TKI alone for SCLC-AT might not be as effective as LUAD-BT, and combination of *EGFR*-TKI and other anti-cancer treatment might be necessary; but *FGF* signaling might be a proper choice of potential therapeutic target. Recent study also reported *FGF9* up-regulation at protein level in transformed-SCLC, and pan-*FGFR* inhibitor AZD4547 could inhibit transdifferentiated SCLC-like tumors growth.^40^ Besides, *VEGF*-*VEGFR* pathway remained actively after transformation, indicating anti-angiogenic therapies might be still effective for transformed-SCLC. In fact, our previous study also proved that transformed-SCLCs could not benefit from the combination of chemotherapy and *EGFR*-TKIs in OStrans; while anti-angiogenic therapies could significantly prolong OStrans.^26^

To be noticed, compared with LUAD-BT or SCLC-P, “Immune” pathways were less enriched in SCLC-AT in transcriptomic level. Besides, TIME analysis using mIF indicated lower Tc infiltration fraction (including Tc-effector and Tc-exhausted) and higher Treg-effector infiltration fraction in SCLC-AT than SCLC-P; while comparison of LUAD-BT and SCLC-AT revealed that the longer TTT, the more drastically reduced Tc-effector/Tc-exhausted ratio was. These results indicated an immuno-exhausted status (higher PD1^+^ and/or LAG3^+^ Tc cells infiltration fraction) in transformed-SCLCs which was associated with the duration of *EGFR*-TKI before transformation. Previous studies reported that PD1/LAG3 co-expression in T cells was related to immunotherapy resistance.^41,42^ However, co-blockade of PD1 and LAG3 could revert T cell proliferative dysfunctionality,^43^ which have been proved to have a remarkable benefit in vivo and in vitro.^44,45^ Thus, bispecific antibodies for LAG3 and PD1, such as IMP321+pembrolizumab, relatlimab+nivolumab, or tebotelimab (PD-1/LAG-3 bispecific antibody), might show potential prospect in treatment of transformed-SCLCs.

SCLCs can be divided into discrete molecular subtypes based on four key transcription factors: *ASCL1*, *NEUROD1*, *POU2F3*, and *YAP1*.^28^ In our study, SCLC-AT showed distinct subtype composition compared with SCLC-P by IHC, which was proved to reached highly agreement with RNA.^34^ Subtype-Y was the most dominant subtype in SCLC-AT, which was rarely seen in SCLC-P. *YAP1* played the oncogenic role as Hippo pathway effector^46^ and associated with LUAD tumorigenesis.^47^ Subtype-Y was also associated with non-neuroendocrine SCLCs.^48^ The high proportion of Subtype-Y in SCLC-AT was another evidence that transformed-SCLC retained transcriptomic features of LUAD. Subtype-Y was also reported to be associated with elevated T cell-inflamed gene expression profile and innate immune cell signatures,^49^ and benefit more from immunotherapy than Subtype-A or Subtype-N.^50^ The molecular subtypes might have application in the choice of potential treatment after transformation.

Till now, effective models of prediction and prognosis for transformed-SCLCs remain urgent and unmet needs. Here we constructed a model to predict SCLC transformation (SCLCtrans Model) with 4-IHC markers (tumor H-score of *BIRC3*, *COL6A6*, *CASP12*, and total H-score of *GATA2*), which was proved to have a remarkable prediction ability in both training and test cohorts. *BIRC3* is an inhibitor of apoptosis protein with established canonical anti-apoptosis function through inhibition of caspase activation,^51^ and associated with stemness reprogramming in glioblastoma.^52^ *COL6A6* (Collagen Type VI alpha 6 chain) acts a vital role in the ECM and may regulate epithelial cell-fibronectin interactions and participate in cell adhesion.^53^ *CASP12* is a cysteine protease, and the inhibition of *CASP12* activity is associated with rapid proliferation in several tumors.^54^ The zinc-finger transcription factor *GATA2* plays an essential role in chemotherapy resistance of gastric cancer,^55^ and promotes castration-resistant prostate cancer development.^56^ We achieved a sensitivity of 87.5% and a specificity of 100% in an independent test cohort in distinguishing primed-to-transform LUADs from never transformed. Our SCLCtrans Model, based on IHC results of 4 markers, is a relatively easy, inexpensive technique for clinical routine testing.

Furthermore, we developed the 8-gene TTT Model and 9-gene OStrans Model to predict TTT and OStrans for LUADs with SCLC transformation. Moreover, as RNA expression pattern of SCLC-AT shared substantial overlap with SCLC-P, samples in SCLC-P were used to verify the OStrans Model. Primary SCLC with higher OStrans score did have poorer 2-year, 3-year, and 4-year OS rate than those with lower score. These two models could be helpful for patients whole-course management.

In future, we will carry out a large-scale prospective study in multicenters, and validate our SCLCtrans Model in a larger sample size. We will also focus on the validation study of transformation mechanisms and potential treatment strategies using patient-derived organoids based on the prospective study.

This study used pre-/post-transformation samples, stage-matching LUAD-NT and SCLC-P samples, to provide insights into the molecular mechanisms and potential clinical decisions of SCLC transformation in *EGFR*-mutant LUADs. We described the signaling pathways and the key transcriptional regulation factors functioned for transformation, which might shed light on the prevention strategy for SCLC transformation in the future. We also constructed models for the whole-course management of *EGFR*-mutant LUADs with potential SCLC transformation, and proposed potential treatment strategies for SCLC transformation.

## Materials and methods

### Study design

We retrospectively collected 40 advanced *EGFR*-mutated LUADs with SCLC transformation (including 27 LUAD-BT and 24 SCLC-AT samples), 33 LUAD-NT, and 27 SCLC-P (enrolled criteria shown in Supplementary material 2). Clinicopathological data were obtained from clinical records. This study was approved by the institutional review board of the Cancer Hospital, Chinese Academy of Medical Science (No. 21/243-2914), and was conducted in accordance with the Declaration of Helsinki.

### RNA extraction and bulk RNA analysis

HE slides of the enrolled patients were reviewed, and RNA was extracted using an RNeasy FFPE Kit (Qiagen, CA, USA). Systematic RNA expression was measured on an nCounter FLEX Analysis System (NanoString, Seattle, WA, USA) using the nCounter Pan-Cancer Pathways gene expression panel (NanoString Technologies Inc.), covering 730 human RNAs associated with cancer-associated pathways (details shown in Supplementary material 3).

### Expression pattern and DEG analysis

Two methods were used to compare RNA expression patterns: the Spearman’s correlation between each two groups according to the median expression level of RNAs, and the number and proportion of DEGs through the Wilcoxon rank-sum test between each two groups. The *P* were adjusted by Benjamini & Hochberg’s method to control FDR.

### Pathway enrichment analysis

Pathway enrichment analysis was performed on the up-regulated and down-regulated DEGs based on bulk RNA data. GSEA was performed based on ST data (details shown in Supplementary material 4).

### Weighted regulation network analysis during transformation

The average value of Spearman’s correlation coefficients in both LUAD-BT and SCLC-AT samples were calculated to evaluate the potential regulatory relationship during transformation between significantly changed regulator genes and other DEGs (regulated genes). A weighted regulatory network was constructed (details shown in Supplementary material 5).

### ST sequencing and analysis

5 μm FFPE slides were prepared and incubated followed by HE staining. Spatial RNA expression was processed according to the Visium Spatial Gene Expression User Guide (10 × Genomics, User Guide CG000407 Rev C, human transcriptome product number 1000338) and all reagents were obtained from the Visium Spatial Gene Expression for FFPE Reagent Kit (10 × Genomics). Batch effect correction, dimensionality reduction, and cluster analysis were conducted, then cell–cell communication analysis was performed among different clusters. Pseudotime analysis was performed in tumor spots from paired LUAD-BT and SCLC-AT samples. For TIME analysis, tumor spots were identified based on HE-stained slides by Loupe Browser 6 (https://support.10xgenomics.com/single-cell-gene-expression/software/visualization/latest/what-is-loupe-cell-browser) and “estimate” algorithm.^33^ Then the quanTIseq algorithm was used on the non-tumor spots to calculate the infiltration fractions of 10 types of immune cells. Details were shown in Supplementary material 6.

### IHC for SCLC molecular subtypes

IHC of *ASCL1*, *NEUROD1*, *POU2F3*, and *YAP1* was performed on 21 SCLC-AT and 25 SCLC-P samples according to the manufacturer’s instructions. The H-scores were evaluated by two independent pathologists who were blinded to the paitnets (YL and LY). H-scores were standardized using z-score normalization. Molecular subtype was determined according to the highest z-score of the sample (details shown in Supplementary material 7).

### Follow-up and survival analysis

Overall survival (OS) for SCLC-P was defined as the period from the date of diagnosis to death of any cause. TTT was defined as the period from first-line treatment for advanced disease to the confirmation of SCLC diagnosis. OStrans was defined as the period from the confirmation of SCLC diagnosis to death of any cause. The last follow-up date was Sep 30th, 2023. Survival analysis was performed by Kaplan–Meier (KM) curves and Cox proportional hazard model.

### Construction of a model to predict SCLC transformation and IHC validation

ROC curves were used to generate binary variables using bulk RNA data of LUAD-NT and LUAD-BT. RNAs with statistical significance in both ROC and univariate Logistic Regression analyses were used through LASSO-penalized Logistic Regression. Then IHC staining of the candidate RNAs were performed in expanded cohort (*N* = 60). We applied LASSO-Logistic to screen IHC markers and trained models using three algorithms to discriminate the LUAD-BT from LUAD-NT in the training cohort (*N* = 42). The performance of these models was evaluated in the test cohort (*N* = 18) using ROC (details shown in Supplementary material 8).

### Construction of models to predict TTT and OStrans

KM analysis was used to generate binary variables using bulk RNA data of LUAD-BT (for TTT Model) and SCLC-AT (for OStrans Model). RNAs with statistical significance were used in model construction through LASSO-penalized Cox regression (details shown in Supplementary material 9).

### mIF

Four mIF panels were developed to evaluate macrophages, T lymphocytes, cancer-associated fibroblasts (CAFs), dendritic cells (DCs) and B cells, respectively. The cell subtypes, including M1/M2-polarized macrophages, Tc-effector, Tc-exhausted, Treg-effector, Treg-exhausted, T8reg, type I/II/III CAFs (CAFI/II/III),^57^ Be/Breg,^58^ and classic/monocyte-derived/plasmacytoid DCs (cDCs, mDCs, pDCs),^59^ were identified according to the combination of different channels in each panel. The details are shown in Supplementary material 10.

### Statistics

R 3.6.2 (https://www.r-project.org/) was used for our statistical analysis. We used Wilcoxon rank-sum test for continuous variables and rank variables, Wilcoxon signed-rank test for paired data. Spearman’s correlation was used for continuous variables. Fisher’s exact test was used for unordered categorical variables. Raw *P* < 0.05 and FDR < 0.25 simultaneously were considered statistically significant.

## Supplementary information

Supplementary Materials

## Data Availability

Bulk and spatial transcriptomic data reported in this article have been deposited in the OMIX (OMIX007321, https://ngdc.cncb.ac.cn/omix/) and GSA-Human (GSA-Human: HRA008517, https://ngdc.cncb.ac.cn/gsa-human) database of National Genomics Data Center, China National Center for Bioinformation, respectively. Any information required to reanalyze the data reported in this article is available from Puyuan Xing (xingpuyuan@cicams.ac.cn) upon request.
