# Supplementary material for: Mechanism exploration and model construction for small cell transformation in EGFR-mutant lung adenocarcinomas
Source: Signal Transduct Target Ther. 2024 Oct 2;9:261. doi: 10.1038/s41392-024-01981-3 (PMC11445518; doi:10.1038/s41392-024-01981-3)
Supplement: Supplementary file 1 — Supplementary Materials [file 41392_2024_1981_MOESM1_ESM.docx]

Supplementary Materials for

Mechanism exploration and model construction for small cell transformation in EGFR-mutant lung adenocarcinomas

Yan Li, Tongji Xie, Shouzheng Wang, Lin Yang, Xuezhi Hao, Yan Wang, Xingsheng Hu, Lin Wang, Junling Li, Jianming Ying, Puyuan Xing

Correspondence to:xingpuyuan@cicams.ac.cn & jmying@cicams.ac.cn.

**This PDF file includes:**

Materials and Methods

Supplementary Text (Supplementary Materials 1-10)

Figures. S1 to S13

Tables S1 to S7

Materials and Methods

Study design

We retrospectively collected 40 advanced *EGFR*-mutated LUADs with SCLC transformation (including 27 LUAD-BT and 24 SCLC-AT samples), 33 LUAD-NT, and 27 SCLC-P (enrolled criteria shown in Supplementary material 2). Clinicopathological data were obtained from clinical records. This study was approved by the institutional review board of the Cancer Hospital, Chinese Academy of Medical Science (No.21/243-2914), and was conducted in accordance with the Declaration of Helsinki.

RNA extraction and bulk RNA analysis

HE slides of the enrolled patients were reviewed, and RNA was extracted using an RNeasy FFPE Kit (Qiagen, CA, USA). Systematic RNA expression was measured on an nCounter FLEX Analysis System (NanoString, Seattle, WA, USA) using the nCounter Pan-Cancer Pathways gene expression panel (NanoString Technologies Inc.), covering 730 human RNAs associated with cancer-associated pathways (details shown in Supplementary material 3).

Expression pattern and DEG analysis

Two methods were used to compare RNA expression patterns: the Spearman’s correlation between each two groups according to the median expression level of RNAs, and the number and proportion of DEGs through the Wilcoxon rank-sum test between each two groups. The P were adjusted by Benjamini & Hochberg’s method to control FDR.

Pathway enrichment analysis

Pathway enrichment analysis was performed on the up-regulated and down-regulated DEGs based on bulk RNA data. GSEA was performed based on ST data. (details shown in Supplementary material 4).

Weighted regulation network analysis during transformation

The average value of Spearman’s correlation coefficients in both LUAD-BT and SCLC-AT samples were calculated to evaluate the potential regulatory relationship during transformation between significantly changed regulator genes and other DEGs (regulated genes). A weighted regulatory network was constructed (details shown in Supplementary material 5).

ST sequencing and analysis

5μm FFPE slides were prepared and incubated followed by HE staining. Spatial RNA expression was processed according to the Visium Spatial Gene Expression User Guide (10×Genomics, User Guide CG000407 Rev C, human transcriptome product number 1000338) and all reagents were obtained from the Visium Spatial Gene Expression for FFPE Reagent Kit (10×Genomics). Batch effect correction, dimensionality reduction, and cluster analysis were conducted, then cell-cell communication analysis was performed among different clusters. Pseudotime analysis was performed in tumor spots from paired LUAD-BT and SCLC-AT samples. For TIME analysis, tumor spots were identified based on HE -stained slides by Loupe Browser 6 (https://support.10xgenomics.com/single-cell-gene-expression/software/visualization/latest/what-is-loupe-cell-browser) and “estimate” algorithm23. Then the quanTIseq algorithm was used on the non-tumor spots to calculate the infiltration fractions of 10 types of immune cells. Details were shown in Supplementary material 6.

IHC for SCLC molecular subtypes

IHC of *ASCL1*, *NEUROD1*, *POU2F3* and *YAP1* was performed on 21 SCLC-AT and 25 SCLC-P samples according to the manufacturer’s instructions. The H-scores were evaluated by two independent pathologists who were blinded to the paitnets (YL and LY). H-scores were standardized using z-score normalization. Molecular subtype was determined according to the highest z-score of the sample (details shown in Supplementary material 7).

Follow-up and survival analysis

Overall survival (OS) for SCLC-P was defined as the period from the date of diagnosis to death of any cause. TTT was defined as the period from first-line treatment for advanced disease to the confirmation of SCLC diagnosis. OStrans was defined as the period from the confirmation of SCLC diagnosis to death of any cause. The last follow-up date was Sep 30th, 2023. Survival analysis was performed by Kaplan-Meier (KM) curves and Cox proportional hazard model.

Construction of a model to predict SCLC transformation and IHC validation

ROC curves were used to generate binary variables using bulk RNA data of LUAD-NT and LUAD-BT. RNAs with statistical significance in both ROC and univariate Logistic Regression analyses were used through LASSO-penalized Logistic Regression. Then IHC staining of the candidate RNAs were performed in expanded cohort (N=60). We applied LASSO-Logistic to screen IHC markers and trained models using three algorithms to discriminate the LUAD-BT from LUAD-NT in the training cohort (N=42). The performance of these models was evaluated in the test cohort (N=18) using ROC (details shown in Supplementary material 8).

Construction of models to predict TTT and OStrans

KM analysis was used to generate binary variables using bulk RNA data of LUAD-BT (for TTT Model) and SCLC-AT (for OStrans Model). RNAs with statistical significance were used in model construction through LASSO-penalized Cox regression. (details shown in Supplementary material 9).

mIF

Four mIF panels were developed to evaluate macrophages, T lymphocytes, cancer associated fibroblasts (CAFs), dendritic cells (DCs) and B cells, respectively. The cell subtypes, including M1/M2-polarized macrophages, Tc-effector, Tc-exhausted, Treg-effector, Treg-exhausted, T8reg, type I/II/III CAFs (CAFI/II/III)55, Be/Breg56, and classic/monocyte-derived/plasmacytoid DCs (cDCs, mDCs, pDCs)57, were identified according to the combination of different channels in each panel. The details are shown in Supplementary material 10.

Statistics

R 3.6.2 (https://www.r-project.org/) was used for our statistical analysis. We used Wilcoxon rank-sum test for continuous variables and rank variables, Wilcoxon signed-rank test for paired data. Spearman’s correlation was used for continuous variables. Fisher’s exact test was used for unordered categorical variables. Raw P<0.05 and FDR<0.25 simultaneously were considered statistically significant.

Supplementary Text

Supplementary material 1: Influence of different *EGFR* mutant type on SCLC transformation mechanisms

We also investigate whether *EGFR* mutant type (19del and L858R) could influence the transformation mechanisms by comparing the amplitude of weighted average up-/down-regulated genes expression (Δ weighted average up-/down-regulated genes expression=weighted average up-/down-regulated genes expression in SCLC-AT sample - weighted average up-/down-regulated genes expression in paired LUAD-BT sample) of 12 patients with both LUAD-BT and SCLC-AT samples. Although the number of patients harboring L858R was relatively small, it seemed that patients harboring L858R were more relied on the up-regulation of neural differentiation, while patients harboring 19del were regulated by both up-regulation of neural differentiation and down-regulation of other mechanisms such as NSCLC, apoptosis, cell adhesion, and immunity.

Supplementary material 2: Enrolled criteria

LUAD-BT and SCLC-AT:

We retrospectively collected 40 advanced *EGFR*-mutated LUAD patients with SCLC transformation diagnosed from Oct 2015 to Jul 2023, with criteria as follows: 1) receiving *EGFR*-TKIs at advanced stage of LUAD; 2) re-biopsy showed SCLC transformation after *EGFR*-TKI resistance. Among 40 SCLC-transformation LUAD patients, 36 patients had pre- or/and post-transformation tissues for transcriptome analysis, including 27 LUAD samples before transformation (LUAD-BT) and 24 SCLC after transformation samples (SCLC-AT) (15 patients had paired LUAD-BT and SCLC-AT samples).

LUAD-NT:

A group of *EGFR*-mutant LUAD-NT patients (N=33) diagnosed from Aug 2011 to Sep 2019 were enrolled with criteria as follows: 1) receiving *EGFR*-TKIs at advanced stage of LUAD; 2) the time from *EGFR*-TKI treatment to death/last follow-up was longer than the median TTT of the 40 SCLC-transformed LUADs enrolled in this study (which was 28.4 months); 3) received at least one re-biopsy after *EGFR*-TKIs therapy and no histological transformation were observed; 4) Formalin-Fixed Paraffin-Embedded (FFPE) samples accessible.

SCLC-P:

Furthermore, stage Ⅲ~Ⅳ SCLC-P patients (N=27) from May 2017 to Apr 2022 were also enrolled with criteria as follows: 1) primary SCLC with no histotry of other tumors; 2) FFPE sample accessible.

All the tumor specimens were FFPE samples obtained from the tissue bank of the Department of Pathology, Cancer Hospital, CAMS.

Supplementary material 3: Bulk RNA extraction and data processing

HE slides of the enrolled patients were reviewed, and RNA was extracted using RNeasy FFPE Kit (Qiagen, CA, USA). The quantities of RNA were determined using a Qubit 3.0 Fluorometer (Thermo Fisher Scientific, Carlsbad, CA, USA). RNA fragmentation and quality were determined by 2100 Bioanalyzer (Agilent, CA, USA).

Systematic RNA expression was measured on an nCounter FLEX Analysis System (NanoString, Seattle, WA, USA) using the nCounter Pan-Cancer Pathways gene expression panel (NanoString technologies Inc.), covering 730 human RNAs associated to cancer-associated pathways. A minimum input of 300 ng of total RNA was used for each sample. Fluorescently color-coded reporter probes and biotin-labelled capture probes were hybridized to the RNA on a thermal cycler overnight and automatically processed and loaded to the NanoString provided sample cartridge in the nCounter Prep Station in accordance with the manufacturer’s protocol.

Raw counts resulting from the analysis were normalized against reference genes through NanoString’s nSolver 4.0, genes selected to have the least variance with the geNorm algorithm. Normalized data were analyzed using R 3.6.2.

Supplementary material 4: Pathway enrichment analysis

On bulk level, Wilcoxon rank-sum test (for unpaired samples) and Wilcoxon sign-rank test (for paired samples) were performed to investigate DEGs. DEGs were defined as statistic significantly (raw P<0.05, FDR<0.25) RNAs with the same regulated tendency in both Wilcoxon rank-sum and Wilcoxon sign-rank test. DEGs in “*EGFR*-TKI resistance” (https://www.kegg.jp/entry/map01521) pathway of KEGG database were showed in pathway diagram.

Pathway enrichment analysis was performed in up-regulated and down-regulated DEGs, respectively, by “clusterProfiler” package referring GO (Gene Ontology) and KEGG (Kyoto Encyclopedia of Genes and Genomes database) database. Typical significant pathways (raw P<0.05, FDR<0.25) and related genes were showed in lollipop diagram and heatmap by “ggpubr” and “pheatmap” packages.

To exploring the potential gradual changes in transcriptome level among four sample groups (LUAD-NT, LUAD-BT, SCLC-AT, and SCLC-P), weighted average expression of up- and down-regulated DEGs during SCLC transformation were calculated for all samples, and displayed in scatter plot.

On ST level, the average value of each gene among spots from LUAD-NT, LUAD-BT, SCLC-AT and SCLC-P samples was calculated. The ranked log2FC between different types of samples were used to perform gene set enrichment analysis (GSEA) by “clusterProfiler” package, information of gene sets in “HALLMARK”, “Kyoto Encyclopedia of Genes and Genomes (KEGG)” and “Gene Ontology (GO)” was obtained from www.gsea-msigdb.org/gsea/msigdb/index.jsp. A total of 1,350 genesets associated tumor and its microenvironment were analyzed as a point of importance. The 1,350 genesets were summarized into six classes according to the information of these genesets provided on the molecular signatures database (MSigDB, www.gsea-msigdb.org/gsea/msigdb/index.jsp), including 421 “Immunity”, 127 “Cell cycle”, 315 “Metabolism & energy”, 286 “Genetic and epigenetic information”, 129 “extracellular matrix (ECM) & metastasis” and 72 “Cell death” genesets. The genesets with P< 0.05 & FDR <0.25 were considered as as statistically significant.

Supplementary material 5:Weighted Regulation network analysis during transformation

The average value of Spearman’s correlation coefficients were calculated to evaluated the potential regulation relationship during transformation between significantly changed regulator genes (including DNA methyltransferases, histone deacetylases, and transcription factors recruited in http://bioinfo.life.hust.edu.cn/HumanTFDB) and other DEGs (regulated genes) in both LUAD-BT and SCLC-AT samples. The correlation results were shown in heatmap according to the trend and statistical significance. Then we constructed a weighted regulation network using the absolute value of the Spearman’s correlation coefficients as the weight of the connection. The chord plot was used to display the weighted regulation network.

Supplementary material 6: ST sequencing and analysis

Slide sections (5μm) of FFPE block were incubated at 42℃ for 2 h and allowed to air dry at room temperature. Subsequently, the slides were dried for 3 h at 60℃. HE staining was performed. Staining durations were adapted based on tissue types. After staining, tissue imaging was conducted following the application of approximately 100 µL of 85% Glycerol (Thermofisher, Catalog number 15514011). The Visium slide was inserted into a cassette. To each well, 100 µL of 0.1 N HCl (Sigma-Aldrich, Product number H1758) was added, and incubation occurred at 42℃ for 15 min. Following the removal of HCl, decrosslinking buffer was introduced. Incubation at 95℃ for 1 h ensued. Pre-hybridization continued according to the guidelines of the Visium Spatial Gene Expression for FFPE reagent kit (10×Genomics, User Guide CG000407 Rev C, human transcriptome Product number 1000338). Pre-hybridization mix (100 µL) was added to each well, incubating at room temperature for 15 min. After the incubation period, the Pre-hybridization mix was replaced with Hybridization mix (100 µL), and the Visium slide underwent overnight incubation at 50℃. For the subsequent library preparation stages, encompassing probe ligation, probe release and extension, probe elution, and FFPE library assembly, the user guide “Visium Spatial Gene Expression for FFPE reagent kit” (10× Genomics, User Guide CG000407 Rev C, human transcriptome Product number 1000338) was strictly adhered to. The completed libraries were subjected to sequencing on the Novaseq6000 platform (Illumina).

Raw count of RNA were normalized, then processed by “Seurat” package. Tumor spots were identified based on HE staining slides by Loupe Browser 6 (https://support.10xgenomics.com/single-cell-gene-expression/software/visualization/latest/what-is-loupe-cell-browser) and “estimate” algorithm.

For each pair of transformed samples with ST data, batch effect correction was performed by “sva” package, then dimensionality reduction was completed by PCA and UMAP analysis. K-means method was used to group tumor spots into different clusters, the optimal number of clusters was determined by the “NbClust” package. In each transformed sample with ST data, cell-cell communication analysis among different clusters of tumor and other spots was performed by the “CellChat” package, and the “CellChatDB.human” was used as the reference. Pseudotime trajectory analysis was performed in tumor spots from each pair of transformed samples with ST data by “monocle” package, and the chronological order of samples was used to determined the direction of trajectory.

To evaluated TIME, the quanTIseq algorithm was used in the non-tumor spots to calculated the infiltration fraction of 10 types of immune cells by “immunedeconv” package. The spots with higher infiltration fraction of M1 macrophages than M2 were defined as “M1-polarized” otherwise as “M2-polarized”. Exhausted of CD8+ T cell or not was based on the PD1 and LAG3 RNA expression level of the spot.

Supplementary material 7: IHC for SCLC molecular subtypes

4μm tissue slides prepared from FFPE tissues of SCLC samples (including 21 SCLC-AT and 25 SCLC-P tissues) were subjected to IHC. IHC was done with rabbit monoclonal anti-MASH1/ASCL1 antibody (Abcam, ab211327, 1:100 dilution), mouse monoclonal anti-NEUROD1 antibody (Abcam, ab60704, 1:150 dilution), rabbit polyclonal anti-POU2F3 (BIOSS, BS-21046R, 1:300 dilution) and rabbit monoclonal anti-YAP1 antibody (Abcam, ab52771, 1:80 dilution) according to the manufacturer’s instructions. The expressions of four subtype-defining markers were evaluated using an H-score according to the following formula: H-score= % cells staining (0-100%) × intensity (range from 1 to 3), where an H-score of 0 corresponded to no staining and a score of 300 to maximum staining intensity in the entire tumor. The results of IHC were evaluated by two blinded independent pathologists (YL and LY). H-score of four markers were standardized using z-score normalization. Molecular subtype was determined according to the highest z-score of the sample. For markers with H-score=0 didn’t participate in the subtype determination of this sample. Pie charts were used to show the composition of the four subtypes in SCLC-AT and SCLC-P.

Supplementary material 8: Construction of a model to predict SCLC transformation and IHC validation

RNA expression was binary classified as high-expression and low-expression for all 730 RNA by each best cut-off value according to ROC curve using bulk RNA data of 12 LUAD-NT and 18 LUAD-BT samples. To achieve relative robust and concise model, only RNAs which could divide samples into two relatively even groups were used in model construction. Twenty-two RNAs which were statistically significant both in ROC and univariate Logistic Regression were enrolled in model construction. To minimize the risk of overfitting, the least absolute shrinkage and selection operator (LASSO)-penalized Logistic regression were applied, and 6 RNAs were selected.

Then IHC of these 6 candidate RNAs was performed on an expand cohort (N=60, consisting of 33 LUAD-NT and 27 LUAD-BT samples) according to the manufacturer’s instructions (rabbit polyclonal anti-COL6A6 antibody [Abcam, ab150926, 1:200 dilution], mouse monoclonal anti-CASP12 antibody [SANT CRUZ, sc-21747, 1:50 dilution], rabbit polyclonal anti-HHIP antibody [Abcam, ab230271, 1:400 dilution], mouse monoclonal anti-ZBTB16 antibody [SANT CRUZ, sc-28319, 1:1000 dilution], rabbit monoclonal anti-BIRC3 antibody [Abcam, ab32059, 1:600 dilution], and rabbit monoclonal anti-GATA2 antibody [Abcam, ab182747, 1:500 dilution]).The IHC staining slides were scanned by NanoZoomer Digital slide scanner (S210, Hamamatsu), and analyzed by QuPath-0.4.3. For each slide, H-scores of the tumor region and all regions were obtained, generating 12 H-scores. We applied LASSO-Logistic to select 4 H-scores and trained models using three algorithms (Logistic Regression Model, LASSO-Logistic Regression Model, and Randomforest Model) to discriminate the LUAD-BT to LUAD-NT in the training cohort (N=42). The performance of these three models were further evaluated in the test cohort (N=18) using ROC. And the Randomforest Model showed best performance and was selected.

Supplementary material 9: Construction of models to predict TTT and OStrans

RNA expression was binary classified as high-expression and low-expression for all 730 RNA by each best cut-off value according to KM survival analysis using bulk RNA data of LUAD-BT (for TTT Model) and SCLC-AT (for OStrans Model). To achieve relative robust and concise model, only RNAs which could divide samples into two relatively even groups were used in model construction. RNAs which were statistically significant both in KM analysis and univariate Cox regression were enrolled. To minimize the risk of overfitting, the LASSO-penalized Cox regression were applied. Ten-fold cross validation was performed to determine the best, and the λ with minimum mean cross-validated error was used to decide the final model. Univariate Cox analysis was performed to select potential influential factors, and factors with P<0.25 were used to perfomed multivariate Cox analysis.

1)Construction of TTT Model

For patients with potential transformation trend, identifying of the time when transformation occurred was also of great importance. We noticed that among 18 patients with LUAD-BT samples, the TTT varied from 5 to 67 months. Thus, we constructed a model to predict TTT with 27 RNAs which were statistically significant both in KM analysis and in univariate Cox regression. Using LASSO-penalized Cox regression analysis, a 8-genes expression model was constructed based on the optimal value of λ. Survival analyses according to the optimal cut-off value of each genes indicated that high expression of 6 genes (including *JUN*, *WNT11*, *WIF1*, *IL11RA*, *PLAU*, *CACNA2D3*) and low expression of 2 genes (including *C19orf40* and *DAXX*) correlated with a significant longer TTT. The heatmap of these 8 genes showed distinct expression pattern between patients with slow transformation (longer TTT) and those with rapid transformation (shorter TTT) (**Supplementary Fig.12a**). The 18 LUAD-BT patients were categorized into two groups: slow transformation group with lower TTT Score (N=9) and rapid transformation group with higher TTT Score (N=9) according to the median of TTT Score (-0.855, details in **Supplementary Table 6**). Using KM analysis, the rapid transformation group did show significantly shorter median TTT (≤-0.855) than the slow transformation group other (P<0.001, median TTT: 16.0 versus 46.1 months, **Supplementary Fig.12b**). According to multivariate Cox proportional hazard analysis, TTT Score was an independent marker for time to transformation (P=0.008, HR=20.904, 95%CI: 2.247-194.513, **Supplementary Fig.12c** and **Supplementary Fig.12d** showed the results of univariate and multivariate Cox analysis.).

2)Construction and validation of OStrans Model

To indicate the prognosis for transformed SCLC, we constructed a model to predict OStrans according to the bulk RNA data of patients with SCLC-AT samples. Thirty-two RNAs with statistically significant both in KM analysis and univariate Cox regression were enrolled in the LASSO-penalized Cox regression analysis, and a 9-genes expression model was constructed based on the optimal value of λ. Survival analyses according to the optimal cut-off value of each genes indicated that high expression of 3 genes (including *DDIT4*, *FGF2*, *CDKN2A*) and low expression of 6 genes (including *BAIAP3*, *TNFRSF10B*, *FGF3*, *SHC4*, *GADD45B*, *SGK2*) correlated with a significant longer OS after transformation . The same trends were observed in the heatmap of these 9 genes in SCLC-AT patients (**Supplementary Fig.13a**). Twenty-one patients with SCLC-AT samples were stratified into a worse poor prognosis group with higher OStrans Score (N=10) and a better good prognosis group with lower OStrans Score (N=11) according to the median of OStrans Score (-0.062, details in **Supplementary Table 7**). **Supplementary Fig.12b** showed significantly difference of OStrans between the two groups (P<0.001, median OStrans:13.3 versus 23.3 months). **Supplementary Fig.13c** showed the results of univariate Cox analysis, and age, molecular subtype, OStrans Score were included in multivariate Cox analysis (**Supplementary Fig.13d** and **Supplementary Fig.13e**, molecular subtype was stratified into NE-high and NE-low in **Supplementary Fig.13d**, and was stratified into Y and non-Y in **Supplementary Fig.13e**).

As the transcriptomic feature of SCLC-P was in proximity to that of SCLC-AT, we also evaluated OStrans Score in 20 SCLC-P patients with bulk RNA data in order to validate the power of the OStrans Model (details in **Supplementary Table 7**). The 2-years, 3-years, and 4-years OS rates of SCLC-P patients with lower OStrans Score were better than those with higher Score (**Supplementary Fig.13f**). It verified that OStrans Score could suggest the prognosis of patients after SCLC transformation in the flank direction.

Supplementary material 10: mIF

Four mIF panels were performed in 10 samples (2 LUAD-NT, 2 SCLC-P, 3 LUAD-BT with paired 3 SCLC-AT). Panel 1: 4',6-diamidino-2-phenylindole (DAPI), panCK (Abcam, ab234297), CD68 (ZSGB-BIO, ZM-0060), IRF5 (Abcam, ab181553), CD86 (ABclonal, A21198), CD206 (Abcam, ab64693) and CD163 (Abcam, ab182422); Panel 2: DAPI, panCK (Abcam, ab234297), CD3 (Abcam, ab237707), CD8 (Thermo Fisher Scientific, MA1-80231), PD1 (Abcam, ab137132), LAG3 (Abcam, ab209236) and FOXP3 (Cell Signaling Technology, #98377); Panel 3: DAPI, panCK (Abcam, ab234297), Vimentin (Cell Signaling Technology, #5741), α-SMA (Abcam, ab124964), HGF (Cell Signaling Technology, #52445), FGF7 (Santa Cruz Biotechnology, sc-365440) and pSMAD2 (Cell Signaling Technology, #18338); Panel 4: DAPI, panCK (Abcam, ab234297), CD20 (Cell Signaling Technology, #48750), CD24 (Santa Cruz Biotechnology, sc-19585), CD123 (Abcam, ab280355), CD11c (Cell Signaling Technology, #45581S) and CD14 (Cell Signaling Technology, #75181).

Slide sections of FFPE block were deparaffinized in xylene and rehydrated in ethanol. After microwave antigen retrieval in heated citric acid buffer (pH 6.0) for 10 min, endogenous peroxidase activity was blocked by 3% H2O2 for 10 min, and nonspecific binding sites were blocked by goat serum for 10 min. Primary antibodies were incubated for 1 h in a humidified chamber at room temperature, followed by incubation with the corresponding secondary horseradish peroxidase-conjugated polymer. Visualization of each target was accomplished using fluorescein TSA Plus (1:100). Then, the slide was again placed in a heated citric acid buffer (pH 6.0) using microwave antigen retrieval to remove redundant antibodies before the next step. Finally, nuclei were subsequently visualized with DAPI, and the sections were coverslipped using antifade mounting medium.

Slides were scanned by Vectra Polaris (Akoya), and were analyzed by Halo Link (Indica Labs). Classification of positive cell on each channel was based on staining intensity and completeness. The cell type was identified according to the combination of different channels. Only cells with DAPI positive were included in the following counting and analysis. In panel 1, cells with “panCK-CD68+” were identified as macrophages, the “M1-polarized” macrophage was defined as macrophage with “CD86+IRF5+CD163-CD206-”, “CD86+IRF5-CD163-CD206-”, “CD86-IRF5+CD163-CD206-”, “CD86+IRF5+CD163-CD206+”, or “CD86+IRF5+CD163+CD206-”, the “M2-polarized” macrophage was defined as macrophage with “CD86-IRF5-CD163+CD206+”, “CD86-IRF5-CD163-CD206+”, “CD86-IRF5-CD163+CD206-”, “CD86-IRF5+CD163+CD206+”, or “CD86+IRF5-CD163+CD206+”, the other macrophages were identified as “Non-polarized” macrophages. In panel 2, cells with “panCK-CD3+” were identified as T lymphocytes, the “Tc-effector” was defined as T lymphocyte with “CD8+PD1-LAG3-FOXP3-”, the “Tc-exhausted” was defined as T lymphocyte with “CD8+PD1+LAG3-FOXP3-”, “CD8+PD1-LAG3+FOXP3-”, or “CD8+PD1+LAG3+FOXP3-”, the “Treg-effector” was defined as T lymphocyte with “CD8-PD1-LAG3-FOXP3+”, the “Treg-exhausted” was defined as T lymphocyte with “CD8-PD1+LAG3-FOXP3+”, “CD8-PD1-LAG3+FOXP3+”, or “CD8-PD1+LAG3+FOXP3+”, the “T8reg” was defined as T lymphocyte with “CD8+FOXP3+”, the other T lymphocytes were identified as “Other-T”; In panel 3, cells with “panCK-Vimentin+” were identified as fibroblasts, the “normal fibroblast (NF)” was defined as fibroblast with “α-SMA-” and fibroblasts with “α-SMA+” were “CAF”, the “type Ⅰ CAF (CAFⅠ)” was defined as CAF with “HGF+pSMAD2-”, the “type Ⅱ CAF (CAFⅡ)” was defined as CAF with “HGF-FGF7+pSMAD2-”, the “type Ⅲ CAF (CAFⅢ)” was defined as CAF with “HGF-FGF7-pSMAD2+”, the other CAFs were identified as “Other-CAF”; In panel 4, the “B cell effector (Be)” was defined as cell with “panCK-CD20+CD11c-CD14-CD123-CD24-”, the “regulatory B cell (Breg)” was defined as cell with “panCK-CD20+CD11c-CD14-CD123-CD24+”, the “cDC” was defined as cell with “panCK-CD20-CD11c+CD14-CD123-CD24-”, the “mDC” was defined as cell with “panCK-CD20-CD11c+CD14+CD123-CD24-”, the “plasmacytoid dendritic cell (pDC)” was defined as cell with “panCK-CD20-CD11c-CD14-CD123+CD24-”. Cell infiltration fractions of different macrophages, T lymphocytes, fibroblasts, DCs and B cells were calculated based on the results from the four mIF panels.


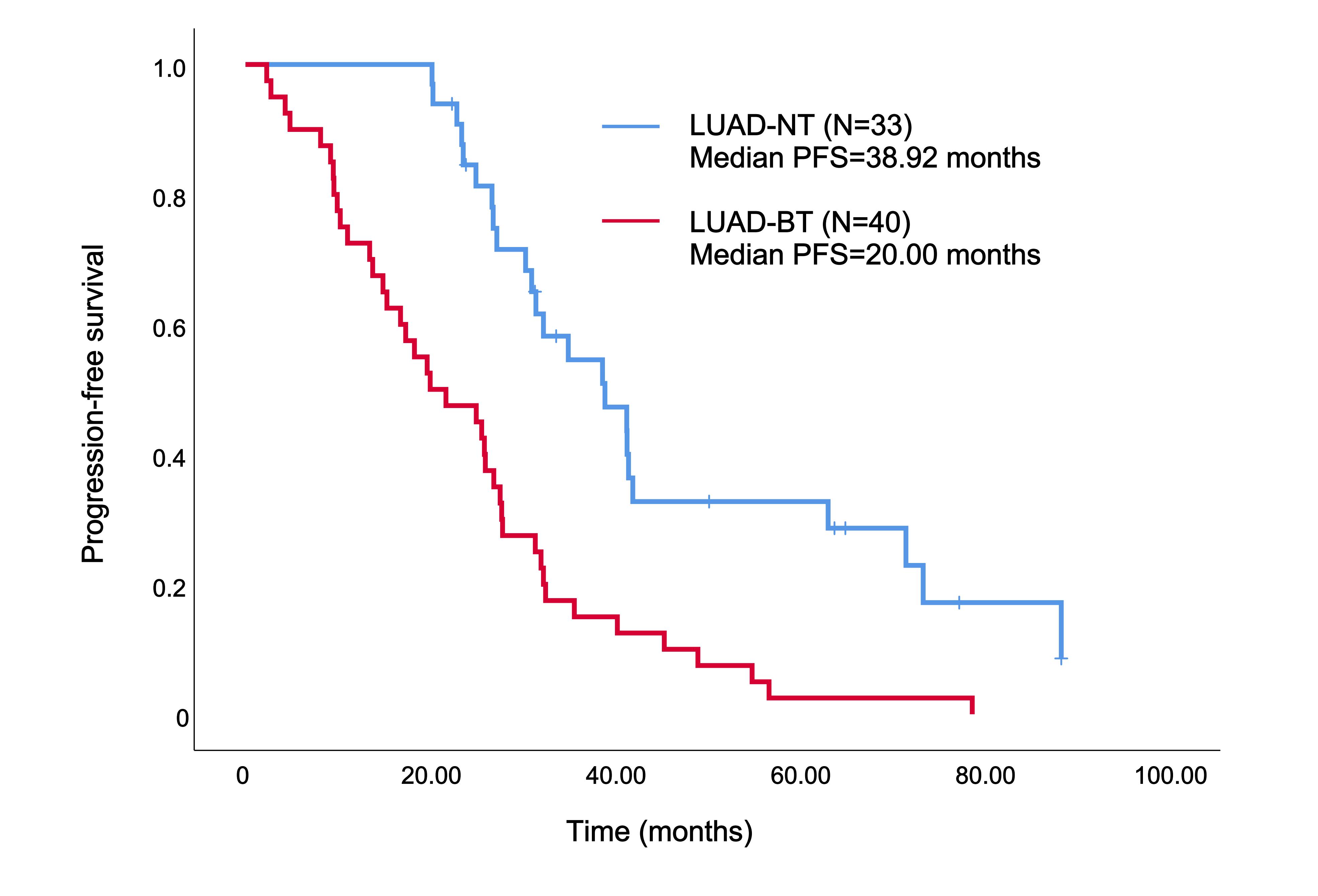


Figure. S1.

**Survival analysis of LUAD patients enrolled in our study**. Kaplan-Meier analysis of PFS between LUAD patients with and without SCLC transformation (in LUAD patients with SCLC transformation, PFS refered to *EGFR* TKI PFS before SCLC transformation).


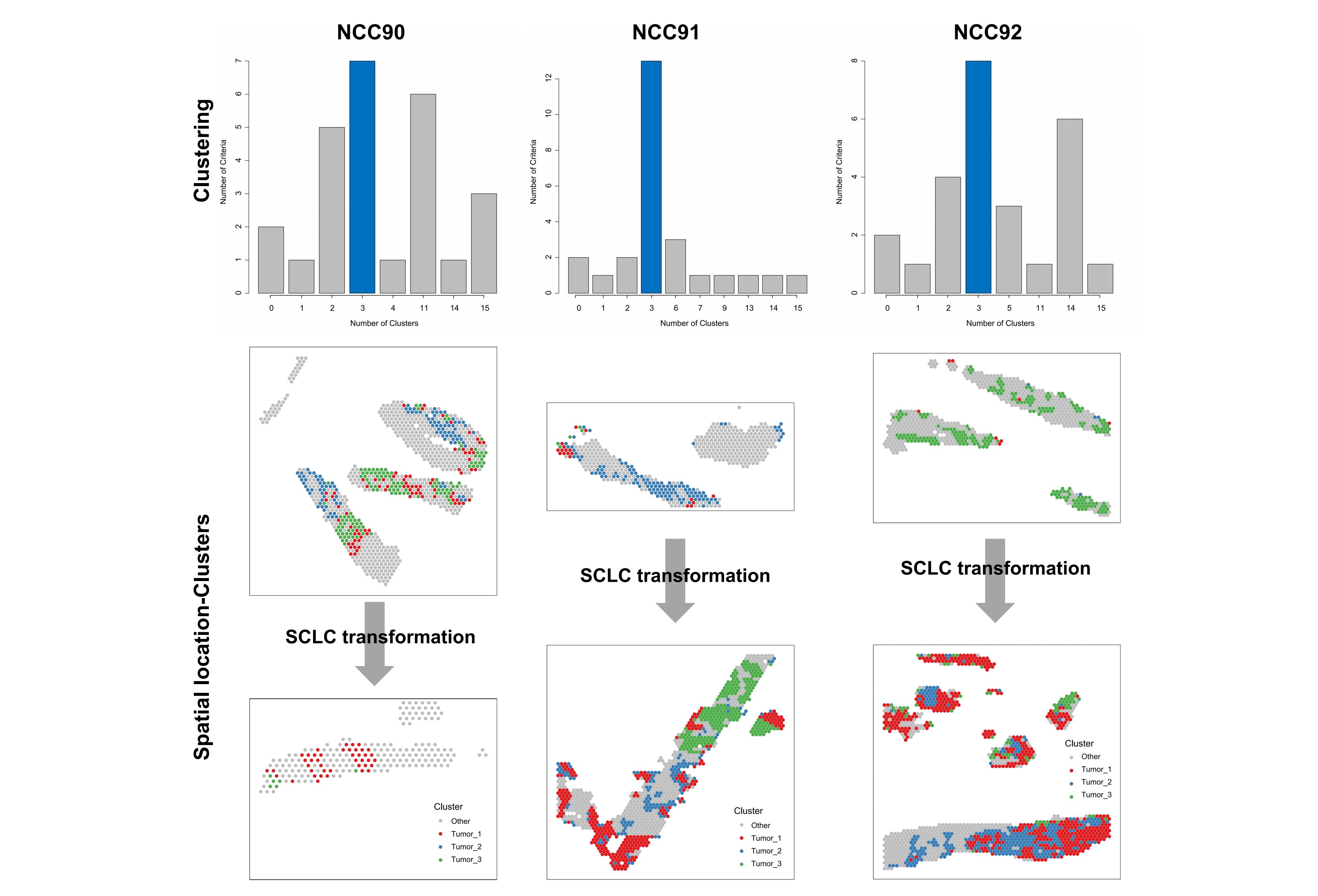


Figure. S2.

**Spatial distribution of tumor spot clusters during transformation in the same patients using ST data**. First row. Determination of optimal cluster number for each patient (patients No.: NCC90, NCC91, NCC92, respectively). second row). Spatial location of tumor spot clusters in LUAD-BT and SCLC-AT sampels for each patients (patients No.: NCC90, NCC91, NCC92, respectively)


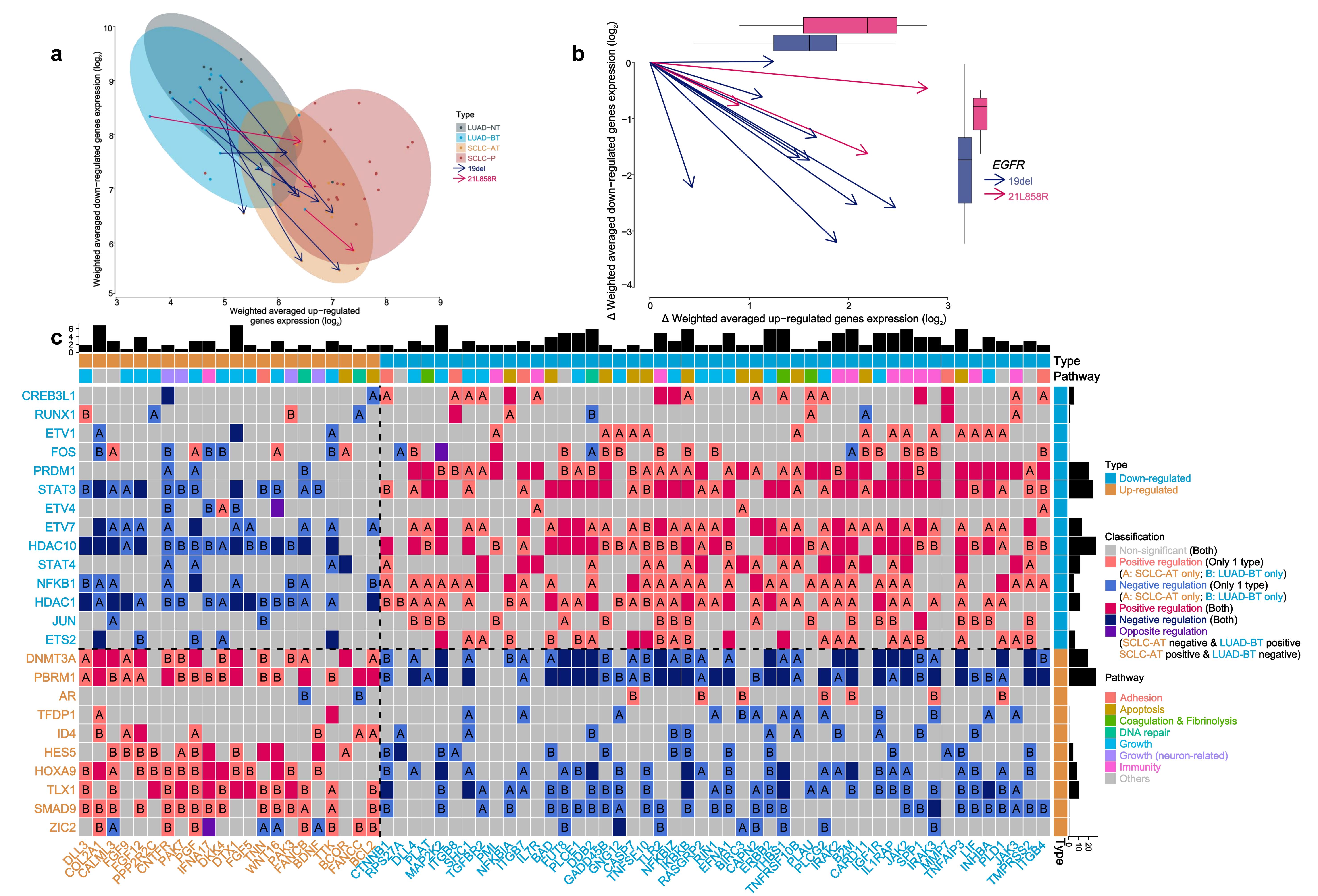


Figure. S3.

1. scatter plot displayed the weighted expression of up-regulated and down-regulated DEGs. b) the different transformation mechanisms between 19del and L858R according to Δweighted average up-/down-regulated gene expression. c) the relationships between regulation factors (including transcription factors, DNA methyltransferases, and histone deacetylases) and regulated genes (other DEGs) during transformation.


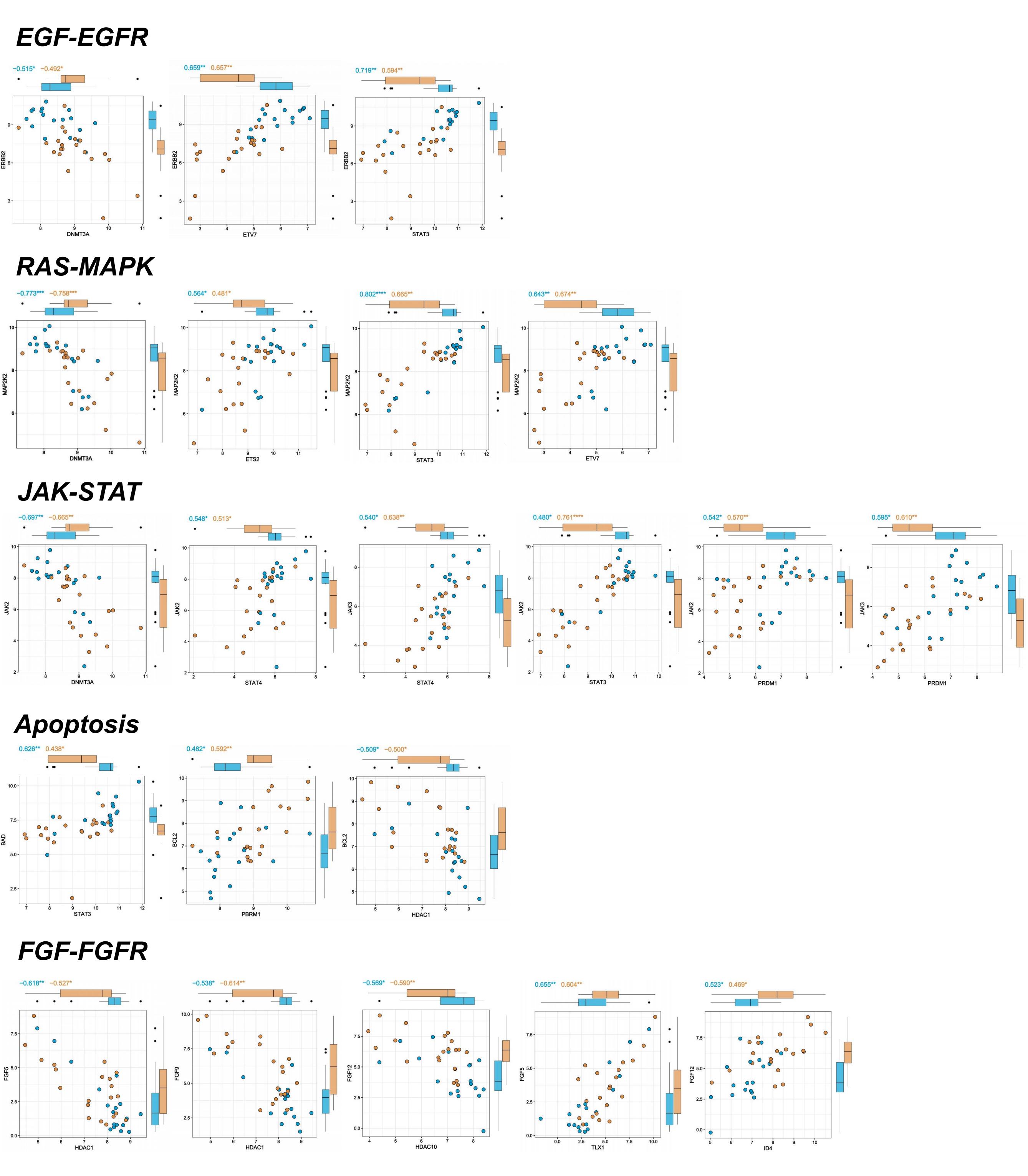


Figure. S4.

**Regulation relationship of genes in *EGFR*-TKI resistance pathway**. The number in blue and yellow at the left top of each plot represented the Spearman’s correlation coefficient in LUAD-BT and SCLC-AT, respectively. The number of stars in plots meant statistic significant: “*” for “0.05>P>0.01”, “**” for “0.01>P>0.001”, “***” for “0.001>P>0.0001”, and “****” for “P<0.0001”


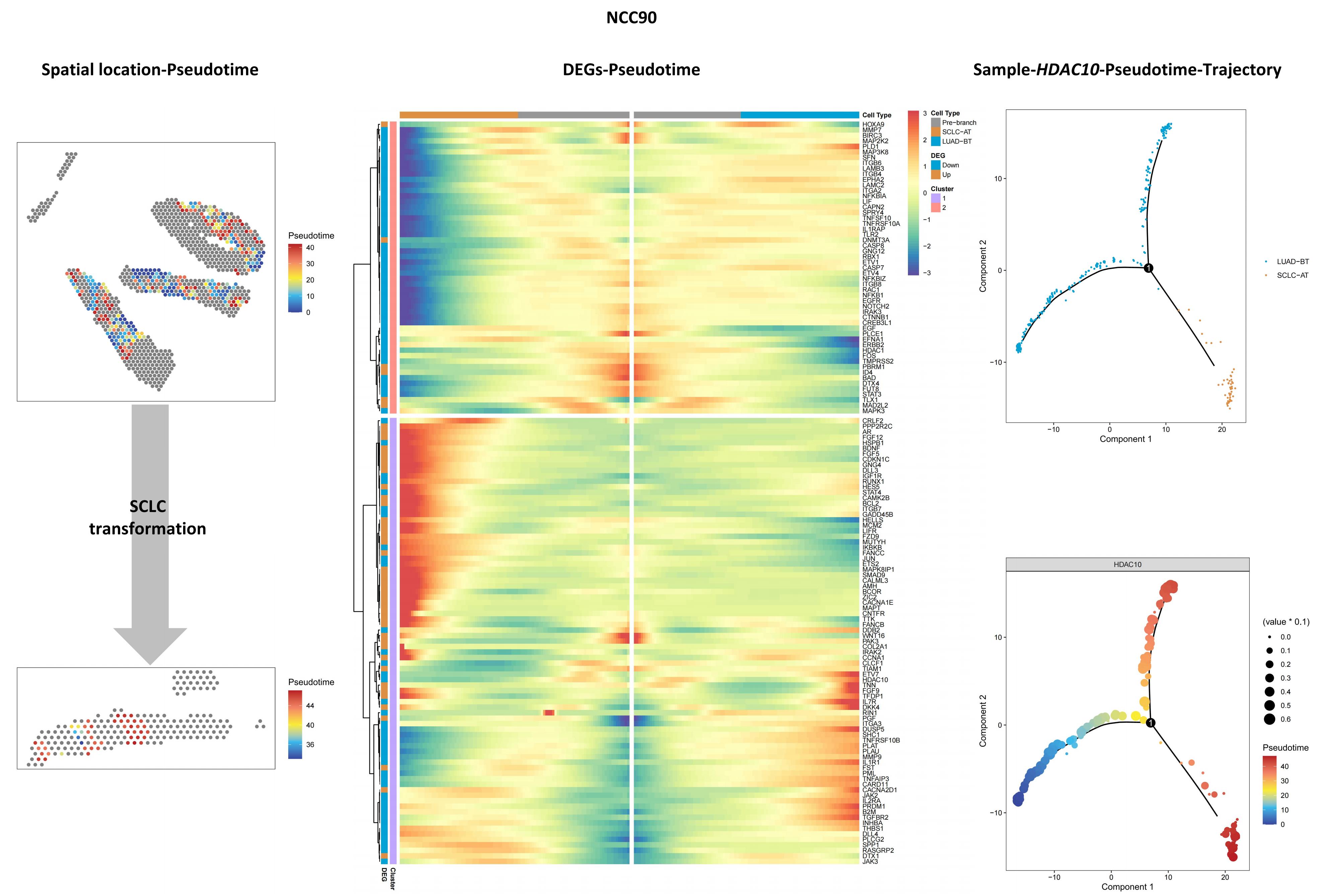


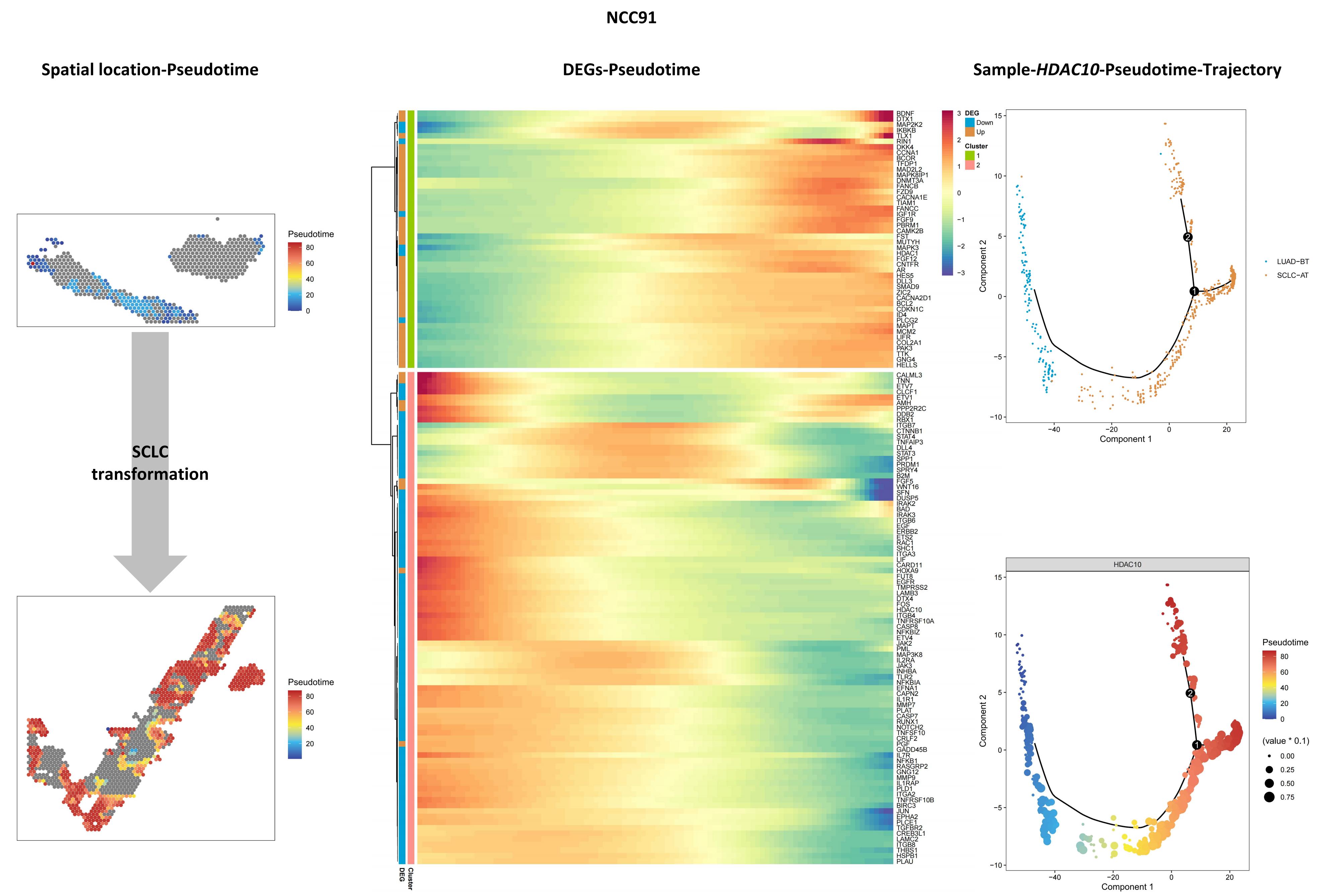


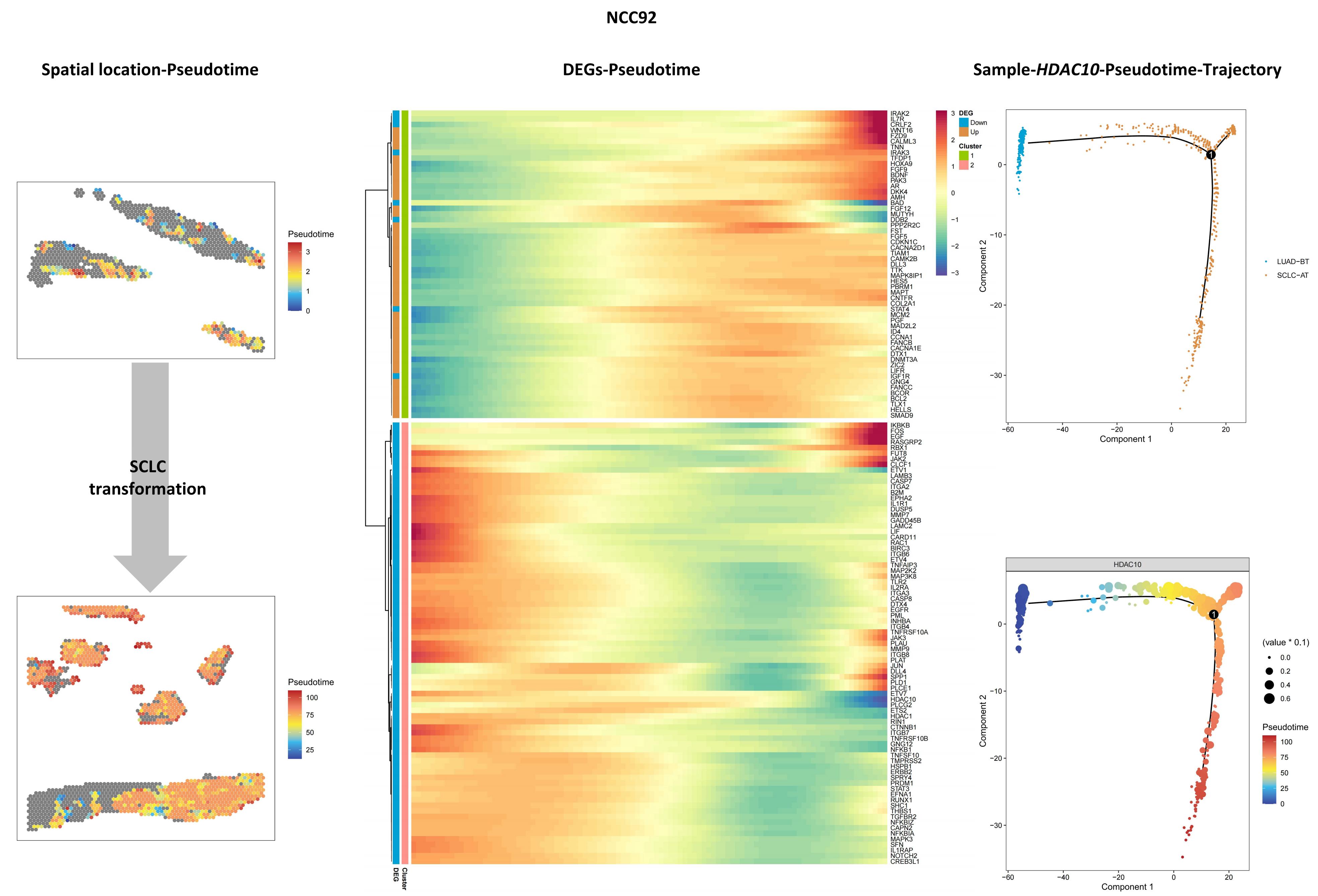


Figure. S5.

**Pseudotime analysis with ST data during transformation in three patients** (patients No.: NCC90, NCC91, NCC92, respectively).


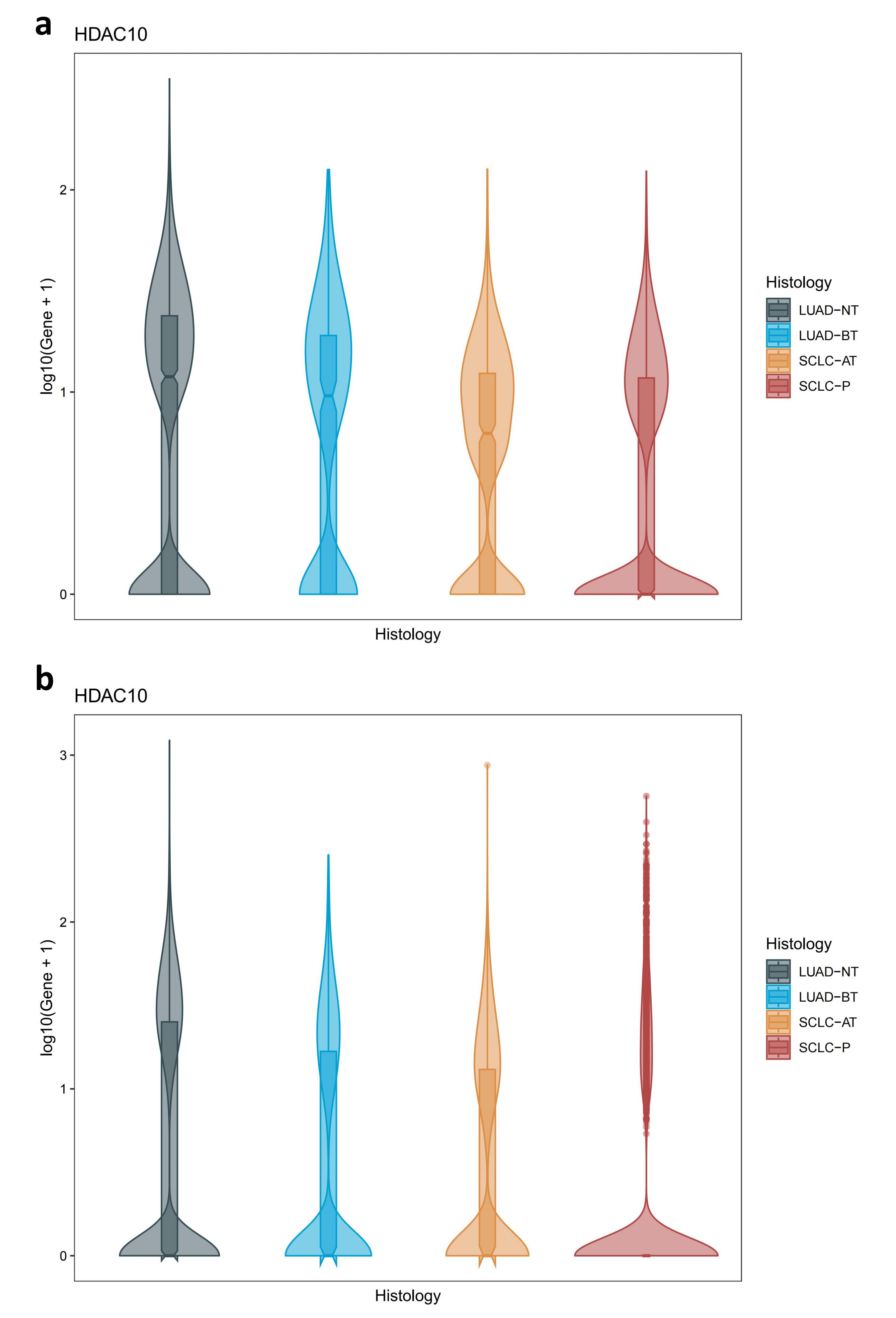


Figure. S6.

**The expression of *HDAC10* in four groups (LUAD-NT, LUAD-BT, SCLC-AT, and SCLC-P) based on ST data**. a) violin plot of *HDAC10* expression in tumor spots. b) violin plot of *HDAC10* expression in non-tumor spots


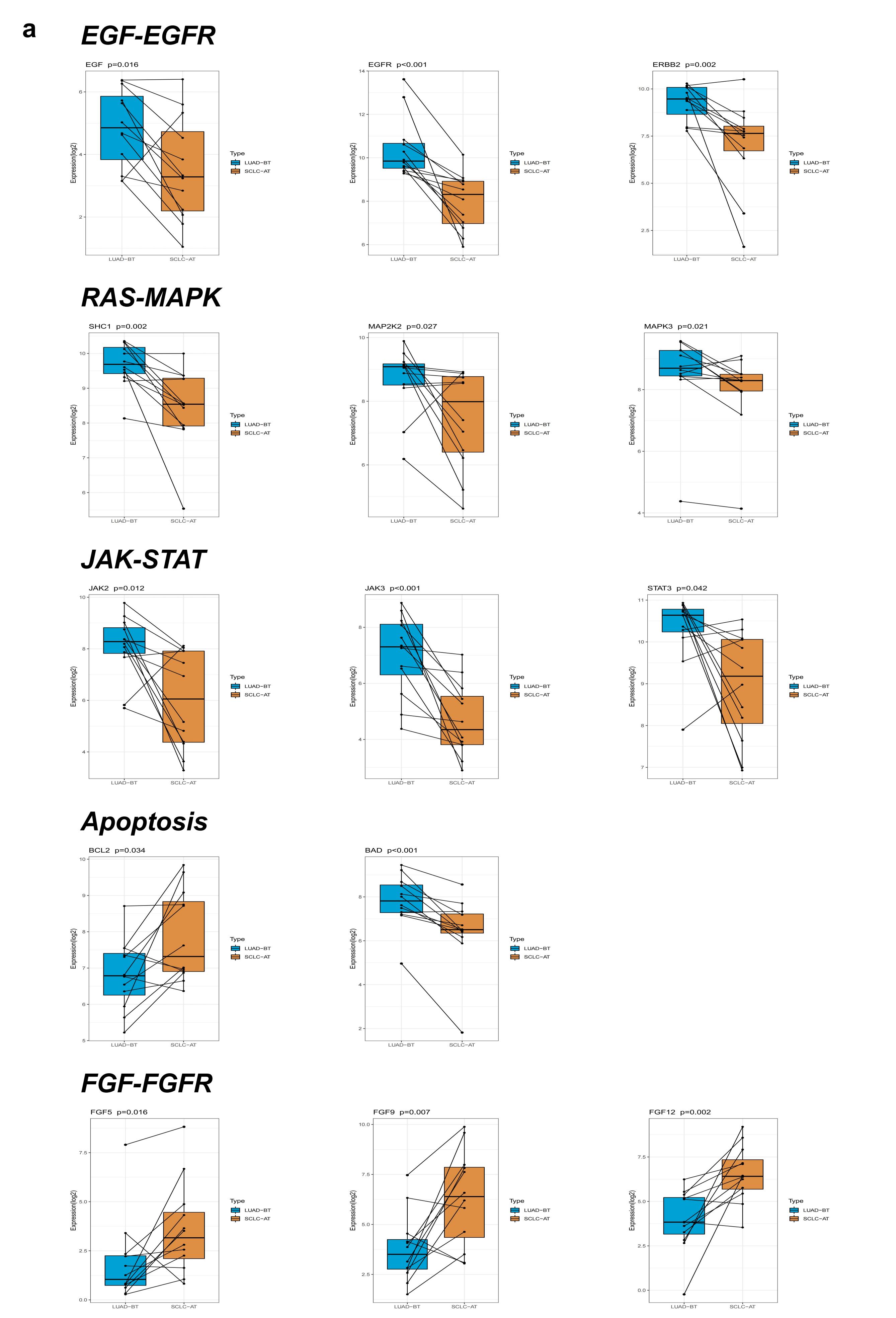


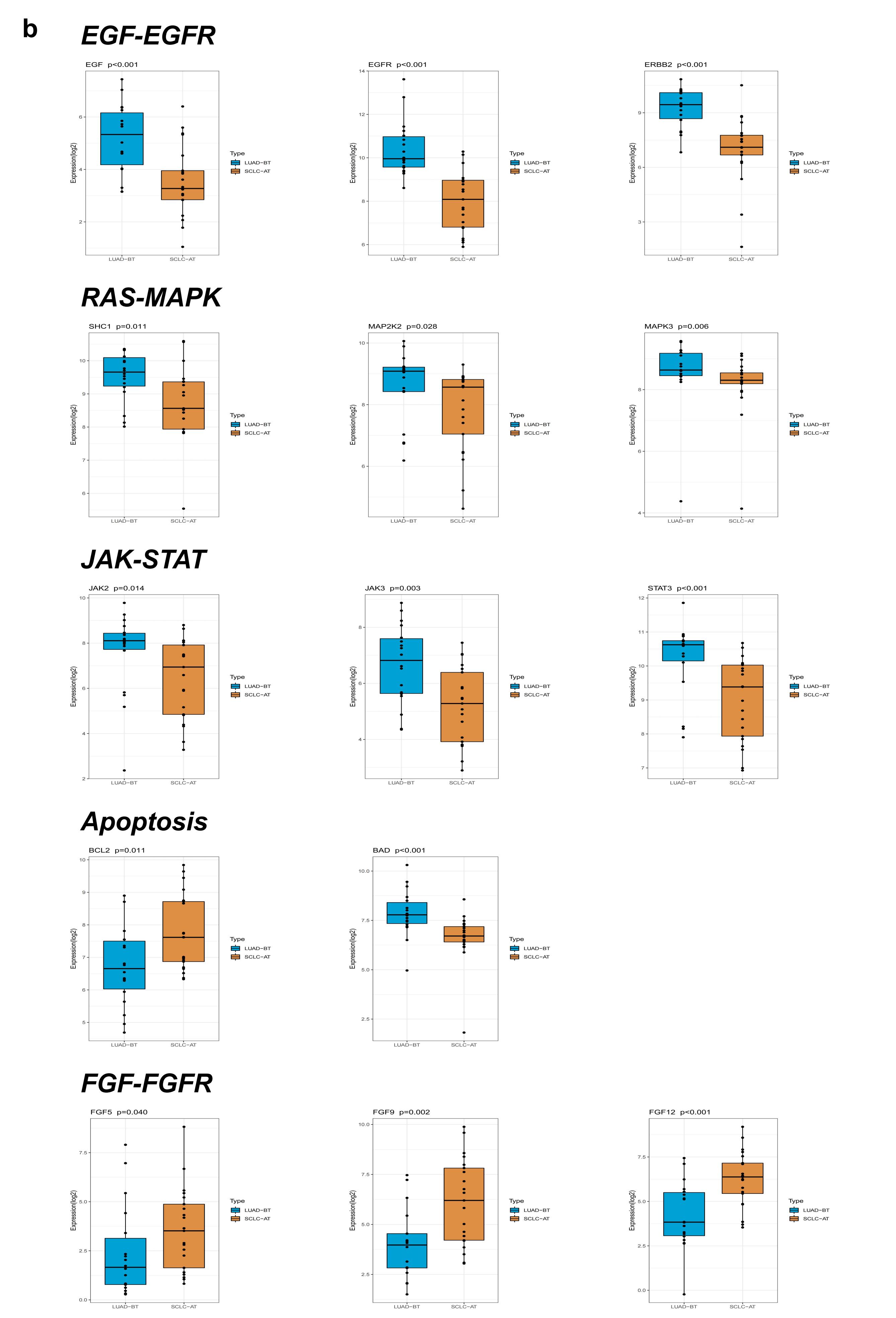


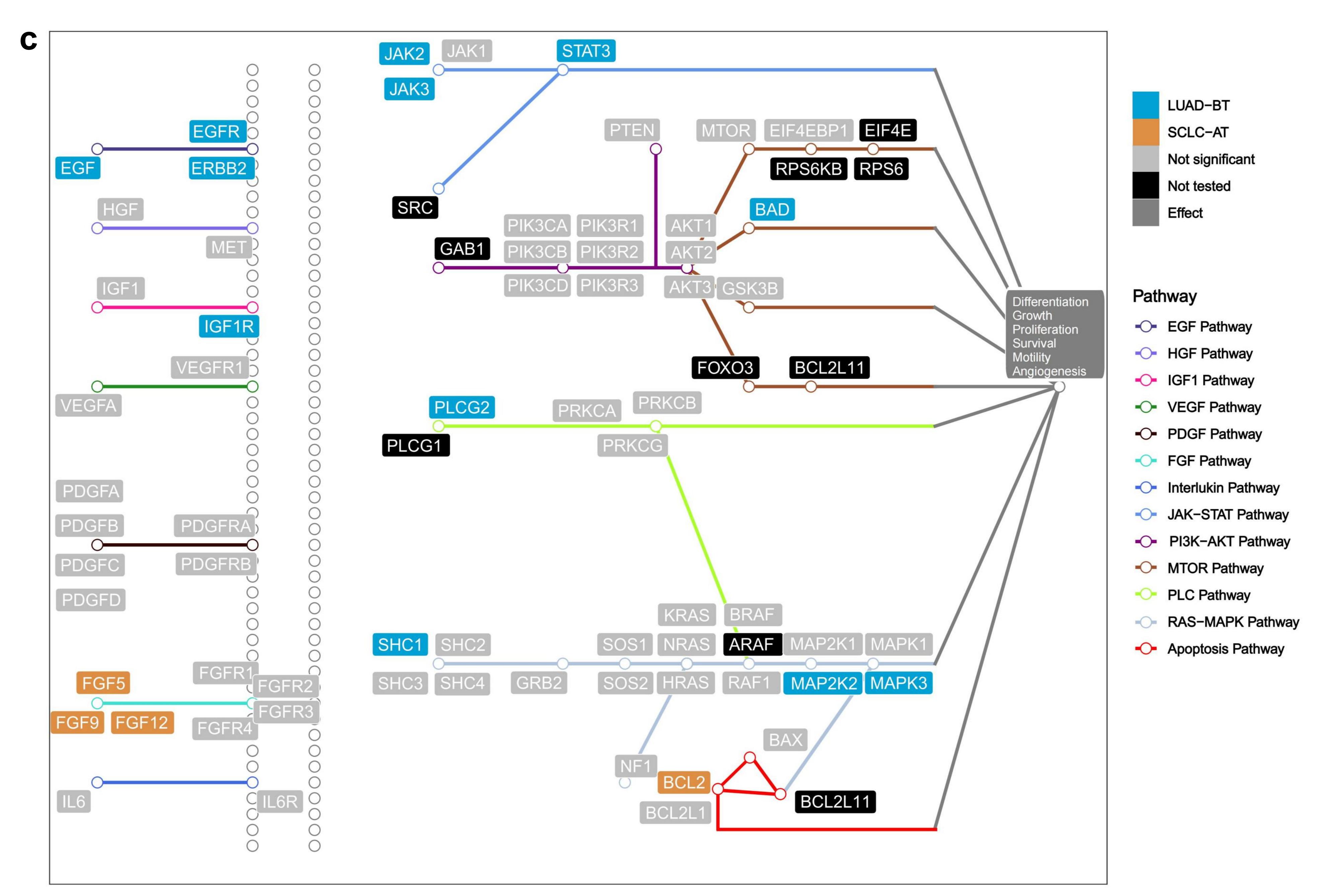
Figure. S7.

**The changes of pathways related to *EGFR*-TKI resistance.** a) different expression of key genes between paired LUAD-BT and SCLC-AT samples in *EGF*-*EGFR*, *RAS*-*MAPK*, *JAK*-*STAT*, apoptosis and *FGF*-*FGFR* pathway. b) different expression of key genes between 18 LUAD-BT and 21 SCLC-AT (unpaired samples) in *EGF*-*EGFR*, *RAS*-*MAPK*, *JAK*-*STAT*, apoptosis and *FGF*-*FGFR* pathway. c) change trends of pathways related to *EGFR*-TKI resistance


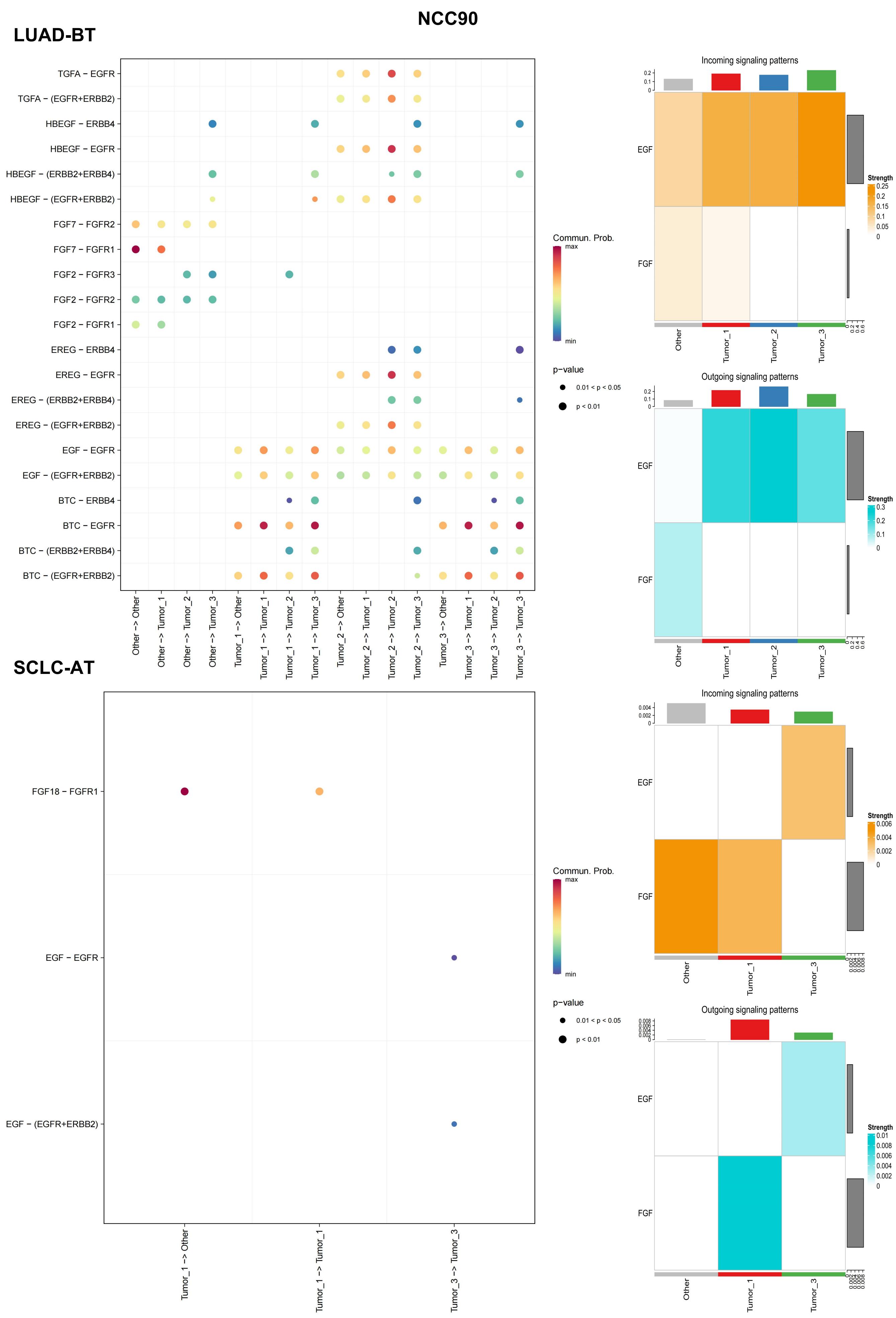


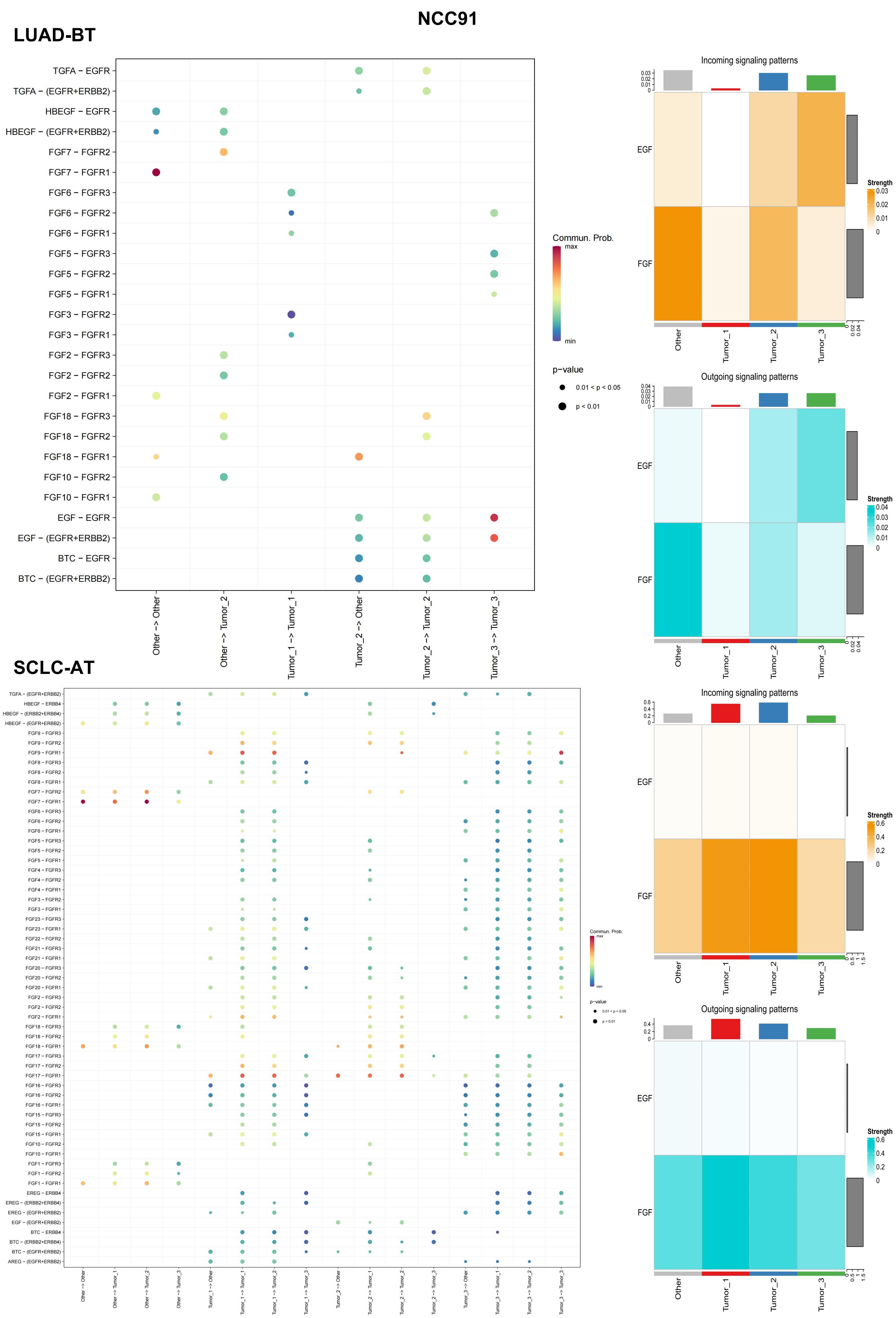


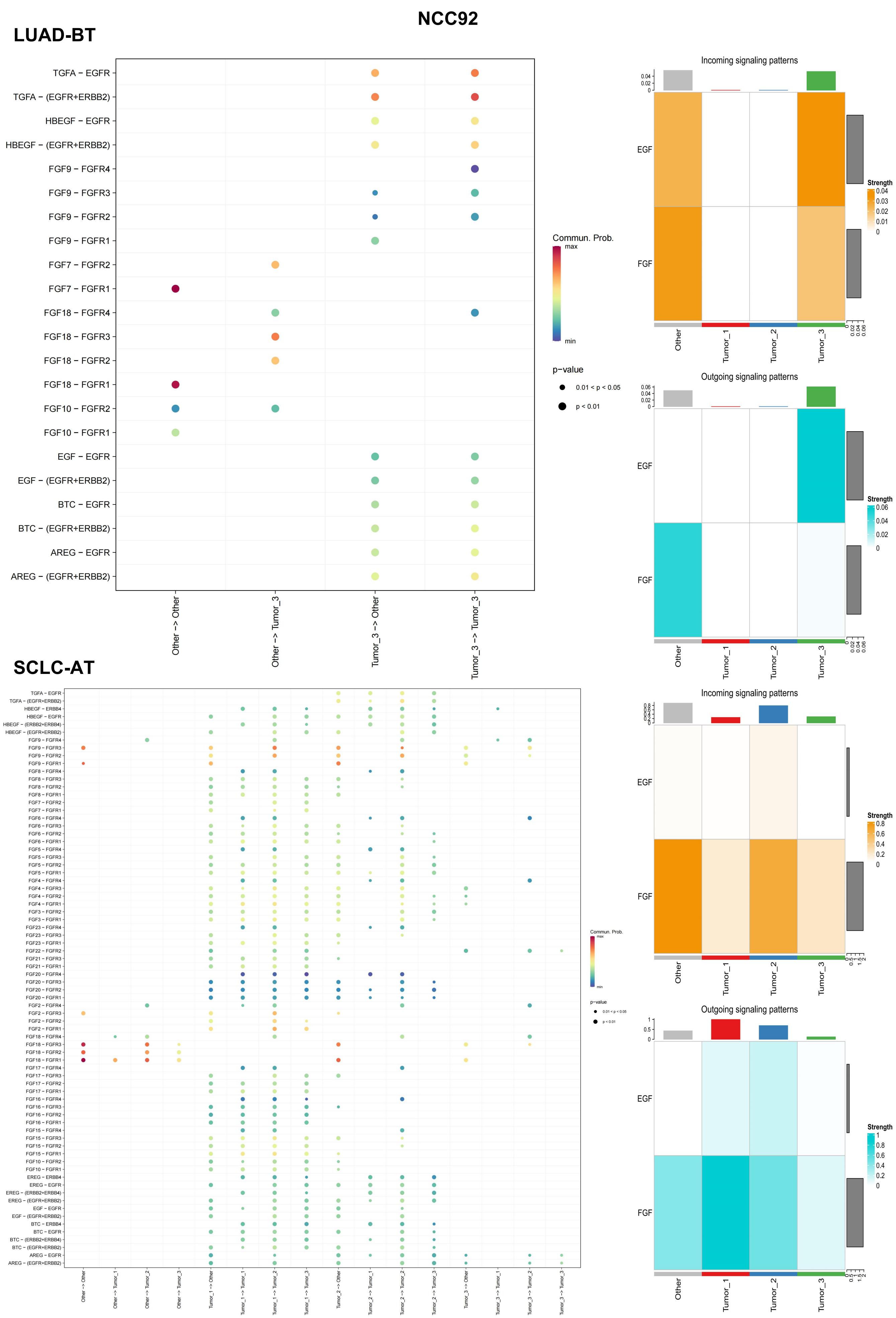


Figure. S8.

**Cell-cell communication based on ST data of paired LUAD-BT and SCLC-AT samples of three patients** (patients No.: NCC90, 91, 92, respectively).


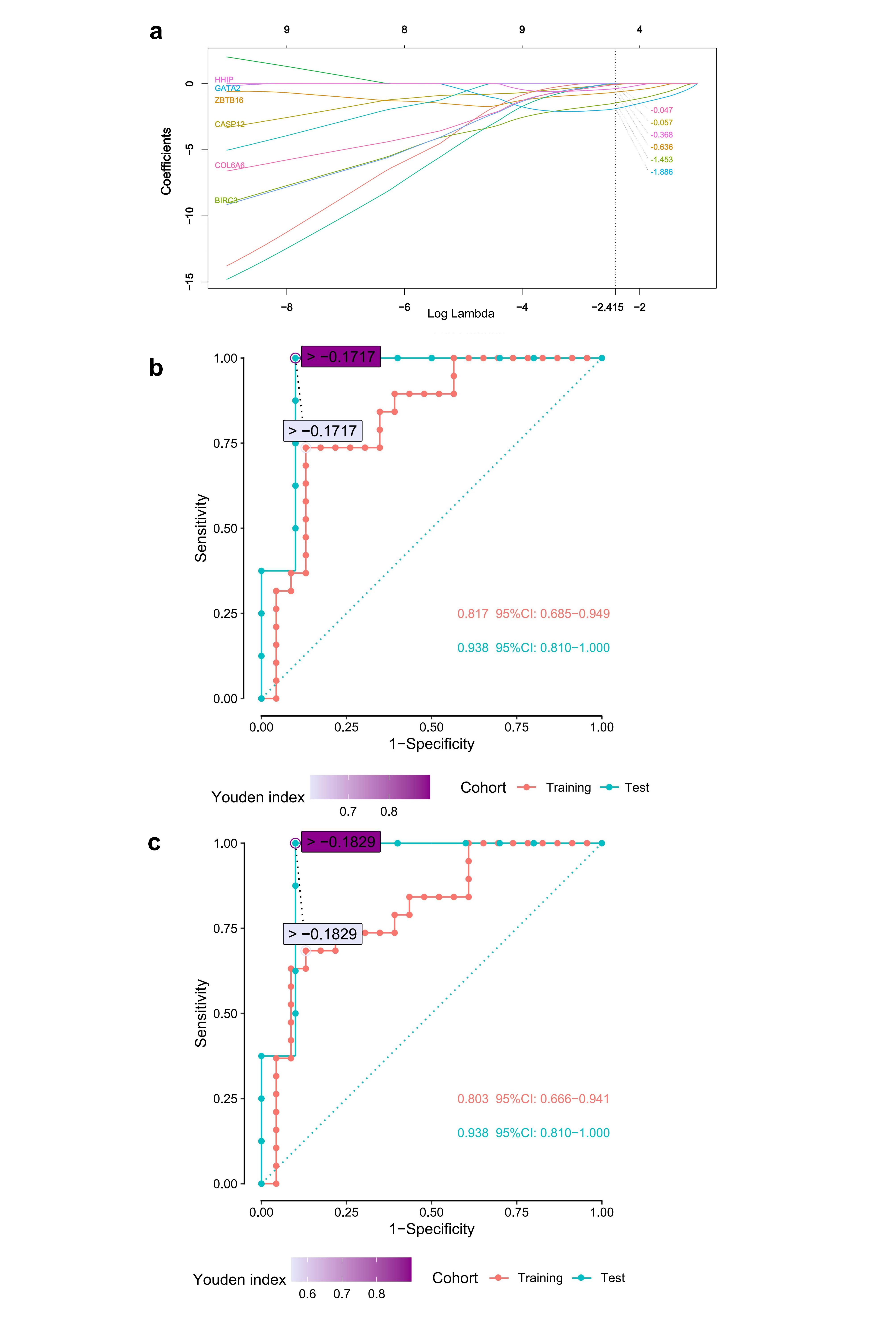


Figure. S9.

**Construction of SCLCtrans Mode**l. a) LASSO-penalized Logistic Regression analysis identified 6 candidate RNAs based on the optimal value of λ. b) ROC curve measuring the performation of SCLCtrans Model to distinguish LUAD-BT to LUAD-NT in both training and test cohort using Logistic Regression Model. c) ROC curve measuring the performation of SCLCtrans Model to distinguish LUAD-BT to LUAD-NT in both training and test cohort using LASSO-Logistic Regression Model.


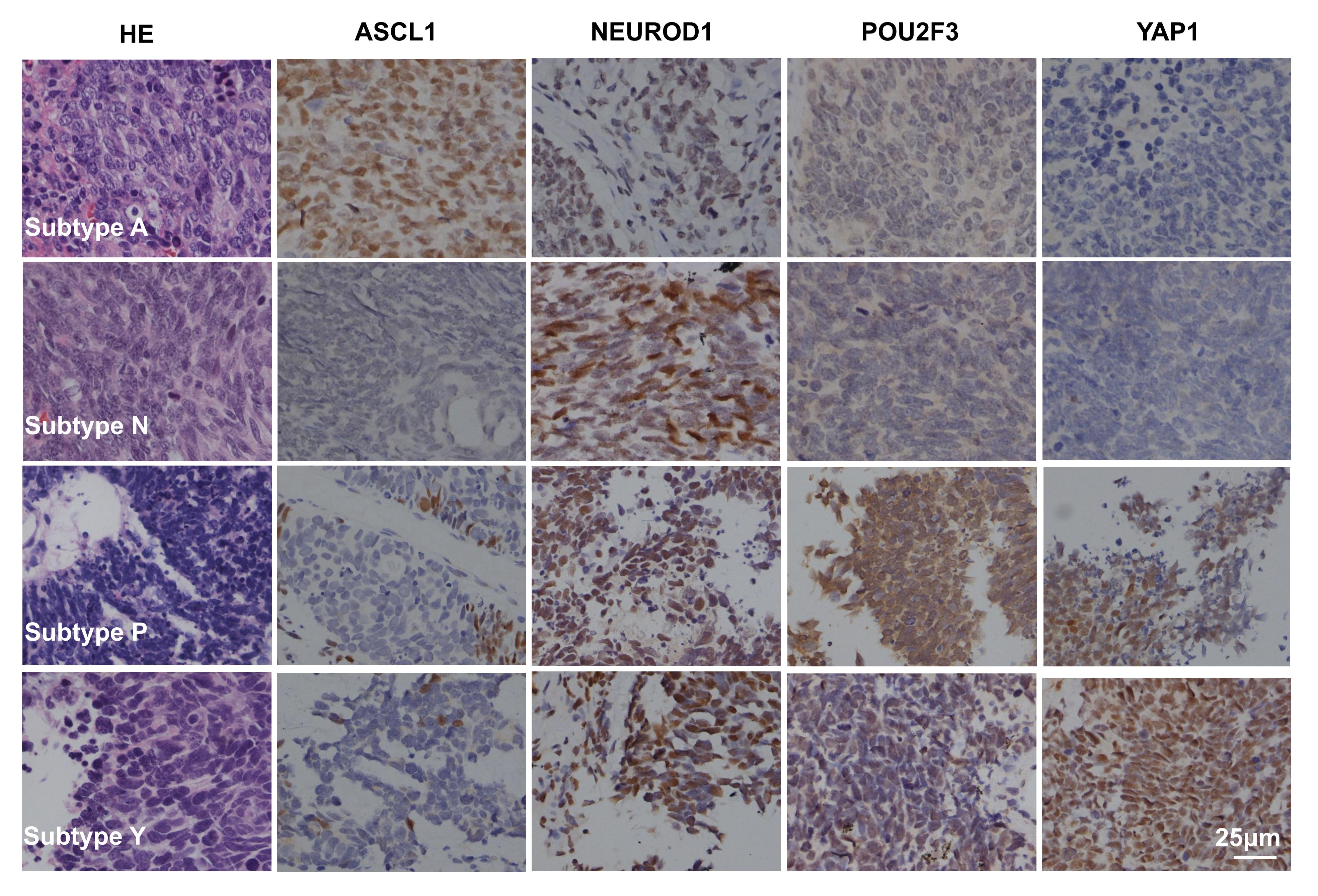


Figure. S10.

**Representative IHC staining of four molecular subtypes in SCLC-AT and SCLC-P** (400×).


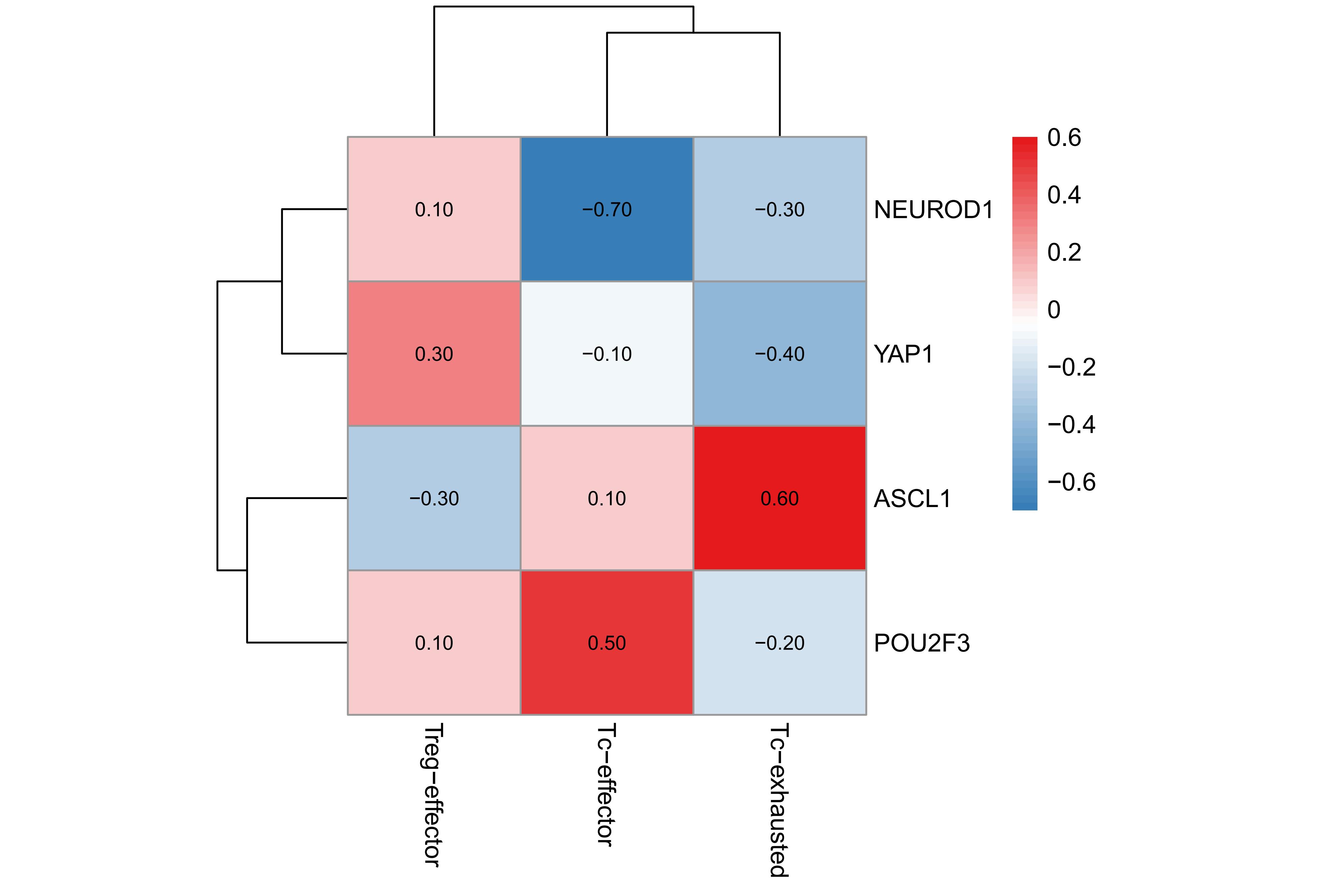


Figure. S11.

**The heatmap according to the Spearman’s correlation coefficients between infiltration fractions of different immune-related cell types and expressions of transcription factors.**


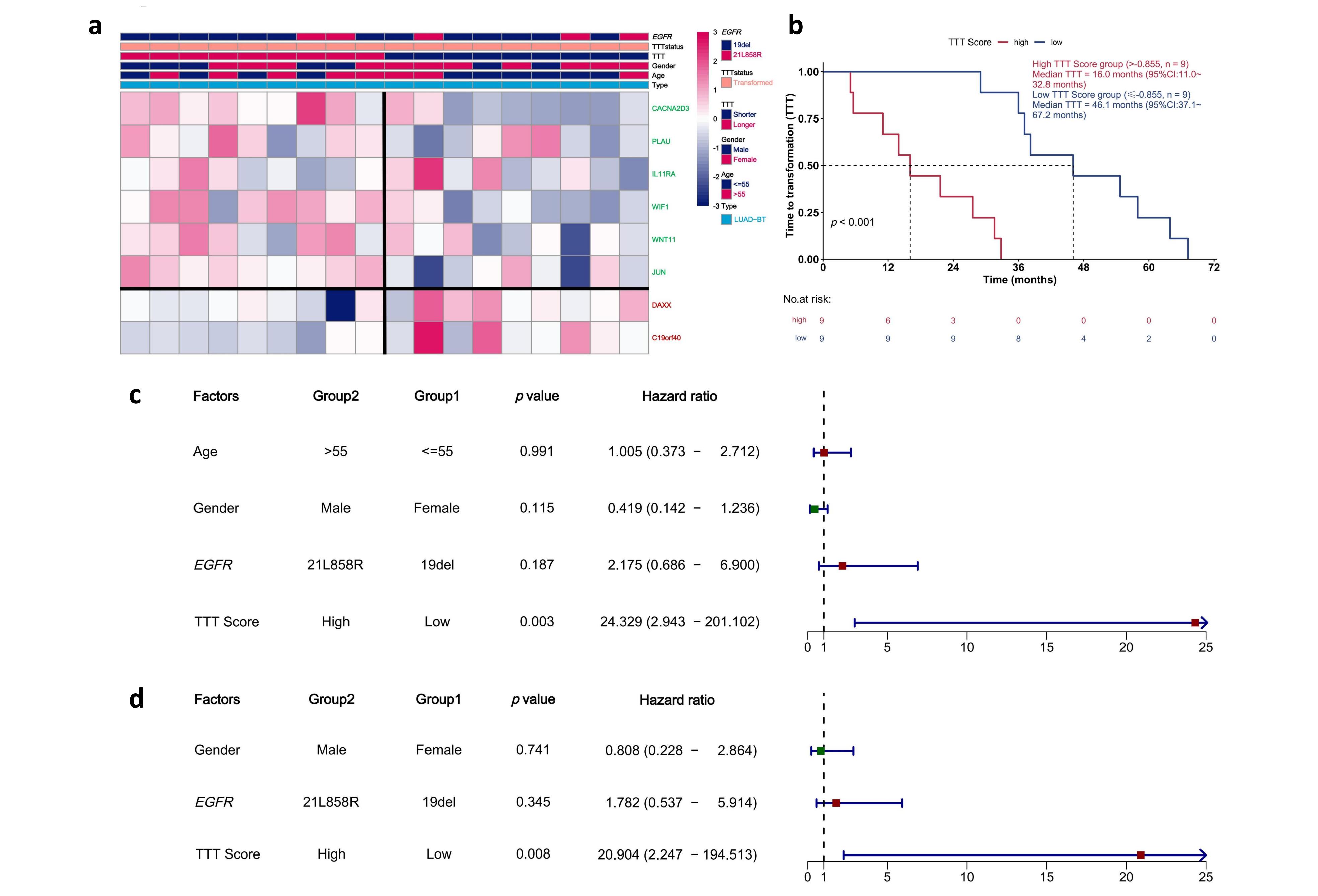


Figure. S12.

**Construction of TTT Model.** a). heatmap of 8 genes involved in the model showed distinct expression pattern between slow and rapid transformation patients (longer TTT versus shorter TTT) with LUAD-BT samples. b). Kaplan-Meier analysis of TTT in patients with LUAD-BT samples according to the median TTT Score. c). univariate Cox analysis for TTT in patients with LUAD-BT samples. d). multivariate Cox analysis for TTT in patients with LUAD-BT samples (variables with P<0.250 in univariate Cox analysis were enrolled in the multivariate analysis).


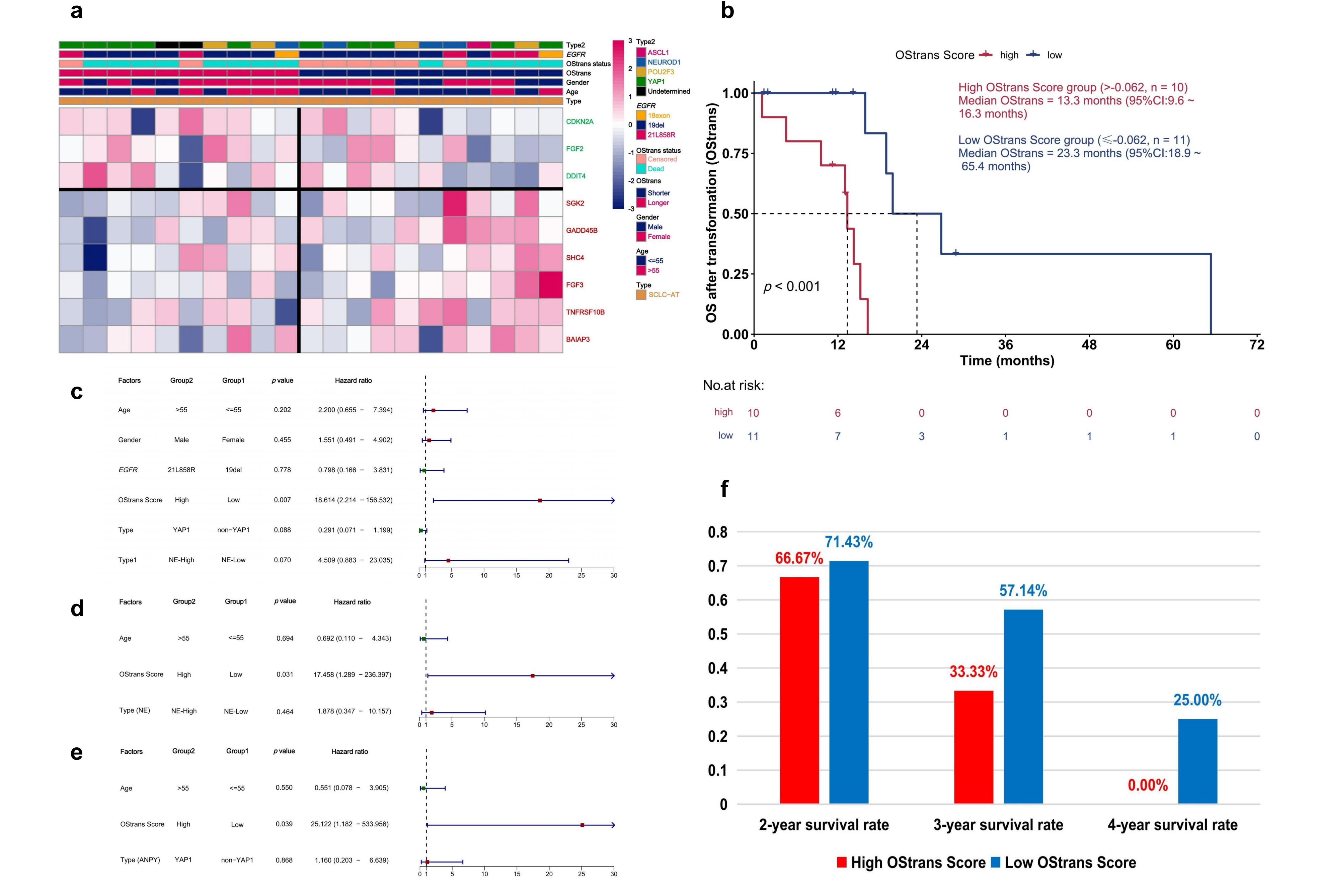


Figure. S13.

**Construction and validation of OStrans Model**. a). heatmap of 9 genes involved in the model showed distinct expression pattern between longer and shorter OStrans patients with SCLC-AT samples. b). Kaplan-Meier analysis of OStrans in patients with SCLC-AT samples according to the median OStrans Score. c). univariate Cox analysis for OS after transformation in patients with SCLC-AT samples. d). multivariate Cox analysis for OStrans in patients with SCLC-AT samples (For molecular subtype, patients were divided into NE-high group and NE-low group, and Subtype-A and Subtype-N were classified as NE-high group). e). multivariate Cox analysis for OStrans in patients with SCLC-AT samples (For molecular subtype, patients were divided into Subtype-Y group and non-Y group). f). comparison of 2-, 3-, and 4-years OS rate in SCLC-P patients according to OStrans Score.

Table S1.

Clinicopathological comparison of 100 patients (LUADs without SCLC transformation, LUADs with SCLC transformation, and primary SCLCs) enrolled in our study.

|  | **LUADs without SCLC transformation** | **LUADs with SCLC transformation** | **Primary SCLCs** |
| --- | --- | --- | --- |
| Total number | 33 | 40 | 27 |
| Age (mean±1.96SD) | 58.5 (43.5~73.4) | 52.8 (29.3~76.3) | 59.7 (41.8~77.6) |
| ≤55 | 11 (33.3%) | 23 (57.5%) | 11 (40.7%) |
| >55 | 22 (66.7%) | 17 (42.5%) | 16 (59.3%) |
| Gender | | | |
| Female | 23 (69.7%) | 25 (62.5%) | 4 (14.8%) |
| Male | 10 (30.3%) | 15 (37.5%) | 23 (85.2%) |
| *EGFR* | | | |
| 19del | 17 (51.5%) | 26 (65.0%) | - |
| L858R | 15 (45.5%)* | 11 (27.5%) | - |
| G719X | 1 (3.0%) | 3 (7.5%) | - |
| EGFR TKI | | | |
| 1st generation TKI | 3 (9.1%) | 15 (37.5%) | - |
| 1st+2nd generation TKI | 0 (0.0%) | 1 (2.5%) | - |
| 1st+3rd generation TKI | 27 (81.8%) | 16 (40.0%) | - |
| 2nd generation TKI | 0 (0.0%) | 1 (2.5%) | - |
| 2nd+3rd generation TKI | 1 (3.0%) | 3 (7.5%) | - |
| 3rd generation TKI | 2 (6.1%) | 4 (10.0%) | - |
| other *EGFR* mutation after 1st or 2nd generation TKI** | | | |
| T790M | 17 (54.8%) | 14 (38.9%) | - |
| T790M+A289I | 0 (0.0%) | 1 (2.8%) | - |
| no other EGFR mutation | 5 (16.1%) | 13 (36.1%) | - |
| NA | 9 (29.0%) | 8 (22.2%) | - |
| other *EGFR* mutation after 3rd generation TKI§ | | | |
| T790M | 4 (13.3%) | 3 (13.0%) |  |
| T790M+C797S | 1 (3.3%) | 2 (8.7%) | - |
| T790M+L718V | 1 (3.3%) | 0 (0.0%) | - |
| T790M+G796S | 1 (3.3%) | 0 (0.0%) | - |
| T790M+A289I | 0 (0.0%) | 0 (0.0%) | - |
| other *EGFR* mutation without T790M¶ | 4 (13.3%) | 0 (0.0%) | - |
| no other *EGFR* mutation | 5 (16.7%) | 4 (17.4%) | - |
| NA | 14 (46.7%) | 14 (60.9%) | - |
| *EGFR*: epidermal growth factor receptor. NA: Not Available. *: Two L858R-mutant LUADs without SCLC transformation had accompanying *EGFR* mutation, including L833V and V834L. **: For LUADs without SCLC transformation, 31 patients received 1st or 2nd generation TKI; for LUADs with SCLC transformation, 36 patients received 1st or 2nd generation TKI. §: For LUADs without SCLC transformation, 30 patients received 3rd generation TKI; for LUADs with SCLC transformation, 23 patients received 3rd generation TKI.  ¶: including L718V, L718Q, G724S, and L718V+C797S | | | |

Table S2.

Clinicopathological details of all 100 patients enrolled in the study.

| **sample id** | **Age** | **Gender** | ***EGFR*** | **IHC** | **nCounter** | **mIF** | **10x Genomics** |
| --- | --- | --- | --- | --- | --- | --- | --- |
| LUAD-NT1 | >55 | Female | 19del | 1 | 1 | 0 | 0 |
| LUAD-NT2 | >55 | Male | 19del | 1 | 1 | 0 | 0 |
| LUAD-NT3 | <=55 | Male | 19del | 1 | 1 | 0 | 0 |
| LUAD-NT4 | <=55 | Male | 19del | 1 | 1 | 0 | 0 |
| LUAD-NT5 | >55 | Female | 21L858R | 1 | 1 | 0 | 0 |
| LUAD-NT6 | >55 | Female | 19del | 1 | 1 | 0 | 0 |
| LUAD-NT7 | >55 | Male | 21L858R | 1 | 1 | 0 | 0 |
| LUAD-NT8 | >55 | Female | 21L858R | 1 | 1 | 0 | 0 |
| LUAD-NT9 | <=55 | Male | 21L858R | 1 | 1 | 0 | 0 |
| LUAD-NT10 | >55 | Male | 19del | 1 | 1 | 0 | 0 |
| LUAD-NT11 | >55 | Female | 21L858R | 1 | 1 | 0 | 0 |
| LUAD-NT12 | >55 | Female | 21L858R | 1 | 1 | 1 | 1 |
| LUAD-BT1,SCLC-AT1 | >55 | Female | 19del | 1 | 1 | 0 | 0 |
| LUAD-BT2,SCLC-AT2 | <=55 | Male | 21L858R | 1 | 1 | 0 | 0 |
| LUAD-BT3,SCLC-AT3 | >55 | Female | 21L858R | 1 | 1 | 0 | 0 |
| LUAD-BT4,SCLC-AT4 | <=55 | Male | 19del | 1 | 1 | 0 | 0 |
| LUAD-BT5,SCLC-AT5 | >55 | Female | 21L858R | 1 | 1 | 0 | 0 |
| LUAD-BT6,SCLC-AT6 | >55 | Male | 19del | 1 | 1 | 0 | 0 |
| LUAD-BT7,SCLC-AT7 | >55 | Female | 19del | 1 | 1 | 0 | 0 |
| LUAD-BT8,SCLC-AT8 | <=55 | Female | 19del | 1 | 1 | 0 | 0 |
| LUAD-BT9,SCLC-AT9 | <=55 | Male | 19del | 1 | 1 | 0 | 0 |
| LUAD-BT10,SCLC-AT10 | <=55 | Female | 19del | 1 | 1 | 0 | 0 |
| LUAD-BT11,SCLC-AT11 | <=55 | Female | 19del | 1 | 1 | 0 | 0 |
| LUAD-BT12,SCLC-AT12 | <=55 | Female | 19del | 1 | 1 | 0 | 0 |
| LUAD-BT13 | <=55 | Female | 21L858R | 1 | 1 | 0 | 0 |
| LUAD-BT14 | <=55 | Male | 19del | 1 | 1 | 0 | 0 |
| LUAD-BT15 | >55 | Female | 19del | 1 | 1 | 0 | 0 |
| LUAD-BT16 | >55 | Female | 19del | 1 | 1 | 0 | 0 |
| LUAD-BT17 | >55 | Male | 21L858R | 1 | 1 | 0 | 0 |
| LUAD-BT18 | <=55 | Male | 19del | 1 | 1 | 0 | 0 |
| SCLC-AT13 | <=55 | Female | 21L858R | 1 | 1 | 0 | 0 |
| SCLC-AT14 | <=55 | Female | 19del | 1 | 1 | 0 | 0 |
| SCLC-AT15 | >55 | Female | 19del | 1 | 1 | 0 | 0 |
| SCLC-AT16 | <=55 | Female | 21L858R | 1 | 1 | 0 | 0 |
| SCLC-AT17 | >55 | Male | 18exon | 1 | 1 | 0 | 0 |
| SCLC-AT18 | <=55 | Male | 19del | 1 | 1 | 0 | 0 |
| SCLC-AT19 | >55 | Female | 18exon | 1 | 1 | 0 | 0 |
| SCLC-AT20 | <=55 | Male | 19del | 1 | 1 | 0 | 0 |
| SCLC-AT21 | <=55 | Female | 19del | 1 | 1 | 0 | 0 |
| SCLC-P1 | >55 | Female | NA | 1 | 1 | 0 | 0 |
| SCLC-P2 | >55 | Male | NA | 1 | 1 | 0 | 0 |
| SCLC-P3 | <=55 | Male | NA | 1 | 1 | 0 | 0 |
| SCLC-P4 | >55 | Male | NA | 1 | 1 | 0 | 0 |
| SCLC-P5 | <=55 | Female | NA | 1 | 1 | 0 | 0 |
| SCLC-P6 | <=55 | Male | NA | 1 | 1 | 0 | 0 |
| SCLC-P7 | <=55 | Female | NA | 1 | 1 | 0 | 0 |
| SCLC-P8 | >55 | Male | NA | 1 | 1 | 0 | 0 |
| SCLC-P9 | >55 | Male | NA | 1 | 1 | 0 | 0 |
| SCLC-P10 | >55 | Male | NA | 1 | 1 | 0 | 0 |
| SCLC-P11 | >55 | Male | NA | 1 | 1 | 0 | 0 |
| SCLC-P12 | >55 | Male | NA | 1 | 1 | 0 | 0 |
| SCLC-P13 | >55 | Male | NA | 1 | 1 | 0 | 0 |
| SCLC-P14 | <=55 | Male | NA | 1 | 1 | 0 | 0 |
| SCLC-P15 | <=55 | Male | NA | 1 | 1 | 0 | 0 |
| SCLC-P16 | >55 | Male | NA | 1 | 1 | 0 | 0 |
| SCLC-P17 | >55 | Male | NA | 1 | 1 | 0 | 0 |
| SCLC-P18 | >55 | Male | NA | 1 | 1 | 0 | 0 |
| SCLC-P19 | >55 | Male | NA | 1 | 1 | 0 | 0 |
| SCLC-P20 | >55 | Male | NA | 1 | 1 | 0 | 0 |
| SCLC-P21 | <=55 | Female | NA | 1 | 0 | 0 | 0 |
| SCLC-P22 | <=55 | Male | NA | 1 | 0 | 0 | 0 |
| SCLC-P23 | <=55 | Male | NA | 1 | 0 | 0 | 0 |
| SCLC-P24 | <=55 | Male | NA | 1 | 0 | 0 | 0 |
| SCLC-P25 | <=55 | Male | NA | 1 | 0 | 0 | 0 |
| LUAD with SCLC transformation (samples unavailable) | <=55 | Male | 21L858R | 0 | 0 | 0 | 0 |
| LUAD with SCLC transformation (samples unavailable) | >55 | Female | 19del | 0 | 0 | 0 | 0 |
| LUAD with SCLC transformation (samples unavailable) | >55 | Female | 19del | 0 | 0 | 0 | 0 |
| LUAD with SCLC transformation (samples unavailable) | >55 | Female | 21L858R | 0 | 0 | 0 | 0 |
| LUAD-NT13 | >55 | Female | 21L858R | 1 | 0 | 1 | 1 |
| LUAD-NT14 | >55 | Female | 21L858R | 1 | 0 | 0 | 0 |
| LUAD-NT15 | >55 | Female | 19del | 1 | 0 | 0 | 0 |
| LUAD-NT16 | >55 | Male | 21L858R | 1 | 0 | 0 | 0 |
| LUAD-NT17 | <=55 | Female | 21L858R | 1 | 0 | 0 | 0 |
| LUAD-NT18 | <=55 | Female | 21L858R | 1 | 0 | 0 | 0 |
| LUAD-NT19 | >55 | Female | 19del | 1 | 0 | 0 | 0 |
| LUAD-NT20 | >55 | Female | 21L858R | 1 | 0 | 0 | 0 |
| LUAD-NT21 | >55 | Male | 18exon | 1 | 0 | 0 | 0 |
| LUAD-NT22 | <=55 | Male | 19del | 1 | 0 | 0 | 0 |
| LUAD-NT23 | <=55 | Female | 19del | 1 | 0 | 0 | 0 |
| LUAD-NT24 | <=55 | Female | 19del | 1 | 0 | 0 | 0 |
| LUAD-NT25 | >55 | Female | 19del | 1 | 0 | 0 | 0 |
| LUAD-NT26 | >55 | Female | 19del | 1 | 0 | 0 | 0 |
| LUAD-NT27 | <=55 | Female | 21L858R | 1 | 0 | 0 | 0 |
| LUAD-NT28 | >55 | Female | 19del | 1 | 0 | 0 | 0 |
| LUAD-NT29 | >55 | Female | 21L858R | 1 | 0 | 0 | 0 |
| LUAD-NT30 | >55 | Female | 19del | 1 | 0 | 0 | 0 |
| LUAD-NT31 | >55 | Male | 21L858R | 1 | 0 | 0 | 0 |
| LUAD-NT32 | <=55 | Female | 19del | 1 | 0 | 0 | 0 |
| LUAD-NT33 | <=55 | Female | 19del | 1 | 0 | 0 | 0 |
| LUAD-BT22,SCLC-AT22 | <=55 | Female | 19del | 1 | 0 | 1 | 1 |
| LUAD-BT23,SCLC-AT23 | <=55 | Male | 19del | 1 | 0 | 1 | 1 |
| LUAD-BT24,SCLC-AT24 | <=55 | Male | 19del | 1 | 0 | 1 | 1 |
| LUAD-BT25 | <=55 | Female | 18exon | 1 | 0 | 0 | 0 |
| LUAD-BT26 | <=55 | Male | 19del | 1 | 0 | 0 | 0 |
| LUAD-BT27 | <=55 | Female | 19del | 1 | 0 | 0 | 0 |
| LUAD-BT28 | >55 | Male | 21L858R | 1 | 0 | 0 | 0 |
| LUAD-BT29 | >55 | Female | 21L858R | 1 | 0 | 0 | 0 |
| LUAD-BT30 | >55 | Female | 19del | 1 | 0 | 0 | 0 |
| SCLC-P26 | >55 | Male | NA | 0 | 0 | 1 | 1 |
| SCLC-P27 | >55 | Male | NA | 0 | 0 | 1 | 1 |

Table S3.

Details of statistically significant DEGs (raw P<0.05, FDR<0.25) of LUAD-BT versus SCLC-AT

| Gene | Unpaired | | | | Paired | | | | Down or Up-regulated during transformation |
| --- | --- | --- | --- | --- | --- | --- | --- | --- | --- |
|  | LUAD-BTMedian | SCLC-ATMedian | p | fdr | LUAD-BTMedian | SCLC-ATMedian | p | fdr |  |
| *ITGB4* | 8.33963 | 5.71193 | 0.00000 | 0.00011 | 8.95524 | 5.08796 | 0.00049 | 0.02742 | Down |
| *EGFR* | 9.95686 | 8.08441 | 0.00000 | 0.00049 | 9.84789 | 8.31204 | 0.00049 | 0.02742 | Down |
| *NFKBIZ* | 8.99666 | 7.53203 | 0.00000 | 0.00063 | 9.06549 | 7.55562 | 0.00049 | 0.02742 | Down |
| *CASP7* | 6.53069 | 5.09075 | 0.00000 | 0.00063 | 6.46483 | 4.99246 | 0.00049 | 0.02742 | Down |
| *ERBB2* | 9.44552 | 7.10462 | 0.00001 | 0.00093 | 9.46580 | 7.64338 | 0.00244 | 0.04456 | Down |
| *LAMC2* | 6.97229 | 5.04442 | 0.00001 | 0.00114 | 7.61880 | 4.63108 | 0.00146 | 0.03564 | Down |
| *TNFSF10* | 9.76666 | 8.21144 | 0.00001 | 0.00120 | 9.76666 | 8.27523 | 0.00928 | 0.07525 | Down |
| *CARD11* | 5.38113 | 3.84353 | 0.00002 | 0.00151 | 5.92020 | 3.89839 | 0.00098 | 0.02970 | Down |
| *ETV7* | 5.82262 | 4.42629 | 0.00003 | 0.00174 | 5.59169 | 4.39802 | 0.00488 | 0.06041 | Down |
| *LAMB3* | 7.13209 | 4.76063 | 0.00004 | 0.00189 | 7.13209 | 3.61820 | 0.00049 | 0.02742 | Down |
| *CAPN2* | 10.83403 | 9.03024 | 0.00005 | 0.00207 | 10.46008 | 8.63919 | 0.00098 | 0.02970 | Down |
| *ITGA2* | 8.32150 | 6.13061 | 0.00006 | 0.00248 | 8.18739 | 6.09148 | 0.00049 | 0.02742 | Down |
| *IL1R1* | 7.78936 | 6.38565 | 0.00006 | 0.00248 | 7.92045 | 6.41307 | 0.00928 | 0.07525 | Down |
| *TLR2* | 7.45737 | 5.72268 | 0.00007 | 0.00273 | 7.30836 | 5.81553 | 0.00244 | 0.04456 | Down |
| *CASP8* | 8.85864 | 7.49739 | 0.00009 | 0.00346 | 8.94504 | 7.28531 | 0.02686 | 0.11267 | Down |
| *PML* | 9.60082 | 8.21766 | 0.00013 | 0.00416 | 9.27230 | 8.07314 | 0.02686 | 0.11267 | Down |
| *ITGB6* | 8.37664 | 6.27045 | 0.00014 | 0.00438 | 8.26475 | 6.27209 | 0.00928 | 0.07525 | Down |
| *TNFRSF10B* | 6.55900 | 5.37170 | 0.00017 | 0.00464 | 6.72875 | 4.81305 | 0.00146 | 0.03564 | Down |
| *BAD* | 7.78272 | 6.70860 | 0.00017 | 0.00464 | 7.81529 | 6.50660 | 0.00098 | 0.02970 | Down |
| *LIF* | 6.55074 | 4.83489 | 0.00019 | 0.00511 | 6.88439 | 4.36589 | 0.02100 | 0.10084 | Down |
| *DUSP5* | 7.92111 | 6.72085 | 0.00025 | 0.00643 | 7.98439 | 6.87359 | 0.01611 | 0.09118 | Down |
| *STAT3* | 10.62459 | 9.38269 | 0.00036 | 0.00882 | 10.63876 | 9.18009 | 0.04248 | 0.14291 | Down |
| *MAP3K8* | 6.99994 | 5.27688 | 0.00041 | 0.00967 | 6.92518 | 5.46121 | 0.02100 | 0.10084 | Down |
| *EGF* | 5.33318 | 3.27322 | 0.00047 | 0.01029 | 4.85302 | 3.28369 | 0.01611 | 0.09118 | Down |
| *GNG12* | 9.20963 | 7.78778 | 0.00047 | 0.01029 | 9.20962 | 7.75994 | 0.01611 | 0.09118 | Down |
| *ITGA3* | 8.99306 | 8.07531 | 0.00067 | 0.01356 | 8.93006 | 8.01720 | 0.00244 | 0.04456 | Down |
| *NFKBIA* | 10.61743 | 9.38126 | 0.00075 | 0.01446 | 10.49302 | 9.27031 | 0.00342 | 0.05198 | Down |
| *CREB3L1* | 8.97162 | 7.26651 | 0.00095 | 0.01691 | 8.69986 | 6.40424 | 0.00342 | 0.05198 | Down |
| *TNFAIP3* | 8.34980 | 6.53595 | 0.00166 | 0.02576 | 8.76947 | 6.28398 | 0.00488 | 0.06041 | Down |
| *EPHA2* | 6.85982 | 5.53991 | 0.00166 | 0.02576 | 6.65415 | 5.05897 | 0.00098 | 0.02970 | Down |
| *STAT4* | 6.02025 | 5.25727 | 0.00166 | 0.02576 | 6.10517 | 5.03745 | 0.00928 | 0.07525 | Down |
| *NFKB1* | 6.46224 | 5.32449 | 0.00166 | 0.02576 | 6.28981 | 5.26967 | 0.01221 | 0.08101 | Down |
| *TMPRSS2* | 7.63948 | 4.73256 | 0.00185 | 0.02632 | 7.63948 | 4.48077 | 0.02100 | 0.10084 | Down |
| *FOS* | 10.41674 | 8.67571 | 0.00185 | 0.02632 | 10.41674 | 8.85076 | 0.01611 | 0.09118 | Down |
| *GADD45B* | 8.30054 | 6.89573 | 0.00185 | 0.02632 | 8.58787 | 7.18290 | 0.00146 | 0.03564 | Down |
| *DTX4* | 8.28021 | 6.25940 | 0.00205 | 0.02632 | 8.31189 | 6.37435 | 0.03418 | 0.12730 | Down |
| *BIRC3* | 5.84997 | 4.27373 | 0.00205 | 0.02632 | 6.00942 | 4.25968 | 0.03418 | 0.12730 | Down |
| *PRDM1* | 7.11365 | 5.39552 | 0.00205 | 0.02632 | 7.11365 | 5.55839 | 0.01221 | 0.08101 | Down |
| *MMP9* | 9.36071 | 7.40537 | 0.00253 | 0.03134 | 9.63706 | 6.22267 | 0.00684 | 0.06931 | Down |
| *JAK3* | 6.81899 | 5.28146 | 0.00253 | 0.03134 | 7.30245 | 4.35063 | 0.00049 | 0.02742 | Down |
| *MMP7* | 8.23478 | 6.17710 | 0.00281 | 0.03306 | 7.83454 | 5.40104 | 0.00928 | 0.07525 | Down |
| *PLCE1* | 7.01814 | 5.47408 | 0.00281 | 0.03306 | 6.84946 | 5.48097 | 0.02100 | 0.10084 | Down |
| *SFN* | 8.90845 | 8.03634 | 0.00311 | 0.03386 | 8.65886 | 8.03190 | 0.02100 | 0.10084 | Down |
| *IL1RAP* | 7.48135 | 5.51252 | 0.00344 | 0.03584 | 7.51989 | 5.32604 | 0.00684 | 0.06931 | Down |
| *B2M* | 14.51406 | 12.81227 | 0.00344 | 0.03584 | 14.71455 | 12.70103 | 0.00244 | 0.04456 | Down |
| *TNFRSF10A* | 5.59107 | 3.91873 | 0.00380 | 0.03902 | 5.59107 | 3.90683 | 0.02686 | 0.11267 | Down |
| *PLAU* | 8.79172 | 7.39841 | 0.00419 | 0.04245 | 8.51431 | 6.59505 | 0.00928 | 0.07525 | Down |
| *SPRY4* | 7.78160 | 6.71615 | 0.00461 | 0.04431 | 7.75568 | 6.96910 | 0.01611 | 0.09118 | Down |
| *DLL4* | 6.67640 | 5.89416 | 0.00508 | 0.04634 | 6.76429 | 5.85589 | 0.02686 | 0.11267 | Down |
| *TGFBR2* | 8.89113 | 8.06566 | 0.00558 | 0.04852 | 8.97958 | 7.82718 | 0.01221 | 0.08101 | Down |
| *MAPK3* | 8.63520 | 8.30559 | 0.00558 | 0.04852 | 8.69449 | 8.29279 | 0.02100 | 0.10084 | Down |
| *EFNA1* | 8.17911 | 6.31641 | 0.00613 | 0.05087 | 8.04731 | 6.34388 | 0.04248 | 0.14291 | Down |
| *CLCF1* | 4.59040 | 3.83069 | 0.00613 | 0.05087 | 4.49484 | 3.47643 | 0.04248 | 0.14291 | Down |
| *ITGB7* | 5.41566 | 4.64868 | 0.00807 | 0.06405 | 5.47416 | 4.24815 | 0.00928 | 0.07525 | Down |
| *HSPB1* | 10.01278 | 9.46481 | 0.00883 | 0.06855 | 10.01278 | 9.34190 | 0.00098 | 0.02970 | Down |
| *ETV4* | 6.97208 | 5.53595 | 0.00964 | 0.07040 | 7.15431 | 5.76587 | 0.03418 | 0.12730 | Down |
| *FUT8* | 8.15770 | 7.29357 | 0.00964 | 0.07040 | 7.87254 | 6.50468 | 0.02686 | 0.11267 | Down |
| *SHC1* | 9.65723 | 8.56366 | 0.01052 | 0.07387 | 9.68708 | 8.54090 | 0.00244 | 0.04456 | Down |
| *NOTCH2* | 9.53848 | 8.91037 | 0.01147 | 0.07546 | 9.52659 | 8.44372 | 0.04248 | 0.14291 | Down |
| *ETS2* | 9.75994 | 8.75498 | 0.01147 | 0.07546 | 9.49026 | 8.69968 | 0.00928 | 0.07525 | Down |
| *CRLF2* | 3.00571 | 2.23703 | 0.01147 | 0.07546 | 2.79598 | 2.09442 | 0.01611 | 0.09118 | Down |
| *JAK2* | 8.10790 | 6.94263 | 0.01360 | 0.08272 | 8.27893 | 6.05294 | 0.01221 | 0.08101 | Down |
| *IRAK2* | 5.64067 | 4.11448 | 0.01360 | 0.08272 | 5.73459 | 3.72621 | 0.00098 | 0.02970 | Down |
| *HDAC1* | 8.33194 | 7.77765 | 0.01360 | 0.08272 | 8.35488 | 7.46279 | 0.00342 | 0.05198 | Down |
| *RBX1* | 9.87848 | 9.48568 | 0.01360 | 0.08272 | 9.86409 | 9.43432 | 0.02100 | 0.10084 | Down |
| *PLD1* | 6.32066 | 3.89209 | 0.01478 | 0.08918 | 6.72065 | 3.76893 | 0.02686 | 0.11267 | Down |
| *IL7R* | 7.49351 | 6.21766 | 0.01605 | 0.09300 | 7.58242 | 6.30961 | 0.04248 | 0.14291 | Down |
| *HDAC10* | 7.63433 | 7.01781 | 0.01605 | 0.09300 | 7.88609 | 6.74900 | 0.01611 | 0.09118 | Down |
| *JUN* | 8.68363 | 8.04116 | 0.01741 | 0.09704 | 8.68363 | 7.86124 | 0.04248 | 0.14291 | Down |
| *RPS27A* | 13.26182 | 12.82262 | 0.01741 | 0.09704 | 13.48810 | 12.76838 | 0.00488 | 0.06041 | Down |
| *IRAK3* | 5.66161 | 3.81762 | 0.01887 | 0.10131 | 5.66161 | 3.31164 | 0.02686 | 0.11267 | Down |
| *RAC1* | 9.64302 | 9.04773 | 0.01887 | 0.10131 | 9.64302 | 9.17294 | 0.01221 | 0.08101 | Down |
| *INHBA* | 6.98064 | 4.81762 | 0.02044 | 0.10811 | 7.23608 | 4.40654 | 0.03418 | 0.12730 | Down |
| *IL2RA* | 5.25141 | 4.21402 | 0.02211 | 0.11286 | 5.65337 | 3.85595 | 0.02100 | 0.10084 | Down |
| *IKBKB* | 7.45357 | 6.51444 | 0.02211 | 0.11286 | 7.79459 | 6.24950 | 0.02100 | 0.10084 | Down |
| *DDB2* | 6.76742 | 5.71652 | 0.02211 | 0.11286 | 6.80241 | 5.40553 | 0.01221 | 0.08101 | Down |
| *RASGRP2* | 5.80973 | 4.58407 | 0.02580 | 0.12557 | 6.22666 | 4.67142 | 0.02686 | 0.11267 | Down |
| *PLAT* | 8.34236 | 7.35598 | 0.02580 | 0.12557 | 8.34236 | 7.39263 | 0.02686 | 0.11267 | Down |
| *ITGB8* | 8.15461 | 6.82002 | 0.02783 | 0.13109 | 8.15461 | 7.02474 | 0.01611 | 0.09118 | Down |
| *MAP2K2* | 9.08500 | 8.56745 | 0.02783 | 0.13109 | 9.08500 | 7.98678 | 0.02686 | 0.11267 | Down |
| *CTNNB1* | 10.89462 | 10.45694 | 0.02783 | 0.13109 | 10.91895 | 10.21247 | 0.01221 | 0.08101 | Down |
| *RIN1* | 5.49025 | 4.51737 | 0.03230 | 0.14380 | 5.79105 | 4.14345 | 0.00928 | 0.07525 | Down |
| *PLCG2* | 6.69738 | 5.58844 | 0.03476 | 0.15102 | 6.95241 | 5.02201 | 0.01221 | 0.08101 | Down |
| *SPP1* | 11.39546 | 10.42711 | 0.04305 | 0.16989 | 11.35647 | 9.12963 | 0.00928 | 0.07525 | Down |
| *IGF1R* | 8.68467 | 7.04434 | 0.04616 | 0.17104 | 8.89549 | 6.83099 | 0.02100 | 0.10084 | Down |
| *THBS1* | 9.67317 | 8.04457 | 0.04616 | 0.17104 | 9.67317 | 7.80255 | 0.02686 | 0.11267 | Down |
| *RUNX1* | 9.42434 | 8.24671 | 0.04616 | 0.17104 | 9.26558 | 7.46533 | 0.00928 | 0.07525 | Down |
| *ETV1* | 7.33906 | 5.85517 | 0.04616 | 0.17104 | 7.33906 | 5.68882 | 0.00488 | 0.06041 | Down |
| *MAPK8IP1* | 4.39068 | 7.30375 | 0.00000 | 0.00049 | 4.64451 | 7.52600 | 0.00342 | 0.05198 | Up |
| *CAMK2B* | 3.63503 | 6.75673 | 0.00001 | 0.00076 | 3.53870 | 6.97639 | 0.01611 | 0.09118 | Up |
| *ZIC2* | 5.77080 | 8.88122 | 0.00002 | 0.00161 | 5.77080 | 9.81944 | 0.00049 | 0.02742 | Up |
| *GNG4* | 5.42004 | 8.51170 | 0.00003 | 0.00174 | 6.18520 | 8.52280 | 0.00146 | 0.03564 | Up |
| *MAPT* | 3.36126 | 6.28472 | 0.00003 | 0.00174 | 3.36127 | 5.79604 | 0.02100 | 0.10084 | Up |
| *CNTFR* | 4.63114 | 7.19244 | 0.00011 | 0.00379 | 4.63114 | 7.06900 | 0.00049 | 0.02742 | Up |
| *DLL3* | 3.38845 | 7.29327 | 0.00014 | 0.00438 | 3.05345 | 8.10288 | 0.00098 | 0.02970 | Up |
| *ID4* | 6.93433 | 8.19418 | 0.00053 | 0.01129 | 6.72702 | 8.17452 | 0.00684 | 0.06931 | Up |
| *CACNA2D1* | 6.22314 | 9.07843 | 0.00075 | 0.01446 | 6.46238 | 8.78763 | 0.03418 | 0.12730 | Up |
| *FGF12* | 3.83404 | 6.37603 | 0.00085 | 0.01584 | 3.83404 | 6.41111 | 0.00244 | 0.04456 | Up |
| *SMAD9* | 6.26628 | 8.16184 | 0.00095 | 0.01691 | 5.94235 | 8.86546 | 0.00244 | 0.04456 | Up |
| *TTK* | 7.38812 | 8.14511 | 0.00119 | 0.02071 | 7.38812 | 8.54974 | 0.00098 | 0.02970 | Up |
| *FGF9* | 3.97039 | 6.20022 | 0.00185 | 0.02632 | 3.50511 | 6.39164 | 0.00684 | 0.06931 | Up |
| *TIAM1* | 7.25781 | 8.18591 | 0.00311 | 0.03386 | 7.42326 | 8.28835 | 0.00098 | 0.02970 | Up |
| *PBRM1* | 8.15903 | 8.99328 | 0.00311 | 0.03386 | 8.12555 | 9.08871 | 0.00488 | 0.06041 | Up |
| *COL2A1* | 4.46118 | 7.59367 | 0.00311 | 0.03386 | 4.17812 | 7.54014 | 0.02100 | 0.10084 | Up |
| *AMH* | 4.50242 | 5.12090 | 0.00461 | 0.04431 | 4.50738 | 5.32170 | 0.01221 | 0.08101 | Up |
| *PPP2R2C* | 2.51995 | 4.89573 | 0.00508 | 0.04634 | 2.15687 | 4.61542 | 0.00684 | 0.06931 | Up |
| *DTX1* | 5.31282 | 6.89573 | 0.00558 | 0.04852 | 5.31282 | 7.49202 | 0.00342 | 0.05198 | Up |
| *PGF* | 6.12722 | 8.40833 | 0.00558 | 0.04852 | 5.83425 | 8.17245 | 0.00928 | 0.07525 | Up |
| *MAD2L2* | 7.54496 | 8.62662 | 0.00613 | 0.05087 | 7.48875 | 8.76951 | 0.01221 | 0.08101 | Up |
| *HELLS* | 7.94402 | 9.02202 | 0.00613 | 0.05087 | 7.62984 | 9.12100 | 0.03418 | 0.12730 | Up |
| *LIFR* | 8.76351 | 9.81198 | 0.00883 | 0.06855 | 8.82275 | 9.97621 | 0.00342 | 0.05198 | Up |
| *BDNF* | 3.55678 | 5.04374 | 0.00964 | 0.07040 | 3.02868 | 4.61102 | 0.00488 | 0.06041 | Up |
| *CACNA1E* | 3.66974 | 4.89573 | 0.00964 | 0.07040 | 3.16337 | 4.93737 | 0.00684 | 0.06931 | Up |
| *FST* | 5.20114 | 6.85983 | 0.00964 | 0.07040 | 5.03484 | 7.23648 | 0.01611 | 0.09118 | Up |
| *FZD9* | 3.78048 | 5.19215 | 0.00964 | 0.07040 | 3.52649 | 5.90836 | 0.01221 | 0.08101 | Up |
| *BCL2* | 6.65414 | 7.61547 | 0.01147 | 0.07546 | 6.78721 | 7.31826 | 0.03418 | 0.12730 | Up |
| *CALML3* | 2.53385 | 4.79162 | 0.01147 | 0.07546 | 2.53385 | 5.44408 | 0.01611 | 0.09118 | Up |
| *TFDP1* | 9.35225 | 10.42638 | 0.01360 | 0.08272 | 9.26858 | 10.70658 | 0.00049 | 0.02742 | Up |
| *FANCB* | 6.00955 | 7.31641 | 0.01360 | 0.08272 | 5.69319 | 7.35162 | 0.00049 | 0.02742 | Up |
| *CCNA1* | 3.11787 | 4.15681 | 0.01605 | 0.09300 | 3.26766 | 4.76298 | 0.00928 | 0.07525 | Up |
| *FANCC* | 7.51199 | 8.18857 | 0.01741 | 0.09704 | 7.34019 | 8.30047 | 0.00342 | 0.05198 | Up |
| *CDKN1C* | 7.65545 | 8.36676 | 0.01887 | 0.10131 | 7.69994 | 8.66680 | 0.00244 | 0.04456 | Up |
| *PAK3* | 2.85127 | 4.29333 | 0.02044 | 0.10811 | 2.85127 | 4.60603 | 0.00928 | 0.07525 | Up |
| *HES5* | 4.05833 | 5.79145 | 0.02211 | 0.11286 | 3.61133 | 5.96896 | 0.01221 | 0.08101 | Up |
| *TLX1* | 2.97497 | 5.16325 | 0.02580 | 0.12557 | 2.59661 | 5.43172 | 0.00488 | 0.06041 | Up |
| *TNN* | 2.51729 | 3.85147 | 0.02783 | 0.13109 | 2.20096 | 4.08291 | 0.02686 | 0.11267 | Up |
| *HOXA9* | 4.48724 | 6.22720 | 0.02783 | 0.13109 | 4.20125 | 6.69722 | 0.01611 | 0.09118 | Up |
| *MCM2* | 7.88196 | 8.95802 | 0.03230 | 0.14380 | 7.95120 | 8.97787 | 0.02686 | 0.11267 | Up |
| *DKK4* | 2.94896 | 4.26651 | 0.03230 | 0.14380 | 2.82437 | 5.01954 | 0.00488 | 0.06041 | Up |
| *IFNA17* | 2.58170 | 4.23707 | 0.03230 | 0.14380 | 2.06726 | 4.31092 | 0.00684 | 0.06931 | Up |
| *BCOR* | 7.48423 | 8.19199 | 0.03476 | 0.15102 | 7.31975 | 8.44367 | 0.04248 | 0.14291 | Up |
| *MUTYH* | 5.84478 | 6.78286 | 0.04012 | 0.16548 | 5.64950 | 6.90230 | 0.00049 | 0.02742 | Up |
| *FGF5* | 1.65818 | 3.51642 | 0.04012 | 0.16548 | 1.04258 | 3.16216 | 0.01611 | 0.09118 | Up |
| *DNMT3A* | 8.27998 | 8.72804 | 0.04305 | 0.16989 | 8.26384 | 9.03830 | 0.00342 | 0.05198 | Up |
| *AR* | 5.55043 | 6.32894 | 0.04616 | 0.17104 | 5.75498 | 6.95937 | 0.02100 | 0.10084 | Up |
| *WNT16* | 2.51682 | 4.15681 | 0.04616 | 0.17104 | 2.31661 | 4.19702 | 0.04248 | 0.14291 | Up |
| *PAK7* | 1.86998 | 3.94307 | 0.04944 | 0.17957 | 1.62336 | 3.99544 | 0.01221 | 0.08101 | Up |

Table S4.

The weighted average expression details of up-/down-regulated DEGs for each sample in four groups.

| **id** | **Type** | **EGFR** | **Weighted average up−regulated genes expression (log2)** | **Weighted average down−regulated genes expression (log2)** |
| --- | --- | --- | --- | --- |
| LUAD-BT1 | LUAD-BT | 19del | 4.890283963 | 8.546455242 |
| LUAD-BT10 | LUAD-BT | 19del | 4.047911882 | 8.674395594 |
| LUAD-BT11 | LUAD-BT | 19del | 4.665793618 | 8.087160862 |
| LUAD-BT12 | LUAD-BT | 19del | 5.628602929 | 7.379382065 |
| LUAD-BT13 | LUAD-BT | NA | 4.746557817 | 7.178834466 |
| LUAD-BT14 | LUAD-BT | NA | 5.92591654 | 7.08058888 |
| LUAD-BT15 | LUAD-BT | NA | 4.374045925 | 8.600112504 |
| LUAD-BT16 | LUAD-BT | NA | 4.599268907 | 8.116963753 |
| LUAD-BT17 | LUAD-BT | NA | 6.391396702 | 8.359346109 |
| LUAD-BT18 | LUAD-BT | NA | 4.764477749 | 9.109882653 |
| LUAD-BT2 | LUAD-BT | 21L858R | 4.446508445 | 8.652394653 |
| LUAD-BT3 | LUAD-BT | 21L858R | 6.503625327 | 6.621340329 |
| LUAD-BT4 | LUAD-BT | 19del | 4.559481249 | 8.87308654 |
| LUAD-BT5 | LUAD-BT | 21L858R | 3.628264166 | 8.337335775 |
| LUAD-BT6 | LUAD-BT | 19del | 4.940889008 | 8.773446868 |
| LUAD-BT7 | LUAD-BT | 19del | 4.727948817 | 8.644099201 |
| LUAD-BT8 | LUAD-BT | 19del | 4.9355152 | 9.087834249 |
| LUAD-BT9 | LUAD-BT | 19del | 4.93155828 | 7.659486057 |
| LUAD-NT1 | LUAD-NT | NA | 5.298125617 | 7.661721951 |
| LUAD-NT10 | LUAD-NT | NA | 4.555673087 | 8.873384195 |
| LUAD-NT11 | LUAD-NT | NA | 4.757175666 | 9.211314018 |
| LUAD-NT12 | LUAD-NT | NA | 5.037364741 | 8.824142129 |
| LUAD-NT2 | LUAD-NT | NA | 5.754106798 | 8.036223124 |
| LUAD-NT3 | LUAD-NT | NA | 3.999994302 | 8.78043716 |
| LUAD-NT4 | LUAD-NT | NA | 4.736983771 | 9.024926627 |
| LUAD-NT5 | LUAD-NT | NA | 4.949772786 | 8.87022359 |
| LUAD-NT6 | LUAD-NT | NA | 4.630340129 | 9.250169009 |
| LUAD-NT7 | LUAD-NT | NA | 5.326162977 | 8.978537454 |
| LUAD-NT8 | LUAD-NT | NA | 5.318073229 | 9.387327362 |
| LUAD-NT9 | LUAD-NT | NA | 7.012378664 | 7.125889939 |
| SCLC-AT1 | SCLC-AT | 19del | 6.394021332 | 6.852748075 |
| SCLC-AT10 | SCLC-AT | 19del | 5.72501074 | 7.341537617 |
| SCLC-AT11 | SCLC-AT | 19del | 7.140425231 | 5.500192335 |
| SCLC-AT12 | SCLC-AT | 19del | 6.759803233 | 6.764029684 |
| SCLC-AT13 | SCLC-AT | NA | 6.999340904 | 6.480620948 |
| SCLC-AT14 | SCLC-AT | NA | 6.05165371 | 6.711934307 |
| SCLC-AT15 | SCLC-AT | NA | 7.180511295 | 7.083701373 |
| SCLC-AT16 | SCLC-AT | NA | 6.024498544 | 7.964023313 |
| SCLC-AT17 | SCLC-AT | NA | 6.071976019 | 6.613742435 |
| SCLC-AT18 | SCLC-AT | NA | 5.833402066 | 8.047824794 |
| SCLC-AT19 | SCLC-AT | NA | 6.90525718 | 6.575002539 |
| SCLC-AT2 | SCLC-AT | 21L858R | 6.639931111 | 7.027875276 |
| SCLC-AT20 | SCLC-AT | NA | 6.349404054 | 7.813188678 |
| SCLC-AT21 | SCLC-AT | NA | 6.944051499 | 7.104140891 |
| SCLC-AT3 | SCLC-AT | 21L858R | 7.401240841 | 5.86270667 |
| SCLC-AT4 | SCLC-AT | 19del | 6.442416453 | 5.670285899 |
| SCLC-AT5 | SCLC-AT | 21L858R | 6.424060923 | 7.873642996 |
| SCLC-AT6 | SCLC-AT | 19del | 5.366262813 | 6.550149139 |
| SCLC-AT7 | SCLC-AT | 19del | 6.333338836 | 6.904859844 |
| SCLC-AT8 | SCLC-AT | 19del | 7.019735947 | 6.556940494 |
| SCLC-AT9 | SCLC-AT | 19del | 6.175699293 | 7.673334705 |
| SCLC-P1 | SCLC-P | NA | 8.494223795 | 7.860038396 |
| SCLC-P10 | SCLC-P | NA | 7.506299743 | 8.582516631 |
| SCLC-P11 | SCLC-P | NA | 6.868096612 | 7.878031869 |
| SCLC-P12 | SCLC-P | NA | 6.939336785 | 6.803128196 |
| SCLC-P13 | SCLC-P | NA | 7.475026432 | 5.527091807 |
| SCLC-P14 | SCLC-P | NA | 6.977439437 | 8.092957259 |
| SCLC-P15 | SCLC-P | NA | 7.821396844 | 7.273909447 |
| SCLC-P16 | SCLC-P | NA | 7.108923679 | 6.803707916 |
| SCLC-P17 | SCLC-P | NA | 7.089859454 | 6.842355217 |
| SCLC-P18 | SCLC-P | NA | 4.652696523 | 7.290038898 |
| SCLC-P19 | SCLC-P | NA | 7.822974164 | 7.290463786 |
| SCLC-P2 | SCLC-P | NA | 7.215421435 | 7.083010079 |
| SCLC-P20 | SCLC-P | NA | 6.6814735 | 7.045188701 |
| SCLC-P3 | SCLC-P | NA | 7.451413872 | 6.347019066 |
| SCLC-P4 | SCLC-P | NA | 7.103170166 | 7.098373428 |
| SCLC-P5 | SCLC-P | NA | 7.863441463 | 6.980610068 |
| SCLC-P6 | SCLC-P | NA | 6.688864926 | 8.581616026 |
| SCLC-P7 | SCLC-P | NA | 7.606685337 | 6.814640213 |
| SCLC-P8 | SCLC-P | NA | 7.70147936 | 7.499756661 |
| SCLC-P9 | SCLC-P | NA | 8.411263159 | 7.770583401 |

Table S5.

The details of 1,350 genesets associated with tumor and TIME and their classification according to the information provided on the MSigDB.

| pathway | Type | BT vs NT | | | AT vs BT | | | AT vs P | | |
| --- | --- | --- | --- | --- | --- | --- | --- | --- | --- | --- |
|  |  | NES | p-value | q-value | NES | p-value | q-value | NES | p-value | q-value |
| HALLMARK_DNA_REPAIR | Genetic and epigenetic information | -0.80049 | 0.87085 | 0.88692 | 0.57118 | 0.99692 | 0.72077 | -1.59619 | 0.00081 | 0.02082 |
| HALLMARK_G2M_CHECKPOINT | Cell cycle | 1.01939 | 0.39375 | 0.71215 | 1.83077 | 0.00000 | 0.00009 | -1.92701 | 0.00005 | 0.00409 |
| HALLMARK_MITOTIC_SPINDLE | Cell cycle | 0.82914 | 0.92067 | 0.88692 | 1.25948 | 0.04472 | 0.10541 | 0.58427 | 0.99850 | 0.87060 |
| HALLMARK_GLYCOLYSIS | Metabolism and energy | 2.07772 | 0.00001 | 0.00041 | -1.85422 | 0.00015 | 0.00147 | 0.76574 | 0.92223 | 0.87060 |
| HALLMARK_OXIDATIVE_PHOSPHORYLATION | Metabolism and energy | -0.61940 | 0.99900 | 0.88692 | -1.06726 | 0.24850 | 0.35589 | -2.04625 | 0.00005 | 0.00409 |
| HALLMARK_INTERFERON_ALPHA_RESPONSE | Immunity | 1.95501 | 0.00004 | 0.00156 | -3.95428 | 0.00003 | 0.00060 | 0.71066 | 0.92766 | 0.87060 |
| HALLMARK_EPITHELIAL_MESENCHYMAL_TRANSITION | ECM and metastasis | 3.57029 | 0.00001 | 0.00041 | -3.62157 | 0.00017 | 0.00155 | 1.80448 | 0.00000 | 0.00035 |
| HALLMARK_IL2_STAT5_SIGNALING | Immunity | 1.04878 | 0.31702 | 0.65965 | -2.33593 | 0.00017 | 0.00157 | 0.78070 | 0.90943 | 0.87060 |
| HALLMARK_COMPLEMENT | Immunity | 1.14642 | 0.13909 | 0.46525 | -2.81071 | 0.00017 | 0.00154 | -1.52670 | 0.00057 | 0.01652 |
| HALLMARK_ANGIOGENESIS | ECM and metastasis | 2.04460 | 0.00027 | 0.00769 | -2.20569 | 0.00004 | 0.00062 | 1.20125 | 0.21294 | 0.51164 |
| HALLMARK_IL6_JAK_STAT3_SIGNALING | Immunity | 1.33107 | 0.05871 | 0.30658 | -2.93765 | 0.00002 | 0.00057 | -1.62060 | 0.00344 | 0.05271 |
| HALLMARK_TGF_BETA_SIGNALING | ECM and metastasis | 1.20138 | 0.17694 | 0.52745 | -1.72546 | 0.00156 | 0.00818 | 0.98688 | 0.50601 | 0.74084 |
| HALLMARK_APOPTOSIS | Cell death | 1.13593 | 0.17269 | 0.51892 | -2.28826 | 0.00008 | 0.00098 | -1.07142 | 0.26479 | 0.56595 |
| HALLMARK_TNFA_SIGNALING_VIA_NFKB | Immunity | -1.76465 | 0.00002 | 0.00095 | -3.19001 | 0.00017 | 0.00155 | -2.83938 | 0.00006 | 0.00425 |
| HALLMARK_INTERFERON_GAMMA_RESPONSE | Immunity | 1.35864 | 0.01549 | 0.13975 | -3.86164 | 0.00015 | 0.00147 | -1.45023 | 0.00142 | 0.02992 |
| HALLMARK_ADIPOGENESIS | Metabolism and energy | -1.15102 | 0.18375 | 0.53413 | -2.32447 | 0.00016 | 0.00151 | -1.12467 | 0.14010 | 0.42214 |
| HALLMARK_APICAL_JUNCTION | ECM and metastasis | 2.00225 | 0.00001 | 0.00041 | -2.18440 | 0.00018 | 0.00161 | 1.48549 | 0.00099 | 0.02381 |
| HALLMARK_CHOLESTEROL_HOMEOSTASIS | Metabolism and energy | -1.10535 | 0.29778 | 0.64182 | -2.00428 | 0.00005 | 0.00075 | -2.16749 | 0.00002 | 0.00289 |
| HALLMARK_FATTY_ACID_METABOLISM | Metabolism and energy | -1.29371 | 0.06340 | 0.31975 | -1.52762 | 0.00036 | 0.00270 | 0.68803 | 0.96576 | 0.87060 |
| KEGG_SPLICEOSOME | Genetic and epigenetic information | -0.45965 | 0.99999 | 0.88692 | 1.14251 | 0.22251 | 0.33012 | -1.84369 | 0.00009 | 0.00552 |
| KEGG_BASE_EXCISION_REPAIR | Genetic and epigenetic information | -0.46810 | 0.99646 | 0.88692 | 0.90915 | 0.62197 | 0.61630 | -0.86769 | 0.68052 | 0.82131 |
| KEGG_PROTEASOME | Immunity | 1.55415 | 0.02400 | 0.18447 | -1.27866 | 0.09747 | 0.18620 | -1.40771 | 0.05624 | 0.26830 |
| KEGG_RNA_POLYMERASE | Genetic and epigenetic information | -0.93249 | 0.56466 | 0.79732 | 0.67505 | 0.89627 | 0.72077 | -1.47362 | 0.05482 | 0.26551 |
| KEGG_GLYCEROPHOSPHOLIPID_METABOLISM | Metabolism and energy | -1.01708 | 0.43442 | 0.73677 | -1.19423 | 0.12146 | 0.21761 | 0.99115 | 0.50292 | 0.73875 |
| KEGG_PYRIMIDINE_METABOLISM | Genetic and epigenetic information | -0.67357 | 0.96985 | 0.88692 | 0.62635 | 0.97697 | 0.72077 | -1.03547 | 0.36978 | 0.65707 |
| KEGG_HOMOLOGOUS_RECOMBINATION | Genetic and epigenetic information | -0.54288 | 0.98173 | 0.88692 | 1.27235 | 0.15013 | 0.25243 | 0.40947 | 0.99884 | 0.87060 |
| KEGG_DNA_REPLICATION | Genetic and epigenetic information | 0.89316 | 0.62524 | 0.82089 | 1.50184 | 0.02117 | 0.06105 | -0.98778 | 0.46083 | 0.71866 |
| KEGG_VEGF_SIGNALING_PATHWAY | ECM and metastasis | -0.84763 | 0.74027 | 0.86073 | -1.25701 | 0.07232 | 0.14938 | 0.87028 | 0.71027 | 0.82947 |
| KEGG_CELL_CYCLE | Cell cycle | 0.95706 | 0.55662 | 0.79503 | 1.57659 | 0.00048 | 0.00336 | -1.22010 | 0.08084 | 0.32320 |
| KEGG_RNA_DEGRADATION | Genetic and epigenetic information | 0.80243 | 0.81552 | 0.88529 | 0.96570 | 0.54739 | 0.57797 | -0.84068 | 0.77632 | 0.85306 |
| KEGG_AMINOACYL_TRNA_BIOSYNTHESIS | Genetic and epigenetic information | -0.86511 | 0.65902 | 0.83341 | 1.13070 | 0.31038 | 0.41226 | 0.73402 | 0.83279 | 0.87041 |
| KEGG_LINOLEIC_ACID_METABOLISM | Metabolism and energy | -1.02558 | 0.43214 | 0.73536 | -1.22989 | 0.15967 | 0.26399 | 1.18151 | 0.24541 | 0.54296 |
| KEGG_LYSINE_DEGRADATION | Metabolism and energy | -0.73773 | 0.86601 | 0.88692 | 0.71498 | 0.88265 | 0.71995 | -0.80331 | 0.81759 | 0.86666 |
| KEGG_OXIDATIVE_PHOSPHORYLATION | Metabolism and energy | -0.80404 | 0.84615 | 0.88692 | -0.58064 | 0.99996 | 0.72077 | -1.78052 | 0.00022 | 0.00916 |
| KEGG_UBIQUITIN_MEDIATED_PROTEOLYSIS | Immunity | 0.60666 | 0.99953 | 0.88692 | 0.56342 | 0.99690 | 0.72077 | -1.79416 | 0.00008 | 0.00510 |
| KEGG_GLYCOSPHINGOLIPID_BIOSYNTHESIS_LACTO_AND_NEOLACTO_SERIES | Metabolism and energy | 0.92721 | 0.55271 | 0.79291 | 0.71113 | 0.86228 | 0.71434 | -0.99568 | 0.44643 | 0.70836 |
| KEGG_NUCLEOTIDE_EXCISION_REPAIR | Genetic and epigenetic information | 0.64632 | 0.96092 | 0.88692 | 0.89957 | 0.64572 | 0.62986 | -0.83725 | 0.75958 | 0.84603 |
| KEGG_GLYCEROLIPID_METABOLISM | Metabolism and energy | -1.25048 | 0.15344 | 0.48554 | 0.57639 | 0.97845 | 0.72077 | 0.53249 | 0.99094 | 0.87060 |
| KEGG_PHENYLALANINE_METABOLISM | Metabolism and energy | -1.29816 | 0.15193 | 0.48355 | 0.65297 | 0.90331 | 0.72077 | -1.39622 | 0.09364 | 0.34606 |
| KEGG_SELENOAMINO_ACID_METABOLISM | Metabolism and energy | -0.96001 | 0.52508 | 0.78007 | -0.65158 | 0.94491 | 0.72077 | 1.05655 | 0.40511 | 0.68539 |
| KEGG_FRUCTOSE_AND_MANNOSE_METABOLISM | Metabolism and energy | -0.54317 | 0.98259 | 0.88692 | -0.85327 | 0.71766 | 0.66103 | -1.37715 | 0.08146 | 0.32398 |
| KEGG_STEROID_BIOSYNTHESIS | Metabolism and energy | -1.58840 | 0.02135 | 0.17289 | -1.03526 | 0.39349 | 0.47830 | -2.35865 | 0.00012 | 0.00631 |
| KEGG_TYROSINE_METABOLISM | Metabolism and energy | -1.22379 | 0.18569 | 0.53752 | -1.04205 | 0.37701 | 0.46607 | -0.87840 | 0.67490 | 0.81885 |
| KEGG_PYRUVATE_METABOLISM | Metabolism and energy | -0.92858 | 0.57592 | 0.79864 | -0.59800 | 0.98923 | 0.72077 | 1.01600 | 0.45856 | 0.71792 |
| KEGG_CYSTEINE_AND_METHIONINE_METABOLISM | Metabolism and energy | 0.55024 | 0.98605 | 0.88692 | 0.57063 | 0.97132 | 0.72077 | -1.35286 | 0.08826 | 0.33878 |
| KEGG_PENTOSE_PHOSPHATE_PATHWAY | Metabolism and energy | 0.65473 | 0.92026 | 0.88692 | -1.12104 | 0.27478 | 0.38053 | -0.52645 | 0.99234 | 0.87060 |
| KEGG_MISMATCH_REPAIR | Genetic and epigenetic information | 0.62333 | 0.93853 | 0.88692 | 1.18239 | 0.24758 | 0.35516 | 0.55995 | 0.96930 | 0.87060 |
| KEGG_REGULATION_OF_AUTOPHAGY | Cell death | -1.08217 | 0.35424 | 0.68772 | 1.63084 | 0.00573 | 0.02246 | 1.01364 | 0.46222 | 0.71875 |
| KEGG_GLYCOLYSIS_GLUCONEOGENESIS | Metabolism and energy | 1.26866 | 0.12105 | 0.43428 | -1.53308 | 0.00979 | 0.03389 | 0.64674 | 0.95123 | 0.87060 |
| KEGG_CELL_ADHESION_MOLECULES_CAMS | ECM and metastasis | -1.36385 | 0.04178 | 0.25336 | 1.07532 | 0.34780 | 0.44265 | 0.89573 | 0.69119 | 0.82445 |
| KEGG_CHEMOKINE_SIGNALING_PATHWAY | Immunity | -1.26718 | 0.06880 | 0.33238 | -1.45331 | 0.00082 | 0.00496 | -1.46067 | 0.00119 | 0.02728 |
| KEGG_TGF_BETA_SIGNALING_PATHWAY | ECM and metastasis | 1.82959 | 0.00045 | 0.01163 | 0.76001 | 0.87342 | 0.71725 | 1.82259 | 0.00003 | 0.00326 |
| KEGG_CYTOKINE_CYTOKINE_RECEPTOR_INTERACTION | Immunity | -1.17846 | 0.13004 | 0.45118 | -1.69768 | 0.00045 | 0.00318 | 1.04191 | 0.39118 | 0.67294 |
| KEGG_LEUKOCYTE_TRANSENDOTHELIAL_MIGRATION | Immunity | 1.49831 | 0.00882 | 0.09953 | -2.11928 | 0.00004 | 0.00066 | 1.10900 | 0.28713 | 0.58733 |
| KEGG_SPHINGOLIPID_METABOLISM | Metabolism and energy | -0.63939 | 0.94873 | 0.88692 | 0.70294 | 0.88824 | 0.72077 | 0.53007 | 0.98809 | 0.87060 |
| KEGG_COMPLEMENT_AND_COAGULATION_CASCADES | Immunity | 1.44580 | 0.03231 | 0.22337 | -3.10040 | 0.00002 | 0.00050 | 0.91569 | 0.63058 | 0.80028 |
| KEGG_FOCAL_ADHESION | ECM and metastasis | 2.52497 | 0.00001 | 0.00041 | -2.53866 | 0.00017 | 0.00155 | 1.61667 | 0.00003 | 0.00307 |
| KEGG_T_CELL_RECEPTOR_SIGNALING_PATHWAY | Immunity | -1.42403 | 0.02390 | 0.18397 | -1.15083 | 0.13940 | 0.23945 | -1.73438 | 0.00037 | 0.01238 |
| KEGG_B_CELL_RECEPTOR_SIGNALING_PATHWAY | Immunity | -0.91520 | 0.61693 | 0.81658 | -1.97552 | 0.00008 | 0.00095 | -1.75312 | 0.00150 | 0.03080 |
| KEGG_ECM_RECEPTOR_INTERACTION | ECM and metastasis | 2.73898 | 0.00000 | 0.00041 | -2.58846 | 0.00002 | 0.00056 | 1.80411 | 0.00004 | 0.00395 |
| KEGG_TRYPTOPHAN_METABOLISM | Metabolism and energy | -1.32563 | 0.10639 | 0.41217 | 0.93403 | 0.58948 | 0.60208 | -1.13884 | 0.23622 | 0.53362 |
| KEGG_JAK_STAT_SIGNALING_PATHWAY | Immunity | -1.21197 | 0.12844 | 0.44835 | 0.88255 | 0.74682 | 0.67285 | 0.87981 | 0.73958 | 0.83863 |
| KEGG_BETA_ALANINE_METABOLISM | Metabolism and energy | -1.21816 | 0.21330 | 0.57217 | 0.78068 | 0.78142 | 0.68692 | 0.79276 | 0.76311 | 0.84720 |
| KEGG_NITROGEN_METABOLISM | Metabolism and energy | -1.60814 | 0.01701 | 0.14863 | 1.33132 | 0.11232 | 0.20602 | -0.96686 | 0.48892 | 0.73125 |
| KEGG_NATURAL_KILLER_CELL_MEDIATED_CYTOTOXICITY | Immunity | -1.35785 | 0.04159 | 0.25336 | -1.12815 | 0.16436 | 0.26915 | -1.01503 | 0.41572 | 0.69168 |
| KEGG_APOPTOSIS | Cell death | -0.62272 | 0.98821 | 0.88692 | -2.10542 | 0.00002 | 0.00057 | -1.27619 | 0.06978 | 0.30113 |
| KEGG_TOLL_LIKE_RECEPTOR_SIGNALING_PATHWAY | Immunity | -1.03921 | 0.39210 | 0.71075 | -1.34304 | 0.02017 | 0.05883 | -1.48829 | 0.00888 | 0.09542 |
| KEGG_ANTIGEN_PROCESSING_AND_PRESENTATION | Immunity | -0.63701 | 0.96511 | 0.88692 | 0.76070 | 0.84529 | 0.70850 | -0.92935 | 0.59113 | 0.78506 |
| KEGG_VALINE_LEUCINE_AND_ISOLEUCINE_DEGRADATION | Metabolism and energy | -1.20920 | 0.19520 | 0.55089 | 0.53375 | 0.98841 | 0.72077 | -0.79853 | 0.82512 | 0.86865 |
| KEGG_GAP_JUNCTION | ECM and metastasis | 1.58241 | 0.00684 | 0.08424 | 1.01797 | 0.46125 | 0.52459 | 0.97256 | 0.53942 | 0.75874 |
| KEGG_FC_GAMMA_R_MEDIATED_PHAGOCYTOSIS | Immunity | 0.94431 | 0.57203 | 0.79815 | -1.58321 | 0.00165 | 0.00852 | -1.24217 | 0.08827 | 0.33878 |
| KEGG_NOD_LIKE_RECEPTOR_SIGNALING_PATHWAY | Immunity | -0.95915 | 0.53286 | 0.78542 | -1.82269 | 0.00040 | 0.00292 | -2.08371 | 0.00010 | 0.00552 |
| KEGG_ALANINE_ASPARTATE_AND_GLUTAMATE_METABOLISM | Metabolism and energy | -1.05550 | 0.39212 | 0.71075 | 0.99995 | 0.48678 | 0.54018 | 0.60620 | 0.95113 | 0.87060 |
| KEGG_PURINE_METABOLISM | Genetic and epigenetic information | -0.76544 | 0.92209 | 0.88692 | 0.88346 | 0.74640 | 0.67281 | 0.56687 | 0.99814 | 0.87060 |
| KEGG_GALACTOSE_METABOLISM | Metabolism and energy | 1.02516 | 0.41307 | 0.72422 | -1.29294 | 0.12227 | 0.21839 | -1.14782 | 0.24681 | 0.54407 |
| KEGG_ADHERENS_JUNCTION | ECM and metastasis | 1.72539 | 0.00231 | 0.04001 | -1.76642 | 0.00030 | 0.00232 | 0.70633 | 0.91798 | 0.87060 |
| KEGG_HISTIDINE_METABOLISM | Metabolism and energy | -1.40330 | 0.07339 | 0.34747 | -0.86480 | 0.69504 | 0.65077 | -0.97249 | 0.48519 | 0.72935 |
| KEGG_BIOSYNTHESIS_OF_UNSATURATED_FATTY_ACIDS | Metabolism and energy | -0.74543 | 0.81615 | 0.88529 | 0.82964 | 0.71907 | 0.66163 | -0.94255 | 0.52894 | 0.75322 |
| KEGG_FATTY_ACID_METABOLISM | Metabolism and energy | -1.31735 | 0.10766 | 0.41281 | -1.17775 | 0.18219 | 0.28758 | 0.78734 | 0.79802 | 0.85918 |
| KEGG_STARCH_AND_SUCROSE_METABOLISM | Metabolism and energy | 1.54252 | 0.02546 | 0.19267 | -1.32152 | 0.07253 | 0.14947 | 0.57086 | 0.97919 | 0.87060 |
| KEGG_RIBOSOME | Genetic and epigenetic information | NA | NA | NA | NA | NA | NA | NA | NA | NA |
| KEGG_GLYCINE_SERINE_AND_THREONINE_METABOLISM | Metabolism and energy | -1.29160 | 0.13924 | 0.46525 | 1.07538 | 0.37794 | 0.46677 | -1.62870 | 0.02190 | 0.15782 |
| KEGG_TIGHT_JUNCTION | ECM and metastasis | 1.52280 | 0.00519 | 0.06892 | -1.07955 | 0.24504 | 0.35272 | 1.48453 | 0.00416 | 0.06038 |
| KEGG_ARGININE_AND_PROLINE_METABOLISM | Metabolism and energy | 1.19431 | 0.18695 | 0.54003 | -1.28394 | 0.08207 | 0.16375 | -1.03936 | 0.36897 | 0.65670 |
| KEGG_CITRATE_CYCLE_TCA_CYCLE | Metabolism and energy | -0.87557 | 0.64984 | 0.83016 | 0.39345 | 0.99928 | 0.72077 | -1.19491 | 0.19389 | 0.48935 |
| GOBP_REGULATION_OF_MITOCHONDRIAL_MEMBRANE_PERMEABILITY_INVOLVED_IN_APOPTOTIC_PROCESS | Cell death | -0.71482 | 0.89132 | 0.88692 | 0.65387 | 0.93432 | 0.72077 | -0.91943 | 0.59909 | 0.78624 |
| GOBP_SPLICEOSOMAL_SNRNP_ASSEMBLY | Genetic and epigenetic information | 0.64088 | 0.95411 | 0.88692 | 1.10118 | 0.33864 | 0.43609 | -0.48518 | 0.99878 | 0.87060 |
| GOBP_REGULATION_OF_MITOCHONDRIAL_OUTER_MEMBRANE_PERMEABILIZATION_INVOLVED_IN_APOPTOTIC_SIGNALING_PATHWAY | Cell death | -0.74846 | 0.82053 | 0.88645 | 0.64781 | 0.91882 | 0.72077 | -0.71662 | 0.88400 | 0.87060 |
| GOBP_APOPTOTIC_MITOCHONDRIAL_CHANGES | Cell death | 1.42034 | 0.02388 | 0.18397 | -1.30926 | 0.02978 | 0.07875 | -1.16437 | 0.14935 | 0.43518 |
| GOBP_MITOCHONDRIAL_OUTER_MEMBRANE_PERMEABILIZATION_INVOLVED_IN_PROGRAMMED_CELL_DEATH | Cell death | -0.67644 | 0.92353 | 0.88692 | 0.73833 | 0.85526 | 0.71197 | -1.03307 | 0.38130 | 0.66718 |
| GOBP_CHROMOSOME_CONDENSATION | Cell cycle | 1.25872 | 0.15491 | 0.48742 | 1.21604 | 0.19614 | 0.30220 | -1.15787 | 0.21902 | 0.51812 |
| GOBP_NUCLEOLAR_LARGE_RRNA_TRANSCRIPTION_BY_RNA_POLYMERASE_I | Genetic and epigenetic information | 0.88892 | 0.59711 | 0.80738 | 0.98891 | 0.50115 | 0.54973 | -0.64755 | 0.92740 | 0.87060 |
| GOBP_BASE_EXCISION_REPAIR | Genetic and epigenetic information | 0.67436 | 0.93830 | 0.88692 | 1.10707 | 0.32739 | 0.42632 | -0.77599 | 0.85235 | 0.87060 |
| GOBP_CELLULAR_COMPONENT_DISASSEMBLY_INVOLVED_IN_EXECUTION_PHASE_OF_APOPTOSIS | Cell death | -0.97617 | 0.50184 | 0.77190 | 0.96961 | 0.52990 | 0.56692 | -0.91128 | 0.59001 | 0.78399 |
| GOBP_UBIQUITIN_DEPENDENT_PROTEIN_CATABOLIC_PROCESS_VIA_THE_MULTIVESICULAR_BODY_SORTING_PATHWAY | Immunity | -1.06951 | 0.36689 | 0.69657 | -1.01113 | 0.43294 | 0.50876 | -1.85169 | 0.00404 | 0.05951 |
| GOBP_POSITIVE_REGULATION_OF_CELL_CYCLE_CHECKPOINT | Cell cycle | -0.89164 | 0.61898 | 0.81760 | 1.55937 | 0.01869 | 0.05556 | -1.60039 | 0.03493 | 0.20636 |
| GOBP_NUCLEAR_ENVELOPE_ORGANIZATION | Cell cycle | -0.61676 | 0.97832 | 0.88692 | 1.02788 | 0.44411 | 0.51566 | -1.33614 | 0.06606 | 0.29159 |
| GOBP_REGULATION_OF_MITOTIC_SPINDLE_ASSEMBLY | Cell cycle | -1.01736 | 0.44712 | 0.74495 | 1.01335 | 0.46752 | 0.52934 | -2.18244 | 0.00053 | 0.01574 |
| GOBP_NEGATIVE_REGULATION_OF_DNA_TEMPLATED_TRANSCRIPTION_ELONGATION | Genetic and epigenetic information | -0.77747 | 0.77642 | 0.87259 | 0.87777 | 0.65502 | 0.63401 | -1.00131 | 0.43568 | 0.70167 |
| GOBP_REGULATION_OF_SPINDLE_ORGANIZATION | Cell cycle | -0.81717 | 0.75960 | 0.86659 | 1.59055 | 0.00576 | 0.02253 | -1.65164 | 0.01131 | 0.10899 |
| GOBP_RNA_5_END_PROCESSING | Genetic and epigenetic information | 0.54508 | 0.97348 | 0.88692 | -1.23391 | 0.17388 | 0.27934 | -1.08247 | 0.32653 | 0.61917 |
| GOBP_NUCLEAR_MEMBRANE_ORGANIZATION | Cell cycle | -0.72673 | 0.87680 | 0.88692 | 0.79227 | 0.79446 | 0.69063 | -1.40724 | 0.05640 | 0.26871 |
| GOBP_RNA_SPLICING | Genetic and epigenetic information | -0.81505 | 0.93216 | 0.88692 | 1.12732 | 0.13113 | 0.22906 | -1.28346 | 0.00199 | 0.03757 |
| GOBP_NUCLEAR_MEMBRANE_REASSEMBLY | Cell cycle | -0.85162 | 0.68002 | 0.84067 | -0.83236 | 0.73718 | 0.66863 | -1.80622 | 0.00966 | 0.10050 |
| GOBP_RNA_SPLICING_VIA_TRANSESTERIFICATION_REACTIONS | Genetic and epigenetic information | -0.83972 | 0.86062 | 0.88692 | 1.26442 | 0.02167 | 0.06210 | -1.35421 | 0.00163 | 0.03218 |
| GOBP_MITOTIC_CHROMOSOME_CONDENSATION | Cell cycle | 0.76057 | 0.77879 | 0.87366 | 1.53905 | 0.02316 | 0.06533 | -1.33390 | 0.12020 | 0.39204 |
| GOBP_MRNA_PROCESSING | Genetic and epigenetic information | -0.81546 | 0.93678 | 0.88692 | 1.19020 | 0.04011 | 0.09725 | -1.24121 | 0.00275 | 0.04530 |
| GOBP_TRANSCRIPTION_ELONGATION_BY_RNA_POLYMERASE_II_PROMOTER | Genetic and epigenetic information | -0.71805 | 0.94668 | 0.88692 | 0.86088 | 0.75692 | 0.67713 | -0.67107 | 0.99484 | 0.87060 |
| GOBP_MATURATION_OF_5_8S_RRNA | Genetic and epigenetic information | -0.56728 | 0.97923 | 0.88692 | 0.95620 | 0.55298 | 0.58125 | -0.95497 | 0.52011 | 0.74743 |
| GOBP_NEGATIVE_REGULATION_OF_RNA_SPLICING | Genetic and epigenetic information | 0.59574 | 0.95763 | 0.88692 | 1.24529 | 0.18008 | 0.28609 | -0.46686 | 0.99798 | 0.87060 |
| GOBP_MATURATION_OF_5_8S_RRNA_FROM_TRICISTRONIC_RRNA_TRANSCRIPT_SSU_RRNA_5_8S_RRNA_LSU_RRNA | Genetic and epigenetic information | -0.64211 | 0.92511 | 0.88692 | 0.98173 | 0.51219 | 0.55640 | -1.01526 | 0.41590 | 0.69168 |
| GOBP_NEGATIVE_REGULATION_OF_MRNA_PROCESSING | Genetic and epigenetic information | 0.56600 | 0.97008 | 0.88692 | 1.33608 | 0.10610 | 0.19800 | 0.49182 | 0.98954 | 0.87060 |
| GOBP_NCRNA_PROCESSING | Genetic and epigenetic information | -0.50149 | 1.00000 | 0.88692 | 0.75301 | 0.97960 | 0.72077 | -1.25873 | 0.00400 | 0.05908 |
| GOBP_MRNA_EXPORT_FROM_NUCLEUS | Genetic and epigenetic information | -0.53423 | 0.99644 | 0.88692 | 1.32250 | 0.07595 | 0.15472 | -0.76219 | 0.90887 | 0.87060 |
| GOBP_RRNA_METABOLIC_PROCESS | Genetic and epigenetic information | 0.68595 | 0.99921 | 0.88692 | 0.74469 | 0.95583 | 0.72077 | -1.17446 | 0.07065 | 0.30304 |
| GOBP_RIBONUCLEOPROTEIN_COMPLEX_SUBUNIT_ORGANIZATION | Genetic and epigenetic information | -0.59879 | 0.99936 | 0.88692 | 1.10587 | 0.26236 | 0.36908 | -1.63917 | 0.00030 | 0.01075 |
| GOBP_REGULATION_OF_CARBOHYDRATE_CATABOLIC_PROCESS | Metabolism and energy | 0.84458 | 0.74104 | 0.86073 | 0.90162 | 0.65049 | 0.63165 | 0.64913 | 0.94794 | 0.87060 |
| GOBP_RRNA_MODIFICATION | Genetic and epigenetic information | -0.57029 | 0.97831 | 0.88692 | 0.81762 | 0.75160 | 0.67436 | -0.60331 | 0.98016 | 0.87060 |
| GOBP_MITOTIC_METAPHASE_PLATE_CONGRESSION | Cell cycle | -0.49840 | 0.99833 | 0.88692 | 1.59440 | 0.00359 | 0.01559 | -1.52018 | 0.01890 | 0.14382 |
| GOBP_RIBONUCLEOPROTEIN_COMPLEX_BIOGENESIS | Genetic and epigenetic information | -0.39395 | 1.00000 | 0.88692 | 0.95026 | 0.66721 | 0.63981 | -1.62578 | 0.00048 | 0.01452 |
| GOBP_MITOTIC_SPINDLE_ORGANIZATION | Cell cycle | 0.67755 | 0.99399 | 0.88692 | 1.73381 | 0.00001 | 0.00035 | -0.88949 | 0.77500 | 0.85306 |
| GOBP_SPLICEOSOMAL_COMPLEX_ASSEMBLY | Genetic and epigenetic information | -0.95665 | 0.53999 | 0.78838 | 1.28657 | 0.09229 | 0.17949 | -1.64480 | 0.00453 | 0.06280 |
| GOBP_POSITIVE_REGULATION_OF_TELOMERE_CAPPING | Cell cycle | -0.77883 | 0.76635 | 0.86907 | 1.13401 | 0.31237 | 0.41424 | -0.77350 | 0.77903 | 0.85492 |
| GOBP_REGULATION_OF_TRANSCRIPTION_BY_RNA_POLYMERASE_I | Genetic and epigenetic information | 0.74971 | 0.85715 | 0.88692 | 0.36702 | 0.99987 | 0.72077 | -1.02454 | 0.39573 | 0.67711 |
| GOBP_REGULATION_OF_NCRNA_TRANSCRIPTION | Genetic and epigenetic information | 0.63407 | 0.92243 | 0.88692 | 1.15988 | 0.27673 | 0.38241 | -1.13663 | 0.26582 | 0.56699 |
| GOBP_CLEAVAGE_INVOLVED_IN_RRNA_PROCESSING | Genetic and epigenetic information | -0.79679 | 0.76216 | 0.86736 | 1.00730 | 0.47608 | 0.53424 | -1.21342 | 0.17971 | 0.47341 |
| GOBP_NEGATIVE_REGULATION_OF_MRNA_METABOLIC_PROCESS | Genetic and epigenetic information | -0.74176 | 0.90355 | 0.88692 | 1.17022 | 0.20988 | 0.31675 | -0.93733 | 0.60041 | 0.78671 |
| GOBP_REGULATION_OF_CENTROSOME_CYCLE | Cell cycle | -0.68939 | 0.92840 | 0.88692 | 1.61221 | 0.00338 | 0.01483 | -0.74189 | 0.91972 | 0.87060 |
| GOBP_RIBOSOMAL_LARGE_SUBUNIT_ASSEMBLY | Genetic and epigenetic information | 0.87608 | 0.60836 | 0.81315 | 1.03510 | 0.43961 | 0.51299 | -1.57312 | 0.04480 | 0.23682 |
| GOBP_MATURATION_OF_SSU_RRNA_FROM_TRICISTRONIC_RRNA_TRANSCRIPT_SSU_RRNA_5_8S_RRNA_LSU_RRNA | Genetic and epigenetic information | 0.72194 | 0.86632 | 0.88692 | 0.77334 | 0.80023 | 0.69338 | -1.25287 | 0.14701 | 0.43199 |
| GOBP_REGULATION_OF_SPINDLE_ASSEMBLY | Cell cycle | -1.16225 | 0.25815 | 0.61103 | 1.43774 | 0.04361 | 0.10388 | -1.86086 | 0.00484 | 0.06500 |
| GOBP_U2_TYPE_PRESPLICEOSOME_ASSEMBLY | Genetic and epigenetic information | 0.83152 | 0.69824 | 0.84737 | 1.09141 | 0.35970 | 0.45269 | -1.24352 | 0.16315 | 0.45204 |
| GOBP_RIBOSOME_BIOGENESIS | Genetic and epigenetic information | 0.61538 | 0.99999 | 0.88692 | 0.80396 | 0.91314 | 0.72077 | -1.43084 | 0.00104 | 0.02472 |
| GOBP_MITOTIC_NUCLEAR_DIVISION | Cell cycle | -0.66561 | 0.99918 | 0.88692 | 1.63664 | 0.00000 | 0.00009 | -1.20260 | 0.02422 | 0.16830 |
| GOBP_METAPHASE_PLATE_CONGRESSION | Cell cycle | -0.43318 | 0.99993 | 0.88692 | 1.66076 | 0.00074 | 0.00465 | -1.26729 | 0.08832 | 0.33878 |
| GOBP_APOPTOTIC_PROCESS_INVOLVED_IN_MORPHOGENESIS | Cell death | 1.13706 | 0.27884 | 0.62560 | 0.67120 | 0.90118 | 0.72077 | 1.70088 | 0.00340 | 0.05223 |
| GOBP_ANOIKIS | Cell death | 1.20916 | 0.19817 | 0.55407 | -1.12286 | 0.26003 | 0.36659 | -0.95128 | 0.52570 | 0.75201 |
| GOBP_ANAPHASE_PROMOTING_COMPLEX_DEPENDENT_CATABOLIC_PROCESS | Cell cycle | -0.88037 | 0.63786 | 0.82706 | 1.12751 | 0.31454 | 0.41627 | -1.31831 | 0.11994 | 0.39204 |
| GOBP_POSITIVE_REGULATION_OF_MRNA_SPLICING_VIA_SPLICEOSOME | Genetic and epigenetic information | 0.62159 | 0.94463 | 0.88692 | 1.12047 | 0.32126 | 0.42148 | -0.93780 | 0.54205 | 0.76069 |
| GOBP_MRNA_CIS_SPLICING_VIA_SPLICEOSOME | Genetic and epigenetic information | -1.06707 | 0.38120 | 0.70361 | 1.10128 | 0.34769 | 0.44265 | -1.10472 | 0.29641 | 0.59560 |
| GOBP_MITOTIC_SPINDLE_ASSEMBLY | Cell cycle | -0.45015 | 0.99987 | 0.88692 | 1.61712 | 0.00157 | 0.00819 | -1.14349 | 0.19852 | 0.49197 |
| GOBP_POSITIVE_REGULATION_OF_UBIQUITIN_PROTEIN_TRANSFERASE_ACTIVITY | Immunity | 0.52863 | 0.98657 | 0.88692 | 0.83810 | 0.71526 | 0.66059 | -0.88885 | 0.63153 | 0.80088 |
| GOBP_TRANSCRIPTION_BY_RNA_POLYMERASE_I | Genetic and epigenetic information | 0.78147 | 0.84372 | 0.88692 | 0.39971 | 0.99981 | 0.72077 | -0.86233 | 0.73071 | 0.83705 |
| GOBP_RRNA_TRANSCRIPTION | Genetic and epigenetic information | 0.86284 | 0.67064 | 0.83659 | -0.58807 | 0.98761 | 0.72077 | -0.97610 | 0.48038 | 0.72656 |
| GOBP_MICROTUBULE_CYTOSKELETON_ORGANIZATION_INVOLVED_IN_MITOSIS | Cell cycle | 0.71142 | 0.99199 | 0.88692 | 1.66588 | 0.00001 | 0.00040 | -0.92001 | 0.71973 | 0.83336 |
| GOBP_REGULATION_OF_TRANSCRIPTION_ELONGATION_BY_RNA_POLYMERASE_II | Genetic and epigenetic information | -0.76986 | 0.87381 | 0.88692 | 0.77834 | 0.85499 | 0.71197 | -0.78292 | 0.91642 | 0.87060 |
| GOBP_DNA_TEMPLATED_TRANSCRIPTION_ELONGATION | Genetic and epigenetic information | -0.82514 | 0.87077 | 0.88692 | 0.92668 | 0.69443 | 0.65038 | -0.87174 | 0.89506 | 0.87060 |
| GOBP_SPINDLE_ORGANIZATION | Cell cycle | -0.60048 | 0.99958 | 0.88692 | 1.68568 | 0.00000 | 0.00021 | -0.96920 | 0.57100 | 0.77299 |
| GOBP_RNA_EXPORT_FROM_NUCLEUS | Genetic and epigenetic information | -0.53607 | 0.99830 | 0.88692 | 1.37962 | 0.03431 | 0.08690 | -0.79567 | 0.89226 | 0.87060 |
| GOBP_POSITIVE_REGULATION_OF_PROTEOLYSIS_INVOLVED_IN_PROTEIN_CATABOLIC_PROCESS | Metabolism and energy | -1.09185 | 0.29388 | 0.64013 | -0.83452 | 0.91251 | 0.72077 | -0.98336 | 0.50813 | 0.74135 |
| GOBP_REGULATION_OF_DNA_REPAIR | Genetic and epigenetic information | 0.68245 | 0.99897 | 0.88692 | 1.11599 | 0.22745 | 0.33533 | -0.55598 | 1.00000 | 0.87060 |
| GOBP_REGULATION_OF_MRNA_PROCESSING | Genetic and epigenetic information | -0.81657 | 0.83729 | 0.88692 | 1.11662 | 0.26078 | 0.36736 | -1.24114 | 0.06127 | 0.27999 |
| GOBP_REGULATION_OF_DNA_TEMPLATED_TRANSCRIPTION_ELONGATION | Genetic and epigenetic information | -0.75584 | 0.90660 | 0.88692 | 0.92751 | 0.63912 | 0.62626 | -0.81983 | 0.88645 | 0.87060 |
| GOBP_HISTONE_H3_K14_ACETYLATION | Genetic and epigenetic information | -1.09512 | 0.34849 | 0.68236 | 1.12517 | 0.31839 | 0.41889 | 0.77264 | 0.78654 | 0.85642 |
| GOBP_DNA_TEMPLATED_TRANSCRIPTION_INITIATION | Genetic and epigenetic information | 0.65614 | 0.99626 | 0.88692 | 0.87542 | 0.74677 | 0.67285 | 0.61307 | 0.98997 | 0.87060 |
| GOBP_MRNA_TRANSPORT | Genetic and epigenetic information | -0.52587 | 0.99976 | 0.88692 | 1.10219 | 0.29554 | 0.39983 | -1.12873 | 0.18305 | 0.47756 |
| GOBP_TELOMERE_MAINTENANCE | Cell cycle | 0.66936 | 0.99674 | 0.88692 | 1.11881 | 0.25178 | 0.35890 | -0.81367 | 0.93084 | 0.87060 |
| GOBP_EXIT_FROM_MITOSIS | Cell cycle | -1.06524 | 0.37961 | 0.70211 | 1.11853 | 0.32203 | 0.42185 | -0.71599 | 0.89120 | 0.87060 |
| GOBP_TELOMERE_MAINTENANCE_VIA_TELOMERE_LENGTHENING | Cell cycle | 0.78087 | 0.88024 | 0.88692 | 0.99191 | 0.50801 | 0.55350 | -0.71398 | 0.96598 | 0.87060 |
| GOBP_TELOMERE_ORGANIZATION | Cell cycle | 0.65381 | 0.99822 | 0.88692 | 1.16156 | 0.17751 | 0.28270 | -0.87323 | 0.82809 | 0.86979 |
| GOBP_TRNA_PROCESSING | Genetic and epigenetic information | -0.94608 | 0.57458 | 0.79849 | 0.94689 | 0.61097 | 0.61184 | -1.15082 | 0.14441 | 0.42961 |
| GOBP_COENZYME_A_METABOLIC_PROCESS | Metabolism and energy | -0.78047 | 0.76755 | 0.86966 | 0.84768 | 0.69274 | 0.64968 | 1.26680 | 0.17043 | 0.46186 |
| GOBP_REGULATION_OF_DOUBLE_STRAND_BREAK_REPAIR | Genetic and epigenetic information | -0.64430 | 0.99194 | 0.88692 | 1.23221 | 0.09983 | 0.18945 | -0.66481 | 0.99740 | 0.87060 |
| GOBP_POSITIVE_REGULATION_OF_RESPONSE_TO_DNA_DAMAGE_STIMULUS | Genetic and epigenetic information | -0.61215 | 0.99840 | 0.88692 | 1.07939 | 0.32280 | 0.42253 | -1.01052 | 0.42735 | 0.69818 |
| GOBP_RNA_MODIFICATION | Genetic and epigenetic information | -0.69427 | 0.98392 | 0.88692 | 0.93770 | 0.64566 | 0.62986 | -0.84005 | 0.90918 | 0.87060 |
| GOBP_RNA_3_END_PROCESSING | Genetic and epigenetic information | -1.01298 | 0.43970 | 0.74070 | 1.18157 | 0.18451 | 0.28994 | -0.93355 | 0.62189 | 0.79729 |
| GOBP_MITOCHONDRIAL_RNA_PROCESSING | Genetic and epigenetic information | -0.62362 | 0.92770 | 0.88692 | 0.86110 | 0.67503 | 0.64207 | -1.22915 | 0.18736 | 0.48255 |
| GOBP_POSITIVE_REGULATION_OF_PROTEASOMAL_UBIQUITIN_DEPENDENT_PROTEIN_CATABOLIC_PROCESS | Immunity | -1.07216 | 0.33852 | 0.67277 | -0.74469 | 0.97030 | 0.72077 | -0.85179 | 0.81649 | 0.86664 |
| GOBP_G0_TO_G1_TRANSITION | Cell cycle | -0.85118 | 0.69636 | 0.84639 | 1.07742 | 0.37222 | 0.46236 | -1.26164 | 0.13216 | 0.41088 |
| GOBP_MATURATION_OF_SSU_RRNA | Genetic and epigenetic information | 0.61853 | 0.97568 | 0.88692 | 0.62712 | 0.95266 | 0.72077 | -1.43256 | 0.04671 | 0.24279 |
| GOBP_TELOMERE_MAINTENANCE_IN_RESPONSE_TO_DNA_DAMAGE | Genetic and epigenetic information | 0.46941 | 0.99577 | 0.88692 | 0.93232 | 0.58362 | 0.59845 | -0.86590 | 0.66871 | 0.81530 |
| GOBP_POSITIVE_REGULATION_OF_CHROMOSOME_SEGREGATION | Cell cycle | -0.60928 | 0.94917 | 0.88692 | 1.61996 | 0.00822 | 0.02977 | -1.50106 | 0.04799 | 0.24617 |
| GOBP_POSITIVE_REGULATION_OF_TELOMERE_MAINTENANCE | Cell cycle | 0.94218 | 0.55757 | 0.79537 | 0.84567 | 0.74272 | 0.67141 | -1.42131 | 0.03529 | 0.20752 |
| GOBP_ATP_METABOLIC_PROCESS | Metabolism and energy | 1.43832 | 0.00668 | 0.08304 | -1.23141 | 0.02872 | 0.07641 | -1.13075 | 0.13769 | 0.41877 |
| GOBP_TRANSCRIPTION_PREINITIATION_COMPLEX_ASSEMBLY | Genetic and epigenetic information | 0.56980 | 0.99669 | 0.88692 | 0.84283 | 0.75077 | 0.67432 | 0.45587 | 0.99937 | 0.87060 |
| GOBP_SPINDLE_ASSEMBLY | Cell cycle | -0.71620 | 0.95776 | 0.88692 | 1.70010 | 0.00003 | 0.00061 | -1.05859 | 0.30656 | 0.60517 |
| GOBP_SNRNA_PROCESSING | Genetic and epigenetic information | -0.66134 | 0.91556 | 0.88692 | 1.01268 | 0.46839 | 0.52934 | 0.60685 | 0.94964 | 0.87060 |
| GOBP_MRNA_CATABOLIC_PROCESS | Genetic and epigenetic information | -0.85934 | 0.80044 | 0.87900 | 0.87612 | 0.79791 | 0.69220 | -1.18129 | 0.06275 | 0.28261 |
| GOBP_TRANSCRIPTION_INITIATION_AT_RNA_POLYMERASE_II_PROMOTER | Genetic and epigenetic information | 0.60879 | 0.99672 | 0.88692 | 0.93986 | 0.60918 | 0.61110 | 0.53133 | 0.99755 | 0.87060 |
| GOBP_RIBOSOME_ASSEMBLY | Genetic and epigenetic information | 1.09092 | 0.32138 | 0.66146 | 0.89317 | 0.64570 | 0.62986 | -1.56901 | 0.02776 | 0.18047 |
| GOBP_MITOTIC_CYTOKINETIC_PROCESS | Cell cycle | 0.53470 | 0.98120 | 0.88692 | -0.66307 | 0.93665 | 0.72077 | -2.05877 | 0.00158 | 0.03158 |
| GOBP_TRNA_METABOLIC_PROCESS | Genetic and epigenetic information | -0.95840 | 0.55813 | 0.79537 | 0.98482 | 0.53858 | 0.57300 | -1.03474 | 0.35568 | 0.64257 |
| GOBP_HISTONE_DEACETYLATION | Genetic and epigenetic information | -0.98833 | 0.48535 | 0.76444 | 0.54442 | 0.99642 | 0.72077 | 0.39632 | 0.99999 | 0.87060 |
| GOBP_REGULATION_OF_TELOMERE_MAINTENANCE | Cell cycle | 0.62931 | 0.99521 | 0.88692 | 0.98413 | 0.52684 | 0.56545 | -1.03975 | 0.35896 | 0.64622 |
| GOBP_RNA_PHOSPHODIESTER_BOND_HYDROLYSIS | Genetic and epigenetic information | -0.87994 | 0.72356 | 0.85522 | 0.51860 | 0.99950 | 0.72077 | -1.00987 | 0.43307 | 0.70113 |
| GOBP_SISTER_CHROMATID_SEGREGATION | Cell cycle | -0.62641 | 0.99943 | 0.88692 | 1.77410 | 0.00000 | 0.00009 | -1.39925 | 0.00254 | 0.04367 |
| GOBP_REGULATION_OF_RESPONSE_TO_DNA_DAMAGE_STIMULUS | Genetic and epigenetic information | 0.90220 | 0.81432 | 0.88529 | 0.97942 | 0.56734 | 0.58951 | -0.79420 | 0.99354 | 0.87060 |
| GOBP_NUCLEAR_CHROMOSOME_SEGREGATION | Cell cycle | -0.81192 | 0.91571 | 0.88692 | 1.76143 | 0.00000 | 0.00009 | -1.15752 | 0.05449 | 0.26452 |
| GOBP_MATURATION_OF_LSU_RRNA | Genetic and epigenetic information | 1.33364 | 0.13345 | 0.45637 | -1.34377 | 0.10202 | 0.19255 | -1.33158 | 0.11628 | 0.38614 |
| GOBP_CHROMOSOME_SEGREGATION | Cell cycle | -0.78061 | 0.96430 | 0.88692 | 1.71311 | 0.00000 | 0.00009 | -1.12359 | 0.07915 | 0.31926 |
| GOBP_REGULATION_OF_RNA_SPLICING | Genetic and epigenetic information | -0.91791 | 0.64925 | 0.83016 | 1.04719 | 0.39138 | 0.47664 | -0.96278 | 0.58378 | 0.77979 |
| GOBP_POSITIVE_REGULATION_OF_MRNA_PROCESSING | Genetic and epigenetic information | -0.71740 | 0.87435 | 0.88692 | 0.96190 | 0.54436 | 0.57630 | -1.11975 | 0.26260 | 0.56379 |
| GOBP_NUCLEAR_TRANSCRIBED_MRNA_CATABOLIC_PROCESS_NONSENSE_MEDIATED_DECAY | Genetic and epigenetic information | 0.47358 | 0.99810 | 0.88692 | 1.04823 | 0.41447 | 0.49541 | 0.58332 | 0.97177 | 0.87060 |
| GOBP_REGULATION_OF_ATP_METABOLIC_PROCESS | Metabolism and energy | 0.89384 | 0.66021 | 0.83351 | -1.47407 | 0.01255 | 0.04099 | -0.66878 | 0.98043 | 0.87060 |
| GOBP_POSITIVE_REGULATION_OF_UBIQUITIN_DEPENDENT_PROTEIN_CATABOLIC_PROCESS | Immunity | -1.15028 | 0.21750 | 0.57661 | -0.69986 | 0.99299 | 0.72077 | -0.99580 | 0.46706 | 0.72126 |
| GOBP_REGULATION_OF_TUMOR_NECROSIS_FACTOR_MEDIATED_SIGNALING_PATHWAY | Immunity | 0.78684 | 0.83183 | 0.88692 | -1.05142 | 0.35226 | 0.44609 | -0.84998 | 0.75100 | 0.84353 |
| GOBP_RNA_PHOSPHODIESTER_BOND_HYDROLYSIS_ENDONUCLEOLYTIC | Genetic and epigenetic information | -0.53162 | 0.99840 | 0.88692 | 0.49627 | 0.99844 | 0.72077 | -0.77949 | 0.91076 | 0.87060 |
| GOBP_RNA_CATABOLIC_PROCESS | Genetic and epigenetic information | -0.70082 | 0.99403 | 0.88692 | 0.80682 | 0.91907 | 0.72077 | -1.16528 | 0.05824 | 0.27311 |
| GOBP_POSITIVE_REGULATION_OF_HISTONE_MODIFICATION | Genetic and epigenetic information | -0.86984 | 0.71830 | 0.85522 | 0.61696 | 0.98260 | 0.72077 | -1.06501 | 0.30427 | 0.60196 |
| GOBP_RIBOSOMAL_LARGE_SUBUNIT_BIOGENESIS | Genetic and epigenetic information | 0.97049 | 0.49958 | 0.77118 | -0.76587 | 0.90784 | 0.72077 | -1.72578 | 0.00439 | 0.06152 |
| GOBP_REGULATION_OF_PLATELET_DERIVED_GROWTH_FACTOR_RECEPTOR_SIGNALING_PATHWAY | ECM and metastasis | 1.88197 | 0.00349 | 0.05267 | -1.62713 | 0.01853 | 0.05523 | 0.79981 | 0.75573 | 0.84458 |
| GOBP_REGULATION_OF_CYCLIN_DEPENDENT_PROTEIN_KINASE_ACTIVITY | Cell cycle | -0.68913 | 0.96476 | 0.88692 | 1.41204 | 0.01642 | 0.05038 | 0.71705 | 0.92605 | 0.87060 |
| GOBP_NEGATIVE_REGULATION_OF_CARBOHYDRATE_METABOLIC_PROCESS | Metabolism and energy | 1.45427 | 0.04255 | 0.25608 | -1.00709 | 0.44313 | 0.51510 | -1.12834 | 0.23935 | 0.53849 |
| GOBP_MRNA_SPLICE_SITE_SELECTION | Genetic and epigenetic information | -1.48483 | 0.03915 | 0.24467 | 1.34660 | 0.08943 | 0.17506 | -1.35706 | 0.08726 | 0.33634 |
| GOBP_PROTEIN_QUALITY_CONTROL_FOR_MISFOLDED_OR_INCOMPLETELY_SYNTHESIZED_PROTEINS | Metabolism and energy | 0.62692 | 0.94773 | 0.88692 | 0.40883 | 0.99881 | 0.72077 | -1.13132 | 0.25695 | 0.55753 |
| GOBP_REGULATION_OF_NUCLEAR_DIVISION | Cell cycle | -0.87271 | 0.73584 | 0.86010 | 1.75804 | 0.00000 | 0.00015 | 0.66350 | 0.97621 | 0.87060 |
| GOBP_CENTROMERE_COMPLEX_ASSEMBLY | Cell cycle | 0.90023 | 0.60290 | 0.81139 | 1.75271 | 0.00114 | 0.00647 | 0.63228 | 0.93665 | 0.87060 |
| GOBP_RRNA_METHYLATION | Genetic and epigenetic information | -0.75493 | 0.80874 | 0.88302 | 0.91499 | 0.60583 | 0.60925 | 0.69691 | 0.87299 | 0.87060 |
| GOBP_ATP_BIOSYNTHETIC_PROCESS | Metabolism and energy | 0.63260 | 0.99237 | 0.88692 | -0.81776 | 0.88976 | 0.72077 | -1.65866 | 0.00246 | 0.04280 |
| GOBP_MITOCHONDRIAL_RNA_METABOLIC_PROCESS | Genetic and epigenetic information | -0.53381 | 0.99295 | 0.88692 | 0.91643 | 0.62085 | 0.61589 | -1.03487 | 0.37839 | 0.66437 |
| GOBP_MRNA_3_END_PROCESSING | Genetic and epigenetic information | -1.13908 | 0.26503 | 0.61685 | 1.02108 | 0.45595 | 0.52229 | -0.92055 | 0.60925 | 0.79175 |
| GOBP_REGULATION_OF_CHROMOSOME_ORGANIZATION | Cell cycle | -0.65990 | 0.99795 | 0.88692 | 1.49159 | 0.00017 | 0.00155 | -1.27880 | 0.01413 | 0.12491 |
| GOBP_DNA_REPLICATION | Genetic and epigenetic information | -0.56604 | 0.99999 | 0.88692 | 1.49183 | 0.00006 | 0.00077 | -0.89502 | 0.86988 | 0.87060 |
| GOBP_POSITIVE_REGULATION_OF_DNA_REPAIR | Genetic and epigenetic information | 0.68654 | 0.99084 | 0.88692 | 1.13771 | 0.23023 | 0.33807 | -0.82210 | 0.90222 | 0.87060 |
| GOBP_TRNA_MODIFICATION | Genetic and epigenetic information | -1.06665 | 0.34832 | 0.68236 | 1.06592 | 0.37181 | 0.46196 | -1.06945 | 0.30083 | 0.59988 |
| GOBP_NUCLEIC_ACID_PHOSPHODIESTER_BOND_HYDROLYSIS | Genetic and epigenetic information | -0.93969 | 0.61529 | 0.81629 | 0.77123 | 0.93965 | 0.72077 | -0.84623 | 0.93768 | 0.87060 |
| GOBP_REGULATION_OF_MITOTIC_NUCLEAR_DIVISION | Cell cycle | -0.91122 | 0.64457 | 0.82854 | 1.74996 | 0.00002 | 0.00049 | -0.76948 | 0.95764 | 0.87060 |
| GOBP_RNA_METHYLATION | Genetic and epigenetic information | -0.64014 | 0.98134 | 0.88692 | 1.13470 | 0.25890 | 0.36590 | 0.46679 | 0.99961 | 0.87060 |
| GOBP_HISTONE_H3_ACETYLATION | Genetic and epigenetic information | -1.03326 | 0.40592 | 0.72060 | 0.83716 | 0.77318 | 0.68387 | 0.45539 | 0.99970 | 0.87060 |
| GOBP_RIBOSOMAL_SMALL_SUBUNIT_BIOGENESIS | Genetic and epigenetic information | 0.65557 | 0.96812 | 0.88692 | 0.77982 | 0.82262 | 0.70045 | -1.34818 | 0.06595 | 0.29159 |
| GOBP_NEGATIVE_REGULATION_OF_TELOMERE_MAINTENANCE_VIA_TELOMERE_LENGTHENING | Cell cycle | -1.09543 | 0.34617 | 0.68050 | 1.22040 | 0.20642 | 0.31304 | 0.40896 | 0.99845 | 0.87060 |
| GOBP_REGULATION_OF_MRNA_METABOLIC_PROCESS | Genetic and epigenetic information | -0.90352 | 0.71686 | 0.85494 | 0.98405 | 0.55237 | 0.58113 | -1.24503 | 0.01717 | 0.13823 |
| GOBP_DNA_MODIFICATION | Genetic and epigenetic information | -0.78258 | 0.88345 | 0.88692 | 0.87463 | 0.74473 | 0.67226 | -1.02438 | 0.39108 | 0.67294 |
| GOBP_NEGATIVE_REGULATION_OF_RESPONSE_TO_DNA_DAMAGE_STIMULUS | Genetic and epigenetic information | 1.38441 | 0.04514 | 0.26611 | 0.78972 | 0.83289 | 0.70443 | 0.81977 | 0.78728 | 0.85650 |
| GOBP_NEGATIVE_REGULATION_OF_DNA_BIOSYNTHETIC_PROCESS | Genetic and epigenetic information | -0.91991 | 0.58740 | 0.80397 | 1.05650 | 0.40360 | 0.48635 | 1.25303 | 0.16288 | 0.45204 |
| GOBP_NUCLEOSIDE_TRIPHOSPHATE_BIOSYNTHETIC_PROCESS | Metabolism and energy | -0.72907 | 0.93884 | 0.88692 | -0.57238 | 1.00000 | 0.72077 | -1.38622 | 0.01999 | 0.14848 |
| GOBP_RNA_POLYADENYLATION | Genetic and epigenetic information | -0.98969 | 0.48041 | 0.76283 | 0.95764 | 0.55275 | 0.58113 | -0.93377 | 0.56358 | 0.76916 |
| GOBP_REGULATION_OF_MRNA_SPLICING_VIA_SPLICEOSOME | Genetic and epigenetic information | -0.61667 | 0.99285 | 0.88692 | 1.24051 | 0.10963 | 0.20303 | -1.03577 | 0.36603 | 0.65315 |
| GOBP_RNA_CAPPING | Genetic and epigenetic information | 1.03517 | 0.40254 | 0.71805 | 0.98043 | 0.51285 | 0.55665 | -0.97305 | 0.47723 | 0.72645 |
| GOBP_POSITIVE_REGULATION_OF_CHROMOSOME_ORGANIZATION | Cell cycle | 0.77882 | 0.91191 | 0.88692 | 1.15212 | 0.22345 | 0.33085 | -1.53584 | 0.00576 | 0.07277 |
| GOBP_CENTROSOME_DUPLICATION | Cell cycle | -0.53070 | 0.99816 | 0.88692 | 1.61726 | 0.00140 | 0.00751 | -0.72892 | 0.95610 | 0.87060 |
| GOBP_CHROMATIN_REMODELING | Cell cycle | -0.66820 | 0.99850 | 0.88692 | 1.14833 | 0.13468 | 0.23376 | -1.20858 | 0.03071 | 0.19211 |
| GOBP_REGULATION_OF_PROTEASOMAL_UBIQUITIN_DEPENDENT_PROTEIN_CATABOLIC_PROCESS | Immunity | -0.95186 | 0.56578 | 0.79753 | -0.56305 | 1.00000 | 0.72077 | -0.93116 | 0.66844 | 0.81522 |
| GOBP_DOUBLE_STRAND_BREAK_REPAIR | Genetic and epigenetic information | -0.51656 | 1.00000 | 0.88692 | 1.31140 | 0.00806 | 0.02940 | -0.74356 | 0.99916 | 0.87060 |
| GOBP_POSITIVE_REGULATION_OF_TRANSCRIPTION_BY_RNA_POLYMERASE_I | Genetic and epigenetic information | 0.90850 | 0.58943 | 0.80397 | 0.69237 | 0.88758 | 0.72077 | -0.76103 | 0.84606 | 0.87060 |
| GOBP_UBIQUINONE_METABOLIC_PROCESS | Metabolism and energy | -1.03762 | 0.42189 | 0.73011 | 1.01838 | 0.46103 | 0.52458 | 0.52912 | 0.97744 | 0.87060 |
| GOBP_NUCLEOSIDE_TRIPHOSPHATE_METABOLIC_PROCESS | Metabolism and energy | 1.28072 | 0.02959 | 0.21254 | -1.12352 | 0.11509 | 0.20948 | -1.12663 | 0.12482 | 0.39926 |
| GOBP_DNA_STRAND_ELONGATION | Genetic and epigenetic information | 0.48817 | 0.99712 | 0.88692 | 1.32919 | 0.09415 | 0.18200 | -1.07527 | 0.31767 | 0.61238 |
| GOBP_LINOLEIC_ACID_METABOLIC_PROCESS | Metabolism and energy | -0.72084 | 0.84339 | 0.88692 | -0.85249 | 0.68803 | 0.64753 | -0.67463 | 0.91083 | 0.87060 |
| GOBP_NEGATIVE_REGULATION_OF_RNA_CATABOLIC_PROCESS | Genetic and epigenetic information | -0.74008 | 0.89491 | 0.88692 | 0.91938 | 0.63289 | 0.62199 | -1.19199 | 0.14968 | 0.43518 |
| GOBP_REGULATION_OF_DOUBLE_STRAND_BREAK_REPAIR_VIA_NONHOMOLOGOUS_END_JOINING | Genetic and epigenetic information | 0.87127 | 0.64438 | 0.82854 | 0.79677 | 0.76837 | 0.68242 | 0.64796 | 0.92163 | 0.87060 |
| GOBP_DNA_TEMPLATED_DNA_REPLICATION | Genetic and epigenetic information | 0.59613 | 0.99991 | 0.88692 | 1.67604 | 0.00001 | 0.00040 | -0.80209 | 0.95784 | 0.87060 |
| GOBP_NEGATIVE_REGULATION_OF_PROTEASOMAL_UBIQUITIN_DEPENDENT_PROTEIN_CATABOLIC_PROCESS | Immunity | -0.77221 | 0.80761 | 0.88245 | 0.58357 | 0.96732 | 0.72077 | -1.17376 | 0.20504 | 0.50150 |
| GOBP_MONOUBIQUITINATED_PROTEIN_DEUBIQUITINATION | Immunity | -1.12907 | 0.29277 | 0.63925 | 0.94422 | 0.57098 | 0.59128 | -0.84050 | 0.73392 | 0.83705 |
| GOBP_HISTONE_MODIFICATION | Genetic and epigenetic information | -0.87039 | 0.83807 | 0.88692 | 0.85670 | 0.90569 | 0.72077 | -0.91020 | 0.89205 | 0.87060 |
| GOBP_ESTABLISHMENT_OF_RNA_LOCALIZATION | Genetic and epigenetic information | -0.53643 | 0.99990 | 0.88692 | 1.01319 | 0.47159 | 0.53134 | -1.15275 | 0.12812 | 0.40498 |
| GOBP_REGULATION_OF_HISTONE_MODIFICATION | Genetic and epigenetic information | -0.76724 | 0.93016 | 0.88692 | 0.63950 | 0.98850 | 0.72077 | -0.84346 | 0.90917 | 0.87060 |
| GOBP_REGULATION_OF_TELOMERE_MAINTENANCE_VIA_TELOMERE_LENGTHENING | Cell cycle | 0.83846 | 0.75403 | 0.86446 | 1.17368 | 0.22604 | 0.33372 | -0.80622 | 0.83723 | 0.87060 |
| GOBP_POSITIVE_REGULATION_OF_DOUBLE_STRAND_BREAK_REPAIR | Genetic and epigenetic information | -1.02930 | 0.41170 | 0.72312 | 1.29794 | 0.07386 | 0.15141 | -0.97165 | 0.51733 | 0.74603 |
| GOBP_RECOMBINATIONAL_REPAIR | Genetic and epigenetic information | -0.47544 | 1.00000 | 0.88692 | 1.45659 | 0.00220 | 0.01063 | -0.92821 | 0.69456 | 0.82532 |
| GOBP_GLUCOSE_CATABOLIC_PROCESS | Metabolism and energy | 2.22091 | 0.00005 | 0.00181 | -1.56734 | 0.02854 | 0.07618 | -0.71260 | 0.87926 | 0.87060 |
| GOBP_NEGATIVE_REGULATION_OF_DNA_REPAIR | Genetic and epigenetic information | 1.18742 | 0.22294 | 0.58181 | 0.90453 | 0.62630 | 0.61898 | 0.62992 | 0.93829 | 0.87060 |
| GOBP_FATTY_ACID_DERIVATIVE_CATABOLIC_PROCESS | Metabolism and energy | -1.18106 | 0.25619 | 0.60940 | -0.77866 | 0.79047 | 0.68946 | 0.62517 | 0.92638 | 0.87060 |
| GOBP_REGULATION_OF_GLYCOLYTIC_PROCESS | Metabolism and energy | 0.85999 | 0.69890 | 0.84746 | 0.97474 | 0.52910 | 0.56620 | 0.56772 | 0.98162 | 0.87060 |
| GOBP_NUCLEOSIDE_METABOLIC_PROCESS | Genetic and epigenetic information | -0.83640 | 0.74069 | 0.86073 | -1.22117 | 0.11891 | 0.21461 | -1.19726 | 0.15849 | 0.44613 |
| GOBP_DNA_RECOMBINATION | Cell cycle | -0.76210 | 0.97053 | 0.88692 | 1.32990 | 0.00446 | 0.01849 | -0.88719 | 0.88946 | 0.87060 |
| GOBP_REGULATION_OF_TELOMERE_CAPPING | Cell cycle | -0.73731 | 0.83390 | 0.88692 | 1.02171 | 0.45532 | 0.52229 | -0.44543 | 0.99900 | 0.87060 |
| GOBP_EXECUTION_PHASE_OF_APOPTOSIS | Cell death | 0.97443 | 0.49578 | 0.77042 | -0.67538 | 0.98586 | 0.72077 | -0.89450 | 0.68775 | 0.82372 |
| GOBP_CHAPERONE_MEDIATED_PROTEIN_FOLDING | Metabolism and energy | 0.96663 | 0.50925 | 0.77375 | -0.65957 | 0.98736 | 0.72077 | -0.82895 | 0.81529 | 0.86579 |
| GOBP_NADH_DEHYDROGENASE_COMPLEX_ASSEMBLY | Metabolism and energy | -0.48134 | 0.99870 | 0.88692 | 0.59282 | 0.97559 | 0.72077 | -1.23234 | 0.13112 | 0.40882 |
| GOBP_NEGATIVE_REGULATION_OF_TELOMERE_MAINTENANCE | Cell cycle | -0.92124 | 0.58374 | 0.80271 | 1.04823 | 0.41595 | 0.49649 | 0.57806 | 0.96846 | 0.87060 |
| GOBP_AMINO_ACID_ACTIVATION | Metabolism and energy | -0.98151 | 0.49431 | 0.76969 | 1.01760 | 0.46125 | 0.52459 | 0.57045 | 0.96745 | 0.87060 |
| GOBP_PROTON_MOTIVE_FORCE_DRIVEN_ATP_SYNTHESIS | Metabolism and energy | -0.45705 | 0.99965 | 0.88692 | -1.36164 | 0.03776 | 0.09309 | -1.83450 | 0.00120 | 0.02742 |
| GOBP_MITOCHONDRIAL_ELECTRON_TRANSPORT_NADH_TO_UBIQUINONE | Metabolism and energy | 0.81109 | 0.77533 | 0.87224 | -1.25246 | 0.11854 | 0.21424 | -1.59865 | 0.01814 | 0.14059 |
| GOBP_PROTEIN_FOLDING | Metabolism and energy | 1.00515 | 0.43476 | 0.73691 | 0.57799 | 0.99832 | 0.72077 | -1.00416 | 0.45181 | 0.71237 |
| GOBP_NEGATIVE_REGULATION_OF_HISTONE_MODIFICATION | Genetic and epigenetic information | -0.47885 | 0.99790 | 0.88692 | 0.83838 | 0.73564 | 0.66806 | -0.91738 | 0.60521 | 0.78933 |
| GOBP_HISTONE_H3_K4_TRIMETHYLATION | Genetic and epigenetic information | -0.65283 | 0.90903 | 0.88692 | 0.91133 | 0.60891 | 0.61104 | -0.65722 | 0.92552 | 0.87060 |
| GOBP_CHROMOSOME_SEPARATION | Cell cycle | -0.84066 | 0.79443 | 0.87715 | 1.75971 | 0.00000 | 0.00021 | -1.09910 | 0.21788 | 0.51811 |
| GOBP_REGULATION_OF_CHROMOSOME_SEGREGATION | Cell cycle | -0.82138 | 0.82629 | 0.88692 | 1.80628 | 0.00000 | 0.00009 | -1.28204 | 0.04287 | 0.23028 |
| GOBP_CELLULAR_CARBOHYDRATE_CATABOLIC_PROCESS | Metabolism and energy | 1.06468 | 0.34958 | 0.68256 | -1.13782 | 0.23240 | 0.34003 | -0.70221 | 0.93220 | 0.87060 |
| GOBP_REGULATION_OF_MRNA_CATABOLIC_PROCESS | Genetic and epigenetic information | -0.97881 | 0.51207 | 0.77460 | 0.91361 | 0.69834 | 0.65280 | -1.11937 | 0.16639 | 0.45614 |
| GOBP_REGULATION_OF_ANOIKIS | Cell death | 1.35403 | 0.11738 | 0.42908 | -1.41772 | 0.06438 | 0.13813 | -0.95871 | 0.50531 | 0.74081 |
| GOBP_POSITIVE_REGULATION_OF_RNA_SPLICING | Genetic and epigenetic information | -0.65039 | 0.94257 | 0.88692 | 0.97946 | 0.51968 | 0.56135 | -1.07551 | 0.31567 | 0.61238 |
| GOBP_REGULATION_OF_PROTEOLYSIS_INVOLVED_IN_PROTEIN_CATABOLIC_PROCESS | Metabolism and energy | -1.04413 | 0.36919 | 0.69724 | -0.72398 | 0.99955 | 0.72077 | -1.05347 | 0.28792 | 0.58739 |
| GOBP_ALTERNATIVE_MRNA_SPLICING_VIA_SPLICEOSOME | Genetic and epigenetic information | -0.84940 | 0.73584 | 0.86010 | 1.39395 | 0.03172 | 0.08220 | -1.35274 | 0.04642 | 0.24221 |
| GOBP_HISTONE_METHYLATION | Genetic and epigenetic information | -1.04876 | 0.36559 | 0.69625 | 1.17763 | 0.14523 | 0.24618 | -0.74594 | 0.98835 | 0.87060 |
| GOBP_NEGATIVE_REGULATION_OF_ATP_METABOLIC_PROCESS | Metabolism and energy | 1.08109 | 0.35082 | 0.68413 | -1.77436 | 0.00981 | 0.03391 | -1.04304 | 0.37838 | 0.66437 |
| GOBP_REGULATION_OF_CHROMOSOME_SEPARATION | Cell cycle | -0.87698 | 0.70980 | 0.85229 | 1.83399 | 0.00000 | 0.00027 | -1.33502 | 0.03160 | 0.19550 |
| GOBP_REGULATION_OF_DOUBLE_STRAND_BREAK_REPAIR_VIA_HOMOLOGOUS_RECOMBINATION | Genetic and epigenetic information | -0.63958 | 0.97405 | 0.88692 | 1.28496 | 0.09679 | 0.18532 | 0.53269 | 0.99503 | 0.87060 |
| GOBP_TRANSCRIPTION_BY_RNA_POLYMERASE_III | Genetic and epigenetic information | -0.55734 | 0.99102 | 0.88692 | 0.78601 | 0.81284 | 0.69780 | -0.93643 | 0.57461 | 0.77501 |
| GOBP_REGULATION_OF_INTRINSIC_APOPTOTIC_SIGNALING_PATHWAY | Cell death | 0.94441 | 0.60620 | 0.81299 | -1.59419 | 0.00025 | 0.00206 | -1.41766 | 0.00607 | 0.07527 |
| GOBP_REGULATION_OF_UBIQUITIN_DEPENDENT_PROTEIN_CATABOLIC_PROCESS | Immunity | -1.03978 | 0.38309 | 0.70547 | 0.41269 | 1.00000 | 0.72077 | -1.08401 | 0.23444 | 0.53298 |
| GOBP_REPLICATION_FORK_PROCESSING | Genetic and epigenetic information | -0.88851 | 0.64726 | 0.82972 | 1.47046 | 0.02217 | 0.06320 | 0.75748 | 0.84190 | 0.87060 |
| GOBP_POSITIVE_REGULATION_OF_PROTEIN_CATABOLIC_PROCESS | Metabolism and energy | -0.89768 | 0.70766 | 0.85129 | -1.26998 | 0.01448 | 0.04560 | -0.93257 | 0.70690 | 0.82851 |
| GOBP_DNA_UNWINDING_INVOLVED_IN_DNA_REPLICATION | Genetic and epigenetic information | 1.19598 | 0.22989 | 0.58924 | 1.53671 | 0.02146 | 0.06166 | -1.38531 | 0.08851 | 0.33899 |
| GOBP_NUCLEAR_TRANSCRIBED_MRNA_CATABOLIC_PROCESS | Genetic and epigenetic information | -0.71398 | 0.95747 | 0.88692 | 0.91552 | 0.67030 | 0.64015 | -1.31920 | 0.03240 | 0.19729 |
| GOBP_NEGATIVE_REGULATION_OF_INTRINSIC_APOPTOTIC_SIGNALING_PATHWAY | Cell death | 1.06282 | 0.32306 | 0.66213 | -1.23912 | 0.06768 | 0.14284 | -1.13958 | 0.18511 | 0.47980 |
| GOBP_PROTEIN_REFOLDING | Metabolism and energy | 0.81429 | 0.72424 | 0.85527 | 0.76642 | 0.80117 | 0.69341 | 0.86183 | 0.67579 | 0.81918 |
| GOBP_DNA_METHYLATION | Genetic and epigenetic information | -0.82774 | 0.77284 | 0.87083 | 1.04182 | 0.41880 | 0.49839 | -0.78136 | 0.90403 | 0.87060 |
| GOBP_REGULATION_OF_INTRINSIC_APOPTOTIC_SIGNALING_PATHWAY_IN_RESPONSE_TO_DNA_DAMAGE | Cell death | 1.51336 | 0.03759 | 0.24004 | -1.33162 | 0.07986 | 0.16058 | 1.04661 | 0.41414 | 0.69141 |
| GOBP_TELOMERE_CAPPING | Cell cycle | -0.93186 | 0.57079 | 0.79815 | 1.16502 | 0.25116 | 0.35850 | -0.52272 | 0.99698 | 0.87060 |
| GOBP_INTRINSIC_APOPTOTIC_SIGNALING_PATHWAY_IN_RESPONSE_TO_ENDOPLASMIC_RETICULUM_STRESS | Cell death | 1.25397 | 0.12513 | 0.44190 | -1.10356 | 0.25413 | 0.36115 | -1.09175 | 0.27819 | 0.57775 |
| GOBP_NUCLEOBASE_CONTAINING_COMPOUND_TRANSPORT | Genetic and epigenetic information | -0.67757 | 0.99371 | 0.88692 | 0.84614 | 0.83927 | 0.70623 | -1.21238 | 0.04850 | 0.24775 |
| GOBP_METAPHASE_ANAPHASE_TRANSITION_OF_CELL_CYCLE | Cell cycle | -0.88107 | 0.69516 | 0.84619 | 1.80107 | 0.00001 | 0.00044 | -1.29947 | 0.05011 | 0.25148 |
| GOBP_RNA_STABILIZATION | Genetic and epigenetic information | -0.78079 | 0.82861 | 0.88692 | 1.02852 | 0.44311 | 0.51510 | -1.19157 | 0.16086 | 0.44958 |
| GOBP_DNA_CATABOLIC_PROCESS_ENDONUCLEOLYTIC | Genetic and epigenetic information | -1.19685 | 0.23156 | 0.58981 | 0.98293 | 0.51051 | 0.55539 | -1.18367 | 0.21164 | 0.50995 |
| GOBP_AUTOPHAGY_OF_MITOCHONDRION | Cell death | -0.79443 | 0.83838 | 0.88692 | -1.08614 | 0.26474 | 0.37111 | -1.19047 | 0.13466 | 0.41437 |
| GOBP_NCRNA_TRANSCRIPTION | Genetic and epigenetic information | -0.30235 | 1.00000 | 0.88692 | 0.75283 | 0.85911 | 0.71329 | -1.05832 | 0.33415 | 0.62554 |
| GOBP_NEGATIVE_REGULATION_OF_METAPHASE_ANAPHASE_TRANSITION_OF_CELL_CYCLE | Cell cycle | -0.64479 | 0.95848 | 0.88692 | 2.05606 | 0.00000 | 0.00009 | -1.15086 | 0.21066 | 0.50890 |
| GOBP_REGULATION_OF_CYSTEINE_TYPE_ENDOPEPTIDASE_ACTIVITY_INVOLVED_IN_APOPTOTIC_SIGNALING_PATHWAY | Cell death | 1.65770 | 0.02776 | 0.20329 | -1.10538 | 0.30618 | 0.40949 | -0.78917 | 0.75694 | 0.84516 |
| GOBP_REGULATION_OF_MITOTIC_CELL_CYCLE_SPINDLE_ASSEMBLY_CHECKPOINT | Cell cycle | -1.03471 | 0.42456 | 0.73193 | 1.64313 | 0.00708 | 0.02650 | -1.23730 | 0.17409 | 0.46456 |
| GOBP_REGULATION_OF_HISTONE_DEACETYLATION | Genetic and epigenetic information | -0.63240 | 0.95625 | 0.88692 | -0.77533 | 0.87126 | 0.71679 | 0.38577 | 0.99978 | 0.87060 |
| GOBP_POSITIVE_REGULATION_OF_DNA_METABOLIC_PROCESS | Genetic and epigenetic information | -0.70450 | 0.99420 | 0.88692 | 1.05671 | 0.33935 | 0.43667 | -0.98349 | 0.53789 | 0.75755 |
| GOBP_HISTONE_H3_K4_METHYLATION | Genetic and epigenetic information | -1.00320 | 0.45814 | 0.75240 | 1.21231 | 0.16363 | 0.26847 | -0.65047 | 0.98966 | 0.87060 |
| GOBP_MITOCHONDRIAL_RESPIRATORY_CHAIN_COMPLEX_ASSEMBLY | Metabolism and energy | -0.71673 | 0.93890 | 0.88692 | 0.57817 | 0.99087 | 0.72077 | -1.62295 | 0.00268 | 0.04473 |
| GOBP_POSITIVE_REGULATION_OF_CHROMOSOME_SEPARATION | Cell cycle | -0.86360 | 0.66703 | 0.83543 | 1.62309 | 0.00732 | 0.02729 | -1.32579 | 0.10675 | 0.36862 |
| GOBP_EPIGENETIC_REGULATION_OF_GENE_EXPRESSION | Genetic and epigenetic information | -0.67779 | 0.98290 | 0.88692 | 1.09845 | 0.29439 | 0.39865 | -0.63099 | 0.99943 | 0.87060 |
| GOBP_CENTRIOLE_ASSEMBLY | Cell cycle | -0.88213 | 0.65647 | 0.83293 | 1.57920 | 0.00640 | 0.02449 | 0.63774 | 0.94979 | 0.87060 |
| GOBP_HOMOLOGOUS_CHROMOSOME_SEGREGATION | Cell cycle | -0.95636 | 0.53768 | 0.78709 | 1.73443 | 0.00029 | 0.00228 | 1.03100 | 0.43426 | 0.70113 |
| GOBP_DNA_BIOSYNTHETIC_PROCESS | Genetic and epigenetic information | 0.85767 | 0.86071 | 0.88692 | 1.06941 | 0.33539 | 0.43320 | -0.59310 | 1.00000 | 0.87060 |
| GOBP_NEGATIVE_REGULATION_OF_CHROMOSOME_ORGANIZATION | Cell cycle | -0.56335 | 0.99766 | 0.88692 | 1.79872 | 0.00001 | 0.00045 | -0.84605 | 0.82423 | 0.86849 |
| GOBP_REGULATION_OF_MITOTIC_SISTER_CHROMATID_SEGREGATION | Cell cycle | -0.71381 | 0.90878 | 0.88692 | 1.98772 | 0.00000 | 0.00009 | -1.00757 | 0.42660 | 0.69760 |
| GOBP_REGULATION_OF_MRNA_3_END_PROCESSING | Genetic and epigenetic information | -0.98775 | 0.48633 | 0.76529 | 0.74284 | 0.82499 | 0.70167 | -1.18055 | 0.21919 | 0.51812 |
| GOBP_NEGATIVE_REGULATION_OF_OXIDATIVE_STRESS_INDUCED_INTRINSIC_APOPTOTIC_SIGNALING_PATHWAY | Cell death | -1.34905 | 0.11591 | 0.42667 | 0.93603 | 0.57351 | 0.59210 | -0.50475 | 0.99207 | 0.87060 |
| GOBP_REGULATION_OF_CELL_CYCLE_CHECKPOINT | Cell cycle | -0.92175 | 0.59198 | 0.80397 | 1.75443 | 0.00037 | 0.00273 | -1.29308 | 0.09866 | 0.35399 |
| GOBP_CYTOKINESIS | Cell cycle | -0.90519 | 0.68163 | 0.84153 | 1.37055 | 0.00909 | 0.03220 | -0.74180 | 0.99394 | 0.87060 |
| GOBP_POSITIVE_REGULATION_OF_DNA_TEMPLATED_TRANSCRIPTION_INITIATION | Genetic and epigenetic information | 0.75927 | 0.89511 | 0.88692 | 0.65606 | 0.94956 | 0.72077 | 0.56628 | 0.98889 | 0.87060 |
| GOBP_DNA_METHYLATION_OR_DEMETHYLATION | Genetic and epigenetic information | -0.77405 | 0.87923 | 0.88692 | 0.92890 | 0.63380 | 0.62246 | -1.07639 | 0.28195 | 0.58272 |
| GOBP_RIBOSE_PHOSPHATE_BIOSYNTHETIC_PROCESS | Genetic and epigenetic information | 0.72495 | 0.99564 | 0.88692 | -0.65359 | 1.00000 | 0.72077 | -1.26581 | 0.02368 | 0.16554 |
| GOBP_NECROTIC_CELL_DEATH | Cell death | -0.89831 | 0.63941 | 0.82706 | -1.76137 | 0.00082 | 0.00497 | 0.51784 | 0.99584 | 0.87060 |
| GOBP_MITOTIC_CYTOKINESIS | Cell cycle | -0.69938 | 0.94786 | 0.88692 | 1.36397 | 0.03791 | 0.09329 | -1.10617 | 0.24158 | 0.53986 |
| GOBP_CELL_CYCLE_DNA_REPLICATION | Cell cycle | -0.82750 | 0.74120 | 0.86073 | 1.83958 | 0.00010 | 0.00111 | 0.76523 | 0.82687 | 0.86938 |
| GOBP_PYRIMIDINE_NUCLEOTIDE_CATABOLIC_PROCESS | Genetic and epigenetic information | -0.97152 | 0.50894 | 0.77345 | -0.80924 | 0.76021 | 0.67878 | -1.00818 | 0.42542 | 0.69717 |
| GOBP_HISTONE_H2A_ACETYLATION | Genetic and epigenetic information | -0.63305 | 0.92622 | 0.88692 | 0.85927 | 0.67999 | 0.64364 | -1.19385 | 0.20986 | 0.50775 |
| GOBP_REGULATION_OF_TYPE_I_INTERFERON_MEDIATED_SIGNALING_PATHWAY | Immunity | 1.05332 | 0.36648 | 0.69657 | -1.24132 | 0.13025 | 0.22836 | -1.27966 | 0.11854 | 0.38990 |
| GOBP_REGULATION_OF_PROTEIN_UBIQUITINATION | Immunity | -0.78800 | 0.91174 | 0.88692 | 0.40776 | 1.00000 | 0.72077 | -0.99288 | 0.48576 | 0.72935 |
| GOBP_3_UTR_MEDIATED_MRNA_STABILIZATION | Genetic and epigenetic information | -0.93465 | 0.55971 | 0.79590 | 1.00295 | 0.48188 | 0.53795 | 0.68056 | 0.88063 | 0.87060 |
| GOBP_POSITIVE_REGULATION_OF_PROTEOLYSIS | Metabolism and energy | -0.84802 | 0.86281 | 0.88692 | -1.76182 | 0.00200 | 0.00990 | -1.08004 | 0.16961 | 0.46063 |
| GOBP_REGULATION_OF_DNA_BIOSYNTHETIC_PROCESS | Genetic and epigenetic information | -0.86343 | 0.74213 | 0.86075 | 1.00997 | 0.47818 | 0.53570 | 0.62401 | 0.98606 | 0.87060 |
| GOBP_MRNA_MODIFICATION | Genetic and epigenetic information | 0.99667 | 0.45142 | 0.74740 | 0.67132 | 0.90363 | 0.72077 | -0.85564 | 0.69278 | 0.82530 |
| GOBP_HISTONE_MRNA_METABOLIC_PROCESS | Genetic and epigenetic information | 0.47524 | 0.99102 | 0.88692 | 0.82845 | 0.71720 | 0.66103 | -0.60419 | 0.95733 | 0.87060 |
| GOBP_TRNA_METHYLATION | Genetic and epigenetic information | -1.09429 | 0.33056 | 0.66700 | 0.96892 | 0.53665 | 0.57201 | -0.77565 | 0.85628 | 0.87060 |
| GOBP_NEGATIVE_REGULATION_OF_DNA_METABOLIC_PROCESS | Genetic and epigenetic information | 0.77505 | 0.93944 | 0.88692 | 1.11029 | 0.27714 | 0.38267 | 0.89586 | 0.69776 | 0.82593 |
| GOBP_PYRIMIDINE_RIBONUCLEOTIDE_BIOSYNTHETIC_PROCESS | Genetic and epigenetic information | -0.98327 | 0.49247 | 0.76857 | -0.79606 | 0.78410 | 0.68750 | 0.49688 | 0.98808 | 0.87060 |
| GOBP_REGULATION_OF_MITOTIC_CELL_CYCLE | Cell cycle | -0.85367 | 0.87827 | 0.88692 | 1.32681 | 0.00067 | 0.00432 | -1.20802 | 0.00750 | 0.08620 |
| GOBP_POLYSACCHARIDE_CATABOLIC_PROCESS | Metabolism and energy | 1.11473 | 0.31470 | 0.65757 | -1.73244 | 0.01273 | 0.04144 | 0.55054 | 0.96846 | 0.87060 |
| GOBP_NEGATIVE_REGULATION_OF_MACROAUTOPHAGY | Cell death | -0.79886 | 0.77276 | 0.87083 | -1.22618 | 0.14878 | 0.25072 | -0.78184 | 0.83073 | 0.87006 |
| GOBP_RESPIRATORY_ELECTRON_TRANSPORT_CHAIN | Metabolism and energy | 1.01851 | 0.40595 | 0.72060 | -1.00651 | 0.44590 | 0.51607 | -1.53817 | 0.00523 | 0.06781 |
| GOBP_POSITIVE_REGULATION_OF_CELL_CYCLE | Cell cycle | -0.90581 | 0.72073 | 0.85522 | 1.47812 | 0.00004 | 0.00062 | -1.02530 | 0.36746 | 0.65510 |
| GOBP_NEGATIVE_REGULATION_OF_NUCLEAR_DIVISION | Cell cycle | 0.73014 | 0.92492 | 0.88692 | 1.98013 | 0.00000 | 0.00009 | 0.92268 | 0.61575 | 0.79492 |
| GOBP_DNA_TEMPLATED_DNA_REPLICATION_MAINTENANCE_OF_FIDELITY | Genetic and epigenetic information | -0.77140 | 0.84188 | 0.88692 | 1.61551 | 0.00266 | 0.01239 | 0.94281 | 0.58007 | 0.77722 |
| GOBP_DNA_REPLICATION_INITIATION | Genetic and epigenetic information | 0.93655 | 0.55107 | 0.79204 | 1.70456 | 0.00149 | 0.00786 | -0.86008 | 0.70724 | 0.82851 |
| GOBP_NEGATIVE_REGULATION_OF_PROTEASOMAL_PROTEIN_CATABOLIC_PROCESS | Immunity | -0.93004 | 0.57704 | 0.79940 | 0.76284 | 0.83410 | 0.70491 | 0.66517 | 0.92971 | 0.87060 |
| GOBP_REGULATION_OF_PROTEIN_STABILITY | Metabolism and energy | -0.85821 | 0.82670 | 0.88692 | -0.74465 | 0.99911 | 0.72077 | -1.05232 | 0.26866 | 0.56879 |
| GOBP_REGULATION_OF_NUCLEOTIDE_EXCISION_REPAIR | Genetic and epigenetic information | -1.08182 | 0.35763 | 0.69029 | 1.32244 | 0.10967 | 0.20303 | -1.15439 | 0.23275 | 0.53195 |
| GOBP_N_TERMINAL_PROTEIN_AMINO_ACID_MODIFICATION | Metabolism and energy | -0.79232 | 0.76977 | 0.87067 | 1.02886 | 0.44487 | 0.51578 | -0.90851 | 0.59813 | 0.78561 |
| GOBP_G1_TO_G0_TRANSITION | Cell cycle | -1.25159 | 0.18872 | 0.54202 | 0.98148 | 0.51137 | 0.55562 | -0.94163 | 0.52598 | 0.75201 |
| GOBP_PYRIMIDINE_NUCLEOSIDE_METABOLIC_PROCESS | Genetic and epigenetic information | 1.14693 | 0.27196 | 0.61963 | -1.59666 | 0.02153 | 0.06182 | -1.19851 | 0.19876 | 0.49206 |
| GOBP_NEGATIVE_REGULATION_OF_CELL_CYCLE_PROCESS | Cell cycle | -0.99104 | 0.49290 | 0.76857 | 1.63045 | 0.00000 | 0.00009 | -0.94313 | 0.71082 | 0.82966 |
| GOBP_REGULATION_OF_UBIQUITIN_PROTEIN_LIGASE_ACTIVITY | Immunity | 0.74813 | 0.78319 | 0.87503 | 1.62006 | 0.01075 | 0.03650 | -1.36353 | 0.11325 | 0.38105 |
| GOBP_REGULATION_OF_HISTONE_H3_K4_METHYLATION | Genetic and epigenetic information | -1.09289 | 0.33447 | 0.66868 | 1.11159 | 0.32276 | 0.42253 | -0.87743 | 0.67461 | 0.81885 |
| GOBP_REGULATION_OF_ATTACHMENT_OF_SPINDLE_MICROTUBULES_TO_KINETOCHORE | Cell cycle | -0.79982 | 0.73977 | 0.86073 | 1.66405 | 0.00637 | 0.02446 | -1.68456 | 0.02541 | 0.17236 |
| GOBP_RNA_POLYMERASE_II_PREINITIATION_COMPLEX_ASSEMBLY | Genetic and epigenetic information | 0.62119 | 0.98291 | 0.88692 | 0.82097 | 0.77043 | 0.68330 | 0.52092 | 0.99465 | 0.87060 |
| GOBP_NEGATIVE_REGULATION_OF_AUTOPHAGY | Cell death | -0.63343 | 0.98409 | 0.88692 | -1.35537 | 0.02478 | 0.06841 | -1.33430 | 0.04569 | 0.23912 |
| GOBP_PROGRAMMED_NECROTIC_CELL_DEATH | Cell death | -1.05593 | 0.38084 | 0.70315 | -1.78137 | 0.00163 | 0.00845 | 0.55430 | 0.98536 | 0.87060 |
| GOBP_CHAPERONE_MEDIATED_PROTEIN_COMPLEX_ASSEMBLY | Metabolism and energy | -1.24320 | 0.18938 | 0.54215 | 0.79775 | 0.76200 | 0.67946 | 0.93603 | 0.57158 | 0.77324 |
| GOBP_RIBONUCLEOSIDE_MONOPHOSPHATE_BIOSYNTHETIC_PROCESS | Genetic and epigenetic information | 1.13246 | 0.27605 | 0.62266 | -0.76165 | 0.86374 | 0.71447 | -1.01965 | 0.40648 | 0.68586 |
| GOBP_CELL_CYCLE_CHECKPOINT_SIGNALING | Cell cycle | -0.61205 | 0.99910 | 0.88692 | 1.75852 | 0.00000 | 0.00009 | -0.95013 | 0.63503 | 0.80266 |
| GOBP_NEGATIVE_REGULATION_OF_UBIQUITIN_DEPENDENT_PROTEIN_CATABOLIC_PROCESS | Immunity | -0.75724 | 0.84319 | 0.88692 | 0.57181 | 0.97813 | 0.72077 | -1.22998 | 0.14501 | 0.42995 |
| GOBP_POSITIVE_REGULATION_OF_DNA_TEMPLATED_TRANSCRIPTION_ELONGATION | Genetic and epigenetic information | -0.46122 | 0.99960 | 0.88692 | 0.89405 | 0.66801 | 0.63987 | 0.46147 | 0.99916 | 0.87060 |
| GOBP_REGULATION_OF_GLUCONEOGENESIS | Metabolism and energy | -0.91751 | 0.59996 | 0.80983 | -0.72751 | 0.93901 | 0.72077 | -0.96407 | 0.51423 | 0.74414 |
| GOBP_CYTOSKELETON_DEPENDENT_CYTOKINESIS | Cell cycle | -0.78917 | 0.86658 | 0.88692 | 1.31039 | 0.04998 | 0.11427 | -0.87025 | 0.80044 | 0.86021 |
| GOBP_INTRINSIC_APOPTOTIC_SIGNALING_PATHWAY | Cell death | 1.25115 | 0.03141 | 0.21892 | -1.53083 | 0.00065 | 0.00422 | -1.34385 | 0.00255 | 0.04367 |
| GOBP_MONOSACCHARIDE_CATABOLIC_PROCESS | Metabolism and energy | 1.79546 | 0.00339 | 0.05158 | -1.25531 | 0.11354 | 0.20753 | 0.71278 | 0.88377 | 0.87060 |
| GOBP_NEGATIVE_REGULATION_OF_CYCLIN_DEPENDENT_PROTEIN_SERINE_THREONINE_KINASE_ACTIVITY | Cell cycle | -0.96755 | 0.51452 | 0.77597 | 1.08430 | 0.36626 | 0.45756 | -0.79870 | 0.78903 | 0.85705 |
| GOBP_NUCLEOSIDE_MONOPHOSPHATE_BIOSYNTHETIC_PROCESS | Genetic and epigenetic information | 1.08267 | 0.32588 | 0.66439 | 0.38251 | 0.99975 | 0.72077 | -1.05123 | 0.35207 | 0.64011 |
| GOBP_PYRIMIDINE_DEOXYRIBONUCLEOTIDE_METABOLIC_PROCESS | Genetic and epigenetic information | -0.85763 | 0.67162 | 0.83731 | 0.73677 | 0.83425 | 0.70491 | -0.73209 | 0.86323 | 0.87060 |
| GOBP_NEGATIVE_REGULATION_OF_NUCLEOTIDE_METABOLIC_PROCESS | Genetic and epigenetic information | 0.99154 | 0.45845 | 0.75240 | -1.52081 | 0.03572 | 0.08960 | -0.72350 | 0.87061 | 0.87060 |
| GOBP_MITOTIC_CELL_CYCLE_PHASE_TRANSITION | Cell cycle | -0.68196 | 0.99960 | 0.88692 | 1.35273 | 0.00045 | 0.00319 | -1.09312 | 0.10411 | 0.36434 |
| GOBP_HISTONE_H3_K9_TRIMETHYLATION | Genetic and epigenetic information | 0.69428 | 0.85494 | 0.88692 | 0.86155 | 0.67354 | 0.64134 | 0.69456 | 0.86566 | 0.87060 |
| GOBP_DNA_DAMAGE_RESPONSE_SIGNAL_TRANSDUCTION_RESULTING_IN_TRANSCRIPTION | Genetic and epigenetic information | -0.90102 | 0.60554 | 0.81299 | 0.67878 | 0.88005 | 0.71913 | -0.80600 | 0.73280 | 0.83705 |
| GOBP_REGULATION_OF_CARBOHYDRATE_METABOLIC_PROCESS | Metabolism and energy | 1.08232 | 0.25051 | 0.60342 | 0.61674 | 0.99350 | 0.72077 | 0.60368 | 0.99601 | 0.87060 |
| GOBP_REGULATION_OF_UBIQUITIN_PROTEIN_TRANSFERASE_ACTIVITY | Immunity | 0.79993 | 0.79717 | 0.87808 | 0.98942 | 0.50545 | 0.55157 | -1.31562 | 0.09241 | 0.34480 |
| GOBP_NEGATIVE_REGULATION_OF_CELL_CYCLE | Cell cycle | -1.10502 | 0.22064 | 0.58130 | 1.48924 | 0.00001 | 0.00038 | -0.98283 | 0.56623 | 0.77095 |
| GOBP_DNA_INTEGRITY_CHECKPOINT_SIGNALING | Cell cycle | -0.66052 | 0.98707 | 0.88692 | 1.60310 | 0.00025 | 0.00206 | -0.70395 | 0.99143 | 0.87060 |
| GOBP_POSITIVE_REGULATION_OF_DOUBLE_STRAND_BREAK_REPAIR_VIA_HOMOLOGOUS_RECOMBINATION | Genetic and epigenetic information | -0.87256 | 0.66553 | 0.83464 | 1.06844 | 0.38402 | 0.47146 | -0.98807 | 0.46019 | 0.71866 |
| GOBP_POSITIVE_REGULATION_OF_HISTONE_DEACETYLATION | Genetic and epigenetic information | -0.79656 | 0.75326 | 0.86446 | -1.27280 | 0.14005 | 0.23995 | -0.71645 | 0.87489 | 0.87060 |
| GOBP_ATP_SYNTHESIS_COUPLED_ELECTRON_TRANSPORT | Metabolism and energy | 0.96141 | 0.52738 | 0.78238 | -0.64170 | 0.99566 | 0.72077 | -1.54566 | 0.00895 | 0.09600 |
| GOBP_GLUCOSE_METABOLIC_PROCESS | Metabolism and energy | 1.42716 | 0.00800 | 0.09391 | -1.20947 | 0.04449 | 0.10516 | -0.79924 | 0.96558 | 0.87060 |
| GOBP_REGULATION_OF_GLUCOSE_METABOLIC_PROCESS | Metabolism and energy | 1.19384 | 0.14036 | 0.46781 | -0.83747 | 0.88308 | 0.71997 | 0.52748 | 0.99836 | 0.87060 |
| GOBP_REGULATION_OF_PROTEIN_POLYUBIQUITINATION | Immunity | 0.59356 | 0.96761 | 0.88692 | 0.83366 | 0.72426 | 0.66335 | 0.65003 | 0.92260 | 0.87060 |
| GOBP_ADP_METABOLIC_PROCESS | Metabolism and energy | 1.51454 | 0.01383 | 0.13107 | 0.90076 | 0.67416 | 0.64168 | 0.63575 | 0.97166 | 0.87060 |
| GOBP_POSITIVE_REGULATION_OF_CELL_CYCLE_PROCESS | Cell cycle | -0.93027 | 0.63967 | 0.82706 | 1.56269 | 0.00001 | 0.00046 | -0.81979 | 0.97024 | 0.87060 |
| GOBP_ESTABLISHMENT_OF_SPINDLE_ORIENTATION | Cell cycle | 0.77175 | 0.83148 | 0.88692 | 1.32429 | 0.09418 | 0.18200 | 0.71745 | 0.87685 | 0.87060 |
| GOBP_PYRIMIDINE_RIBONUCLEOTIDE_METABOLIC_PROCESS | Genetic and epigenetic information | -1.06925 | 0.37289 | 0.69893 | -0.95678 | 0.52911 | 0.56620 | -0.65260 | 0.95055 | 0.87060 |
| GOBP_REGULATION_OF_CELLULAR_CARBOHYDRATE_METABOLIC_PROCESS | Metabolism and energy | 1.18197 | 0.12372 | 0.44033 | -0.84100 | 0.91516 | 0.72077 | -0.78329 | 0.96512 | 0.87060 |
| GOBP_POSITIVE_REGULATION_OF_MRNA_METABOLIC_PROCESS | Genetic and epigenetic information | -0.91184 | 0.64744 | 0.82972 | 0.85385 | 0.78298 | 0.68731 | -1.20446 | 0.08941 | 0.33928 |
| GOBP_DNA_CATABOLIC_PROCESS | Genetic and epigenetic information | -1.36607 | 0.09158 | 0.38511 | 0.75458 | 0.82176 | 0.70030 | -1.26295 | 0.14015 | 0.42214 |
| GOBP_DNA_DAMAGE_RESPONSE_SIGNAL_TRANSDUCTION_BY_P53_CLASS_MEDIATOR_RESULTING_IN_CELL_CYCLE_ARREST | Cell cycle | -0.89403 | 0.61528 | 0.81629 | 0.81708 | 0.73016 | 0.66558 | 0.82539 | 0.71565 | 0.83162 |
| GOBP_PURINE_CONTAINING_COMPOUND_BIOSYNTHETIC_PROCESS | Genetic and epigenetic information | -0.69329 | 0.99167 | 0.88692 | 0.51781 | 0.99991 | 0.72077 | -1.23962 | 0.03180 | 0.19621 |
| GOBP_MRNA_CLEAVAGE | Genetic and epigenetic information | -1.21102 | 0.22490 | 0.58381 | 0.69489 | 0.86944 | 0.71613 | -1.03711 | 0.38558 | 0.66997 |
| GOBP_POSITIVE_REGULATION_OF_TRANSLATION | Metabolism and energy | 0.96069 | 0.54541 | 0.78956 | -0.68718 | 0.99656 | 0.72077 | -1.46240 | 0.00707 | 0.08257 |
| GOBP_DEOXYRIBONUCLEOTIDE_CATABOLIC_PROCESS | Genetic and epigenetic information | -0.77140 | 0.79768 | 0.87808 | -0.76219 | 0.85333 | 0.71147 | -0.89491 | 0.62308 | 0.79786 |
| GOBP_OXIDATIVE_PHOSPHORYLATION | Metabolism and energy | 1.20475 | 0.11706 | 0.42908 | -0.87393 | 0.83191 | 0.70395 | -1.78026 | 0.00018 | 0.00805 |
| GOBP_SNRNA_METABOLIC_PROCESS | Genetic and epigenetic information | -0.58299 | 0.98709 | 0.88692 | 1.08639 | 0.34989 | 0.44447 | -0.67262 | 0.97267 | 0.87060 |
| GOBP_PURINE_CONTAINING_COMPOUND_METABOLIC_PROCESS | Genetic and epigenetic information | -0.75076 | 0.99109 | 0.88692 | 0.53485 | 1.00000 | 0.72077 | 0.61750 | 0.99972 | 0.87060 |
| GOBP_REGULATION_OF_PROTEIN_CATABOLIC_PROCESS | Metabolism and energy | -0.77060 | 0.97076 | 0.88692 | -0.95867 | 0.71482 | 0.66033 | -1.19733 | 0.01792 | 0.13994 |
| GOBP_REGULATION_OF_CELL_CYCLE_PHASE_TRANSITION | Cell cycle | -0.93557 | 0.65994 | 0.83351 | 1.44974 | 0.00001 | 0.00045 | -1.11262 | 0.07637 | 0.31358 |
| GOBP_NUCLEOSIDE_MONOPHOSPHATE_METABOLIC_PROCESS | Genetic and epigenetic information | -0.66503 | 0.96293 | 0.88692 | -0.91310 | 0.67975 | 0.64364 | -0.97583 | 0.50097 | 0.73864 |
| GOBP_NCRNA_CATABOLIC_PROCESS | Genetic and epigenetic information | 0.80101 | 0.79134 | 0.87715 | 0.53284 | 0.98815 | 0.72077 | 0.66766 | 0.92360 | 0.87060 |
| GOBP_REGULATION_OF_EXTRINSIC_APOPTOTIC_SIGNALING_PATHWAY_IN_ABSENCE_OF_LIGAND | Cell death | 1.17797 | 0.21001 | 0.56852 | 1.30333 | 0.10491 | 0.19627 | 1.08418 | 0.35623 | 0.64318 |
| GOBP_POSITIVE_REGULATION_OF_CYTOKINESIS | Cell cycle | -0.72764 | 0.87427 | 0.88692 | 1.51688 | 0.01572 | 0.04872 | 0.60918 | 0.96162 | 0.87060 |
| GOBP_PROTEIN_STABILIZATION | Metabolism and energy | -0.88826 | 0.72313 | 0.85522 | -0.82181 | 0.96145 | 0.72077 | -1.18829 | 0.07129 | 0.30361 |
| GOBP_MITOTIC_CELL_CYCLE_CHECKPOINT_SIGNALING | Cell cycle | -0.66742 | 0.98749 | 0.88692 | 1.74171 | 0.00000 | 0.00015 | -0.96098 | 0.57670 | 0.77629 |
| GOBP_REGULATION_OF_NECROTIC_CELL_DEATH | Cell death | -1.05447 | 0.38549 | 0.70700 | -1.41959 | 0.04002 | 0.09711 | 0.64708 | 0.93792 | 0.87060 |
| GOBP_REGULATION_OF_CHOLESTEROL_BIOSYNTHETIC_PROCESS | Metabolism and energy | 0.68885 | 0.86871 | 0.88692 | -1.16521 | 0.23630 | 0.34400 | -0.80602 | 0.74436 | 0.84107 |
| GOBP_ELECTRON_TRANSPORT_CHAIN | Metabolism and energy | 1.02023 | 0.39596 | 0.71285 | -1.50291 | 0.00052 | 0.00358 | -1.34369 | 0.01623 | 0.13435 |
| GOBP_PYRIMIDINE_NUCLEOTIDE_METABOLIC_PROCESS | Genetic and epigenetic information | -1.05815 | 0.37568 | 0.70055 | -0.90476 | 0.66580 | 0.63902 | -0.77836 | 0.86743 | 0.87060 |
| GOBP_PURINE_NUCLEOSIDE_METABOLIC_PROCESS | Genetic and epigenetic information | -1.08330 | 0.35756 | 0.69029 | -0.59251 | 0.98013 | 0.72077 | -1.15722 | 0.23366 | 0.53298 |
| GOBP_NEGATIVE_REGULATION_OF_TYPE_I_INTERFERON_MEDIATED_SIGNALING_PATHWAY | Immunity | 1.13188 | 0.29791 | 0.64182 | -1.26503 | 0.15617 | 0.25978 | -1.56275 | 0.04302 | 0.23040 |
| GOBP_INTRINSIC_APOPTOTIC_SIGNALING_PATHWAY_IN_RESPONSE_TO_DNA_DAMAGE | Cell death | 1.34707 | 0.04570 | 0.26769 | -1.48884 | 0.00400 | 0.01703 | -0.93616 | 0.62140 | 0.79729 |
| GOBP_MESENCHYMAL_CELL_MIGRATION | ECM and metastasis | -0.94690 | 0.55420 | 0.79414 | 1.14728 | 0.25546 | 0.36270 | 1.54364 | 0.00813 | 0.09147 |
| GOBP_POSITIVE_REGULATION_OF_CYCLIN_DEPENDENT_PROTEIN_KINASE_ACTIVITY | Cell cycle | -0.76799 | 0.82000 | 0.88608 | 1.05800 | 0.39997 | 0.48357 | -0.86800 | 0.69227 | 0.82515 |
| GOBP_DEOXYRIBONUCLEOSIDE_MONOPHOSPHATE_METABOLIC_PROCESS | Genetic and epigenetic information | -0.74376 | 0.82394 | 0.88692 | -0.70340 | 0.90522 | 0.72077 | -1.00436 | 0.43243 | 0.70113 |
| GOBP_FATTY_ACYL_COA_METABOLIC_PROCESS | Metabolism and energy | -0.69558 | 0.90191 | 0.88692 | 0.90482 | 0.63245 | 0.62172 | 0.55737 | 0.98043 | 0.87060 |
| GOBP_REGULATION_OF_CYTOKINESIS | Cell cycle | -0.94516 | 0.56593 | 0.79753 | 1.58071 | 0.00155 | 0.00815 | -0.75506 | 0.94774 | 0.87060 |
| GOBP_REGULATION_OF_DNA_DAMAGE_CHECKPOINT | Cell cycle | -0.79699 | 0.75993 | 0.86659 | 1.54866 | 0.01717 | 0.05214 | -0.73260 | 0.86977 | 0.87060 |
| GOBP_KINETOCHORE_ORGANIZATION | Cell cycle | 0.78774 | 0.75989 | 0.86659 | 1.99296 | 0.00003 | 0.00060 | -0.65307 | 0.93691 | 0.87060 |
| GOBP_SMALL_REGULATORY_NCRNA_PROCESSING | Genetic and epigenetic information | 0.64389 | 0.97602 | 0.88692 | -0.85892 | 0.77422 | 0.68454 | -0.99089 | 0.46099 | 0.71866 |
| GOBP_SPINDLE_LOCALIZATION | Cell cycle | 0.74265 | 0.90619 | 0.88692 | 1.44319 | 0.02418 | 0.06730 | 0.65215 | 0.94984 | 0.87060 |
| GOBP_SIGNAL_TRANSDUCTION_IN_RESPONSE_TO_DNA_DAMAGE | Genetic and epigenetic information | -0.67706 | 0.99090 | 0.88692 | 1.26298 | 0.05088 | 0.11585 | -0.95003 | 0.62729 | 0.79952 |
| GOBP_REGULATION_OF_HISTONE_METHYLATION | Genetic and epigenetic information | -0.95446 | 0.54610 | 0.78986 | 1.06450 | 0.37774 | 0.46664 | -0.90616 | 0.67529 | 0.81900 |
| GOBP_CARBOHYDRATE_CATABOLIC_PROCESS | Metabolism and energy | 1.29136 | 0.04950 | 0.28016 | -1.35365 | 0.00938 | 0.03292 | 0.57329 | 0.99733 | 0.87060 |
| GOBP_NUCLEOSIDE_BISPHOSPHATE_BIOSYNTHETIC_PROCESS | Genetic and epigenetic information | -0.65020 | 0.96318 | 0.88692 | 0.68630 | 0.92427 | 0.72077 | 0.60360 | 0.97578 | 0.87060 |
| GOBP_POSITIVE_REGULATION_OF_TELOMERE_MAINTENANCE_VIA_TELOMERE_LENGTHENING | Cell cycle | 1.16879 | 0.23606 | 0.59150 | 0.92181 | 0.60394 | 0.60842 | -1.42703 | 0.05994 | 0.27791 |
| GOBP_REGULATION_OF_DNA_TEMPLATED_DNA_REPLICATION | Genetic and epigenetic information | 0.37941 | 0.99999 | 0.88692 | 1.69634 | 0.00070 | 0.00441 | 0.79813 | 0.79988 | 0.86021 |
| GOBP_NEGATIVE_REGULATION_OF_MITOTIC_CELL_CYCLE | Cell cycle | -1.01190 | 0.44019 | 0.74070 | 1.49186 | 0.00023 | 0.00195 | -1.00254 | 0.45521 | 0.71501 |
| GOBP_RNA_DESTABILIZATION | Genetic and epigenetic information | -0.93154 | 0.59474 | 0.80655 | 0.83150 | 0.79362 | 0.69036 | -1.20651 | 0.11165 | 0.37775 |
| GOBP_NUCLEOSOME_ORGANIZATION | Cell cycle | -1.02614 | 0.41934 | 0.72789 | 1.28558 | 0.09208 | 0.17922 | -1.55687 | 0.00937 | 0.09841 |
| GOBP_PYRIMIDINE_NUCLEOSIDE_MONOPHOSPHATE_METABOLIC_PROCESS | Genetic and epigenetic information | -0.93830 | 0.55504 | 0.79427 | -0.74346 | 0.84982 | 0.70999 | 0.62461 | 0.93107 | 0.87060 |
| GOBP_HISTONE_H4_ACETYLATION | Genetic and epigenetic information | -0.75805 | 0.87369 | 0.88692 | 0.89635 | 0.67154 | 0.64095 | -0.78686 | 0.89115 | 0.87060 |
| GOBP_CARBOHYDRATE_PHOSPHORYLATION | Metabolism and energy | -0.62205 | 0.93769 | 0.88692 | -0.84596 | 0.71177 | 0.65926 | -1.11267 | 0.28762 | 0.58739 |
| GOBP_POSITIVE_REGULATION_OF_TRANSCRIPTION_ELONGATION_BY_RNA_POLYMERASE_II | Genetic and epigenetic information | -0.51868 | 0.99584 | 0.88692 | 0.71775 | 0.88674 | 0.72077 | 0.37129 | 0.99993 | 0.87060 |
| GOBP_INTRINSIC_APOPTOTIC_SIGNALING_PATHWAY_BY_P53_CLASS_MEDIATOR | Cell death | -0.80182 | 0.81535 | 0.88529 | 0.75561 | 0.87076 | 0.71669 | 0.76233 | 0.86077 | 0.87060 |
| GOBP_NUCLEOSIDE_DIPHOSPHATE_METABOLIC_PROCESS | Genetic and epigenetic information | 1.25225 | 0.08086 | 0.36132 | 0.81112 | 0.84077 | 0.70692 | 0.54376 | 0.99842 | 0.87060 |
| GOBP_APOPTOTIC_DNA_FRAGMENTATION | Cell death | -1.30938 | 0.14448 | 0.47533 | 0.86034 | 0.67375 | 0.64143 | -1.53915 | 0.05075 | 0.25304 |
| GOBP_PHOSPHATIDIC_ACID_METABOLIC_PROCESS | Metabolism and energy | 1.02016 | 0.41445 | 0.72510 | -1.27552 | 0.10801 | 0.20083 | 1.02493 | 0.44563 | 0.70792 |
| GOBP_NEGATIVE_REGULATION_OF_PROTEOLYSIS_INVOLVED_IN_PROTEIN_CATABOLIC_PROCESS | Metabolism and energy | -0.85851 | 0.70587 | 0.85127 | 0.52948 | 0.99322 | 0.72077 | -0.94917 | 0.55204 | 0.76495 |
| GOBP_ATTACHMENT_OF_MITOTIC_SPINDLE_MICROTUBULES_TO_KINETOCHORE | Cell cycle | -0.74378 | 0.81817 | 0.88533 | 1.51673 | 0.02651 | 0.07215 | -0.94480 | 0.52519 | 0.75201 |
| GOBP_REGULATION_OF_APOPTOTIC_SIGNALING_PATHWAY | Cell death | 1.13083 | 0.11125 | 0.41658 | -1.57231 | 0.00184 | 0.00930 | -1.06733 | 0.20854 | 0.50616 |
| GOBP_REGULATION_OF_MITOTIC_CELL_CYCLE_PHASE_TRANSITION | Cell cycle | -0.74111 | 0.98523 | 0.88692 | 1.39306 | 0.00064 | 0.00416 | -1.02700 | 0.36490 | 0.65182 |
| GOBP_MONOSACCHARIDE_BIOSYNTHETIC_PROCESS | Metabolism and energy | 1.07918 | 0.29965 | 0.64285 | -1.26427 | 0.05868 | 0.12874 | -1.04466 | 0.35089 | 0.63947 |
| GOBP_NEGATIVE_REGULATION_OF_CELL_CYCLE_G2_M_PHASE_TRANSITION | Cell cycle | 0.71879 | 0.93571 | 0.88692 | 1.44921 | 0.02094 | 0.06055 | -1.07611 | 0.30222 | 0.60071 |
| GOBP_HISTONE_H3_K9_ACETYLATION | Genetic and epigenetic information | -1.13466 | 0.30634 | 0.65133 | 1.04153 | 0.42968 | 0.50656 | 0.96480 | 0.53009 | 0.75361 |
| GOBP_NUCLEOSIDE_PHOSPHATE_BIOSYNTHETIC_PROCESS | Genetic and epigenetic information | -0.70249 | 0.99345 | 0.88692 | 0.47316 | 1.00000 | 0.72077 | -1.06427 | 0.23533 | 0.53298 |
| GOBP_ENDOTHELIAL_CELL_CHEMOTAXIS | ECM and metastasis | 1.19138 | 0.22458 | 0.58344 | -1.51042 | 0.03351 | 0.08556 | -1.20810 | 0.18400 | 0.47902 |
| GOBP_FATTY_ACID_CATABOLIC_PROCESS | Metabolism and energy | -1.05848 | 0.35908 | 0.69153 | -0.68958 | 0.99341 | 0.72077 | 0.60321 | 0.98812 | 0.87060 |
| GOBP_MONOSACCHARIDE_METABOLIC_PROCESS | Metabolism and energy | 1.29156 | 0.02452 | 0.18687 | -1.20542 | 0.03080 | 0.08067 | -0.80060 | 0.98165 | 0.87060 |
| GOBP_POSITIVE_REGULATION_OF_CELL_CYCLE_PHASE_TRANSITION | Cell cycle | -0.92246 | 0.61859 | 0.81759 | 1.40506 | 0.01541 | 0.04791 | 0.52251 | 0.99893 | 0.87060 |
| GOBP_NEGATIVE_REGULATION_OF_PROTEIN_CATABOLIC_PROCESS | Metabolism and energy | 0.92172 | 0.63565 | 0.82645 | 0.75465 | 0.89499 | 0.72077 | -1.41592 | 0.01590 | 0.13285 |
| GOBP_SELECTIVE_AUTOPHAGY | Cell death | -0.73169 | 0.91481 | 0.88692 | -1.03337 | 0.38097 | 0.46905 | -0.83304 | 0.83353 | 0.87060 |
| GOBP_NEGATIVE_REGULATION_OF_HISTONE_METHYLATION | Genetic and epigenetic information | -0.50298 | 0.98665 | 0.88692 | 1.23042 | 0.19833 | 0.30458 | -0.78241 | 0.78597 | 0.85642 |
| GOBP_NUCLEOTIDE_EXCISION_REPAIR | Genetic and epigenetic information | -1.04293 | 0.38920 | 0.70968 | 1.26664 | 0.09989 | 0.18945 | -0.72670 | 0.96564 | 0.87060 |
| GOBP_MITOTIC_DNA_INTEGRITY_CHECKPOINT_SIGNALING | Cell cycle | -0.86727 | 0.71105 | 0.85294 | 1.51094 | 0.00573 | 0.02246 | -0.67401 | 0.98779 | 0.87060 |
| GOBP_REGULATION_OF_TRANSCRIPTION_BY_RNA_POLYMERASE_III | Genetic and epigenetic information | 0.48856 | 0.99437 | 0.88692 | 0.82679 | 0.73161 | 0.66619 | 0.76997 | 0.80014 | 0.86021 |
| GOBP_PROTEIN_LOCALIZATION_TO_CELL_JUNCTION | ECM and metastasis | -0.93695 | 0.58438 | 0.80271 | 1.39690 | 0.02133 | 0.06130 | 0.61855 | 0.98251 | 0.87060 |
| GOBP_GLYCOLYTIC_PROCESS_THROUGH_FRUCTOSE_6_PHOSPHATE | Metabolism and energy | 2.12228 | 0.00025 | 0.00710 | -1.09342 | 0.31950 | 0.41977 | -1.39931 | 0.09234 | 0.34480 |
| GOBP_REGULATION_OF_DNA_TEMPLATED_TRANSCRIPTION_INITIATION | Genetic and epigenetic information | 0.70483 | 0.95820 | 0.88692 | 0.75551 | 0.87087 | 0.71669 | 0.64552 | 0.96281 | 0.87060 |
| GOBP_PYRIMIDINE_NUCLEOSIDE_TRIPHOSPHATE_METABOLIC_PROCESS | Genetic and epigenetic information | 0.86581 | 0.64166 | 0.82751 | 0.57238 | 0.96180 | 0.72077 | 0.53157 | 0.97888 | 0.87060 |
| GOBP_PHOSPHATIDYLETHANOLAMINE_METABOLIC_PROCESS | Metabolism and energy | 0.95652 | 0.50782 | 0.77250 | -1.70256 | 0.01133 | 0.03786 | 0.62685 | 0.93250 | 0.87060 |
| GOBP_REGULATION_OF_OXIDATIVE_STRESS_INDUCED_INTRINSIC_APOPTOTIC_SIGNALING_PATHWAY | Cell death | -0.98758 | 0.48555 | 0.76444 | -1.04014 | 0.38549 | 0.47226 | -1.01537 | 0.41555 | 0.69168 |
| GOBP_REGULATION_OF_NUCLEOTIDE_METABOLIC_PROCESS | Genetic and epigenetic information | -0.71658 | 0.93721 | 0.88692 | -1.24249 | 0.06947 | 0.14537 | -0.93531 | 0.61352 | 0.79377 |
| GOBP_REGULATION_OF_POLYSACCHARIDE_METABOLIC_PROCESS | Metabolism and energy | 1.11558 | 0.28369 | 0.63136 | 1.09456 | 0.34512 | 0.44080 | 0.92858 | 0.59287 | 0.78511 |
| GOBP_NEGATIVE_REGULATION_OF_TYPE_I_INTERFERON_PRODUCTION | Immunity | -0.62797 | 0.94726 | 0.88692 | -1.32131 | 0.09154 | 0.17851 | -1.08957 | 0.30329 | 0.60124 |
| GOBP_DNA_DEALKYLATION | Genetic and epigenetic information | 0.78153 | 0.80582 | 0.88169 | 0.77843 | 0.80254 | 0.69400 | -1.09182 | 0.29701 | 0.59561 |
| GOBP_REGULATION_OF_DNA_REPLICATION | Genetic and epigenetic information | -0.51836 | 0.99992 | 0.88692 | 1.37778 | 0.01537 | 0.04786 | 0.52402 | 0.99942 | 0.87060 |
| GOBP_DOUBLE_STRAND_BREAK_REPAIR_VIA_NONHOMOLOGOUS_END_JOINING | Genetic and epigenetic information | 0.55603 | 0.99755 | 0.88692 | 0.80328 | 0.80566 | 0.69495 | -1.02085 | 0.40302 | 0.68350 |
| GOBP_REGULATION_OF_AUTOPHAGY_OF_MITOCHONDRION | Cell death | -0.62411 | 0.95187 | 0.88692 | -1.26315 | 0.12371 | 0.22019 | -1.27351 | 0.12865 | 0.40498 |
| GOBP_DIACYLGLYCEROL_METABOLIC_PROCESS | Metabolism and energy | 0.90021 | 0.59605 | 0.80695 | 0.76315 | 0.80738 | 0.69597 | 0.84798 | 0.69689 | 0.82593 |
| GOBP_RIBONUCLEOSIDE_DIPHOSPHATE_METABOLIC_PROCESS | Genetic and epigenetic information | 1.37635 | 0.03364 | 0.22821 | -1.25377 | 0.04964 | 0.11397 | 0.61455 | 0.98568 | 0.87060 |
| GOBP_LOW_DENSITY_LIPOPROTEIN_PARTICLE_CLEARANCE | Metabolism and energy | 0.86751 | 0.65033 | 0.83016 | -1.46122 | 0.04464 | 0.10534 | -0.87633 | 0.65427 | 0.81068 |
| GOBP_POSITIVE_REGULATION_OF_APOPTOTIC_SIGNALING_PATHWAY | Cell death | 0.85906 | 0.80112 | 0.87944 | -1.84592 | 0.00005 | 0.00070 | -1.10218 | 0.22017 | 0.51940 |
| GOBP_POSITIVE_REGULATION_OF_DNA_RECOMBINATION | Genetic and epigenetic information | -1.06672 | 0.35516 | 0.68797 | 0.99494 | 0.50182 | 0.55012 | -1.01674 | 0.41185 | 0.68967 |
| GOBP_ESTABLISHMENT_OF_MITOTIC_SPINDLE_LOCALIZATION | Cell cycle | 0.90664 | 0.60411 | 0.81250 | 1.21767 | 0.19130 | 0.29679 | -0.77084 | 0.85381 | 0.87060 |
| GOBP_CELL_CYCLE_G2_M_PHASE_TRANSITION | Cell cycle | 0.69959 | 0.99339 | 0.88692 | 1.57839 | 0.00019 | 0.00167 | -0.83443 | 0.90607 | 0.87060 |
| GOBP_DNA_METHYLATION_DEPENDENT_HETEROCHROMATIN_FORMATION | Genetic and epigenetic information | 0.66194 | 0.90208 | 0.88692 | 1.11217 | 0.33487 | 0.43273 | 0.45995 | 0.99397 | 0.87060 |
| GOBP_FATTY_ACID_BETA_OXIDATION | Metabolism and energy | -1.04705 | 0.38491 | 0.70674 | -0.79889 | 0.90390 | 0.72077 | 0.50100 | 0.99830 | 0.87060 |
| GOBP_NEGATIVE_REGULATION_OF_GENE_EXPRESSION_EPIGENETIC | Genetic and epigenetic information | 0.55009 | 0.99889 | 0.88692 | 1.24737 | 0.12108 | 0.21724 | 0.55173 | 0.99428 | 0.87060 |
| GOBP_NADH_REGENERATION | Metabolism and energy | 2.10994 | 0.00031 | 0.00850 | -1.16070 | 0.25258 | 0.35956 | -0.91346 | 0.56026 | 0.76846 |
| GOBP_REGULATION_OF_DNA_RECOMBINATION | Genetic and epigenetic information | -0.92626 | 0.61379 | 0.81628 | 1.11469 | 0.27196 | 0.37803 | -0.78470 | 0.94532 | 0.87060 |
| GOBP_REGULATION_OF_ENDOPLASMIC_RETICULUM_STRESS_INDUCED_INTRINSIC_APOPTOTIC_SIGNALING_PATHWAY | Cell death | -1.04555 | 0.40339 | 0.71899 | 0.72488 | 0.85589 | 0.71197 | -1.25448 | 0.14445 | 0.42961 |
| GOBP_REGULATION_OF_HISTONE_H3_K9_METHYLATION | Genetic and epigenetic information | -0.62930 | 0.93457 | 0.88692 | 0.98004 | 0.51458 | 0.55791 | -1.24284 | 0.16363 | 0.45237 |
| GOBP_NEGATIVE_REGULATION_OF_DNA_BINDING_TRANSCRIPTION_FACTOR_ACTIVITY | Genetic and epigenetic information | 1.00715 | 0.42824 | 0.73270 | 0.79913 | 0.88401 | 0.72038 | 0.82052 | 0.84610 | 0.87060 |
| GOBP_PURINE_NUCLEOSIDE_MONOPHOSPHATE_BIOSYNTHETIC_PROCESS | Genetic and epigenetic information | 1.20461 | 0.22972 | 0.58924 | -0.74891 | 0.83495 | 0.70519 | -1.27876 | 0.14970 | 0.43518 |
| GOBP_NUCLEOLUS_ORGANIZATION | Cell cycle | -0.72380 | 0.82926 | 0.88692 | 1.10282 | 0.35092 | 0.44525 | -1.43385 | 0.08357 | 0.32864 |
| GOBP_NUCLEOSIDE_BISPHOSPHATE_METABOLIC_PROCESS | Genetic and epigenetic information | -0.87565 | 0.71966 | 0.85522 | 0.82036 | 0.82913 | 0.70286 | 1.13133 | 0.24623 | 0.54363 |
| GOBP_PURINE_CONTAINING_COMPOUND_TRANSMEMBRANE_TRANSPORT | Genetic and epigenetic information | -0.93733 | 0.55784 | 0.79537 | -1.03580 | 0.39180 | 0.47692 | -0.94652 | 0.52770 | 0.75322 |
| GOBP_DNA_STRAND_ELONGATION_INVOLVED_IN_DNA_REPLICATION | Genetic and epigenetic information | 0.71714 | 0.82073 | 0.88645 | 1.63778 | 0.00882 | 0.03149 | -0.80276 | 0.73328 | 0.83705 |
| GOBP_CARBOHYDRATE_DERIVATIVE_TRANSPORT | Metabolism and energy | -0.87361 | 0.69650 | 0.84639 | -1.69692 | 0.00079 | 0.00486 | -0.97218 | 0.51323 | 0.74385 |
| GOBP_REGULATION_OF_CELL_DIVISION | Cell cycle | 0.93029 | 0.66184 | 0.83352 | 1.19239 | 0.11504 | 0.20947 | 0.77358 | 0.90948 | 0.87060 |
| GOBP_LONG_CHAIN_FATTY_ACID_METABOLIC_PROCESS | Metabolism and energy | -1.13188 | 0.24530 | 0.60067 | -1.14438 | 0.15263 | 0.25526 | 1.12797 | 0.26097 | 0.56202 |
| GOBP_NUCLEAR_TRANSCRIBED_MRNA_CATABOLIC_PROCESS_EXONUCLEOLYTIC | Genetic and epigenetic information | -0.59887 | 0.94551 | 0.88692 | 0.90618 | 0.61511 | 0.61363 | 0.66326 | 0.89791 | 0.87060 |
| GOBP_POSITIVE_REGULATION_OF_MITOTIC_NUCLEAR_DIVISION | Cell cycle | -1.10255 | 0.31792 | 0.65965 | 1.14577 | 0.27247 | 0.37826 | 0.77203 | 0.81845 | 0.86666 |
| GOBP_NEGATIVE_REGULATION_OF_DNA_REPLICATION | Genetic and epigenetic information | 0.60041 | 0.96765 | 0.88692 | 1.19949 | 0.21907 | 0.32652 | 0.65612 | 0.92057 | 0.87060 |
| GOBP_REGULATION_OF_CENTRIOLE_REPLICATION | Cell cycle | -0.64372 | 0.92026 | 0.88692 | 1.42078 | 0.05890 | 0.12912 | 0.65557 | 0.90879 | 0.87060 |
| GOBP_POSITIVE_REGULATION_OF_DNA_BIOSYNTHETIC_PROCESS | Genetic and epigenetic information | -0.90659 | 0.63073 | 0.82455 | 0.90492 | 0.65825 | 0.63574 | -0.85735 | 0.77166 | 0.85184 |
| GOBP_HOMOLOGOUS_RECOMBINATION | Genetic and epigenetic information | -0.99512 | 0.47169 | 0.76062 | 1.59756 | 0.00283 | 0.01300 | -0.85074 | 0.77350 | 0.85281 |
| GOBP_REGULATION_OF_ALTERNATIVE_MRNA_SPLICING_VIA_SPLICEOSOME | Genetic and epigenetic information | -0.83466 | 0.74485 | 0.86231 | 1.35496 | 0.06019 | 0.13105 | -1.23093 | 0.12912 | 0.40562 |
| GOBP_REGULATION_OF_CELL_CYCLE_G2_M_PHASE_TRANSITION | Cell cycle | -0.57722 | 0.99786 | 0.88692 | 1.59484 | 0.00064 | 0.00416 | -0.90298 | 0.71522 | 0.83151 |
| GOBP_PHOSPHATIDYLSERINE_METABOLIC_PROCESS | Metabolism and energy | 0.75178 | 0.81614 | 0.88529 | -1.57784 | 0.02342 | 0.06579 | 1.16294 | 0.26880 | 0.56879 |
| GOBP_REGULATION_OF_CHOLESTEROL_METABOLIC_PROCESS | Metabolism and energy | -1.33213 | 0.10540 | 0.41009 | -1.16988 | 0.20099 | 0.30711 | -0.92043 | 0.58558 | 0.78118 |
| GOBP_GMP_METABOLIC_PROCESS | Genetic and epigenetic information | 0.85925 | 0.66104 | 0.83351 | -0.99571 | 0.45607 | 0.52229 | -1.26724 | 0.14250 | 0.42598 |
| GOBP_NEGATIVE_REGULATION_OF_MITOTIC_CELL_CYCLE_PHASE_TRANSITION | Cell cycle | -0.85680 | 0.78369 | 0.87529 | 1.47533 | 0.00121 | 0.00674 | -0.89793 | 0.78972 | 0.85708 |
| GOBP_GLYCEROPHOSPHOLIPID_BIOSYNTHETIC_PROCESS | Metabolism and energy | -0.93690 | 0.61601 | 0.81645 | 0.45852 | 1.00000 | 0.72077 | 0.59646 | 0.99785 | 0.87060 |
| GOBP_POSITIVE_REGULATION_OF_INTRINSIC_APOPTOTIC_SIGNALING_PATHWAY | Cell death | -0.75545 | 0.85746 | 0.88692 | -1.72110 | 0.00195 | 0.00970 | -1.37570 | 0.05657 | 0.26871 |
| GOBP_POSITIVE_REGULATION_OF_HISTONE_H3_K4_METHYLATION | Genetic and epigenetic information | -0.71616 | 0.86266 | 0.88692 | 1.22160 | 0.20051 | 0.30664 | -1.22900 | 0.16731 | 0.45753 |
| GOBP_NUCLEOSIDE_MONOPHOSPHATE_CATABOLIC_PROCESS | Genetic and epigenetic information | 1.04535 | 0.39047 | 0.71025 | -1.17711 | 0.22514 | 0.33307 | -0.73208 | 0.84662 | 0.87060 |
| GOBP_POSITIVE_REGULATION_OF_HISTONE_METHYLATION | Genetic and epigenetic information | -0.84426 | 0.72122 | 0.85522 | 1.11195 | 0.31495 | 0.41638 | -1.34017 | 0.07323 | 0.30755 |
| GOBP_FACULTATIVE_HETEROCHROMATIN_FORMATION | Cell cycle | 0.71509 | 0.87416 | 0.88692 | 1.21269 | 0.20720 | 0.31372 | -0.77508 | 0.82387 | 0.86849 |
| GOBP_NEGATIVE_REGULATION_OF_INTRINSIC_APOPTOTIC_SIGNALING_PATHWAY_IN_RESPONSE_TO_DNA_DAMAGE | Cell death | 1.45529 | 0.06140 | 0.31312 | -1.37560 | 0.06946 | 0.14537 | 1.12607 | 0.30993 | 0.60854 |
| GOBP_INTRINSIC_APOPTOTIC_SIGNALING_PATHWAY_IN_RESPONSE_TO_OXIDATIVE_STRESS | Cell death | 0.81847 | 0.76335 | 0.86736 | -1.13544 | 0.23227 | 0.34003 | -0.95783 | 0.51844 | 0.74642 |
| GOBP_RESPONSE_TO_AMINO_ACID_STARVATION | Metabolism and energy | 0.73964 | 0.89038 | 0.88692 | 0.78638 | 0.80906 | 0.69649 | -0.75631 | 0.89455 | 0.87060 |
| GOBP_REGULATION_OF_CARBOHYDRATE_BIOSYNTHETIC_PROCESS | Metabolism and energy | 0.96723 | 0.52165 | 0.77916 | -0.83534 | 0.87653 | 0.71849 | 0.52034 | 0.99848 | 0.87060 |
| GOBP_DNA_SYNTHESIS_INVOLVED_IN_DNA_REPAIR | Genetic and epigenetic information | -0.60304 | 0.96857 | 0.88692 | 1.37152 | 0.06719 | 0.14233 | -0.60509 | 0.98296 | 0.87060 |
| GOBP_EXTRINSIC_APOPTOTIC_SIGNALING_PATHWAY | Cell death | 1.21045 | 0.06942 | 0.33446 | -1.65896 | 0.00022 | 0.00188 | 0.97672 | 0.55756 | 0.76763 |
| GOBP_CELLULAR_RESPIRATION | Metabolism and energy | 0.76910 | 0.98198 | 0.88692 | -0.81642 | 0.97068 | 0.72077 | -1.70818 | 0.00013 | 0.00649 |
| GOBP_ACETYL_COA_METABOLIC_PROCESS | Metabolism and energy | -1.46563 | 0.04380 | 0.26152 | 0.74724 | 0.83596 | 0.70531 | -0.84194 | 0.72818 | 0.83643 |
| GOBP_CELL_CYCLE_G1_S_PHASE_TRANSITION | Cell cycle | -0.87659 | 0.76996 | 0.87067 | 0.96421 | 0.60288 | 0.60771 | -1.07856 | 0.21479 | 0.51404 |
| GOBP_HETEROCHROMATIN_ORGANIZATION | Cell cycle | 0.75720 | 0.91752 | 0.88692 | 1.19665 | 0.17635 | 0.28180 | 0.58719 | 0.98767 | 0.87060 |
| GOBP_POSITIVE_REGULATION_OF_MITOTIC_CELL_CYCLE_PHASE_TRANSITION | Cell cycle | -0.78974 | 0.84692 | 0.88692 | 1.32319 | 0.05574 | 0.12391 | -0.81742 | 0.87029 | 0.87060 |
| GOBP_TUMOR_NECROSIS_FACTOR_MEDIATED_SIGNALING_PATHWAY | Immunity | -0.84506 | 0.76328 | 0.86736 | -1.05179 | 0.33194 | 0.43013 | 0.79746 | 0.83604 | 0.87060 |
| GOBP_NUCLEOBASE_BIOSYNTHETIC_PROCESS | Genetic and epigenetic information | 1.04259 | 0.39372 | 0.71215 | 0.74809 | 0.81497 | 0.69797 | -0.91960 | 0.56266 | 0.76847 |
| GOBP_RIBONUCLEOSIDE_MONOPHOSPHATE_METABOLIC_PROCESS | Genetic and epigenetic information | 0.80608 | 0.80965 | 0.88346 | -1.05247 | 0.35093 | 0.44525 | -1.13478 | 0.22603 | 0.52430 |
| GOBP_ADHERENS_JUNCTION_ORGANIZATION | ECM and metastasis | 1.01988 | 0.41136 | 0.72308 | 0.76581 | 0.83596 | 0.70531 | 1.27959 | 0.12193 | 0.39401 |
| GOBP_REGULATION_OF_NUCLEAR_TRANSCRIBED_MRNA_CATABOLIC_PROCESS_DEADENYLATION_DEPENDENT_DECAY | Genetic and epigenetic information | -1.19278 | 0.23693 | 0.59203 | 0.86103 | 0.67978 | 0.64364 | -1.66120 | 0.02203 | 0.15817 |
| GOBP_SERINE_FAMILY_AMINO_ACID_BIOSYNTHETIC_PROCESS | Metabolism and energy | 1.00043 | 0.44621 | 0.74495 | 0.98977 | 0.50001 | 0.54906 | 0.86412 | 0.66643 | 0.81364 |
| GOBP_INTERSTRAND_CROSS_LINK_REPAIR | Genetic and epigenetic information | -0.58393 | 0.97795 | 0.88692 | 1.47464 | 0.02543 | 0.06990 | 0.87692 | 0.67122 | 0.81597 |
| GOBP_DNA_DAMAGE_RESPONSE_SIGNAL_TRANSDUCTION_BY_P53_CLASS_MEDIATOR | Genetic and epigenetic information | -0.73057 | 0.90642 | 0.88692 | 0.69359 | 0.92620 | 0.72077 | -0.77215 | 0.90997 | 0.87060 |
| GOBP_LIPOPROTEIN_METABOLIC_PROCESS | Metabolism and energy | -0.89821 | 0.67888 | 0.84067 | 0.73410 | 0.93081 | 0.72077 | -0.78539 | 0.95427 | 0.87060 |
| GOBP_GENE_SILENCING_BY_RNA | Genetic and epigenetic information | -0.36616 | 1.00000 | 0.88692 | 0.39913 | 1.00000 | 0.72077 | -0.98226 | 0.50404 | 0.73970 |
| GOBP_HISTONE_H3_K9_MODIFICATION | Genetic and epigenetic information | -1.01948 | 0.43404 | 0.73668 | 1.09871 | 0.33482 | 0.43273 | 0.73496 | 0.86780 | 0.87060 |
| GOBP_NEGATIVE_REGULATION_OF_TUMOR_NECROSIS_FACTOR_MEDIATED_SIGNALING_PATHWAY | Immunity | 0.80599 | 0.73374 | 0.86010 | 0.64139 | 0.92119 | 0.72077 | 1.30534 | 0.13218 | 0.41088 |
| GOBP_GLYCEROLIPID_BIOSYNTHETIC_PROCESS | Metabolism and energy | -0.96270 | 0.55979 | 0.79590 | -0.79109 | 0.99356 | 0.72077 | 0.56431 | 0.99965 | 0.87060 |
| GOBP_FATTY_ACID_DERIVATIVE_METABOLIC_PROCESS | Metabolism and energy | -1.23124 | 0.15038 | 0.48104 | 0.78518 | 0.83436 | 0.70491 | 0.86660 | 0.71348 | 0.83074 |
| GOBP_REGULATION_OF_PROGRAMMED_NECROTIC_CELL_DEATH | Cell death | -1.26011 | 0.16865 | 0.51113 | -1.58876 | 0.02034 | 0.05925 | 0.78304 | 0.78218 | 0.85564 |
| GOBP_INTRINSIC_APOPTOTIC_SIGNALING_PATHWAY_IN_RESPONSE_TO_DNA_DAMAGE_BY_P53_CLASS_MEDIATOR | Cell death | 0.87057 | 0.67544 | 0.83911 | -1.11408 | 0.26062 | 0.36725 | 0.65170 | 0.93661 | 0.87060 |
| GOBP_PROTEIN_LOCALIZATION_TO_CELL_CELL_JUNCTION | ECM and metastasis | -1.07972 | 0.36800 | 0.69702 | -1.31036 | 0.12213 | 0.21828 | -0.92383 | 0.55740 | 0.76763 |
| GOBP_POSITIVE_REGULATION_OF_MITOTIC_CELL_CYCLE | Cell cycle | -0.63723 | 0.99107 | 0.88692 | 1.15530 | 0.20687 | 0.31335 | -1.03514 | 0.36472 | 0.65181 |
| GOBP_NADPH_REGENERATION | Metabolism and energy | 0.95829 | 0.50446 | 0.77250 | -1.33356 | 0.10396 | 0.19536 | -0.92135 | 0.56585 | 0.77095 |
| GOBP_PYRUVATE_METABOLIC_PROCESS | Metabolism and energy | 1.43857 | 0.01985 | 0.16326 | 0.72715 | 0.91872 | 0.72077 | -0.92767 | 0.64453 | 0.80665 |
| GOBP_PHOSPHOLIPID_BIOSYNTHETIC_PROCESS | Metabolism and energy | -0.90754 | 0.69855 | 0.84746 | -0.78861 | 0.99347 | 0.72077 | 0.55003 | 0.99984 | 0.87060 |
| GOBP_FATTY_ACYL_COA_BIOSYNTHETIC_PROCESS | Metabolism and energy | -0.52265 | 0.98417 | 0.88692 | 1.01919 | 0.45867 | 0.52339 | 0.58121 | 0.96032 | 0.87060 |
| GOBP_HISTONE_H4_K16_ACETYLATION | Genetic and epigenetic information | -0.80100 | 0.75439 | 0.86446 | 1.07814 | 0.37623 | 0.46555 | -1.05470 | 0.35736 | 0.64447 |
| GOBP_PURINE_NUCLEOSIDE_MONOPHOSPHATE_METABOLIC_PROCESS | Genetic and epigenetic information | 0.92380 | 0.57459 | 0.79849 | -0.96448 | 0.52270 | 0.56295 | -1.38706 | 0.06696 | 0.29394 |
| GOBP_REGULATION_OF_DNA_METHYLATION | Genetic and epigenetic information | 0.65816 | 0.91463 | 0.88692 | 0.66288 | 0.90561 | 0.72077 | -0.87678 | 0.64652 | 0.80711 |
| GOBP_CELLULAR_RESPONSE_TO_FATTY_ACID | Metabolism and energy | 1.05992 | 0.36094 | 0.69224 | -1.31958 | 0.08982 | 0.17570 | 1.45478 | 0.03881 | 0.21754 |
| GOBP_NEGATIVE_REGULATION_OF_NECROTIC_CELL_DEATH | Cell death | -1.12759 | 0.31046 | 0.65424 | 0.63953 | 0.91979 | 0.72077 | 0.98534 | 0.50252 | 0.73875 |
| GOBP_IMP_METABOLIC_PROCESS | Genetic and epigenetic information | 1.31884 | 0.15542 | 0.48853 | -0.87987 | 0.62781 | 0.61969 | -0.81457 | 0.71989 | 0.83336 |
| GOBP_CELLULAR_MODIFIED_AMINO_ACID_BIOSYNTHETIC_PROCESS | Metabolism and energy | 1.64023 | 0.01321 | 0.12614 | -1.14564 | 0.21955 | 0.32695 | 0.71191 | 0.88372 | 0.87060 |
| GOBP_POSITIVE_REGULATION_OF_TRANSCRIPTION_FROM_RNA_POLYMERASE_II_PROMOTER_IN_RESPONSE_TO_STRESS | Genetic and epigenetic information | -1.01062 | 0.45702 | 0.75193 | -1.16027 | 0.24139 | 0.34948 | 0.71073 | 0.85301 | 0.87060 |
| GOBP_CARBOHYDRATE_BIOSYNTHETIC_PROCESS | Metabolism and energy | 1.21666 | 0.06867 | 0.33207 | -1.08874 | 0.18509 | 0.29012 | 0.63811 | 0.99256 | 0.87060 |
| GOBP_PURINE_CONTAINING_COMPOUND_CATABOLIC_PROCESS | Genetic and epigenetic information | -1.00066 | 0.46283 | 0.75485 | 0.83190 | 0.75510 | 0.67631 | 0.79267 | 0.80415 | 0.86190 |
| GOBP_UNSATURATED_FATTY_ACID_METABOLIC_PROCESS | Metabolism and energy | -1.13371 | 0.24229 | 0.59767 | -1.22711 | 0.06669 | 0.14167 | 0.91637 | 0.65020 | 0.80851 |
| GOBP_ATTACHMENT_OF_SPINDLE_MICROTUBULES_TO_KINETOCHORE | Cell cycle | -0.51378 | 0.99434 | 0.88692 | 1.82640 | 0.00015 | 0.00144 | -1.17088 | 0.19849 | 0.49197 |
| GOBP_LIPID_OXIDATION | Metabolism and energy | -1.26705 | 0.09504 | 0.39193 | -0.98817 | 0.50457 | 0.55102 | 0.51031 | 0.99941 | 0.87060 |
| GOBP_MITOTIC_G2_M_TRANSITION_CHECKPOINT | Cell cycle | 0.88798 | 0.65325 | 0.83176 | 1.42179 | 0.03586 | 0.08987 | -0.94587 | 0.55086 | 0.76478 |
| GOBP_REGULATION_OF_CHROMATIN_ORGANIZATION | Cell cycle | -0.71890 | 0.88254 | 0.88692 | 1.31707 | 0.09924 | 0.18880 | 0.66384 | 0.92543 | 0.87060 |
| GOBP_REGULATION_OF_DNA_TEMPLATED_TRANSCRIPTION_IN_RESPONSE_TO_STRESS | Genetic and epigenetic information | -1.61765 | 0.00961 | 0.10558 | -1.29386 | 0.08863 | 0.17404 | -1.51509 | 0.02984 | 0.18929 |
| GOBP_MACROAUTOPHAGY | Cell death | -0.71218 | 0.99376 | 0.88692 | -0.76491 | 0.99792 | 0.72077 | -1.25870 | 0.00865 | 0.09422 |
| GOBP_REGULATION_OF_SISTER_CHROMATID_COHESION | Cell cycle | -0.56313 | 0.96583 | 0.88692 | 1.46856 | 0.04167 | 0.10015 | -1.43491 | 0.07400 | 0.30892 |
| GOBP_REGULATION_OF_EXTRINSIC_APOPTOTIC_SIGNALING_PATHWAY | Cell death | 1.40270 | 0.01530 | 0.13878 | -1.62646 | 0.00022 | 0.00188 | 1.00224 | 0.49107 | 0.73239 |
| GOBP_RNA_PHOSPHODIESTER_BOND_HYDROLYSIS_EXONUCLEOLYTIC | Genetic and epigenetic information | -0.71807 | 0.88638 | 0.88692 | 0.79864 | 0.78627 | 0.68800 | -0.91991 | 0.59625 | 0.78553 |
| GOBP_REGULATION_OF_CELL_CYCLE_G1_S_PHASE_TRANSITION | Cell cycle | -1.01817 | 0.42660 | 0.73267 | 0.95515 | 0.61049 | 0.61174 | -0.95414 | 0.61975 | 0.79693 |
| GOBP_POSITIVE_REGULATION_OF_NUCLEAR_DIVISION | Cell cycle | -1.01899 | 0.43296 | 0.73583 | 1.34018 | 0.06784 | 0.14305 | 0.99216 | 0.49821 | 0.73734 |
| GOBP_CELLULAR_CARBOHYDRATE_BIOSYNTHETIC_PROCESS | Metabolism and energy | 1.15056 | 0.20734 | 0.56819 | -0.72444 | 0.96975 | 0.72077 | 0.93903 | 0.59557 | 0.78511 |
| GOBP_NEGATIVE_REGULATION_OF_MIRNA_METABOLIC_PROCESS | Genetic and epigenetic information | -0.90064 | 0.60637 | 0.81299 | -1.80549 | 0.00810 | 0.02948 | 0.78302 | 0.77001 | 0.85114 |
| GOBP_MITOCHONDRIAL_ELECTRON_TRANSPORT_CYTOCHROME_C_TO_OXYGEN | Metabolism and energy | 1.14043 | 0.28985 | 0.63799 | -0.95092 | 0.52061 | 0.56137 | 1.18182 | 0.25511 | 0.55504 |
| GOBP_MITOTIC_G2_DNA_DAMAGE_CHECKPOINT_SIGNALING | Cell cycle | 0.48949 | 0.99628 | 0.88692 | 1.56740 | 0.01095 | 0.03704 | -0.91673 | 0.59159 | 0.78511 |
| GOBP_GLUCOSE_6_PHOSPHATE_METABOLIC_PROCESS | Metabolism and energy | 0.54732 | 0.98148 | 0.88692 | -1.08675 | 0.31539 | 0.41686 | -0.93317 | 0.55271 | 0.76552 |
| GOBP_MIRNA_METABOLIC_PROCESS | Genetic and epigenetic information | -1.41176 | 0.03643 | 0.23773 | -1.40342 | 0.01755 | 0.05306 | -1.41023 | 0.02766 | 0.18033 |
| GOBP_HISTONE_H3_K9_METHYLATION | Genetic and epigenetic information | -0.68455 | 0.90591 | 0.88692 | 1.08267 | 0.36631 | 0.45756 | -0.73836 | 0.88080 | 0.87060 |
| GOBP_PYRIMIDINE_CONTAINING_COMPOUND_BIOSYNTHETIC_PROCESS | Genetic and epigenetic information | -0.82517 | 0.73466 | 0.86010 | -0.64283 | 0.97123 | 0.72077 | 0.61875 | 0.95141 | 0.87060 |
| GOBP_CELL_JUNCTION_MAINTENANCE | ECM and metastasis | 0.80294 | 0.79018 | 0.87715 | 1.08512 | 0.35793 | 0.45102 | 1.34061 | 0.08508 | 0.33202 |
| GOBP_LIPID_DROPLET_ORGANIZATION | Metabolism and energy | 0.94643 | 0.52991 | 0.78354 | -1.21218 | 0.16700 | 0.27226 | -0.58034 | 0.98468 | 0.87060 |
| GOBP_DEOXYRIBONUCLEOTIDE_METABOLIC_PROCESS | Genetic and epigenetic information | -0.92562 | 0.58393 | 0.80271 | 0.76859 | 0.82579 | 0.70167 | -0.98596 | 0.46746 | 0.72135 |
| GOBP_ENERGY_DERIVATION_BY_OXIDATION_OF_ORGANIC_COMPOUNDS | Metabolism and energy | 1.19940 | 0.05664 | 0.30022 | -0.97552 | 0.59533 | 0.60447 | -1.12790 | 0.09703 | 0.35126 |
| GOBP_NEGATIVE_REGULATION_OF_APOPTOTIC_SIGNALING_PATHWAY | Cell death | 1.27774 | 0.03258 | 0.22462 | -1.20923 | 0.03424 | 0.08682 | 0.87600 | 0.78081 | 0.85527 |
| GOBP_CELLULAR_LIPID_CATABOLIC_PROCESS | Metabolism and energy | -1.30777 | 0.03762 | 0.24004 | -0.99268 | 0.50012 | 0.54906 | 0.89228 | 0.74896 | 0.84250 |
| GOBP_AUTOPHAGOSOME_MATURATION | Cell death | -0.82724 | 0.76338 | 0.86736 | -0.94272 | 0.59616 | 0.60457 | -1.40078 | 0.03979 | 0.22125 |
| GOBP_CELLULAR_RESPONSE_TO_GLUCOSE_STARVATION | Metabolism and energy | 1.39213 | 0.06306 | 0.31878 | 0.64543 | 0.94489 | 0.72077 | -1.11304 | 0.25893 | 0.55900 |
| GOBP_CELLULAR_GLUCOSE_HOMEOSTASIS | Metabolism and energy | -0.89803 | 0.68271 | 0.84172 | 0.86678 | 0.77091 | 0.68330 | 0.83324 | 0.81285 | 0.86488 |
| GOBP_CARBOHYDRATE_HOMEOSTASIS | Metabolism and energy | 1.11720 | 0.16927 | 0.51234 | 0.78791 | 0.92127 | 0.72077 | 0.81088 | 0.88407 | 0.87060 |
| GOBP_LONG_CHAIN_FATTY_ACID_BIOSYNTHETIC_PROCESS | Metabolism and energy | -1.12792 | 0.30441 | 0.64873 | 0.47690 | 0.99248 | 0.72077 | 0.84339 | 0.70174 | 0.82740 |
| GOBP_PHOSPHATIDYLCHOLINE_BIOSYNTHETIC_PROCESS | Metabolism and energy | 0.67587 | 0.91331 | 0.88692 | 0.59894 | 0.95518 | 0.72077 | 1.01357 | 0.46290 | 0.71875 |
| GOBP_REGULATION_OF_TRANSCRIPTION_REGULATORY_REGION_DNA_BINDING | Genetic and epigenetic information | -0.88594 | 0.65270 | 0.83163 | 1.16476 | 0.24203 | 0.35020 | 1.12152 | 0.29999 | 0.59931 |
| GOBP_MRNA_TRANSCRIPTION | Genetic and epigenetic information | -0.68484 | 0.92759 | 0.88692 | -1.48151 | 0.01947 | 0.05734 | -1.17721 | 0.18609 | 0.48085 |
| GOBP_POSITIVE_REGULATION_OF_CELL_DIVISION | Cell cycle | 1.12530 | 0.22933 | 0.58924 | 1.06218 | 0.37924 | 0.46770 | 0.86841 | 0.72333 | 0.83517 |
| GOBP_POSITIVE_REGULATION_OF_CELL_CYCLE_G1_S_PHASE_TRANSITION | Cell cycle | -0.97088 | 0.51172 | 0.77435 | 0.90769 | 0.64274 | 0.62838 | 0.74167 | 0.86731 | 0.87060 |
| GOBP_MITOTIC_RECOMBINATION | Cell cycle | 0.54555 | 0.98147 | 0.88692 | 1.37861 | 0.07598 | 0.15472 | -0.87499 | 0.65303 | 0.80997 |
| GOBP_ACTIVATION_OF_CYSTEINE_TYPE_ENDOPEPTIDASE_ACTIVITY_INVOLVED_IN_APOPTOTIC_PROCESS | Cell death | 1.05419 | 0.34833 | 0.68236 | -1.91193 | 0.00009 | 0.00102 | -1.83474 | 0.00067 | 0.01805 |
| GOBP_POSITIVE_REGULATION_OF_MIRNA_METABOLIC_PROCESS | Genetic and epigenetic information | -1.61692 | 0.00789 | 0.09373 | -1.57145 | 0.00809 | 0.02948 | -1.54313 | 0.01970 | 0.14723 |
| GOBP_REGULATION_OF_LIPID_BIOSYNTHETIC_PROCESS | Metabolism and energy | -1.44232 | 0.01012 | 0.10889 | -1.03685 | 0.34236 | 0.43897 | -1.06829 | 0.26831 | 0.56879 |
| GOBP_LONG_CHAIN_FATTY_ACYL_COA_METABOLIC_PROCESS | Metabolism and energy | 0.56605 | 0.97330 | 0.88692 | 0.98040 | 0.51415 | 0.55782 | 0.55401 | 0.97341 | 0.87060 |
| GOBP_POSITIVE_REGULATION_OF_DNA_REPLICATION | Genetic and epigenetic information | 0.66151 | 0.94747 | 0.88692 | 1.20698 | 0.20025 | 0.30643 | -0.63786 | 0.97295 | 0.87060 |
| GOBP_MISMATCH_REPAIR | Genetic and epigenetic information | -0.62191 | 0.94942 | 0.88692 | 1.43408 | 0.04573 | 0.10735 | 0.63244 | 0.93653 | 0.87060 |
| GOBP_SERINE_FAMILY_AMINO_ACID_METABOLIC_PROCESS | Metabolism and energy | 0.75217 | 0.84844 | 0.88692 | 0.83891 | 0.72555 | 0.66344 | -0.90231 | 0.62187 | 0.79729 |
| GOBP_NEGATIVE_REGULATION_OF_FATTY_ACID_BIOSYNTHETIC_PROCESS | Metabolism and energy | -1.32170 | 0.13548 | 0.45889 | 0.95264 | 0.55069 | 0.58027 | 0.67962 | 0.87875 | 0.87060 |
| GOBP_POSITIVE_REGULATION_OF_STEROID_BIOSYNTHETIC_PROCESS | Metabolism and energy | -0.96471 | 0.51824 | 0.77803 | 0.74530 | 0.82095 | 0.70000 | 0.68325 | 0.88322 | 0.87060 |
| GOBP_TRIGLYCERIDE_BIOSYNTHETIC_PROCESS | Metabolism and energy | -1.15698 | 0.25844 | 0.61103 | -1.37530 | 0.05972 | 0.13046 | -1.32543 | 0.09639 | 0.35071 |
| GOBP_NEGATIVE_REGULATION_OF_DNA_RECOMBINATION | Genetic and epigenetic information | -0.55512 | 0.98072 | 0.88692 | 1.36211 | 0.07995 | 0.16064 | 0.67304 | 0.90668 | 0.87060 |
| GOBP_PROTEIN_MONOUBIQUITINATION | Immunity | -0.37041 | 1.00000 | 0.88692 | 1.29363 | 0.07906 | 0.15935 | -0.81247 | 0.87074 | 0.87060 |
| GOBP_POSITIVE_REGULATION_OF_FATTY_ACID_TRANSPORT | Metabolism and energy | -0.96622 | 0.51619 | 0.77648 | -1.04822 | 0.37416 | 0.46376 | 1.22883 | 0.19767 | 0.49137 |
| GOBP_STEROID_BIOSYNTHETIC_PROCESS | Metabolism and energy | -1.37834 | 0.02212 | 0.17620 | 0.50186 | 0.99986 | 0.72077 | -1.16309 | 0.10489 | 0.36518 |
| GOBP_REGULATION_OF_MIRNA_METABOLIC_PROCESS | Genetic and epigenetic information | -1.47710 | 0.02316 | 0.18082 | -1.63081 | 0.00224 | 0.01079 | -1.31951 | 0.06444 | 0.28668 |
| GOBP_PURINE_RIBONUCLEOSIDE_METABOLIC_PROCESS | Genetic and epigenetic information | -0.87692 | 0.64171 | 0.82751 | -0.92595 | 0.56807 | 0.58990 | -1.11173 | 0.29147 | 0.59212 |
| GOBP_INTRACELLULAR_LIPID_TRANSPORT | Metabolism and energy | -1.31213 | 0.11119 | 0.41658 | 0.64455 | 0.93983 | 0.72077 | 1.01876 | 0.45404 | 0.71382 |
| GOBP_NADH_METABOLIC_PROCESS | Metabolism and energy | 1.91257 | 0.00148 | 0.02936 | -1.61249 | 0.01201 | 0.03970 | 0.74148 | 0.84388 | 0.87060 |
| GOBP_NEGATIVE_REGULATION_OF_TRANSCRIPTION_REGULATORY_REGION_DNA_BINDING | Genetic and epigenetic information | -1.08496 | 0.36338 | 0.69433 | 1.32078 | 0.12184 | 0.21815 | 1.32607 | 0.12142 | 0.39333 |
| GOBP_POLYSACCHARIDE_BIOSYNTHETIC_PROCESS | Metabolism and energy | 1.11492 | 0.25658 | 0.60970 | 0.60816 | 0.97692 | 0.72077 | 1.08125 | 0.35052 | 0.63933 |
| GOBP_RESPONSE_TO_TYPE_I_INTERFERON | Immunity | 1.41541 | 0.03553 | 0.23544 | -1.37554 | 0.02411 | 0.06719 | 0.89470 | 0.67081 | 0.81579 |
| GOBP_SISTER_CHROMATID_COHESION | Cell cycle | -0.40092 | 0.99994 | 0.88692 | 1.62104 | 0.00262 | 0.01224 | -0.99666 | 0.44858 | 0.70977 |
| GOBP_REGULATION_OF_ATP_BIOSYNTHETIC_PROCESS | Metabolism and energy | 0.93959 | 0.52380 | 0.78003 | -1.56738 | 0.03852 | 0.09424 | -0.99058 | 0.45041 | 0.71069 |
| GOBP_GLYCOLIPID_BIOSYNTHETIC_PROCESS | Metabolism and energy | 0.70975 | 0.95053 | 0.88692 | 0.90560 | 0.65658 | 0.63492 | 0.69466 | 0.92589 | 0.87060 |
| GOBP_DNA_TEMPLATED_TRANSCRIPTION_TERMINATION | Genetic and epigenetic information | 0.54961 | 0.97178 | 0.88692 | -0.56537 | 0.98329 | 0.72077 | -1.01889 | 0.41082 | 0.68905 |
| GOBP_PYRIMIDINE_CONTAINING_COMPOUND_METABOLIC_PROCESS | Genetic and epigenetic information | -0.85301 | 0.73664 | 0.86059 | 0.73950 | 0.89484 | 0.72077 | -1.17555 | 0.15273 | 0.43865 |
| GOBP_POSITIVE_REGULATION_OF_TRANSLATIONAL_INITIATION | Metabolism and energy | -0.59513 | 0.95833 | 0.88692 | -0.95675 | 0.52367 | 0.56342 | -0.63674 | 0.95366 | 0.87060 |
| GOBP_NUCLEOSOME_ASSEMBLY | Cell cycle | -0.90945 | 0.60754 | 0.81299 | 1.40011 | 0.05114 | 0.11615 | -1.36652 | 0.07347 | 0.30775 |
| GOBP_CELLULAR_AMINO_ACID_METABOLIC_PROCESS | Metabolism and energy | -1.06389 | 0.32170 | 0.66156 | 0.80559 | 0.91627 | 0.72077 | 0.61628 | 0.99779 | 0.87060 |
| GOBP_POSITIVE_REGULATION_OF_TRIGLYCERIDE_METABOLIC_PROCESS | Metabolism and energy | -1.67635 | 0.00843 | 0.09674 | -1.06540 | 0.34960 | 0.44432 | -1.54890 | 0.03943 | 0.22047 |
| GOBP_PURINE_NUCLEOBASE_METABOLIC_PROCESS | Genetic and epigenetic information | 0.80849 | 0.71970 | 0.85522 | 0.94576 | 0.56119 | 0.58618 | -0.90141 | 0.59453 | 0.78511 |
| GOBP_PHOSPHOLIPID_CATABOLIC_PROCESS | Metabolism and energy | -1.52297 | 0.01889 | 0.15802 | -0.93235 | 0.61113 | 0.61184 | 0.94790 | 0.57018 | 0.77238 |
| GOBP_MITOTIC_G1_S_TRANSITION_CHECKPOINT_SIGNALING | Cell cycle | -0.81407 | 0.73812 | 0.86073 | 0.82741 | 0.72927 | 0.66520 | 0.75654 | 0.81446 | 0.86576 |
| GOBP_ALPHA_AMINO_ACID_CATABOLIC_PROCESS | Metabolism and energy | -1.33798 | 0.06349 | 0.31993 | 0.90582 | 0.66916 | 0.64015 | -0.86737 | 0.77592 | 0.85306 |
| GOBP_V_D_J_RECOMBINATION | Immunity | 0.66574 | 0.89168 | 0.88692 | 0.95859 | 0.54287 | 0.57579 | 0.71707 | 0.84652 | 0.87060 |
| GOBP_NEGATIVE_REGULATION_OF_UBIQUITIN_PROTEIN_TRANSFERASE_ACTIVITY | Immunity | 1.23008 | 0.22166 | 0.58181 | 1.27145 | 0.16735 | 0.27248 | -1.29670 | 0.15230 | 0.43805 |
| GOBP_GLUTAMINE_FAMILY_AMINO_ACID_BIOSYNTHETIC_PROCESS | Metabolism and energy | -1.09875 | 0.34895 | 0.68236 | 0.77743 | 0.77821 | 0.68621 | -0.94451 | 0.51944 | 0.74698 |
| GOBP_NEGATIVE_REGULATION_OF_INTRINSIC_APOPTOTIC_SIGNALING_PATHWAY_BY_P53_CLASS_MEDIATOR | Cell death | -1.13724 | 0.29940 | 0.64285 | 0.70882 | 0.85940 | 0.71329 | 0.53769 | 0.97606 | 0.87060 |
| GOBP_NUCLEAR_TRANSCRIBED_MRNA_CATABOLIC_PROCESS_DEADENYLATION_DEPENDENT_DECAY | Genetic and epigenetic information | -1.03656 | 0.40353 | 0.71899 | 0.95516 | 0.56934 | 0.59063 | -1.51503 | 0.01703 | 0.13782 |
| GOBP_POSITIVE_REGULATION_OF_CELL_CYCLE_G2_M_PHASE_TRANSITION | Cell cycle | -0.82920 | 0.72108 | 0.85522 | 1.55548 | 0.01411 | 0.04466 | -0.94381 | 0.53735 | 0.75720 |
| GOBP_NEGATIVE_REGULATION_OF_FATTY_ACID_METABOLIC_PROCESS | Metabolism and energy | -1.32403 | 0.11273 | 0.41999 | 0.80116 | 0.77125 | 0.68330 | 1.16117 | 0.26223 | 0.56339 |
| GOBP_AMP_METABOLIC_PROCESS | Genetic and epigenetic information | 1.19120 | 0.23635 | 0.59154 | -1.05770 | 0.36115 | 0.45379 | -1.23445 | 0.17608 | 0.46681 |
| GOBP_HOMOPHILIC_CELL_ADHESION_VIA_PLASMA_MEMBRANE_ADHESION_MOLECULES | ECM and metastasis | 1.85470 | 0.00002 | 0.00088 | 1.04027 | 0.40789 | 0.48992 | 1.67667 | 0.00001 | 0.00207 |
| GOBP_POSITIVE_REGULATION_OF_STEROID_METABOLIC_PROCESS | Metabolism and energy | -1.51138 | 0.03352 | 0.22784 | 0.73799 | 0.83861 | 0.70591 | -0.82281 | 0.74802 | 0.84245 |
| GOBP_ALPHA_AMINO_ACID_METABOLIC_PROCESS | Metabolism and energy | -1.01578 | 0.43181 | 0.73536 | 0.81054 | 0.88359 | 0.72016 | -0.81005 | 0.96294 | 0.87060 |
| GOBP_NEUTRAL_AMINO_ACID_TRANSPORT | Metabolism and energy | 1.18167 | 0.19563 | 0.55089 | 1.00853 | 0.47650 | 0.53437 | 1.01153 | 0.46613 | 0.72075 |
| GOBP_REGULATION_OF_TRIGLYCERIDE_METABOLIC_PROCESS | Metabolism and energy | -1.19315 | 0.21592 | 0.57507 | -1.19478 | 0.16839 | 0.27342 | -1.15018 | 0.22268 | 0.52153 |
| GOBP_NUCLEOSIDE_CATABOLIC_PROCESS | Genetic and epigenetic information | 1.03690 | 0.39591 | 0.71285 | -1.23345 | 0.15681 | 0.26039 | -1.30608 | 0.11696 | 0.38755 |
| GOBP_REGULATION_OF_HETEROCHROMATIN_FORMATION | Cell cycle | 0.69058 | 0.88718 | 0.88692 | 1.15901 | 0.27299 | 0.37866 | 0.50255 | 0.98829 | 0.87060 |
| GOBP_RIBONUCLEOSIDE_METABOLIC_PROCESS | Genetic and epigenetic information | 0.91403 | 0.58781 | 0.80397 | -1.38885 | 0.05590 | 0.12421 | -1.18830 | 0.18930 | 0.48492 |
| GOBP_REGULATION_OF_GENE_SILENCING_BY_RNA | Genetic and epigenetic information | -0.68548 | 0.92133 | 0.88692 | -1.31448 | 0.07403 | 0.15159 | -0.74586 | 0.89809 | 0.87060 |
| GOBP_RIBOSOMAL_SMALL_SUBUNIT_ASSEMBLY | Genetic and epigenetic information | NA | NA | NA | NA | NA | NA | NA | NA | NA |
| GOBP_PYRIMIDINE_CONTAINING_COMPOUND_CATABOLIC_PROCESS | Genetic and epigenetic information | 0.85339 | 0.70581 | 0.85127 | 1.00401 | 0.48230 | 0.53795 | -1.34320 | 0.08071 | 0.32289 |
| GOBP_NUCLEAR_TRANSCRIBED_MRNA_POLY_A_TAIL_SHORTENING | Genetic and epigenetic information | -1.22786 | 0.19441 | 0.55089 | 0.88481 | 0.65367 | 0.63337 | -1.46869 | 0.05204 | 0.25679 |
| GOBP_CELLULAR_AMINO_ACID_BIOSYNTHETIC_PROCESS | Metabolism and energy | -0.76223 | 0.87260 | 0.88692 | 0.81960 | 0.79167 | 0.68981 | -1.06632 | 0.31390 | 0.61099 |
| GOBP_RIBONUCLEOSIDE_CATABOLIC_PROCESS | Genetic and epigenetic information | 1.13388 | 0.29059 | 0.63799 | -1.39396 | 0.07876 | 0.15895 | -1.43952 | 0.07079 | 0.30304 |
| GOBP_REGULATION_OF_MONOCYTE_CHEMOTAXIS | Immunity | -0.93584 | 0.55980 | 0.79590 | -2.03915 | 0.00076 | 0.00471 | 1.05187 | 0.41043 | 0.68892 |
| GOBP_NEUTROPHIL_HOMEOSTASIS | Immunity | -0.57555 | 0.95730 | 0.88692 | -1.12729 | 0.27820 | 0.38394 | -0.97066 | 0.48110 | 0.72656 |
| GOBP_LEUKOCYTE_ADHESION_TO_VASCULAR_ENDOTHELIAL_CELL | Immunity | 0.84393 | 0.72936 | 0.85849 | -1.75177 | 0.00201 | 0.00990 | -1.46207 | 0.03706 | 0.21173 |
| GOBP_REGULATION_OF_COMPLEMENT_ACTIVATION | Immunity | 0.86630 | 0.63271 | 0.82583 | -2.52720 | 0.00001 | 0.00034 | 0.83311 | 0.70814 | 0.82853 |
| GOBP_POSITIVE_REGULATION_OF_MONOCYTE_CHEMOTAXIS | Immunity | -0.78963 | 0.75808 | 0.86598 | -1.68264 | 0.01663 | 0.05089 | 1.09637 | 0.35546 | 0.64257 |
| GOBP_LEUKOCYTE_HOMEOSTASIS | Immunity | -0.99554 | 0.47172 | 0.76062 | -1.38246 | 0.01369 | 0.04377 | -1.26328 | 0.07036 | 0.30262 |
| GOBP_LEUKOCYTE_TETHERING_OR_ROLLING | Immunity | 1.45403 | 0.05763 | 0.30366 | -1.41813 | 0.05028 | 0.11484 | -0.86434 | 0.68663 | 0.82339 |
| GOBP_NEGATIVE_REGULATION_OF_B_CELL_ACTIVATION | Immunity | -1.03344 | 0.41695 | 0.72712 | -1.86455 | 0.00164 | 0.00845 | -1.71883 | 0.01012 | 0.10119 |
| GOBP_MONOCYTE_CHEMOTAXIS | Immunity | -1.29313 | 0.10707 | 0.41235 | -2.13776 | 0.00002 | 0.00047 | -1.57649 | 0.01090 | 0.10650 |
| GOBP_RESPONSE_TO_CHEMOKINE | Immunity | -1.15656 | 0.21625 | 0.57572 | -2.09905 | 0.00003 | 0.00060 | -1.57124 | 0.00425 | 0.06080 |
| GOBP_POSITIVE_REGULATION_OF_VASCULAR_ENDOTHELIAL_GROWTH_FACTOR_PRODUCTION | ECM and metastasis | 1.37465 | 0.09887 | 0.40088 | -1.86718 | 0.00256 | 0.01203 | 0.89092 | 0.63793 | 0.80349 |
| GOBP_ANTIGEN_PROCESSING_AND_PRESENTATION_OF_PEPTIDE_OR_POLYSACCHARIDE_ANTIGEN_VIA_MHC_CLASS_II | Immunity | 1.25053 | 0.18837 | 0.54194 | -2.70565 | 0.00001 | 0.00035 | -1.95328 | 0.00398 | 0.05896 |
| GOBP_REGULATION_OF_CELL_MIGRATION_INVOLVED_IN_SPROUTING_ANGIOGENESIS | ECM and metastasis | 1.08281 | 0.32692 | 0.66505 | -1.62594 | 0.00949 | 0.03319 | 0.81705 | 0.75336 | 0.84411 |
| GOBP_NATURAL_KILLER_CELL_DIFFERENTIATION | Immunity | -1.16157 | 0.26674 | 0.61716 | 0.77457 | 0.79250 | 0.69003 | -1.16939 | 0.22351 | 0.52155 |
| GOBP_TOLL_LIKE_RECEPTOR_4_SIGNALING_PATHWAY | Immunity | -0.94972 | 0.54390 | 0.78945 | -2.69114 | 0.00001 | 0.00043 | -1.07663 | 0.31327 | 0.61034 |
| GOBP_POSITIVE_REGULATION_OF_LEUKOCYTE_CHEMOTAXIS | Immunity | -1.03293 | 0.40370 | 0.71899 | -1.99828 | 0.00003 | 0.00060 | 0.86692 | 0.73103 | 0.83705 |
| GOBP_REGULATION_OF_LEUKOCYTE_CHEMOTAXIS | Immunity | -1.04503 | 0.37829 | 0.70202 | -2.25120 | 0.00005 | 0.00071 | -1.27866 | 0.04709 | 0.24336 |
| GOBP_ENDOTHELIAL_CELL_DEVELOPMENT | ECM and metastasis | 0.98282 | 0.47893 | 0.76283 | -1.93061 | 0.00008 | 0.00097 | 1.18898 | 0.19956 | 0.49298 |
| GOBP_VASCULAR_ENDOTHELIAL_GROWTH_FACTOR_PRODUCTION | ECM and metastasis | -1.04249 | 0.40517 | 0.72021 | -1.99393 | 0.00053 | 0.00361 | 0.94886 | 0.55869 | 0.76784 |
| GOBP_IMMUNOGLOBULIN_PRODUCTION_INVOLVED_IN_IMMUNOGLOBULIN_MEDIATED_IMMUNE_RESPONSE | Immunity | -1.01849 | 0.43444 | 0.73677 | 0.59336 | 0.97612 | 0.72077 | -0.75672 | 0.90584 | 0.87060 |
| GOBP_NEGATIVE_REGULATION_OF_HUMORAL_IMMUNE_RESPONSE | Immunity | -1.27676 | 0.17004 | 0.51376 | -1.95106 | 0.00336 | 0.01483 | 0.76235 | 0.79154 | 0.85708 |
| GOBP_REGULATION_OF_VASCULOGENESIS | ECM and metastasis | -1.41517 | 0.07883 | 0.35651 | 0.60628 | 0.93648 | 0.72077 | 1.57305 | 0.01849 | 0.14261 |
| GOBP_STEROID_CATABOLIC_PROCESS | Metabolism and energy | -1.44821 | 0.05585 | 0.29876 | 0.97487 | 0.52219 | 0.56264 | 0.84056 | 0.70700 | 0.82851 |
| GOBP_POSITIVE_REGULATION_OF_LEUKOCYTE_MIGRATION | Immunity | 1.29053 | 0.04757 | 0.27498 | -2.44133 | 0.00007 | 0.00091 | 0.79995 | 0.86267 | 0.87060 |
| GOBP_ESTABLISHMENT_OF_ENDOTHELIAL_BARRIER | ECM and metastasis | -1.15221 | 0.24990 | 0.60309 | -1.91905 | 0.00022 | 0.00188 | 0.95605 | 0.55545 | 0.76637 |
| GOBP_REGULATION_OF_HOMOTYPIC_CELL_CELL_ADHESION | ECM and metastasis | -1.02461 | 0.42914 | 0.73326 | -1.48580 | 0.02856 | 0.07620 | -1.28364 | 0.11855 | 0.38990 |
| GOBP_T_CELL_DIFFERENTIATION_INVOLVED_IN_IMMUNE_RESPONSE | Immunity | -1.21114 | 0.16763 | 0.50902 | -1.93281 | 0.00012 | 0.00124 | 0.72659 | 0.90042 | 0.87060 |
| GOBP_ANTIGEN_PROCESSING_AND_PRESENTATION_OF_EXOGENOUS_PEPTIDE_ANTIGEN_VIA_MHC_CLASS_II | Immunity | 1.04481 | 0.39390 | 0.71215 | -2.43647 | 0.00002 | 0.00047 | -2.28618 | 0.00023 | 0.00916 |
| GOBP_NEGATIVE_REGULATION_OF_CELL_JUNCTION_ASSEMBLY | ECM and metastasis | 1.11961 | 0.29177 | 0.63883 | 0.94718 | 0.56464 | 0.58800 | 1.13072 | 0.30308 | 0.60113 |
| GOBP_MONONUCLEAR_CELL_MIGRATION | Immunity | -1.28857 | 0.05317 | 0.29119 | -2.41007 | 0.00015 | 0.00147 | -1.00159 | 0.45709 | 0.71693 |
| GOBP_REGULATION_OF_B_CELL_PROLIFERATION | Immunity | -1.39473 | 0.04862 | 0.27714 | -1.78425 | 0.00038 | 0.00278 | -1.72379 | 0.00253 | 0.04367 |
| GOBP_MYELOID_LEUKOCYTE_MIGRATION | Immunity | -1.20241 | 0.11033 | 0.41625 | -2.35140 | 0.00029 | 0.00230 | -1.69315 | 0.00009 | 0.00552 |
| GOBP_POSITIVE_REGULATION_OF_TOLL_LIKE_RECEPTOR_SIGNALING_PATHWAY | Immunity | 1.05507 | 0.37204 | 0.69806 | -2.63308 | 0.00001 | 0.00038 | -0.93005 | 0.55820 | 0.76776 |
| GOBP_REGULATION_OF_MONONUCLEAR_CELL_MIGRATION | Immunity | -1.11121 | 0.26820 | 0.61880 | -2.23610 | 0.00004 | 0.00069 | 0.73935 | 0.91551 | 0.87060 |
| GOBP_MYELOID_LEUKOCYTE_ACTIVATION | Immunity | -1.46714 | 0.00358 | 0.05326 | -2.29759 | 0.00029 | 0.00231 | -1.52060 | 0.00043 | 0.01396 |
| GOBP_RESPONSE_TO_MACROPHAGE_COLONY_STIMULATING_FACTOR | Immunity | 1.34673 | 0.13547 | 0.45889 | -1.95456 | 0.00303 | 0.01380 | 0.81126 | 0.73358 | 0.83705 |
| GOBP_POSITIVE_REGULATION_OF_MONONUCLEAR_CELL_MIGRATION | Immunity | -1.10550 | 0.29713 | 0.64150 | -1.99366 | 0.00005 | 0.00076 | -1.01851 | 0.40704 | 0.68622 |
| GOBP_LYMPHOCYTE_HOMEOSTASIS | Immunity | -1.09050 | 0.32068 | 0.66109 | -1.49871 | 0.00923 | 0.03256 | -1.31834 | 0.06494 | 0.28849 |
| GOBP_PURINERGIC_NUCLEOTIDE_RECEPTOR_SIGNALING_PATHWAY | Genetic and epigenetic information | -0.96265 | 0.52155 | 0.77916 | -1.01013 | 0.43323 | 0.50899 | 0.94576 | 0.56230 | 0.76847 |
| GOBP_REGULATION_OF_CELL_SUBSTRATE_JUNCTION_ORGANIZATION | ECM and metastasis | 1.32548 | 0.07446 | 0.34854 | -1.65725 | 0.00163 | 0.00845 | 0.87956 | 0.69036 | 0.82445 |
| GOBP_NEGATIVE_REGULATION_OF_BLOOD_VESSEL_ENDOTHELIAL_CELL_MIGRATION | ECM and metastasis | -1.20698 | 0.20915 | 0.56852 | -0.98666 | 0.47658 | 0.53437 | 0.71290 | 0.87198 | 0.87060 |
| GOBP_CELL_ADHESION_MEDIATED_BY_INTEGRIN | ECM and metastasis | 1.68758 | 0.00227 | 0.03977 | -2.79566 | 0.00002 | 0.00056 | -1.18836 | 0.13753 | 0.41853 |
| GOBP_CELL_CELL_ADHESION_MEDIATED_BY_INTEGRIN | ECM and metastasis | -0.85872 | 0.66322 | 0.83352 | -2.40574 | 0.00005 | 0.00072 | -1.40692 | 0.08943 | 0.33928 |
| GOBP_NEGATIVE_REGULATION_OF_B_CELL_PROLIFERATION | Immunity | -1.09706 | 0.34993 | 0.68303 | -1.89680 | 0.00412 | 0.01743 | -1.72134 | 0.01935 | 0.14571 |
| GOBP_ANTIGEN_PROCESSING_AND_PRESENTATION_OF_EXOGENOUS_PEPTIDE_ANTIGEN | Immunity | 0.95558 | 0.50843 | 0.77324 | -2.46701 | 0.00001 | 0.00045 | -2.12484 | 0.00085 | 0.02175 |
| GOBP_NEGATIVE_REGULATION_OF_ADAPTIVE_IMMUNE_RESPONSE | Immunity | -1.16907 | 0.22291 | 0.58181 | -1.65632 | 0.00257 | 0.01207 | 1.04745 | 0.40764 | 0.68666 |
| GOBP_POSITIVE_REGULATION_OF_CELL_SUBSTRATE_JUNCTION_ORGANIZATION | ECM and metastasis | 1.21819 | 0.19560 | 0.55089 | -1.52689 | 0.02683 | 0.07279 | 0.98131 | 0.50938 | 0.74167 |
| GOBP_REGULATION_OF_B_CELL_DIFFERENTIATION | Immunity | 0.81343 | 0.74886 | 0.86385 | -2.00462 | 0.00057 | 0.00381 | -1.26817 | 0.13332 | 0.41257 |
| GOBP_REGULATION_OF_COLLAGEN_METABOLIC_PROCESS | ECM and metastasis | 1.31770 | 0.11089 | 0.41658 | -2.09047 | 0.00008 | 0.00097 | 1.66941 | 0.00319 | 0.04999 |
| GOBP_POSITIVE_REGULATION_OF_PHOSPHOLIPASE_ACTIVITY | Metabolism and energy | -0.96528 | 0.52117 | 0.77916 | -1.67029 | 0.00308 | 0.01389 | 1.25733 | 0.13850 | 0.42024 |
| GOBP_NEGATIVE_REGULATION_OF_LYMPHOCYTE_ACTIVATION | Immunity | -1.28953 | 0.06368 | 0.32064 | -1.71796 | 0.00008 | 0.00097 | 1.00640 | 0.48289 | 0.72821 |
| GOBP_T_CELL_MEDIATED_IMMUNITY | Immunity | -1.35724 | 0.04858 | 0.27712 | -1.94416 | 0.00003 | 0.00061 | -0.93727 | 0.61856 | 0.79589 |
| GOBP_CYCLIC_NUCLEOTIDE_BIOSYNTHETIC_PROCESS | Genetic and epigenetic information | 0.70229 | 0.86134 | 0.88692 | 1.12565 | 0.31780 | 0.41852 | -1.09652 | 0.30944 | 0.60854 |
| GOBP_REGULATION_OF_B_CELL_ACTIVATION | Immunity | -1.17682 | 0.17562 | 0.52416 | -1.86767 | 0.00005 | 0.00072 | -1.79939 | 0.00010 | 0.00552 |
| GOBP_INTERLEUKIN_6_PRODUCTION | Immunity | -1.13415 | 0.22388 | 0.58301 | -2.19918 | 0.00007 | 0.00091 | 0.61934 | 0.99130 | 0.87060 |
| GOBP_POSITIVE_REGULATION_OF_CHEMOKINE_PRODUCTION | Immunity | -1.11945 | 0.27983 | 0.62660 | -2.02703 | 0.00005 | 0.00073 | -0.99397 | 0.45946 | 0.71866 |
| GOBP_REGULATION_OF_PHOSPHOLIPASE_ACTIVITY | Metabolism and energy | 0.94132 | 0.56076 | 0.79653 | -1.78980 | 0.00049 | 0.00340 | 1.09791 | 0.32648 | 0.61917 |
| GOBP_POSITIVE_REGULATION_OF_INTERLEUKIN_6_PRODUCTION | Immunity | -0.94206 | 0.57394 | 0.79849 | -2.17279 | 0.00003 | 0.00060 | 0.64247 | 0.97283 | 0.87060 |
| GOBP_POSITIVE_REGULATION_OF_T_CELL_CYTOKINE_PRODUCTION | Immunity | -1.37636 | 0.09058 | 0.38325 | -1.70147 | 0.01036 | 0.03545 | 0.61501 | 0.94252 | 0.87060 |
| GOBP_T_CELL_ACTIVATION_INVOLVED_IN_IMMUNE_RESPONSE | Immunity | -1.27699 | 0.09003 | 0.38249 | -1.80623 | 0.00004 | 0.00063 | 1.01601 | 0.46105 | 0.71866 |
| GOBP_LYMPHOCYTE_ACTIVATION_INVOLVED_IN_IMMUNE_RESPONSE | Immunity | -1.04462 | 0.37031 | 0.69724 | -1.63297 | 0.00015 | 0.00147 | 0.63522 | 0.99229 | 0.87060 |
| GOBP_NEGATIVE_REGULATION_OF_IMMUNE_EFFECTOR_PROCESS | Immunity | -1.16537 | 0.19455 | 0.55089 | -1.97581 | 0.00004 | 0.00067 | 0.98469 | 0.52386 | 0.75092 |
| GOBP_LEUKOCYTE_MIGRATION | Immunity | -1.12087 | 0.18876 | 0.54202 | -2.27834 | 0.00326 | 0.01453 | -1.38860 | 0.00047 | 0.01452 |
| GOBP_ADAPTIVE_IMMUNE_RESPONSE_BASED_ON_SOMATIC_RECOMBINATION_OF_IMMUNE_RECEPTORS_BUILT_FROM_IMMUNOGLOBULIN_SUPERFAMILY_DOMAINS | Immunity | -1.24896 | 0.06039 | 0.30972 | -2.42547 | 0.00058 | 0.00386 | -0.95287 | 0.66466 | 0.81342 |
| GOBP_INTERLEUKIN_10_PRODUCTION | Immunity | -1.09341 | 0.32056 | 0.66109 | -1.99888 | 0.00008 | 0.00098 | 0.73069 | 0.88195 | 0.87060 |
| GOBP_CELL_JUNCTION_DISASSEMBLY | ECM and metastasis | 1.68023 | 0.01810 | 0.15399 | -2.44491 | 0.00001 | 0.00036 | 0.77806 | 0.78284 | 0.85564 |
| GOBP_REGULATION_OF_HUMORAL_IMMUNE_RESPONSE | Immunity | -1.22497 | 0.18025 | 0.52973 | -1.69520 | 0.00423 | 0.01779 | 0.78777 | 0.79856 | 0.85933 |
| GOBP_PRODUCTION_OF_MOLECULAR_MEDIATOR_INVOLVED_IN_INFLAMMATORY_RESPONSE | Immunity | -0.68953 | 0.95259 | 0.88692 | -2.29569 | 0.00002 | 0.00055 | -1.72052 | 0.00151 | 0.03083 |
| GOBP_ANTIGEN_PROCESSING_AND_PRESENTATION_OF_EXOGENOUS_ANTIGEN | Immunity | 0.62740 | 0.95187 | 0.88692 | -2.25583 | 0.00005 | 0.00075 | -1.49969 | 0.04372 | 0.23268 |
| GOBP_REGULATION_OF_LYMPHOCYTE_MEDIATED_IMMUNITY | Immunity | -1.53790 | 0.00278 | 0.04587 | -1.55443 | 0.00037 | 0.00273 | 0.63499 | 0.98930 | 0.87060 |
| GOBP_REGULATION_OF_TOLL_LIKE_RECEPTOR_4_SIGNALING_PATHWAY | Immunity | 1.02534 | 0.41211 | 0.72348 | -2.35324 | 0.00001 | 0.00045 | -1.24471 | 0.16023 | 0.44882 |
| GOBP_POSITIVE_REGULATION_OF_INTERLEUKIN_2_PRODUCTION | Immunity | -0.94350 | 0.55077 | 0.79204 | -2.26293 | 0.00003 | 0.00061 | -1.46659 | 0.04898 | 0.24893 |
| GOBP_LEUKOCYTE_MEDIATED_IMMUNITY | Immunity | -1.51880 | 0.00026 | 0.00740 | -1.98896 | 0.00235 | 0.01125 | -1.13016 | 0.06846 | 0.29860 |
| GOBP_MACROPHAGE_ACTIVATION | Immunity | -1.33120 | 0.06016 | 0.30920 | -2.14342 | 0.00003 | 0.00061 | -1.26811 | 0.06318 | 0.28325 |
| GOBP_LEUKOCYTE_CHEMOTAXIS | Immunity | -1.35208 | 0.02080 | 0.16955 | -2.33069 | 0.00027 | 0.00218 | -1.59530 | 0.00016 | 0.00746 |
| GOBP_ACTIVATION_OF_PHOSPHOLIPASE_C_ACTIVITY | Metabolism and energy | 1.01448 | 0.42640 | 0.73267 | -1.85072 | 0.00262 | 0.01224 | 1.20770 | 0.21445 | 0.51362 |
| GOBP_SPROUTING_ANGIOGENESIS | ECM and metastasis | 1.30982 | 0.04780 | 0.27498 | -1.41416 | 0.00534 | 0.02127 | 1.34061 | 0.03525 | 0.20747 |
| GOBP_NEGATIVE_REGULATION_OF_CELL_SUBSTRATE_JUNCTION_ORGANIZATION | ECM and metastasis | 1.07515 | 0.35817 | 0.69082 | -1.39883 | 0.08611 | 0.17011 | 1.08229 | 0.37519 | 0.66199 |
| GOBP_REGULATION_OF_NEUTROPHIL_MIGRATION | Immunity | 1.73009 | 0.00513 | 0.06875 | -2.55533 | 0.00001 | 0.00043 | -1.36067 | 0.06713 | 0.29394 |
| GOBP_NEGATIVE_REGULATION_OF_B_CELL_MEDIATED_IMMUNITY | Immunity | -0.62114 | 0.92758 | 0.88692 | -1.55145 | 0.03998 | 0.09711 | 0.90598 | 0.60938 | 0.79175 |
| GOBP_NEGATIVE_REGULATION_OF_T_CELL_RECEPTOR_SIGNALING_PATHWAY | Immunity | 1.01783 | 0.42228 | 0.73039 | -1.96690 | 0.00146 | 0.00774 | 0.68338 | 0.88867 | 0.87060 |
| GOBP_DENDRITIC_CELL_MIGRATION | Immunity | -1.06223 | 0.37985 | 0.70230 | -2.02407 | 0.00052 | 0.00358 | 0.45822 | 0.99669 | 0.87060 |
| GOBP_NEGATIVE_REGULATION_OF_MONONUCLEAR_CELL_MIGRATION | Immunity | -1.09019 | 0.35442 | 0.68772 | -1.83587 | 0.00523 | 0.02091 | 0.96369 | 0.53234 | 0.75502 |
| GOBP_CELL_ACTIVATION_INVOLVED_IN_IMMUNE_RESPONSE | Immunity | -1.31111 | 0.02635 | 0.19712 | -1.84041 | 0.00072 | 0.00452 | -1.11054 | 0.12962 | 0.40617 |
| GOBP_HETEROTYPIC_CELL_CELL_ADHESION | ECM and metastasis | 1.72897 | 0.00308 | 0.04839 | -1.96351 | 0.00010 | 0.00114 | -1.26445 | 0.10017 | 0.35676 |
| GOBP_REGULATION_OF_IMMUNE_EFFECTOR_PROCESS | Immunity | -1.53120 | 0.00019 | 0.00581 | -2.04050 | 0.00212 | 0.01034 | 0.68844 | 0.99224 | 0.87060 |
| GOBP_CHEMOKINE_PRODUCTION | Immunity | -1.04706 | 0.38023 | 0.70242 | -2.08782 | 0.00003 | 0.00060 | -1.05097 | 0.33654 | 0.62778 |
| GOBP_ADAPTIVE_IMMUNE_RESPONSE | Immunity | -1.37341 | 0.00444 | 0.06167 | -2.29665 | 0.00513 | 0.02062 | -1.33265 | 0.00139 | 0.02991 |
| GOBP_LYMPHOCYTE_MEDIATED_IMMUNITY | Immunity | -1.38991 | 0.00953 | 0.10543 | -2.06034 | 0.00049 | 0.00340 | -0.90281 | 0.83564 | 0.87060 |
| GOBP_REGULATION_OF_T_CELL_MEDIATED_IMMUNITY | Immunity | -1.32858 | 0.07382 | 0.34837 | -1.59949 | 0.00208 | 0.01019 | 0.88485 | 0.68869 | 0.82372 |
| GOBP_POSITIVE_REGULATION_OF_IMMUNE_EFFECTOR_PROCESS | Immunity | -1.56734 | 0.00046 | 0.01163 | -1.90429 | 0.00036 | 0.00267 | -1.23799 | 0.02438 | 0.16878 |
| GOBP_IMMUNE_RESPONSE_REGULATING_CELL_SURFACE_RECEPTOR_SIGNALING_PATHWAY | Immunity | -1.41687 | 0.00701 | 0.08610 | -1.94030 | 0.00038 | 0.00280 | -1.33455 | 0.00584 | 0.07356 |
| GOBP_NEGATIVE_REGULATION_OF_ANTIGEN_RECEPTOR_MEDIATED_SIGNALING_PATHWAY | Immunity | 0.82934 | 0.72383 | 0.85522 | -1.65832 | 0.00991 | 0.03417 | 0.68380 | 0.89861 | 0.87060 |
| GOBP_REGULATION_OF_CD4_POSITIVE_ALPHA_BETA_T_CELL_ACTIVATION | Immunity | -1.18286 | 0.19873 | 0.55539 | -2.20066 | 0.00002 | 0.00051 | 0.64313 | 0.96319 | 0.87060 |
| GOBP_MYELOID_LEUKOCYTE_MEDIATED_IMMUNITY | Immunity | -1.61099 | 0.00232 | 0.04001 | -1.63719 | 0.00036 | 0.00269 | -1.35854 | 0.02538 | 0.17231 |
| GOBP_NEGATIVE_REGULATION_OF_INTERLEUKIN_12_PRODUCTION | Immunity | -0.62876 | 0.92398 | 0.88692 | -1.81786 | 0.00753 | 0.02785 | -0.47284 | 0.99669 | 0.87060 |
| GOBP_REGULATION_OF_MACROPHAGE_ACTIVATION | Immunity | -1.14902 | 0.24951 | 0.60260 | -1.88030 | 0.00027 | 0.00218 | -1.07558 | 0.30627 | 0.60517 |
| GOBP_T_HELPER_17_CELL_DIFFERENTIATION | Immunity | -1.03393 | 0.42019 | 0.72875 | -1.27405 | 0.12225 | 0.21839 | -0.84499 | 0.71386 | 0.83079 |
| GOBP_REGULATION_OF_LEUKOCYTE_MIGRATION | Immunity | 1.36721 | 0.01066 | 0.11142 | -2.51322 | 0.00026 | 0.00212 | 0.77369 | 0.92559 | 0.87060 |
| GOBP_ALPHA_BETA_T_CELL_PROLIFERATION | Immunity | -0.95276 | 0.53975 | 0.78821 | -1.84501 | 0.00098 | 0.00568 | -0.80298 | 0.81810 | 0.86666 |
| GOBP_LEUKOCYTE_CELL_CELL_ADHESION | Immunity | -1.32865 | 0.01276 | 0.12350 | -2.08785 | 0.00307 | 0.01386 | -1.40600 | 0.00044 | 0.01409 |
| GOBP_POSITIVE_REGULATION_OF_T_CELL_PROLIFERATION | Immunity | -1.19404 | 0.17220 | 0.51819 | -2.21219 | 0.00003 | 0.00060 | -1.39002 | 0.02391 | 0.16668 |
| GOBP_MYD88_DEPENDENT_TOLL_LIKE_RECEPTOR_SIGNALING_PATHWAY | Immunity | -0.63130 | 0.93310 | 0.88692 | -1.98550 | 0.00128 | 0.00700 | -1.00318 | 0.43434 | 0.70113 |
| GOBP_POSITIVE_REGULATION_OF_B_CELL_PROLIFERATION | Immunity | -1.32313 | 0.10088 | 0.40326 | -1.21600 | 0.13879 | 0.23864 | -1.19050 | 0.17720 | 0.46858 |
| GOBP_REGULATION_OF_HUMORAL_IMMUNE_RESPONSE_MEDIATED_BY_CIRCULATING_IMMUNOGLOBULIN | Immunity | -0.79862 | 0.74622 | 0.86258 | -1.53143 | 0.04055 | 0.09796 | 0.77949 | 0.77562 | 0.85306 |
| GOBP_REGULATION_OF_LEUKOCYTE_MEDIATED_IMMUNITY | Immunity | -1.59885 | 0.00030 | 0.00848 | -1.86714 | 0.00027 | 0.00218 | -0.96237 | 0.60541 | 0.78933 |
| GOBP_POSITIVE_REGULATION_OF_CYTOKINE_PRODUCTION | Immunity | -1.11733 | 0.18371 | 0.53413 | -1.95319 | 0.00962 | 0.03350 | 0.70245 | 0.99417 | 0.87060 |
| GOBP_CYTOKINE_PRODUCTION_INVOLVED_IN_IMMUNE_RESPONSE | Immunity | -1.01159 | 0.44184 | 0.74163 | -1.79696 | 0.00009 | 0.00099 | 0.86604 | 0.74654 | 0.84198 |
| GOBP_TRANSFORMING_GROWTH_FACTOR_BETA_PRODUCTION | ECM and metastasis | 1.76975 | 0.00445 | 0.06167 | -2.36866 | 0.00001 | 0.00043 | -1.40841 | 0.05752 | 0.27192 |
| GOBP_CELLULAR_RESPONSE_TO_LIPOPROTEIN_PARTICLE_STIMULUS | Metabolism and energy | -1.26158 | 0.16074 | 0.49833 | -1.93965 | 0.00097 | 0.00566 | -1.63969 | 0.01927 | 0.14571 |
| GOBP_NEGATIVE_REGULATION_OF_INTERLEUKIN_6_PRODUCTION | Immunity | -1.06470 | 0.36614 | 0.69647 | -1.40324 | 0.03428 | 0.08689 | 0.76703 | 0.83134 | 0.87012 |
| GOBP_NEUTROPHIL_MIGRATION | Immunity | -1.07445 | 0.32518 | 0.66423 | -2.57390 | 0.00005 | 0.00072 | -2.00647 | 0.00002 | 0.00289 |
| GOBP_REGULATION_OF_ALPHA_BETA_T_CELL_ACTIVATION | Immunity | -1.33145 | 0.05784 | 0.30429 | -2.01445 | 0.00003 | 0.00062 | -0.94810 | 0.59435 | 0.78511 |
| GOBP_REGULATION_OF_ANTIGEN_RECEPTOR_MEDIATED_SIGNALING_PATHWAY | Immunity | -0.84591 | 0.73202 | 0.85930 | -1.84032 | 0.00027 | 0.00220 | -0.94134 | 0.57354 | 0.77458 |
| GOBP_POSITIVE_REGULATION_OF_T_CELL_MEDIATED_IMMUNITY | Immunity | -1.28066 | 0.12666 | 0.44488 | -1.56526 | 0.00910 | 0.03223 | 0.76250 | 0.83680 | 0.87060 |
| GOBP_CELLULAR_RESPONSE_TO_LOW_DENSITY_LIPOPROTEIN_PARTICLE_STIMULUS | Metabolism and energy | -1.18866 | 0.24838 | 0.60103 | -1.77302 | 0.00990 | 0.03417 | -1.78893 | 0.01331 | 0.12051 |
| GOBP_MYELOID_LEUKOCYTE_CYTOKINE_PRODUCTION | Immunity | 0.88697 | 0.65668 | 0.83293 | -1.73498 | 0.00181 | 0.00916 | -1.01517 | 0.41344 | 0.69065 |
| GOBP_POSITIVE_REGULATION_OF_LIPASE_ACTIVITY | Metabolism and energy | -1.30667 | 0.09506 | 0.39193 | -1.48576 | 0.01112 | 0.03741 | 1.17683 | 0.21500 | 0.51420 |
| GOBP_CELL_MIGRATION_INVOLVED_IN_SPROUTING_ANGIOGENESIS | ECM and metastasis | -0.94957 | 0.54753 | 0.79084 | -1.76475 | 0.00111 | 0.00631 | -1.26192 | 0.11026 | 0.37464 |
| GOBP_L_GLUTAMATE_IMPORT_ACROSS_PLASMA_MEMBRANE | Metabolism and energy | 0.48085 | 0.98924 | 0.88692 | 0.71709 | 0.84444 | 0.70816 | 0.61510 | 0.93173 | 0.87060 |
| GOBP_POSITIVE_REGULATION_OF_COLLAGEN_METABOLIC_PROCESS | ECM and metastasis | 1.17075 | 0.24540 | 0.60068 | -1.93728 | 0.00163 | 0.00845 | 1.72277 | 0.00255 | 0.04367 |
| GOBP_COLLAGEN_METABOLIC_PROCESS | ECM and metastasis | 2.48925 | 0.00000 | 0.00041 | -2.74657 | 0.00003 | 0.00061 | 1.32767 | 0.05279 | 0.25918 |
| GOBP_REGULATION_OF_NEUTROPHIL_CHEMOTAXIS | Immunity | 1.62182 | 0.01962 | 0.16202 | -2.22187 | 0.00007 | 0.00086 | -1.44549 | 0.05453 | 0.26452 |
| GOBP_INTERLEUKIN_12_PRODUCTION | Immunity | -0.86644 | 0.69418 | 0.84619 | -1.91370 | 0.00014 | 0.00141 | -1.08646 | 0.28674 | 0.58711 |
| GOBP_REGULATION_OF_GRANULOCYTE_CHEMOTAXIS | Immunity | 1.46024 | 0.03874 | 0.24342 | -2.34447 | 0.00001 | 0.00045 | 0.89346 | 0.65526 | 0.81068 |
| GOBP_FATTY_ACID_TRANSMEMBRANE_TRANSPORT | Metabolism and energy | -0.88692 | 0.62536 | 0.82089 | -1.52850 | 0.04326 | 0.10325 | 1.07884 | 0.37821 | 0.66437 |
| GOBP_GRANULOCYTE_MIGRATION | Immunity | -1.05228 | 0.35979 | 0.69205 | -2.53207 | 0.00008 | 0.00096 | -1.87662 | 0.00007 | 0.00477 |
| GOBP_CD4_POSITIVE_ALPHA_BETA_T_CELL_PROLIFERATION | Immunity | -0.92652 | 0.57181 | 0.79815 | -1.75752 | 0.00902 | 0.03203 | 0.62711 | 0.92933 | 0.87060 |
| GOBP_FC_RECEPTOR_SIGNALING_PATHWAY | Immunity | -1.41009 | 0.04976 | 0.28086 | -1.66504 | 0.00302 | 0.01377 | -1.28315 | 0.09719 | 0.35126 |
| GOBP_NEGATIVE_REGULATION_OF_INTERFERON_GAMMA_PRODUCTION | Immunity | -1.28820 | 0.13217 | 0.45536 | -1.58979 | 0.01255 | 0.04099 | 0.74324 | 0.84567 | 0.87060 |
| GOBP_CYCLIC_NUCLEOTIDE_METABOLIC_PROCESS | Genetic and epigenetic information | -1.00365 | 0.45984 | 0.75303 | 1.20903 | 0.19931 | 0.30572 | 0.76026 | 0.82740 | 0.86963 |
| GOBP_IMMUNE_RESPONSE_REGULATING_SIGNALING_PATHWAY | Immunity | -1.25735 | 0.03488 | 0.23287 | -2.36161 | 0.00483 | 0.01963 | -1.19492 | 0.01559 | 0.13148 |
| GOBP_B_CELL_ACTIVATION | Immunity | -0.94413 | 0.61139 | 0.81528 | -1.52773 | 0.00054 | 0.00367 | -1.31839 | 0.00454 | 0.06280 |
| GOBP_POSITIVE_REGULATION_OF_B_CELL_ACTIVATION | Immunity | -1.12384 | 0.26672 | 0.61716 | -1.27551 | 0.05602 | 0.12438 | -1.28418 | 0.07112 | 0.30321 |
| GOBP_B_CELL_MEDIATED_IMMUNITY | Immunity | -0.94326 | 0.58020 | 0.80103 | -2.10697 | 0.00005 | 0.00071 | 0.43150 | 0.99999 | 0.87060 |
| GOBP_REGULATION_OF_PHOSPHOLIPASE_C_ACTIVITY | Metabolism and energy | 1.18942 | 0.20181 | 0.55999 | -1.84389 | 0.00118 | 0.00662 | 1.16678 | 0.24692 | 0.54407 |
| GOBP_COMPLEMENT_ACTIVATION | Immunity | -1.49079 | 0.02546 | 0.19267 | -2.42656 | 0.00001 | 0.00045 | 1.17063 | 0.23172 | 0.53116 |
| GOBP_NEGATIVE_REGULATION_OF_LEUKOCYTE_CELL_CELL_ADHESION | Immunity | -1.28403 | 0.07594 | 0.35169 | -1.60794 | 0.00038 | 0.00278 | 1.03160 | 0.42906 | 0.69921 |
| GOBP_REGULATION_OF_LEUKOCYTE_ADHESION_TO_VASCULAR_ENDOTHELIAL_CELL | Immunity | 0.85339 | 0.67667 | 0.83998 | -1.62393 | 0.01444 | 0.04553 | -1.09996 | 0.29242 | 0.59300 |
| GOBP_ACTIVATION_OF_IMMUNE_RESPONSE | Immunity | -1.38518 | 0.00794 | 0.09388 | -2.27576 | 0.00087 | 0.00519 | -1.12009 | 0.10764 | 0.36987 |
| GOBP_INTERLEUKIN_4_PRODUCTION | Immunity | -1.59336 | 0.01557 | 0.13982 | -1.96983 | 0.00087 | 0.00520 | -1.30600 | 0.11202 | 0.37785 |
| GOBP_NEGATIVE_REGULATION_OF_ENDOTHELIAL_CELL_PROLIFERATION | ECM and metastasis | -1.01551 | 0.44186 | 0.74163 | -1.22385 | 0.14035 | 0.24012 | 1.40272 | 0.05299 | 0.25918 |
| GOBP_NEGATIVE_REGULATION_OF_LYMPHOCYTE_MEDIATED_IMMUNITY | Immunity | -1.04205 | 0.39887 | 0.71513 | -1.65994 | 0.00348 | 0.01519 | 1.03796 | 0.42416 | 0.69646 |
| GOBP_REGULATION_OF_BLOOD_VESSEL_ENDOTHELIAL_CELL_MIGRATION | ECM and metastasis | -0.93510 | 0.58520 | 0.80271 | -1.64039 | 0.00078 | 0.00482 | 0.77858 | 0.85314 | 0.87060 |
| GOBP_ENDOTHELIAL_CELL_PROLIFERATION | ECM and metastasis | 1.31201 | 0.03691 | 0.23864 | -1.34533 | 0.00823 | 0.02977 | 1.23077 | 0.09655 | 0.35109 |
| GOBP_VASCULOGENESIS | ECM and metastasis | -1.07019 | 0.34639 | 0.68051 | -1.33219 | 0.03436 | 0.08696 | 1.77786 | 0.00008 | 0.00518 |
| GOBP_NEGATIVE_REGULATION_OF_CD4_POSITIVE_ALPHA_BETA_T_CELL_DIFFERENTIATION | Immunity | 1.05800 | 0.37409 | 0.69984 | -1.57982 | 0.02744 | 0.07386 | 0.87844 | 0.64975 | 0.80851 |
| GOBP_GAMMA_DELTA_T_CELL_ACTIVATION | Immunity | -1.24740 | 0.18546 | 0.53731 | -0.93716 | 0.55482 | 0.58222 | -1.14434 | 0.25265 | 0.55183 |
| GOBP_POSITIVE_REGULATION_OF_CELL_CELL_ADHESION | ECM and metastasis | -1.18054 | 0.11835 | 0.43015 | -2.20954 | 0.00087 | 0.00519 | -1.50237 | 0.00018 | 0.00805 |
| GOBP_T_CELL_MEDIATED_CYTOTOXICITY | Immunity | -1.64881 | 0.00716 | 0.08657 | -1.41869 | 0.03956 | 0.09624 | -0.98325 | 0.46917 | 0.72219 |
| GOBP_NEGATIVE_REGULATION_OF_IMMUNE_SYSTEM_PROCESS | Immunity | -1.14700 | 0.14054 | 0.46819 | -2.15859 | 0.00510 | 0.02055 | 0.91161 | 0.76606 | 0.84882 |
| GOBP_POSITIVE_REGULATION_OF_CYTOKINE_PRODUCTION_INVOLVED_IN_IMMUNE_RESPONSE | Immunity | -1.07591 | 0.33825 | 0.67263 | -1.66366 | 0.00104 | 0.00598 | 0.79851 | 0.81760 | 0.86666 |
| GOBP_NEGATIVE_REGULATION_OF_T_CELL_MEDIATED_IMMUNITY | Immunity | -0.99626 | 0.47397 | 0.76129 | -1.00808 | 0.43627 | 0.51082 | 0.90388 | 0.61818 | 0.79589 |
| GOBP_REGULATION_OF_ENDOTHELIAL_CELL_MIGRATION | ECM and metastasis | -1.15208 | 0.19256 | 0.54778 | -2.05422 | 0.00010 | 0.00106 | 0.92287 | 0.66516 | 0.81342 |
| GOBP_POSITIVE_REGULATION_OF_LYMPHOCYTE_MEDIATED_IMMUNITY | Immunity | -1.59851 | 0.00304 | 0.04819 | -1.28007 | 0.03762 | 0.09291 | -1.21919 | 0.09433 | 0.34708 |
| GOBP_POSITIVE_REGULATION_OF_LYMPHOCYTE_MIGRATION | Immunity | -0.95917 | 0.52884 | 0.78342 | -1.80317 | 0.00186 | 0.00939 | -1.58472 | 0.01969 | 0.14723 |
| GOBP_POSITIVE_REGULATION_OF_INTERFERON_GAMMA_PRODUCTION | Immunity | -0.98431 | 0.49075 | 0.76857 | -1.86541 | 0.00011 | 0.00118 | -1.04455 | 0.35505 | 0.64217 |
| GOBP_HUMORAL_IMMUNE_RESPONSE_MEDIATED_BY_CIRCULATING_IMMUNOGLOBULIN | Immunity | -0.93299 | 0.57281 | 0.79815 | -2.26461 | 0.00001 | 0.00043 | 0.79882 | 0.78760 | 0.85663 |
| GOBP_NEGATIVE_REGULATION_OF_CELL_CELL_ADHESION | ECM and metastasis | -1.17083 | 0.16081 | 0.49833 | -1.62106 | 0.00014 | 0.00137 | 0.99582 | 0.50850 | 0.74135 |
| GOBP_POSITIVE_REGULATION_OF_LEUKOCYTE_PROLIFERATION | Immunity | -1.17436 | 0.16694 | 0.50814 | -2.11181 | 0.00008 | 0.00097 | -1.13339 | 0.15115 | 0.43674 |
| GOBP_CD4_POSITIVE_ALPHA_BETA_T_CELL_ACTIVATION | Immunity | -1.34828 | 0.04957 | 0.28027 | -2.11608 | 0.00004 | 0.00062 | -0.98108 | 0.50538 | 0.74081 |
| GOBP_CD4_POSITIVE_ALPHA_BETA_T_CELL_DIFFERENTIATION | Immunity | -1.14965 | 0.23127 | 0.58981 | -2.13477 | 0.00002 | 0.00056 | -1.11392 | 0.22994 | 0.52942 |
| GOBP_T_CELL_PROLIFERATION | Immunity | -1.40897 | 0.01216 | 0.12043 | -1.83137 | 0.00017 | 0.00154 | -1.02272 | 0.38911 | 0.67147 |
| GOBP_ALPHA_BETA_T_CELL_ACTIVATION | Immunity | -1.53743 | 0.00283 | 0.04648 | -1.88718 | 0.00009 | 0.00103 | -1.15528 | 0.11874 | 0.39013 |
| GOBP_RESPONSE_TO_INTERFERON_ALPHA | Immunity | 1.18503 | 0.24672 | 0.60103 | -2.68897 | 0.00001 | 0.00034 | 0.78543 | 0.76835 | 0.85044 |
| GOBP_POSITIVE_REGULATION_OF_MYELOID_LEUKOCYTE_CYTOKINE_PRODUCTION_INVOLVED_IN_IMMUNE_RESPONSE | Immunity | -0.59226 | 0.96958 | 0.88692 | -1.40275 | 0.05259 | 0.11892 | 0.67386 | 0.91008 | 0.87060 |
| GOBP_POSITIVE_REGULATION_OF_HOMOTYPIC_CELL_CELL_ADHESION | ECM and metastasis | -1.35239 | 0.11564 | 0.42665 | -1.32003 | 0.12975 | 0.22773 | -1.22604 | 0.19702 | 0.49125 |
| GOBP_REGULATION_OF_LEUKOCYTE_PROLIFERATION | Immunity | -1.39982 | 0.00876 | 0.09918 | -2.05716 | 0.00042 | 0.00300 | -1.09537 | 0.17461 | 0.46526 |
| GOBP_POSITIVE_REGULATION_OF_ADAPTIVE_IMMUNE_RESPONSE | Immunity | -1.18249 | 0.17966 | 0.52935 | -1.73272 | 0.00007 | 0.00087 | -1.23085 | 0.08452 | 0.33049 |
| GOBP_NEUTROPHIL_CHEMOTAXIS | Immunity | -1.13337 | 0.24427 | 0.59886 | -2.58764 | 0.00003 | 0.00061 | -2.20557 | 0.00002 | 0.00267 |
| GOBP_REGULATION_OF_CELL_CELL_ADHESION | ECM and metastasis | -1.21680 | 0.05467 | 0.29436 | -1.80126 | 0.01111 | 0.03740 | -1.17953 | 0.02181 | 0.15746 |
| GOBP_MYELOID_CELL_ACTIVATION_INVOLVED_IN_IMMUNE_RESPONSE | Immunity | -1.55372 | 0.00639 | 0.08052 | -1.92714 | 0.00003 | 0.00061 | -1.38539 | 0.02396 | 0.16683 |
| GOBP_POSITIVE_REGULATION_OF_INTERLEUKIN_4_PRODUCTION | Immunity | -1.28325 | 0.15115 | 0.48229 | -2.16071 | 0.00022 | 0.00190 | -1.31108 | 0.11629 | 0.38614 |
| GOBP_REGULATION_OF_ADAPTIVE_IMMUNE_RESPONSE | Immunity | -1.23994 | 0.09012 | 0.38249 | -2.08927 | 0.00013 | 0.00132 | -1.02833 | 0.37191 | 0.65782 |
| GOBP_PRODUCTION_OF_MOLECULAR_MEDIATOR_OF_IMMUNE_RESPONSE | Immunity | -1.01705 | 0.42846 | 0.73270 | -1.57484 | 0.00023 | 0.00195 | -1.03701 | 0.33782 | 0.62870 |
| GOBP_LEUKOCYTE_DEGRANULATION | Immunity | -1.40969 | 0.03709 | 0.23868 | -1.68511 | 0.00081 | 0.00494 | -1.46385 | 0.01758 | 0.13901 |
| GOBP_POSITIVE_REGULATION_OF_LEUKOCYTE_APOPTOTIC_PROCESS | Immunity | -0.80252 | 0.75426 | 0.86446 | 0.88520 | 0.65096 | 0.63186 | 1.04066 | 0.42524 | 0.69710 |
| GOBP_POSITIVE_REGULATION_OF_TYPE_2_IMMUNE_RESPONSE | Immunity | -1.21010 | 0.22727 | 0.58683 | -1.84596 | 0.00622 | 0.02399 | 0.85647 | 0.67675 | 0.81982 |
| GOBP_REGULATION_OF_EPITHELIAL_TO_MESENCHYMAL_TRANSITION | ECM and metastasis | 1.40349 | 0.02965 | 0.21272 | -1.41468 | 0.01008 | 0.03466 | 1.20282 | 0.16216 | 0.45174 |
| GOBP_B_CELL_RECEPTOR_SIGNALING_PATHWAY | Immunity | -1.40480 | 0.05119 | 0.28509 | -1.75647 | 0.00113 | 0.00639 | -1.40288 | 0.04495 | 0.23713 |
| GOBP_POSITIVE_REGULATION_OF_LEUKOCYTE_CELL_CELL_ADHESION | Immunity | -1.24433 | 0.06830 | 0.33189 | -2.12953 | 0.00040 | 0.00290 | -1.57120 | 0.00011 | 0.00587 |
| GOBP_POSITIVE_REGULATION_OF_ENDOTHELIAL_CELL_PROLIFERATION | ECM and metastasis | 1.25813 | 0.09337 | 0.38747 | -1.24737 | 0.06201 | 0.13405 | 1.15562 | 0.22365 | 0.52165 |
| GOBP_NEGATIVE_REGULATION_OF_LEUKOCYTE_MEDIATED_IMMUNITY | Immunity | -1.16379 | 0.22724 | 0.58683 | -1.89680 | 0.00012 | 0.00130 | 0.79228 | 0.81362 | 0.86521 |
| GOBP_NEGATIVE_REGULATION_OF_CELL_ADHESION | ECM and metastasis | -0.97945 | 0.52224 | 0.77986 | -1.65501 | 0.00067 | 0.00432 | 1.07225 | 0.30977 | 0.60854 |
| GOBP_TOLL_LIKE_RECEPTOR_SIGNALING_PATHWAY | Immunity | -0.78178 | 0.88320 | 0.88692 | -2.74091 | 0.00004 | 0.00069 | -0.77733 | 0.95276 | 0.87060 |
| GOBP_REGULATION_OF_LEUKOCYTE_MEDIATED_CYTOTOXICITY | Immunity | -1.75628 | 0.00065 | 0.01538 | -1.35348 | 0.03025 | 0.07960 | 0.95852 | 0.56126 | 0.76847 |
| GOBP_LEUKOCYTE_PROLIFERATION | Immunity | -1.42611 | 0.00300 | 0.04815 | -1.87739 | 0.00135 | 0.00730 | 0.67110 | 0.99394 | 0.87060 |
| GOBP_INTERFERON_GAMMA_PRODUCTION | Immunity | -1.19754 | 0.16133 | 0.49873 | -1.95619 | 0.00004 | 0.00062 | -0.88544 | 0.76210 | 0.84683 |
| GOBP_REGULATION_OF_ALPHA_BETA_T_CELL_DIFFERENTIATION | Immunity | -1.17614 | 0.20840 | 0.56852 | -2.31468 | 0.00002 | 0.00050 | -1.10081 | 0.25992 | 0.56044 |
| GOBP_POSITIVE_REGULATION_OF_LEUKOCYTE_MEDIATED_IMMUNITY | Immunity | -1.53931 | 0.00468 | 0.06415 | -1.48485 | 0.00257 | 0.01207 | -1.11852 | 0.18957 | 0.48536 |
| GOBP_REGULATION_OF_PRODUCTION_OF_MOLECULAR_MEDIATOR_OF_IMMUNE_RESPONSE | Immunity | -1.23732 | 0.09264 | 0.38561 | -1.61394 | 0.00013 | 0.00130 | -1.08448 | 0.22531 | 0.52342 |
| GOBP_DENDRITIC_CELL_DIFFERENTIATION | Immunity | -0.97900 | 0.49824 | 0.77103 | -2.80182 | 0.00001 | 0.00043 | -1.22159 | 0.15063 | 0.43624 |
| GOBP_ANTIGEN_RECEPTOR_MEDIATED_SIGNALING_PATHWAY | Immunity | -1.30191 | 0.05363 | 0.29201 | -2.10270 | 0.00010 | 0.00106 | -1.48354 | 0.00217 | 0.03928 |
| GOBP_ALPHA_BETA_T_CELL_DIFFERENTIATION | Immunity | -1.35176 | 0.04655 | 0.27039 | -1.94458 | 0.00004 | 0.00065 | -1.20970 | 0.09481 | 0.34801 |
| GOBP_INFLAMMATORY_RESPONSE_TO_ANTIGENIC_STIMULUS | Immunity | -1.12874 | 0.26799 | 0.61854 | -0.97379 | 0.52328 | 0.56323 | 0.84527 | 0.74303 | 0.84002 |
| GOBP_NEGATIVE_REGULATION_OF_MYELOID_LEUKOCYTE_DIFFERENTIATION | Immunity | 1.17024 | 0.21359 | 0.57243 | -1.87392 | 0.00056 | 0.00378 | 1.22005 | 0.18030 | 0.47355 |
| GOBP_POSITIVE_REGULATION_OF_ALPHA_BETA_T_CELL_ACTIVATION | Immunity | -1.00080 | 0.46213 | 0.75453 | -2.25572 | 0.00002 | 0.00050 | -1.09182 | 0.27452 | 0.57338 |
| GOBP_POSITIVE_REGULATION_OF_INTERLEUKIN_1_PRODUCTION | Immunity | -1.47069 | 0.02501 | 0.18996 | -2.04786 | 0.00003 | 0.00061 | -1.42130 | 0.03202 | 0.19683 |
| GOBP_APOPTOTIC_CELL_CLEARANCE | Cell death | -0.99985 | 0.46500 | 0.75589 | -2.21566 | 0.00003 | 0.00061 | 0.74453 | 0.85610 | 0.87060 |
| GOBP_TYPE_2_IMMUNE_RESPONSE | Immunity | -1.17287 | 0.23880 | 0.59497 | -1.54018 | 0.01786 | 0.05370 | 1.03898 | 0.42440 | 0.69656 |
| GOBP_LYMPHOCYTE_MIGRATION | Immunity | -1.09969 | 0.28700 | 0.63427 | -2.39090 | 0.00004 | 0.00068 | -1.06567 | 0.29509 | 0.59482 |
| GOBP_POSITIVE_REGULATION_OF_T_CELL_MIGRATION | Immunity | 0.92342 | 0.57049 | 0.79815 | -1.89189 | 0.00136 | 0.00732 | -1.19386 | 0.18618 | 0.48089 |
| GOBP_T_CELL_TOLERANCE_INDUCTION | Immunity | 0.86238 | 0.62265 | 0.81990 | -1.35839 | 0.11564 | 0.21038 | -0.82956 | 0.68375 | 0.82159 |
| GOBP_REGULATION_OF_T_HELPER_CELL_DIFFERENTIATION | Immunity | -0.84885 | 0.70421 | 0.85060 | -2.02747 | 0.00013 | 0.00135 | 0.67674 | 0.91498 | 0.87060 |
| GOBP_POSITIVE_REGULATION_OF_PRODUCTION_OF_MOLECULAR_MEDIATOR_OF_IMMUNE_RESPONSE | Immunity | -1.25768 | 0.09586 | 0.39410 | -1.55870 | 0.00082 | 0.00497 | -1.22182 | 0.07527 | 0.31133 |
| GOBP_COLLAGEN_FIBRIL_ORGANIZATION | ECM and metastasis | 3.14216 | 0.00000 | 0.00041 | -3.07904 | 0.00001 | 0.00047 | 1.90256 | 0.00001 | 0.00219 |
| GOBP_TOLL_LIKE_RECEPTOR_2_SIGNALING_PATHWAY | Immunity | 0.69468 | 0.85845 | 0.88692 | -2.38881 | 0.00004 | 0.00065 | -1.15864 | 0.24773 | 0.54439 |
| GOBP_ANTIGEN_PROCESSING_AND_PRESENTATION | Immunity | 0.85478 | 0.76833 | 0.87004 | -3.01940 | 0.00002 | 0.00056 | -1.50294 | 0.01127 | 0.10871 |
| GOBP_ANTIGEN_PROCESSING_AND_PRESENTATION_OF_PEPTIDE_ANTIGEN | Immunity | -0.76249 | 0.83149 | 0.88692 | -3.01792 | 0.00001 | 0.00043 | -1.89281 | 0.00202 | 0.03767 |
| GOBP_T_CELL_MIGRATION | Immunity | 1.04425 | 0.36542 | 0.69625 | -2.42542 | 0.00002 | 0.00050 | -1.16605 | 0.17381 | 0.46456 |
| GOBP_REGULATION_OF_T_HELPER_1_TYPE_IMMUNE_RESPONSE | Immunity | -0.80737 | 0.75245 | 0.86412 | -2.16786 | 0.00011 | 0.00123 | 0.73279 | 0.84632 | 0.87060 |
| GOBP_CYCLIC_NUCLEOTIDE_MEDIATED_SIGNALING | Genetic and epigenetic information | -0.96033 | 0.53585 | 0.78709 | 1.21150 | 0.15684 | 0.26039 | 1.23924 | 0.13248 | 0.41119 |
| GOBP_MONONUCLEAR_CELL_DIFFERENTIATION | Immunity | -1.22906 | 0.04536 | 0.26668 | -1.59291 | 0.01149 | 0.03827 | -1.27876 | 0.00227 | 0.04070 |
| GOBP_POSITIVE_REGULATION_OF_INFLAMMATORY_RESPONSE | Immunity | -1.09444 | 0.28884 | 0.63630 | -2.55818 | 0.00006 | 0.00079 | -1.46381 | 0.00490 | 0.06560 |
| GOBP_REGULATION_OF_LYMPHOCYTE_ACTIVATION | Immunity | -1.30177 | 0.01346 | 0.12792 | -1.79144 | 0.01190 | 0.03943 | -1.28582 | 0.00115 | 0.02692 |
| GOBP_REGULATION_OF_T_CELL_ACTIVATION | Immunity | -1.38433 | 0.00571 | 0.07423 | -1.72609 | 0.00182 | 0.00921 | -1.22944 | 0.01082 | 0.10590 |
| GOBP_B_CELL_HOMEOSTASIS | Immunity | -0.70618 | 0.88264 | 0.88692 | -1.63944 | 0.01131 | 0.03782 | -1.63759 | 0.01954 | 0.14654 |
| GOBP_NEGATIVE_REGULATION_OF_LEUKOCYTE_PROLIFERATION | Immunity | -1.52507 | 0.01006 | 0.10876 | -1.76127 | 0.00013 | 0.00132 | -1.01709 | 0.40944 | 0.68819 |
| GOBP_POSITIVE_REGULATION_OF_MACROPHAGE_CHEMOTAXIS | Immunity | 0.89529 | 0.59046 | 0.80397 | -1.82310 | 0.00686 | 0.02590 | 1.28939 | 0.14984 | 0.43518 |
| GOBP_POSITIVE_REGULATION_OF_INTERLEUKIN_10_PRODUCTION | Immunity | -0.94249 | 0.55393 | 0.79395 | -1.82894 | 0.00170 | 0.00868 | 1.14764 | 0.27340 | 0.57240 |
| GOBP_REGULATION_OF_CD4_POSITIVE_ALPHA_BETA_T_CELL_DIFFERENTIATION | Immunity | -0.94904 | 0.54780 | 0.79088 | -2.26843 | 0.00001 | 0.00045 | -0.90732 | 0.63671 | 0.80282 |
| GOBP_EPITHELIAL_TO_MESENCHYMAL_TRANSITION | ECM and metastasis | 1.42048 | 0.01164 | 0.11882 | -1.39232 | 0.00448 | 0.01854 | 1.22712 | 0.10012 | 0.35676 |
| GOBP_POSITIVE_REGULATION_OF_MACROPHAGE_MIGRATION | Immunity | -0.91541 | 0.58979 | 0.80397 | -2.06846 | 0.00052 | 0.00356 | 1.08672 | 0.36295 | 0.64997 |
| GOBP_GRANULOCYTE_CHEMOTAXIS | Immunity | -1.09053 | 0.29913 | 0.64254 | -2.60040 | 0.00005 | 0.00071 | -1.92099 | 0.00005 | 0.00403 |
| GOBP_REGULATION_OF_EXTRINSIC_APOPTOTIC_SIGNALING_PATHWAY_VIA_DEATH_DOMAIN_RECEPTORS | Cell death | 1.56591 | 0.02011 | 0.16512 | -1.88262 | 0.00059 | 0.00393 | 0.79601 | 0.79128 | 0.85708 |
| GOBP_NEGATIVE_REGULATION_OF_CD4_POSITIVE_ALPHA_BETA_T_CELL_ACTIVATION | Immunity | 1.00300 | 0.44200 | 0.74167 | -1.29552 | 0.10468 | 0.19598 | 1.14987 | 0.27756 | 0.57742 |
| GOBP_REGULATION_OF_LYMPHOCYTE_MIGRATION | Immunity | -0.93478 | 0.57771 | 0.79962 | -2.03828 | 0.00003 | 0.00061 | -1.24138 | 0.11027 | 0.37464 |
| GOBP_REGULATION_OF_T_CELL_MIGRATION | Immunity | 0.94972 | 0.53558 | 0.78709 | -1.96953 | 0.00020 | 0.00174 | -0.95535 | 0.52953 | 0.75333 |
| GOBP_POSITIVE_REGULATION_OF_CELL_ADHESION | ECM and metastasis | -1.05318 | 0.32784 | 0.66505 | -2.37804 | 0.00926 | 0.03261 | -1.00590 | 0.44050 | 0.70560 |
| GOBP_B_CELL_ACTIVATION_INVOLVED_IN_IMMUNE_RESPONSE | Immunity | -0.82235 | 0.79117 | 0.87715 | -1.14075 | 0.17463 | 0.27984 | -0.92534 | 0.63441 | 0.80220 |
| GOBP_POSITIVE_REGULATION_OF_LYMPHOCYTE_DIFFERENTIATION | Immunity | -1.07302 | 0.32714 | 0.66505 | -1.75244 | 0.00005 | 0.00072 | -1.33759 | 0.02449 | 0.16921 |
| GOBP_TOLL_LIKE_RECEPTOR_3_SIGNALING_PATHWAY | Immunity | -0.84272 | 0.68851 | 0.84366 | -2.55589 | 0.00001 | 0.00035 | 0.85857 | 0.67581 | 0.81918 |
| GOBP_T_CELL_HOMEOSTASIS | Immunity | -1.22707 | 0.18069 | 0.53014 | -1.17186 | 0.18939 | 0.29524 | 0.78091 | 0.80508 | 0.86206 |
| GOBP_CD8_POSITIVE_ALPHA_BETA_T_CELL_ACTIVATION | Immunity | -1.33587 | 0.11474 | 0.42479 | 0.85909 | 0.68497 | 0.64579 | -0.89169 | 0.62216 | 0.79729 |
| GOBP_REGULATION_OF_LEUKOCYTE_DIFFERENTIATION | Immunity | -1.09341 | 0.25399 | 0.60696 | -1.84436 | 0.00098 | 0.00569 | -1.21237 | 0.02208 | 0.15840 |
| GOBP_T_CELL_DIFFERENTIATION | Immunity | -1.37497 | 0.01008 | 0.10886 | -1.51812 | 0.00075 | 0.00470 | -1.35501 | 0.00165 | 0.03233 |
| GOBP_POSITIVE_REGULATION_OF_EPITHELIAL_TO_MESENCHYMAL_TRANSITION | ECM and metastasis | 1.45753 | 0.03941 | 0.24531 | -1.70155 | 0.00240 | 0.01144 | 1.31536 | 0.09350 | 0.34606 |
| GOBP_B_CELL_PROLIFERATION | Immunity | -1.35800 | 0.04780 | 0.27498 | -1.35576 | 0.01732 | 0.05249 | -1.24956 | 0.07528 | 0.31133 |
| GOBP_CD4_POSITIVE_ALPHA_BETA_T_CELL_CYTOKINE_PRODUCTION | Immunity | -1.79370 | 0.00248 | 0.04194 | 0.73862 | 0.82518 | 0.70167 | 0.97165 | 0.52107 | 0.74848 |
| GOBP_MACROPHAGE_CYTOKINE_PRODUCTION | Immunity | 0.94433 | 0.53759 | 0.78709 | -2.09266 | 0.00013 | 0.00134 | 0.92323 | 0.59825 | 0.78561 |
| GOBP_POSITIVE_REGULATION_OF_CYTOKINE_PRODUCTION_INVOLVED_IN_INFLAMMATORY_RESPONSE | Immunity | -0.98293 | 0.49287 | 0.76857 | -1.34285 | 0.09605 | 0.18456 | -1.56852 | 0.03602 | 0.20979 |
| GOBP_COLLAGEN_BIOSYNTHETIC_PROCESS | ECM and metastasis | 1.92125 | 0.00083 | 0.01893 | -2.20552 | 0.00001 | 0.00043 | 1.59022 | 0.00741 | 0.08573 |
| GOBP_REGULATION_OF_T_CELL_MEDIATED_CYTOTOXICITY | Immunity | -1.35001 | 0.09879 | 0.40088 | -1.20527 | 0.17296 | 0.27838 | 0.99535 | 0.48895 | 0.73125 |
| GOBP_REGULATION_OF_MYELOID_LEUKOCYTE_DIFFERENTIATION | Immunity | 0.96901 | 0.52254 | 0.78003 | -1.78755 | 0.00004 | 0.00067 | 0.87154 | 0.73566 | 0.83705 |
| GOBP_RESPONSE_TO_TRANSFORMING_GROWTH_FACTOR_BETA | ECM and metastasis | 1.46195 | 0.00185 | 0.03407 | -1.63110 | 0.00050 | 0.00348 | 1.31617 | 0.01380 | 0.12291 |
| GOBP_NEGATIVE_REGULATION_OF_ALPHA_BETA_T_CELL_ACTIVATION | Immunity | 0.92697 | 0.57225 | 0.79815 | -1.31531 | 0.07742 | 0.15679 | 1.11213 | 0.31780 | 0.61238 |
| GOBP_POSITIVE_REGULATION_OF_ENDOTHELIAL_CELL_MIGRATION | ECM and metastasis | 1.06867 | 0.30800 | 0.65277 | -1.97278 | 0.00003 | 0.00061 | 0.98076 | 0.52849 | 0.75322 |
| GOBP_REGULATION_OF_LYMPHOCYTE_DIFFERENTIATION | Immunity | -1.09818 | 0.26565 | 0.61711 | -1.55438 | 0.00018 | 0.00165 | -1.08700 | 0.20853 | 0.50616 |
| GOBP_CELL_MATRIX_ADHESION | ECM and metastasis | 1.91555 | 0.00001 | 0.00041 | -2.60318 | 0.00028 | 0.00223 | 1.03848 | 0.40413 | 0.68501 |
| GOBP_FC_GAMMA_RECEPTOR_SIGNALING_PATHWAY | Immunity | -1.02187 | 0.43463 | 0.73689 | -1.67369 | 0.00847 | 0.03047 | -1.46068 | 0.05050 | 0.25289 |
| GOBP_IMMUNOGLOBULIN_PRODUCTION | Immunity | -0.94573 | 0.56961 | 0.79804 | -1.10474 | 0.21417 | 0.32176 | -0.90978 | 0.69664 | 0.82593 |
| GOBP_POSITIVE_REGULATION_OF_ALPHA_BETA_T_CELL_PROLIFERATION | Immunity | -0.74425 | 0.82163 | 0.88663 | -1.93988 | 0.00189 | 0.00947 | -0.93539 | 0.54361 | 0.76098 |
| GOBP_MHC_CLASS_II_BIOSYNTHETIC_PROCESS | Immunity | -1.28591 | 0.15734 | 0.49183 | -1.12403 | 0.27880 | 0.38411 | -0.82841 | 0.71251 | 0.83027 |
| GOBP_POSITIVE_REGULATION_OF_INTERLEUKIN_12_PRODUCTION | Immunity | -1.05897 | 0.37917 | 0.70211 | -1.79637 | 0.00211 | 0.01033 | -1.24944 | 0.13515 | 0.41475 |
| GOBP_REGULATION_OF_B_CELL_MEDIATED_IMMUNITY | Immunity | -0.94627 | 0.55525 | 0.79434 | -1.31616 | 0.05545 | 0.12375 | 0.55563 | 0.99003 | 0.87060 |
| GOBP_POSITIVE_REGULATION_OF_NEUTROPHIL_MIGRATION | Immunity | 1.67481 | 0.01256 | 0.12255 | -2.40013 | 0.00001 | 0.00041 | -1.33913 | 0.09228 | 0.34480 |
| GOBP_T_CELL_LINEAGE_COMMITMENT | Immunity | -1.01590 | 0.44510 | 0.74405 | 0.73153 | 0.84691 | 0.70908 | 0.73878 | 0.83788 | 0.87060 |
| GOBP_LEUKOCYTE_MEDIATED_CYTOTOXICITY | Immunity | -1.76462 | 0.00015 | 0.00493 | -1.48103 | 0.00266 | 0.01239 | 0.68052 | 0.96144 | 0.87060 |
| GOBP_MYELOID_LEUKOCYTE_DIFFERENTIATION | Immunity | 1.03721 | 0.34294 | 0.67707 | -2.15199 | 0.00021 | 0.00181 | -1.04036 | 0.32892 | 0.62103 |
| GOBP_INTERLEUKIN_2_PRODUCTION | Immunity | -1.65188 | 0.00396 | 0.05698 | -1.89582 | 0.00015 | 0.00145 | -1.08056 | 0.29572 | 0.59534 |
| GOBP_ENDOTHELIAL_CELL_MIGRATION | ECM and metastasis | 1.27417 | 0.03506 | 0.23381 | -2.41814 | 0.00022 | 0.00188 | 0.81771 | 0.87148 | 0.87060 |
| GOBP_PEPTIDE_ANTIGEN_ASSEMBLY_WITH_MHC_PROTEIN_COMPLEX | Immunity | NA | NA | NA | NA | NA | NA | NA | NA | NA |
| GOBP_NEGATIVE_REGULATION_OF_ALPHA_BETA_T_CELL_DIFFERENTIATION | Immunity | 0.99888 | 0.44831 | 0.74506 | -1.59588 | 0.02100 | 0.06065 | 1.12470 | 0.31496 | 0.61180 |
| GOBP_POSITIVE_REGULATION_OF_FOCAL_ADHESION_ASSEMBLY | ECM and metastasis | 1.31509 | 0.13559 | 0.45889 | -1.38158 | 0.07486 | 0.15304 | 1.19977 | 0.22735 | 0.52613 |
| GOBP_REGULATION_OF_LIPASE_ACTIVITY | Metabolism and energy | -1.20571 | 0.16503 | 0.50571 | -1.72110 | 0.00024 | 0.00203 | 0.98014 | 0.52573 | 0.75201 |
| GOBP_REGULATION_OF_ENDOTHELIAL_CELL_DIFFERENTIATION | ECM and metastasis | -1.28809 | 0.13487 | 0.45867 | 0.79649 | 0.78144 | 0.68692 | 1.25514 | 0.15855 | 0.44613 |
| GOBP_T_HELPER_17_TYPE_IMMUNE_RESPONSE | Immunity | -1.08118 | 0.34694 | 0.68111 | -1.41176 | 0.04020 | 0.09738 | 0.67149 | 0.92139 | 0.87060 |
| GOBP_POSITIVE_REGULATION_OF_NATURAL_KILLER_CELL_MEDIATED_IMMUNITY | Immunity | -1.63379 | 0.01188 | 0.11968 | 0.95897 | 0.54558 | 0.57724 | -0.97759 | 0.47636 | 0.72645 |
| GOBP_POSITIVE_REGULATION_OF_CD4_POSITIVE_ALPHA_BETA_T_CELL_ACTIVATION | Immunity | -0.86312 | 0.68298 | 0.84172 | -2.26626 | 0.00001 | 0.00043 | -0.87519 | 0.68285 | 0.82131 |
| GOBP_PHOSPHATIDYLINOSITOL_PHOSPHATE_BIOSYNTHETIC_PROCESS | Metabolism and energy | -1.02147 | 0.42871 | 0.73292 | 0.52646 | 0.99440 | 0.72077 | -0.95537 | 0.54264 | 0.76096 |
| GOBP_POSITIVE_REGULATION_OF_ALPHA_BETA_T_CELL_DIFFERENTIATION | Immunity | -0.96780 | 0.51649 | 0.77653 | -2.24182 | 0.00001 | 0.00045 | -1.19886 | 0.16236 | 0.45174 |
| GOBP_REGULATION_OF_OXIDATIVE_PHOSPHORYLATION | Metabolism and energy | 1.70431 | 0.01673 | 0.14716 | -1.59071 | 0.02547 | 0.06996 | -1.29597 | 0.13548 | 0.41539 |
| GOBP_MYELOID_DENDRITIC_CELL_ACTIVATION | Immunity | -0.72601 | 0.85330 | 0.88692 | -2.44028 | 0.00001 | 0.00038 | -0.94200 | 0.53784 | 0.75755 |
| GOBP_POSITIVE_REGULATION_OF_TRANSFORMING_GROWTH_FACTOR_BETA_PRODUCTION | ECM and metastasis | 1.10059 | 0.32699 | 0.66505 | -1.70517 | 0.01364 | 0.04369 | -1.25371 | 0.16440 | 0.45338 |
| GOBP_REGULATION_OF_NATURAL_KILLER_CELL_MEDIATED_IMMUNITY | Immunity | -1.65878 | 0.00575 | 0.07465 | 0.57057 | 0.97855 | 0.72077 | 0.85781 | 0.70300 | 0.82749 |
| GOBP_POSITIVE_REGULATION_OF_MACROPHAGE_ACTIVATION | Immunity | -1.32409 | 0.12268 | 0.43786 | -1.88570 | 0.00284 | 0.01304 | 0.81285 | 0.74214 | 0.83983 |
| GOBP_NEGATIVE_REGULATION_OF_T_HELPER_CELL_DIFFERENTIATION | Immunity | 1.05053 | 0.38508 | 0.70674 | -1.48633 | 0.05402 | 0.12135 | 0.90582 | 0.61056 | 0.79247 |
| GOBP_CHOLESTEROL_STORAGE | Metabolism and energy | -1.39391 | 0.08714 | 0.37614 | -1.97055 | 0.00215 | 0.01047 | -0.87496 | 0.63576 | 0.80282 |
| GOBP_REGULATION_OF_GLUCOSE_TRANSMEMBRANE_TRANSPORT | Metabolism and energy | -0.83216 | 0.76293 | 0.86736 | 1.01088 | 0.47400 | 0.53240 | 0.98977 | 0.50530 | 0.74081 |
| GOBP_REGULATION_OF_EXTRACELLULAR_MATRIX_ORGANIZATION | ECM and metastasis | 1.49369 | 0.02797 | 0.20436 | -2.01020 | 0.00008 | 0.00096 | 1.83338 | 0.00010 | 0.00558 |
| GOBP_POSITIVE_REGULATION_OF_MACROPHAGE_CYTOKINE_PRODUCTION | Immunity | 0.52520 | 0.98393 | 0.88692 | -1.83029 | 0.00446 | 0.01849 | 1.03566 | 0.43335 | 0.70113 |
| GOBP_HETEROPHILIC_CELL_CELL_ADHESION_VIA_PLASMA_MEMBRANE_CELL_ADHESION_MOLECULES | ECM and metastasis | 1.48047 | 0.03480 | 0.23262 | 0.94638 | 0.57681 | 0.59432 | 0.96022 | 0.54806 | 0.76418 |
| GOBP_REGULATION_OF_LEUKOCYTE_APOPTOTIC_PROCESS | Immunity | -1.21290 | 0.15580 | 0.48876 | -1.76995 | 0.00013 | 0.00132 | -0.86020 | 0.79406 | 0.85722 |
| GOBP_REGULATION_OF_T_CELL_RECEPTOR_SIGNALING_PATHWAY | Immunity | 1.01913 | 0.41534 | 0.72570 | -2.28519 | 0.00001 | 0.00043 | -0.99302 | 0.45242 | 0.71278 |
| GOBP_T_CELL_CYTOKINE_PRODUCTION | Immunity | -1.26324 | 0.15461 | 0.48742 | -1.38469 | 0.05619 | 0.12466 | 1.04108 | 0.42186 | 0.69509 |
| GOBP_REGULATION_OF_LIPID_STORAGE | Metabolism and energy | -0.97324 | 0.50754 | 0.77250 | -1.96762 | 0.00016 | 0.00148 | -0.71233 | 0.94202 | 0.87060 |
| GOBP_NEGATIVE_REGULATION_OF_EXTRINSIC_APOPTOTIC_SIGNALING_PATHWAY_VIA_DEATH_DOMAIN_RECEPTORS | Cell death | 1.62595 | 0.02277 | 0.17984 | -2.01783 | 0.00076 | 0.00472 | 0.76589 | 0.80324 | 0.86123 |
| GOBP_MACROPHAGE_ACTIVATION_INVOLVED_IN_IMMUNE_RESPONSE | Immunity | -0.84179 | 0.68723 | 0.84324 | -1.96342 | 0.00243 | 0.01153 | -0.78628 | 0.76945 | 0.85090 |
| GOBP_REGULATION_OF_TOLL_LIKE_RECEPTOR_SIGNALING_PATHWAY | Immunity | -0.85701 | 0.72080 | 0.85522 | -2.33422 | 0.00002 | 0.00050 | -1.01635 | 0.41185 | 0.68967 |
| GOBP_REGULATION_OF_AMINO_ACID_TRANSMEMBRANE_TRANSPORT | Metabolism and energy | 0.76888 | 0.76771 | 0.86968 | 0.93750 | 0.57200 | 0.59128 | -1.33409 | 0.12012 | 0.39204 |
| GOBP_CYTOKINE_PRODUCTION_INVOLVED_IN_INFLAMMATORY_RESPONSE | Immunity | -0.72176 | 0.90040 | 0.88692 | -1.87427 | 0.00020 | 0.00176 | -1.39107 | 0.04865 | 0.24836 |
| GOBP_POSITIVE_REGULATION_OF_RECEPTOR_SIGNALING_PATHWAY_VIA_STAT | Immunity | -0.97754 | 0.50067 | 0.77188 | 1.21403 | 0.18507 | 0.29012 | 1.03789 | 0.42447 | 0.69656 |
| GOBP_POSITIVE_REGULATION_OF_TUMOR_NECROSIS_FACTOR_SUPERFAMILY_CYTOKINE_PRODUCTION | Immunity | -1.04713 | 0.37785 | 0.70150 | -2.50978 | 0.00003 | 0.00061 | -0.91142 | 0.69200 | 0.82498 |
| GOBP_ACTIVATED_T_CELL_PROLIFERATION | Immunity | -1.08383 | 0.34228 | 0.67661 | -1.25157 | 0.11265 | 0.20648 | -0.92134 | 0.59717 | 0.78557 |
| GOBP_T_HELPER_1_CELL_DIFFERENTIATION | Immunity | 1.29583 | 0.15933 | 0.49495 | -2.20505 | 0.00028 | 0.00222 | 1.13126 | 0.31121 | 0.60881 |
| GOBP_POSITIVE_REGULATION_OF_ENDOTHELIAL_CELL_APOPTOTIC_PROCESS | ECM and metastasis | 1.00508 | 0.44026 | 0.74070 | -1.58988 | 0.03240 | 0.08351 | 1.37912 | 0.08899 | 0.33899 |
| GOBP_EXTRACELLULAR_MATRIX_ASSEMBLY | ECM and metastasis | 1.58754 | 0.01762 | 0.15177 | -2.42492 | 0.00001 | 0.00043 | 1.73172 | 0.00091 | 0.02251 |
| GOBP_LEUKOCYTE_ACTIVATION_INVOLVED_IN_INFLAMMATORY_RESPONSE | Immunity | -1.75571 | 0.00166 | 0.03143 | -2.03731 | 0.00009 | 0.00100 | -1.76021 | 0.00450 | 0.06276 |
| GOBP_POSITIVE_REGULATION_OF_BLOOD_VESSEL_ENDOTHELIAL_CELL_MIGRATION | ECM and metastasis | 0.98721 | 0.46817 | 0.75874 | -1.58923 | 0.00667 | 0.02533 | 0.82687 | 0.75523 | 0.84452 |
| GOBP_COLLAGEN_CATABOLIC_PROCESS | ECM and metastasis | 2.40123 | 0.00000 | 0.00041 | -2.48408 | 0.00001 | 0.00043 | 0.79615 | 0.78897 | 0.85705 |
| GOBP_CELL_SUBSTRATE_JUNCTION_ORGANIZATION | ECM and metastasis | 1.77892 | 0.00046 | 0.01163 | -2.17135 | 0.00003 | 0.00061 | 1.08616 | 0.33140 | 0.62346 |
| GOBP_COMPLEMENT_ACTIVATION_ALTERNATIVE_PATHWAY | Immunity | -0.91463 | 0.58717 | 0.80397 | -2.21949 | 0.00037 | 0.00272 | 0.67047 | 0.88699 | 0.87060 |
| GOBP_MEMBRANE_LIPID_CATABOLIC_PROCESS | Metabolism and energy | -1.15665 | 0.26102 | 0.61293 | -1.24023 | 0.13922 | 0.23931 | -0.62534 | 0.97194 | 0.87060 |
| GOBP_REGULATION_OF_INTEGRIN_MEDIATED_SIGNALING_PATHWAY | ECM and metastasis | 1.52505 | 0.05347 | 0.29193 | -1.67601 | 0.01618 | 0.04979 | 1.14416 | 0.29595 | 0.59541 |
| GOBP_FOCAL_ADHESION_ASSEMBLY | ECM and metastasis | 1.54907 | 0.00957 | 0.10544 | -1.84189 | 0.00007 | 0.00090 | 1.11411 | 0.29203 | 0.59267 |
| GOBP_NEGATIVE_REGULATION_OF_LEUKOCYTE_CHEMOTAXIS | Immunity | 1.07145 | 0.36004 | 0.69224 | -2.23675 | 0.00018 | 0.00162 | 1.03680 | 0.43260 | 0.70113 |
| GOBP_TUMOR_NECROSIS_FACTOR_SUPERFAMILY_CYTOKINE_PRODUCTION | Immunity | -1.03479 | 0.39276 | 0.71150 | -2.52367 | 0.00010 | 0.00107 | -0.94837 | 0.62943 | 0.80001 |
| GOBP_T_HELPER_1_TYPE_IMMUNE_RESPONSE | Immunity | 0.92732 | 0.57329 | 0.79827 | -2.23845 | 0.00001 | 0.00043 | 0.84276 | 0.72499 | 0.83517 |
| GOBP_BLOOD_VESSEL_ENDOTHELIAL_CELL_MIGRATION | ECM and metastasis | -1.15192 | 0.21281 | 0.57207 | -1.77933 | 0.00004 | 0.00066 | 0.84680 | 0.77519 | 0.85306 |
| GOBP_POSITIVE_REGULATION_OF_LEUKOCYTE_ADHESION_TO_VASCULAR_ENDOTHELIAL_CELL | Immunity | 0.82631 | 0.70069 | 0.84921 | -1.62707 | 0.01989 | 0.05833 | -0.83599 | 0.70786 | 0.82851 |
| GOBP_POSITIVE_REGULATION_OF_CELL_ADHESION_MEDIATED_BY_INTEGRIN | ECM and metastasis | 1.45033 | 0.07693 | 0.35296 | -1.80775 | 0.00639 | 0.02447 | -1.55582 | 0.04063 | 0.22357 |
| GOBP_LEUKOCYTE_APOPTOTIC_PROCESS | Immunity | -1.05757 | 0.35783 | 0.69038 | -1.81676 | 0.00004 | 0.00065 | -0.98840 | 0.48652 | 0.72962 |
| GOBP_LIPID_STORAGE | Metabolism and energy | -0.90755 | 0.63571 | 0.82645 | -1.80894 | 0.00014 | 0.00135 | -0.74979 | 0.94745 | 0.87060 |
| GOBP_PATTERN_RECOGNITION_RECEPTOR_SIGNALING_PATHWAY | Immunity | -0.94125 | 0.59935 | 0.80952 | -2.33409 | 0.00013 | 0.00130 | 0.57876 | 0.99831 | 0.87060 |
| GOBP_NEGATIVE_REGULATION_OF_INTERLEUKIN_10_PRODUCTION | Immunity | -0.90978 | 0.59565 | 0.80693 | -1.37482 | 0.08691 | 0.17135 | -1.38851 | 0.08947 | 0.33928 |
| GOBP_POSITIVE_REGULATION_OF_NATURAL_KILLER_CELL_MEDIATED_CYTOTOXICITY | Immunity | -1.72261 | 0.00510 | 0.06857 | 0.95667 | 0.54728 | 0.57797 | -0.77266 | 0.80764 | 0.86320 |
| GOBP_T_CELL_RECEPTOR_SIGNALING_PATHWAY | Immunity | -1.17210 | 0.18321 | 0.53385 | -2.02767 | 0.00005 | 0.00071 | -1.43343 | 0.00915 | 0.09713 |
| GOBP_POSITIVE_REGULATION_OF_HUMORAL_IMMUNE_RESPONSE | Immunity | -1.27581 | 0.16398 | 0.50396 | 0.97501 | 0.52047 | 0.56137 | -1.03457 | 0.38806 | 0.67109 |
| GOBP_MATURE_B_CELL_DIFFERENTIATION | Immunity | -0.77757 | 0.80518 | 0.88169 | -1.57712 | 0.01430 | 0.04517 | -1.02958 | 0.38766 | 0.67097 |
| GOBP_NATURAL_KILLER_CELL_MEDIATED_IMMUNITY | Immunity | -1.41971 | 0.03754 | 0.24004 | -0.96201 | 0.55415 | 0.58197 | 0.69616 | 0.92534 | 0.87060 |
| GOBP_NEGATIVE_REGULATION_OF_PRODUCTION_OF_MOLECULAR_MEDIATOR_OF_IMMUNE_RESPONSE | Immunity | -1.09080 | 0.33168 | 0.66776 | -1.33157 | 0.06394 | 0.13730 | 0.89271 | 0.65229 | 0.80954 |
| GOBP_POSITIVE_REGULATION_OF_MYELOID_LEUKOCYTE_DIFFERENTIATION | Immunity | -0.93898 | 0.56638 | 0.79753 | -1.41386 | 0.02674 | 0.07265 | -1.36445 | 0.05653 | 0.26871 |
| GOBP_CHRONIC_INFLAMMATORY_RESPONSE | Immunity | 1.02199 | 0.41880 | 0.72781 | -2.45888 | 0.00003 | 0.00060 | -1.74048 | 0.01721 | 0.13823 |
| GOBP_POSITIVE_REGULATION_OF_T_HELPER_CELL_DIFFERENTIATION | Immunity | 0.94432 | 0.52426 | 0.78003 | -1.85882 | 0.00380 | 0.01636 | 0.60610 | 0.94426 | 0.87060 |
| GOBP_REGULATION_OF_SPROUTING_ANGIOGENESIS | ECM and metastasis | 1.17416 | 0.22101 | 0.58131 | 1.07858 | 0.36898 | 0.45955 | 1.15931 | 0.25871 | 0.55900 |
| GOBP_INTERLEUKIN_17_PRODUCTION | Immunity | -0.99921 | 0.46643 | 0.75763 | -1.16480 | 0.19948 | 0.30580 | 0.71379 | 0.87885 | 0.87060 |
| GOBP_ANGIOGENESIS_INVOLVED_IN_WOUND_HEALING | ECM and metastasis | -0.96044 | 0.52450 | 0.78003 | -1.55645 | 0.02683 | 0.07279 | -1.04335 | 0.37394 | 0.66042 |
| GOBP_SPHINGOLIPID_BIOSYNTHETIC_PROCESS | Metabolism and energy | -0.88351 | 0.69525 | 0.84619 | 0.91260 | 0.66867 | 0.64015 | 0.79419 | 0.84630 | 0.87060 |
| GOBP_CELL_SUBSTRATE_ADHESION | ECM and metastasis | 1.77420 | 0.00001 | 0.00047 | -2.47623 | 0.00205 | 0.01007 | 1.24344 | 0.02841 | 0.18306 |
| GOBP_REGULATION_OF_LIPID_KINASE_ACTIVITY | Metabolism and energy | -0.96890 | 0.51651 | 0.77653 | -1.47940 | 0.01209 | 0.03986 | 0.76178 | 0.85318 | 0.87060 |
| GOBP_PHOSPHOLIPID_DEPHOSPHORYLATION | Metabolism and energy | -0.68681 | 0.92324 | 0.88692 | 0.56183 | 0.98259 | 0.72077 | 0.48131 | 0.99706 | 0.87060 |
| GOBP_REGULATION_OF_LEUKOCYTE_TETHERING_OR_ROLLING | Immunity | 1.36073 | 0.13047 | 0.45171 | -1.52428 | 0.04796 | 0.11103 | 0.81042 | 0.73347 | 0.83705 |
| GOBP_PEPTIDE_CATABOLIC_PROCESS | Metabolism and energy | -1.05828 | 0.38834 | 0.70940 | -1.46239 | 0.04366 | 0.10392 | 1.18209 | 0.24330 | 0.54148 |
| GOBP_NEGATIVE_REGULATION_OF_CELL_MATRIX_ADHESION | ECM and metastasis | 1.07994 | 0.33318 | 0.66850 | -0.98284 | 0.48404 | 0.53885 | -0.89631 | 0.63219 | 0.80102 |
| GOBP_REGULATION_OF_CELL_MATRIX_ADHESION | ECM and metastasis | 1.43642 | 0.01546 | 0.13975 | -1.79729 | 0.00009 | 0.00100 | 0.68549 | 0.95801 | 0.87060 |
| GOBP_ENDOTHELIAL_CELL_APOPTOTIC_PROCESS | ECM and metastasis | 1.19313 | 0.18262 | 0.53309 | -1.84557 | 0.00032 | 0.00244 | 1.12168 | 0.29621 | 0.59560 |
| GOBP_POSITIVE_REGULATION_OF_CD4_POSITIVE_ALPHA_BETA_T_CELL_DIFFERENTIATION | Immunity | 0.75432 | 0.83470 | 0.88692 | -2.15600 | 0.00015 | 0.00147 | -1.09432 | 0.29674 | 0.59561 |
| GOBP_VASCULAR_ENDOTHELIAL_GROWTH_FACTOR_SIGNALING_PATHWAY | ECM and metastasis | 1.07974 | 0.32754 | 0.66505 | -1.06439 | 0.33657 | 0.43416 | 1.20231 | 0.20549 | 0.50185 |
| GOBP_POSITIVE_REGULATION_OF_EXTRACELLULAR_MATRIX_ORGANIZATION | ECM and metastasis | -1.02836 | 0.42833 | 0.73270 | -1.53738 | 0.02839 | 0.07582 | 1.32651 | 0.11136 | 0.37764 |
| GOBP_INTEGRIN_MEDIATED_SIGNALING_PATHWAY | ECM and metastasis | 1.79663 | 0.00021 | 0.00632 | -2.96566 | 0.00004 | 0.00065 | 1.03501 | 0.42418 | 0.69646 |
| GOBP_NEGATIVE_REGULATION_OF_IMMUNE_RESPONSE | Immunity | -1.12749 | 0.22441 | 0.58344 | -1.82162 | 0.00012 | 0.00124 | 0.70703 | 0.96184 | 0.87060 |
| GOBP_REGULATION_OF_MYELOID_LEUKOCYTE_MEDIATED_IMMUNITY | Immunity | -1.32083 | 0.08987 | 0.38249 | -2.13994 | 0.00004 | 0.00069 | -1.11874 | 0.24028 | 0.53887 |
| GOBP_INTERLEUKIN_1_PRODUCTION | Immunity | -1.21131 | 0.14692 | 0.47712 | -2.02868 | 0.00004 | 0.00062 | -1.15161 | 0.15813 | 0.44555 |
| GOBP_MATURE_B_CELL_DIFFERENTIATION_INVOLVED_IN_IMMUNE_RESPONSE | Immunity | 0.85058 | 0.68739 | 0.84324 | -1.50952 | 0.02891 | 0.07667 | -1.04111 | 0.37211 | 0.65793 |
| GOBP_NEGATIVE_REGULATION_OF_LIPID_STORAGE | Metabolism and energy | -1.23465 | 0.19957 | 0.55652 | -1.54922 | 0.03308 | 0.08473 | -0.82239 | 0.72601 | 0.83517 |
| GOBP_TRANSFORMING_GROWTH_FACTOR_BETA_RECEPTOR_SIGNALING_PATHWAY | ECM and metastasis | 1.29752 | 0.02828 | 0.20614 | -1.65541 | 0.00019 | 0.00170 | 1.10533 | 0.25867 | 0.55900 |
| GOBP_NEGATIVE_REGULATION_OF_LEUKOCYTE_MIGRATION | Immunity | 1.34434 | 0.08414 | 0.36995 | -2.21700 | 0.00002 | 0.00056 | 1.00538 | 0.47570 | 0.72645 |
| GOBP_POSITIVE_REGULATION_OF_PATTERN_RECOGNITION_RECEPTOR_SIGNALING_PATHWAY | Immunity | 0.80935 | 0.78249 | 0.87471 | -2.36214 | 0.00001 | 0.00043 | -1.03587 | 0.37574 | 0.66229 |
| GOBP_EXTRACELLULAR_MATRIX_DISASSEMBLY | ECM and metastasis | 2.14368 | 0.00002 | 0.00082 | -2.53700 | 0.00001 | 0.00047 | 1.20843 | 0.18150 | 0.47549 |
| GOBP_CD4_POSITIVE_OR_CD8_POSITIVE_ALPHA_BETA_T_CELL_LINEAGE_COMMITMENT | Immunity | -1.06108 | 0.38881 | 0.70940 | -0.88089 | 0.65198 | 0.63237 | -1.16533 | 0.23130 | 0.53059 |
| GOBP_REGULATION_OF_CELL_SUBSTRATE_ADHESION | ECM and metastasis | 1.74773 | 0.00003 | 0.00139 | -2.21486 | 0.00021 | 0.00181 | 1.15926 | 0.15952 | 0.44764 |
| GOBP_REGULATION_OF_CELLULAR_RESPIRATION | Metabolism and energy | 1.01297 | 0.42580 | 0.73267 | -1.33180 | 0.06945 | 0.14537 | -1.19791 | 0.17320 | 0.46435 |
| GOBP_INTERFERON_ALPHA_PRODUCTION | Immunity | 0.89138 | 0.61385 | 0.81628 | -1.11893 | 0.27248 | 0.37826 | -0.94013 | 0.54122 | 0.76007 |
| GOBP_INFLAMMATORY_CELL_APOPTOTIC_PROCESS | Immunity | 1.05667 | 0.37431 | 0.69984 | -1.09009 | 0.31808 | 0.41870 | 0.67052 | 0.89590 | 0.87060 |
| GOBP_LIPID_IMPORT_INTO_CELL | Metabolism and energy | 1.02913 | 0.41096 | 0.72308 | 0.81661 | 0.73074 | 0.66571 | 1.21055 | 0.22625 | 0.52460 |
| GOBP_B_CELL_DIFFERENTIATION | Immunity | -0.74087 | 0.94813 | 0.88692 | 0.71056 | 0.95391 | 0.72077 | -1.08376 | 0.23864 | 0.53727 |
| GOBP_NATURAL_KILLER_CELL_ACTIVATION | Immunity | -1.28700 | 0.09135 | 0.38488 | 1.06426 | 0.37425 | 0.46376 | 0.86816 | 0.72694 | 0.83563 |
| GOBP_CELLULAR_RESPONSE_TO_VASCULAR_ENDOTHELIAL_GROWTH_FACTOR_STIMULUS | ECM and metastasis | 1.15022 | 0.22099 | 0.58131 | -1.44506 | 0.01821 | 0.05453 | 1.00877 | 0.47120 | 0.72371 |
| GOBP_LYMPHOCYTE_COSTIMULATION | Immunity | -1.19897 | 0.20295 | 0.56118 | -2.15029 | 0.00003 | 0.00061 | -1.62583 | 0.01275 | 0.11766 |
| GOBP_REGULATION_OF_MACROPHAGE_CHEMOTAXIS | Immunity | -0.87590 | 0.64936 | 0.83016 | -1.80994 | 0.00395 | 0.01688 | 1.27830 | 0.14782 | 0.43273 |
| GOBP_REGULATION_OF_HETEROTYPIC_CELL_CELL_ADHESION | ECM and metastasis | 1.62692 | 0.02430 | 0.18589 | -2.18555 | 0.00016 | 0.00148 | -1.33087 | 0.10783 | 0.36987 |
| GOBP_NEGATIVE_REGULATION_OF_T_CELL_PROLIFERATION | Immunity | -1.62446 | 0.00507 | 0.06839 | -1.36076 | 0.03469 | 0.08754 | 0.83675 | 0.75376 | 0.84431 |
| GOBP_FC_RECEPTOR_MEDIATED_STIMULATORY_SIGNALING_PATHWAY | Immunity | -1.12581 | 0.29760 | 0.64182 | -1.67201 | 0.00860 | 0.03085 | -1.67482 | 0.01473 | 0.12837 |
| GOBP_SPHINGOLIPID_METABOLIC_PROCESS | Metabolism and energy | -1.14966 | 0.19788 | 0.55392 | 0.83875 | 0.82575 | 0.70167 | 0.59965 | 0.99543 | 0.87060 |
| GOBP_INTERLEUKIN_8_PRODUCTION | Immunity | 0.84253 | 0.78847 | 0.87712 | -1.79772 | 0.00013 | 0.00134 | -0.80570 | 0.88111 | 0.87060 |
| GOBP_ORGAN_OR_TISSUE_SPECIFIC_IMMUNE_RESPONSE | Immunity | -1.98328 | 0.00011 | 0.00369 | 1.13347 | 0.30080 | 0.40429 | 1.39283 | 0.06863 | 0.29911 |
| GOBP_REGULATION_OF_CD8_POSITIVE_ALPHA_BETA_T_CELL_ACTIVATION | Immunity | -1.22968 | 0.21002 | 0.56852 | -1.14099 | 0.26587 | 0.37219 | -0.84071 | 0.68371 | 0.82159 |
| GOBP_REGULATION_OF_CELL_ADHESION_MEDIATED_BY_INTEGRIN | ECM and metastasis | 1.52866 | 0.02600 | 0.19535 | -1.99243 | 0.00013 | 0.00135 | -1.50759 | 0.02683 | 0.17639 |
| GOBP_REGULATION_OF_INFLAMMATORY_RESPONSE_TO_ANTIGENIC_STIMULUS | Immunity | -0.64386 | 0.94579 | 0.88692 | -1.00071 | 0.45096 | 0.52023 | 0.83716 | 0.72590 | 0.83517 |
| GOBP_POSITIVE_REGULATION_OF_CELL_SUBSTRATE_ADHESION | ECM and metastasis | 1.57947 | 0.00311 | 0.04873 | -2.51044 | 0.00005 | 0.00071 | 1.16564 | 0.19368 | 0.48932 |
| GOBP_RESPONSE_TO_INTERLEUKIN_6 | Immunity | -1.62835 | 0.01057 | 0.11107 | -1.17689 | 0.19754 | 0.30363 | 1.37224 | 0.07555 | 0.31184 |
| GOBP_BLOOD_VESSEL_REMODELING | ECM and metastasis | 1.06797 | 0.34414 | 0.67863 | -1.32546 | 0.07396 | 0.15151 | 0.89624 | 0.64306 | 0.80559 |
| GOBP_CELLULAR_RESPONSE_TO_INTERFERON_GAMMA | Immunity | -1.19340 | 0.16647 | 0.50722 | -2.29939 | 0.00003 | 0.00062 | -1.18457 | 0.12299 | 0.39616 |
| GOBP_REGULATION_OF_ENDOTHELIAL_CELL_CHEMOTAXIS | ECM and metastasis | 1.70001 | 0.01720 | 0.14960 | -1.46424 | 0.05360 | 0.12057 | 0.78309 | 0.77377 | 0.85281 |
| GOBP_LYMPHANGIOGENESIS | ECM and metastasis | -0.90276 | 0.60357 | 0.81193 | 1.13559 | 0.30922 | 0.41186 | 1.53815 | 0.02551 | 0.17261 |
| GOBP_REGULATION_OF_IMMUNOGLOBULIN_PRODUCTION | Immunity | -1.18945 | 0.19298 | 0.54873 | -1.21649 | 0.10513 | 0.19640 | -1.02697 | 0.38994 | 0.67155 |
| GOBP_CELL_DEATH_IN_RESPONSE_TO_OXIDATIVE_STRESS | Cell death | 1.00507 | 0.43559 | 0.73753 | -1.36161 | 0.02240 | 0.06355 | -1.24164 | 0.09114 | 0.34303 |
| GOBP_POSITIVE_REGULATION_OF_REGULATORY_T_CELL_DIFFERENTIATION | Immunity | -1.32504 | 0.12835 | 0.44835 | -1.46717 | 0.05508 | 0.12309 | -1.61358 | 0.03165 | 0.19550 |
| GOBP_POSITIVE_REGULATION_OF_CELL_MIGRATION_INVOLVED_IN_SPROUTING_ANGIOGENESIS | ECM and metastasis | 1.19747 | 0.23087 | 0.58977 | -1.89439 | 0.00330 | 0.01465 | 0.98782 | 0.49906 | 0.73795 |
| GOBP_IMMUNE_RESPONSE_TO_TUMOR_CELL | Immunity | -1.07002 | 0.37996 | 0.70230 | -1.00705 | 0.43473 | 0.50948 | 1.05763 | 0.40512 | 0.68539 |
| GOBP_REGULATION_OF_GLUCOSE_IMPORT | Metabolism and energy | 1.04444 | 0.37072 | 0.69724 | 1.03171 | 0.43814 | 0.51222 | 0.98730 | 0.50528 | 0.74081 |
| GOBP_POSITIVE_REGULATION_OF_NATURAL_KILLER_CELL_ACTIVATION | Immunity | 0.95811 | 0.50473 | 0.77250 | -0.99289 | 0.45922 | 0.52367 | -0.87957 | 0.63641 | 0.80282 |
| GOBP_CYTOKINE_MEDIATED_SIGNALING_PATHWAY | Immunity | -1.05184 | 0.33105 | 0.66730 | -1.81974 | 0.01111 | 0.03740 | 0.77719 | 0.97022 | 0.87060 |
| GOBP_SOMATIC_DIVERSIFICATION_OF_IMMUNOGLOBULINS_INVOLVED_IN_IMMUNE_RESPONSE | Immunity | -0.89354 | 0.63902 | 0.82706 | 0.53506 | 0.98986 | 0.72077 | -0.73649 | 0.91701 | 0.87060 |
| GOBP_POSITIVE_REGULATION_OF_GLUCOSE_TRANSMEMBRANE_TRANSPORT | Metabolism and energy | -0.90516 | 0.61611 | 0.81645 | 0.88882 | 0.66097 | 0.63678 | 0.98682 | 0.50349 | 0.73923 |
| GOBP_REGULATION_OF_INFLAMMATORY_RESPONSE | Immunity | -1.05643 | 0.32782 | 0.66505 | -2.04731 | 0.00204 | 0.01006 | -1.11203 | 0.09735 | 0.35154 |
| GOBP_POSITIVE_REGULATION_OF_INTERLEUKIN_8_PRODUCTION | Immunity | 0.95549 | 0.53084 | 0.78440 | -1.72299 | 0.00127 | 0.00695 | 0.51439 | 0.99618 | 0.87060 |
| GOBP_LYMPHOCYTE_CHEMOTAXIS | Immunity | -1.26882 | 0.12872 | 0.44835 | -2.14389 | 0.00003 | 0.00060 | -1.24575 | 0.11648 | 0.38637 |
| GOBP_MACROPHAGE_DIFFERENTIATION | Immunity | 1.49219 | 0.03383 | 0.22826 | -2.16380 | 0.00003 | 0.00061 | 0.94145 | 0.57656 | 0.77629 |
| GOBP_POSITIVE_REGULATION_OF_INTERLEUKIN_17_PRODUCTION | Immunity | -0.99899 | 0.46970 | 0.76002 | -1.13246 | 0.25874 | 0.36590 | -1.15404 | 0.23691 | 0.53480 |
| GOBP_NEGATIVE_REGULATION_OF_CELL_SUBSTRATE_ADHESION | ECM and metastasis | 1.43794 | 0.03965 | 0.24659 | -1.26347 | 0.08481 | 0.16799 | 1.17686 | 0.22056 | 0.51985 |
| GOBP_RESPONSE_TO_INTERFERON_GAMMA | Immunity | -1.11507 | 0.25917 | 0.61128 | -2.44226 | 0.00005 | 0.00073 | -1.25503 | 0.05554 | 0.26691 |
| GOBP_CARBOHYDRATE_DERIVATIVE_CATABOLIC_PROCESS | Metabolism and energy | -1.09997 | 0.27036 | 0.61914 | -1.56876 | 0.00032 | 0.00244 | -0.79583 | 0.96577 | 0.87060 |
| GOBP_POSITIVE_REGULATION_OF_ANTIGEN_RECEPTOR_MEDIATED_SIGNALING_PATHWAY | Immunity | -0.77519 | 0.78284 | 0.87480 | -1.53987 | 0.03179 | 0.08228 | 0.84384 | 0.69859 | 0.82593 |
| GOBP_MONOCYTE_CHEMOTACTIC_PROTEIN_1_PRODUCTION | Immunity | 1.43130 | 0.08682 | 0.37553 | -2.35561 | 0.00005 | 0.00071 | -0.68087 | 0.90500 | 0.87060 |
| GOBP_POSITIVE_REGULATION_OF_IMMUNOGLOBULIN_PRODUCTION | Immunity | -1.18716 | 0.21253 | 0.57179 | -1.22102 | 0.12845 | 0.22655 | -1.22919 | 0.13910 | 0.42081 |
| GOBP_REGULATION_OF_T_HELPER_17_CELL_DIFFERENTIATION | Immunity | -0.65628 | 0.90608 | 0.88692 | -1.14834 | 0.25235 | 0.35951 | -0.85440 | 0.67080 | 0.81579 |
| GOBP_INTEGRIN_ACTIVATION | ECM and metastasis | 1.42166 | 0.08150 | 0.36316 | -1.87361 | 0.00311 | 0.01404 | -1.34093 | 0.10274 | 0.36260 |
| GOBP_NEGATIVE_REGULATION_OF_EPITHELIAL_TO_MESENCHYMAL_TRANSITION | ECM and metastasis | -1.11551 | 0.30923 | 0.65368 | 0.82050 | 0.74777 | 0.67301 | 1.04433 | 0.41806 | 0.69243 |
| GOBP_POSITIVE_REGULATION_OF_CELLULAR_RESPONSE_TO_TRANSFORMING_GROWTH_FACTOR_BETA_STIMULUS | ECM and metastasis | -1.09404 | 0.33904 | 0.67314 | 0.88054 | 0.66178 | 0.63721 | 0.82562 | 0.73317 | 0.83705 |
| GOBP_VERY_LONG_CHAIN_FATTY_ACID_METABOLIC_PROCESS | Metabolism and energy | -0.53344 | 0.98846 | 0.88692 | 0.91878 | 0.60920 | 0.61110 | 0.63816 | 0.93764 | 0.87060 |
| GOBP_RESPONSE_TO_INTERLEUKIN_1 | Immunity | -1.24155 | 0.10858 | 0.41470 | -2.03004 | 0.00005 | 0.00074 | -1.57575 | 0.00139 | 0.02991 |
| GOBP_POSITIVE_REGULATION_OF_GRANULOCYTE_CHEMOTAXIS | Immunity | 1.37818 | 0.09707 | 0.39707 | -2.08600 | 0.00043 | 0.00307 | -1.28368 | 0.13005 | 0.40670 |
| GOBP_INFLAMMASOME_COMPLEX_ASSEMBLY | Cell death | -0.94616 | 0.54492 | 0.78948 | -1.06830 | 0.34486 | 0.44068 | -0.95244 | 0.51694 | 0.74581 |
| GOBP_POSITIVE_REGULATION_OF_T_HELPER_1_TYPE_IMMUNE_RESPONSE | Immunity | -0.84248 | 0.68630 | 0.84324 | -1.35169 | 0.10495 | 0.19628 | -1.08418 | 0.32607 | 0.61917 |
| GOBP_POSITIVE_REGULATION_OF_B_CELL_MEDIATED_IMMUNITY | Immunity | -1.14317 | 0.27213 | 0.61963 | -0.94118 | 0.56966 | 0.59085 | -0.83501 | 0.75323 | 0.84411 |
| GOBP_HYALURONAN_METABOLIC_PROCESS | ECM and metastasis | 0.96191 | 0.50728 | 0.77250 | -1.51685 | 0.02442 | 0.06778 | 0.82619 | 0.73597 | 0.83705 |
| GOBP_NEGATIVE_REGULATION_OF_INFLAMMATORY_RESPONSE | Immunity | -0.99276 | 0.47993 | 0.76283 | -1.49191 | 0.00141 | 0.00756 | 0.74490 | 0.92076 | 0.87060 |
| GOBP_MACROPHAGE_CHEMOTAXIS | Immunity | -1.41375 | 0.05950 | 0.30840 | -1.53682 | 0.01836 | 0.05491 | 1.17442 | 0.24178 | 0.54000 |
| GOBP_REGULATION_OF_ENDOTHELIAL_CELL_DEVELOPMENT | ECM and metastasis | -1.22198 | 0.21584 | 0.57507 | 1.01779 | 0.46160 | 0.52475 | 0.73250 | 0.82805 | 0.86979 |
| GOBP_HOMOTYPIC_CELL_CELL_ADHESION | ECM and metastasis | 1.92987 | 0.00007 | 0.00255 | -2.12344 | 0.00003 | 0.00060 | -1.44882 | 0.01534 | 0.13133 |
| GOBP_MACROPHAGE_MIGRATION | Immunity | -1.14547 | 0.25383 | 0.60680 | -1.96771 | 0.00008 | 0.00097 | 1.04623 | 0.41005 | 0.68869 |
| GOBP_REGULATION_OF_NATURAL_KILLER_CELL_ACTIVATION | Immunity | -0.87125 | 0.66774 | 0.83543 | 0.77394 | 0.81302 | 0.69780 | -1.07414 | 0.31772 | 0.61238 |
| GOBP_NEGATIVE_REGULATION_OF_CYTOKINE_PRODUCTION | Immunity | -1.06905 | 0.30937 | 0.65369 | -1.92949 | 0.00059 | 0.00391 | 0.80991 | 0.90294 | 0.87060 |
| GOBP_SCF_DEPENDENT_PROTEASOMAL_UBIQUITIN_DEPENDENT_PROTEIN_CATABOLIC_PROCESS | Immunity | -0.90165 | 0.62408 | 0.82042 | 1.19214 | 0.21110 | 0.31832 | 1.25520 | 0.14759 | 0.43263 |
| GOBP_NEGATIVE_REGULATION_OF_T_CELL_APOPTOTIC_PROCESS | Immunity | -1.67157 | 0.00872 | 0.09902 | -1.54308 | 0.02989 | 0.07892 | -1.02935 | 0.39470 | 0.67638 |
| GOBP_REGULATION_OF_CELL_JUNCTION_ASSEMBLY | ECM and metastasis | 1.36240 | 0.01453 | 0.13368 | 0.98585 | 0.53925 | 0.57324 | 1.46826 | 0.00156 | 0.03133 |
| GOBP_POSITIVE_REGULATION_OF_GLUCOSE_IMPORT | Metabolism and energy | -0.79850 | 0.77331 | 0.87083 | 1.02208 | 0.45433 | 0.52208 | 1.03432 | 0.43222 | 0.70113 |
| GOBP_POSITIVE_REGULATION_OF_CELL_MATRIX_ADHESION | ECM and metastasis | 1.30050 | 0.10183 | 0.40384 | -2.14941 | 0.00003 | 0.00059 | 0.92199 | 0.61207 | 0.79295 |
| GOBP_CELLULAR_RESPONSE_TO_INTERLEUKIN_1 | Immunity | -1.25208 | 0.11226 | 0.41977 | -2.17449 | 0.00003 | 0.00061 | -1.50265 | 0.00727 | 0.08442 |
| GOBP_LYMPHOCYTE_APOPTOTIC_PROCESS | Immunity | -1.46202 | 0.02342 | 0.18153 | -1.45088 | 0.01090 | 0.03693 | 0.70849 | 0.91964 | 0.87060 |
| GOBP_REGULATION_OF_LYMPHOCYTE_APOPTOTIC_PROCESS | Immunity | -1.72681 | 0.00153 | 0.02995 | -1.39382 | 0.02874 | 0.07641 | -0.81456 | 0.83474 | 0.87060 |
| GOBP_NEGATIVE_REGULATION_OF_INTERLEUKIN_2_PRODUCTION | Immunity | -1.70497 | 0.00565 | 0.07366 | -1.20544 | 0.18086 | 0.28671 | 0.87020 | 0.66705 | 0.81408 |
| GOBP_HUMORAL_IMMUNE_RESPONSE | Immunity | -1.60773 | 0.00034 | 0.00931 | -1.82769 | 0.00022 | 0.00191 | 1.23332 | 0.06888 | 0.29963 |
| GOBP_POSITIVE_REGULATION_OF_MYELOID_LEUKOCYTE_MEDIATED_IMMUNITY | Immunity | 1.06206 | 0.37047 | 0.69724 | -2.16521 | 0.00037 | 0.00271 | 0.67176 | 0.89205 | 0.87060 |
| GOBP_REGULATION_OF_T_HELPER_17_TYPE_IMMUNE_RESPONSE | Immunity | -0.74180 | 0.83887 | 0.88692 | -1.38371 | 0.06544 | 0.13981 | 0.62203 | 0.94357 | 0.87060 |
| GOBP_REGULATION_OF_T_CELL_APOPTOTIC_PROCESS | Immunity | -1.71123 | 0.00354 | 0.05309 | -1.35551 | 0.06197 | 0.13403 | 0.74755 | 0.84396 | 0.87060 |
| GOBP_REGULATION_OF_LIPID_LOCALIZATION | Metabolism and energy | -1.01891 | 0.42555 | 0.73267 | -1.38249 | 0.00396 | 0.01688 | 1.13093 | 0.22752 | 0.52617 |
| GOBP_POSITIVE_REGULATION_OF_TRANSCRIPTION_FROM_RNA_POLYMERASE_II_PROMOTER_INVOLVED_IN_CELLULAR_RESPONSE_TO_CHEMICAL_STIMULUS | Genetic and epigenetic information | -0.87969 | 0.63778 | 0.82706 | 1.08578 | 0.36929 | 0.45982 | -0.79605 | 0.76629 | 0.84885 |
| GOBP_PROTEIN_MATURATION | Metabolism and energy | 1.51658 | 0.00030 | 0.00826 | -1.62238 | 0.00123 | 0.00679 | 0.92217 | 0.71740 | 0.83274 |
| GOBP_NEGATIVE_REGULATION_OF_TUMOR_NECROSIS_FACTOR_SUPERFAMILY_CYTOKINE_PRODUCTION | Immunity | 1.28043 | 0.10581 | 0.41056 | -2.17337 | 0.00002 | 0.00047 | 0.91942 | 0.62109 | 0.79729 |
| GOBP_REGULATION_OF_LEUKOCYTE_DEGRANULATION | Immunity | -1.32602 | 0.09712 | 0.39707 | -2.23528 | 0.00002 | 0.00056 | -1.21693 | 0.15155 | 0.43729 |
| GOBP_LIPID_EXPORT_FROM_CELL | Metabolism and energy | 1.36427 | 0.07575 | 0.35131 | -1.28110 | 0.08864 | 0.17404 | 1.21141 | 0.19037 | 0.48587 |
| GOBP_POSITIVE_REGULATION_OF_ACTIVATED_T_CELL_PROLIFERATION | Immunity | -1.15677 | 0.27483 | 0.62213 | -1.51240 | 0.03758 | 0.09286 | -1.27194 | 0.14536 | 0.43022 |
| GOBP_REGULATION_OF_CELLULAR_RESPONSE_TO_TRANSFORMING_GROWTH_FACTOR_BETA_STIMULUS | ECM and metastasis | 1.35591 | 0.02701 | 0.19942 | -1.37348 | 0.00778 | 0.02863 | 1.34735 | 0.02811 | 0.18175 |
| GOBP_POSITIVE_REGULATION_OF_T_CELL_MEDIATED_CYTOTOXICITY | Immunity | -0.98517 | 0.49035 | 0.76857 | -0.88607 | 0.63637 | 0.62439 | 0.93720 | 0.56892 | 0.77202 |
| GOBP_LOW_DENSITY_LIPOPROTEIN_PARTICLE_REMODELING | Metabolism and energy | 1.38316 | 0.11856 | 0.43067 | -1.49722 | 0.05505 | 0.12308 | 1.20767 | 0.23038 | 0.52984 |
| GOBP_POSITIVE_REGULATION_OF_GLYCOLYTIC_PROCESS | Metabolism and energy | -0.60915 | 0.94109 | 0.88692 | 1.19419 | 0.23779 | 0.34543 | 1.12118 | 0.32334 | 0.61686 |
| GOBP_REGULATION_OF_MACROPHAGE_MIGRATION | Immunity | 1.04444 | 0.37746 | 0.70150 | -2.29297 | 0.00001 | 0.00043 | 0.99009 | 0.49836 | 0.73734 |
| GOBP_NEGATIVE_REGULATION_OF_ENDOTHELIAL_CELL_APOPTOTIC_PROCESS | ECM and metastasis | 1.16269 | 0.24342 | 0.59843 | -1.65701 | 0.01000 | 0.03447 | 0.91257 | 0.61113 | 0.79249 |
| GOBP_REGULATION_OF_PATTERN_RECOGNITION_RECEPTOR_SIGNALING_PATHWAY | Immunity | -0.94823 | 0.56394 | 0.79703 | -2.20504 | 0.00003 | 0.00061 | -1.23957 | 0.08090 | 0.32323 |
| GOBP_NEGATIVE_REGULATION_OF_LYMPHOCYTE_DIFFERENTIATION | Immunity | 0.97271 | 0.49434 | 0.76969 | -1.13212 | 0.21820 | 0.32550 | 1.32316 | 0.08490 | 0.33175 |
| GOBP_INNATE_IMMUNE_RESPONSE_ACTIVATING_SIGNAL_TRANSDUCTION | Immunity | -1.37263 | 0.08899 | 0.38154 | 0.79332 | 0.77442 | 0.68460 | 0.97873 | 0.51292 | 0.74380 |
| GOBP_INTERLEUKIN_6_MEDIATED_SIGNALING_PATHWAY | Immunity | -1.23634 | 0.20408 | 0.56316 | 0.72192 | 0.83960 | 0.70634 | 1.11829 | 0.33052 | 0.62319 |
| GOBP_RESPONSE_TO_FATTY_ACID | Metabolism and energy | 1.11907 | 0.25952 | 0.61165 | -1.58256 | 0.00548 | 0.02164 | 1.16458 | 0.23458 | 0.53298 |
| GOBP_NEGATIVE_REGULATION_OF_NATURAL_KILLER_CELL_MEDIATED_IMMUNITY | Immunity | -0.88792 | 0.62278 | 0.81990 | -1.63544 | 0.02761 | 0.07412 | 1.31109 | 0.13727 | 0.41810 |
| GOBP_MEMBRANE_LIPID_METABOLIC_PROCESS | Metabolism and energy | -1.11860 | 0.23156 | 0.58981 | 0.69736 | 0.97451 | 0.72077 | 0.50575 | 0.99997 | 0.87060 |
| GOBP_REGULATION_OF_OXIDATIVE_STRESS_INDUCED_CELL_DEATH | Cell death | -0.86605 | 0.70181 | 0.84944 | -1.32322 | 0.04553 | 0.10705 | -1.25970 | 0.09720 | 0.35126 |
| GOBP_AMINO_ACID_IMPORT_ACROSS_PLASMA_MEMBRANE | Metabolism and energy | 0.74823 | 0.88397 | 0.88692 | 1.02475 | 0.44967 | 0.51920 | -0.94159 | 0.56193 | 0.76847 |
| GOBP_POSITIVE_REGULATION_OF_LYMPHOCYTE_CHEMOTAXIS | Immunity | -1.64100 | 0.01264 | 0.12311 | 0.75927 | 0.80350 | 0.69448 | -1.66045 | 0.02506 | 0.17138 |
| GOBP_NEGATIVE_REGULATION_OF_CELL_CYCLE_G1_S_PHASE_TRANSITION | Cell cycle | -1.25702 | 0.12436 | 0.44081 | 0.83059 | 0.77823 | 0.68621 | -0.86679 | 0.76024 | 0.84603 |
| GOBP_REGULATION_OF_LIPID_TRANSPORT | Metabolism and energy | -0.99239 | 0.48018 | 0.76283 | -1.14684 | 0.12210 | 0.21828 | 1.15567 | 0.20119 | 0.49590 |
| GOBP_PLATELET_DERIVED_GROWTH_FACTOR_RECEPTOR_SIGNALING_PATHWAY | ECM and metastasis | 1.67688 | 0.00649 | 0.08146 | -1.91398 | 0.00018 | 0.00164 | 0.94842 | 0.56933 | 0.77207 |
| GOBP_POLYSACCHARIDE_METABOLIC_PROCESS | Metabolism and energy | 1.31083 | 0.05918 | 0.30823 | -1.03334 | 0.37355 | 0.46345 | 0.90492 | 0.66988 | 0.81578 |
| GOBP_REGULATORY_T_CELL_DIFFERENTIATION | Immunity | -0.95512 | 0.53295 | 0.78542 | 0.56752 | 0.97398 | 0.72077 | -1.26177 | 0.13498 | 0.41446 |
| GOBP_NEGATIVE_REGULATION_OF_CYTOKINE_PRODUCTION_INVOLVED_IN_IMMUNE_RESPONSE | Immunity | -0.85715 | 0.68478 | 0.84238 | -1.40924 | 0.05049 | 0.11518 | 0.90948 | 0.61737 | 0.79533 |
| GOBP_IMMUNE_RESPONSE_REGULATING_CELL_SURFACE_RECEPTOR_SIGNALING_PATHWAY_INVOLVED_IN_PHAGOCYTOSIS | Immunity | -0.81561 | 0.73389 | 0.86010 | -1.48573 | 0.04002 | 0.09711 | -1.19820 | 0.19474 | 0.48960 |
| GOBP_T_CELL_CHEMOTAXIS | Immunity | -0.85069 | 0.68287 | 0.84172 | -1.33341 | 0.09757 | 0.18633 | -1.23935 | 0.16374 | 0.45237 |
| GOBP_POSITIVE_REGULATION_OF_AUTOPHAGY | Cell death | -0.87459 | 0.72975 | 0.85849 | -1.08289 | 0.23532 | 0.34304 | -1.00229 | 0.45393 | 0.71382 |
| GOBP_NEUTROPHIL_MEDIATED_IMMUNITY | Immunity | -1.78261 | 0.00166 | 0.03143 | -1.59370 | 0.01268 | 0.04133 | -1.08742 | 0.30046 | 0.59979 |
| GOBP_POSITIVE_REGULATION_OF_LIPID_KINASE_ACTIVITY | Metabolism and energy | -0.73639 | 0.85458 | 0.88692 | -1.50891 | 0.02481 | 0.06847 | 0.91640 | 0.60804 | 0.79084 |
| GOBP_REGULATION_OF_AMINO_ACID_TRANSPORT | Metabolism and energy | -1.04572 | 0.39619 | 0.71285 | 1.50393 | 0.01675 | 0.05119 | -1.39907 | 0.05664 | 0.26885 |
| GOBP_LEUKOCYTE_MIGRATION_INVOLVED_IN_INFLAMMATORY_RESPONSE | Immunity | -0.77531 | 0.77630 | 0.87259 | -2.20254 | 0.00026 | 0.00214 | -1.58998 | 0.03666 | 0.21141 |
| GOBP_POSITIVE_REGULATION_OF_INNATE_IMMUNE_RESPONSE | Immunity | -1.12526 | 0.24277 | 0.59793 | 0.68366 | 0.96296 | 0.72077 | -0.93416 | 0.65361 | 0.81053 |
| GOBP_NEGATIVE_REGULATION_OF_LEUKOCYTE_APOPTOTIC_PROCESS | Immunity | -1.31545 | 0.09794 | 0.39941 | -1.96306 | 0.00010 | 0.00113 | -0.98511 | 0.47132 | 0.72371 |
| GOBP_POSITIVE_REGULATION_OF_INTERFERON_ALPHA_PRODUCTION | Immunity | 0.77190 | 0.77910 | 0.87374 | 0.79432 | 0.76488 | 0.68044 | -0.80053 | 0.76300 | 0.84720 |
| GOBP_BLOOD_VESSEL_ENDOTHELIAL_CELL_PROLIFERATION_INVOLVED_IN_SPROUTING_ANGIOGENESIS | ECM and metastasis | 1.52133 | 0.04837 | 0.27695 | -1.34025 | 0.09602 | 0.18456 | 1.71112 | 0.00324 | 0.05043 |
| GOBP_NEGATIVE_REGULATION_OF_LIPID_LOCALIZATION | Metabolism and energy | -1.22626 | 0.17732 | 0.52789 | -1.12908 | 0.23642 | 0.34403 | 0.67098 | 0.92518 | 0.87060 |
| GOBP_REGULATION_OF_ANTIGEN_PROCESSING_AND_PRESENTATION | Immunity | 0.79214 | 0.73572 | 0.86010 | -2.93655 | 0.00001 | 0.00034 | -1.49780 | 0.05828 | 0.27311 |
| GOBP_T_CELL_APOPTOTIC_PROCESS | Immunity | -1.37694 | 0.06305 | 0.31878 | -1.38427 | 0.03568 | 0.08956 | 1.02478 | 0.44462 | 0.70790 |
| GOBP_ANTIGEN_PROCESSING_AND_PRESENTATION_OF_ENDOGENOUS_ANTIGEN | Immunity | -1.60748 | 0.01817 | 0.15424 | -2.13202 | 0.00063 | 0.00416 | 0.88956 | 0.63157 | 0.80088 |
| GOBP_REGULATION_OF_INNATE_IMMUNE_RESPONSE | Immunity | -1.14774 | 0.18078 | 0.53014 | -1.33392 | 0.00322 | 0.01438 | -0.99400 | 0.48684 | 0.72974 |
| GOBP_INTERLEUKIN_5_PRODUCTION | Immunity | -1.47262 | 0.05063 | 0.28347 | -1.31406 | 0.11803 | 0.21355 | 0.92888 | 0.58036 | 0.77722 |
| GOBP_NEGATIVE_REGULATION_OF_CYTOKINE_PRODUCTION_INVOLVED_IN_INFLAMMATORY_RESPONSE | Immunity | 0.61903 | 0.94382 | 0.88692 | -1.75163 | 0.00750 | 0.02777 | -0.79998 | 0.77222 | 0.85203 |
| GOBP_POSITIVE_REGULATION_OF_ATP_METABOLIC_PROCESS | Metabolism and energy | 0.76203 | 0.82440 | 0.88692 | 0.86874 | 0.67975 | 0.64364 | 1.00606 | 0.47328 | 0.72581 |
| GOBP_NEGATIVE_REGULATION_OF_STEROID_METABOLIC_PROCESS | Metabolism and energy | -1.15934 | 0.26638 | 0.61716 | 0.70209 | 0.87426 | 0.71760 | -1.40776 | 0.07293 | 0.30713 |
| GOBP_POSITIVE_REGULATION_OF_RESPONSE_TO_CYTOKINE_STIMULUS | Immunity | -0.94496 | 0.55775 | 0.79537 | -1.61489 | 0.00348 | 0.01519 | -1.15765 | 0.19005 | 0.48572 |
| GOBP_PHOSPHATIDYLINOSITOL_BIOSYNTHETIC_PROCESS | Metabolism and energy | -1.07818 | 0.31826 | 0.65974 | 0.63684 | 0.98211 | 0.72077 | 0.40673 | 1.00000 | 0.87060 |
| GOBP_MEMBRANE_LIPID_BIOSYNTHETIC_PROCESS | Metabolism and energy | -0.92024 | 0.63565 | 0.82645 | 0.79294 | 0.87710 | 0.71850 | 0.62277 | 0.98976 | 0.87060 |
| GOBP_REGULATION_OF_CELLULAR_RESPONSE_TO_VASCULAR_ENDOTHELIAL_GROWTH_FACTOR_STIMULUS | ECM and metastasis | 1.25816 | 0.17807 | 0.52833 | 0.91080 | 0.61170 | 0.61218 | 0.99502 | 0.48900 | 0.73125 |
| GOBP_POSITIVE_REGULATION_OF_HETEROTYPIC_CELL_CELL_ADHESION | ECM and metastasis | 1.62884 | 0.03466 | 0.23192 | -2.13649 | 0.00089 | 0.00530 | 0.88136 | 0.64087 | 0.80463 |
| GOBP_GLYCOSPHINGOLIPID_METABOLIC_PROCESS | Metabolism and energy | 0.76138 | 0.88784 | 0.88692 | 0.93839 | 0.59651 | 0.60471 | 0.83549 | 0.75147 | 0.84374 |
| GOBP_POSITIVE_REGULATION_OF_NUCLEOTIDE_METABOLIC_PROCESS | Genetic and epigenetic information | -0.82159 | 0.74908 | 0.86385 | 0.80244 | 0.78130 | 0.68692 | 0.75672 | 0.83579 | 0.87060 |
| GOBP_TRANSLATIONAL_ELONGATION | Metabolism and energy | 0.99569 | 0.45340 | 0.74892 | -1.35568 | 0.05766 | 0.12710 | -1.33566 | 0.08310 | 0.32820 |
| GOBP_POSITIVE_REGULATION_OF_DNA_BINDING_TRANSCRIPTION_FACTOR_ACTIVITY | Genetic and epigenetic information | -1.03628 | 0.38310 | 0.70547 | -1.38920 | 0.00046 | 0.00322 | 0.84317 | 0.85229 | 0.87060 |
| GOBP_GRANULOCYTE_ACTIVATION | Immunity | -1.38191 | 0.06743 | 0.33006 | -2.64748 | 0.00001 | 0.00043 | -1.38206 | 0.06037 | 0.27852 |
| GOBP_MYELOID_DENDRITIC_CELL_DIFFERENTIATION | Immunity | 0.88613 | 0.60686 | 0.81299 | -2.33284 | 0.00006 | 0.00085 | -0.94714 | 0.51970 | 0.74703 |
| GOBP_CARBOHYDRATE_TRANSMEMBRANE_TRANSPORT | Metabolism and energy | -0.85181 | 0.76707 | 0.86926 | 0.74120 | 0.91893 | 0.72077 | 1.04768 | 0.39783 | 0.67826 |
| GOBP_POSITIVE_REGULATION_OF_AMINO_ACID_TRANSPORT | Metabolism and energy | -1.09086 | 0.34806 | 0.68236 | 1.43774 | 0.04778 | 0.11071 | -1.12596 | 0.26679 | 0.56790 |
| GOBP_RECEPTOR_SIGNALING_PATHWAY_VIA_STAT | Immunity | -1.02783 | 0.40699 | 0.72132 | 1.03952 | 0.40938 | 0.49115 | 1.01512 | 0.46352 | 0.71900 |
| GOBP_MONOCYTE_DIFFERENTIATION | Immunity | -0.86065 | 0.67951 | 0.84067 | -1.96289 | 0.00066 | 0.00424 | 0.87363 | 0.66962 | 0.81578 |
| GOBP_PROTEIN_AUTOUBIQUITINATION | Immunity | -1.01480 | 0.43750 | 0.73890 | 0.70066 | 0.92730 | 0.72077 | -1.40120 | 0.02970 | 0.18868 |
| GOBP_VASCULAR_ENDOTHELIAL_GROWTH_FACTOR_RECEPTOR_SIGNALING_PATHWAY | ECM and metastasis | 1.27660 | 0.11925 | 0.43183 | 0.61181 | 0.96690 | 0.72077 | 1.14426 | 0.26738 | 0.56858 |
| GOBP_LONG_CHAIN_FATTY_ACID_TRANSPORT | Metabolism and energy | -0.97193 | 0.51132 | 0.77424 | -1.54964 | 0.00610 | 0.02360 | 1.11899 | 0.29463 | 0.59447 |
| GOBP_RESPONSE_TO_CARBOHYDRATE | Metabolism and energy | -1.01807 | 0.42645 | 0.73267 | 1.00297 | 0.49671 | 0.54652 | 1.00321 | 0.49254 | 0.73330 |
| GOBP_REGULATION_OF_LYMPHOCYTE_CHEMOTAXIS | Immunity | -1.65408 | 0.01017 | 0.10906 | -1.27749 | 0.12984 | 0.22775 | -1.74213 | 0.01369 | 0.12245 |
| GOBP_POSITIVE_REGULATION_OF_MACROAUTOPHAGY | Cell death | 0.61763 | 0.99144 | 0.88692 | -0.96974 | 0.53434 | 0.57026 | 0.71971 | 0.90406 | 0.87060 |
| GOBP_PYRIMIDINE_NUCLEOBASE_METABOLIC_PROCESS | Genetic and epigenetic information | -0.68560 | 0.87974 | 0.88692 | 1.48778 | 0.03536 | 0.08895 | -0.99736 | 0.44211 | 0.70647 |
| GOBP_CARBOHYDRATE_TRANSPORT | Metabolism and energy | 0.88966 | 0.75299 | 0.86441 | 0.75942 | 0.91516 | 0.72077 | 0.78801 | 0.87775 | 0.87060 |
| GOBP_NEGATIVE_REGULATION_OF_T_CELL_DIFFERENTIATION | Immunity | 0.85622 | 0.70573 | 0.85127 | -1.26591 | 0.10069 | 0.19045 | 1.28680 | 0.12110 | 0.39311 |
| GOBP_REGULATION_OF_B_CELL_APOPTOTIC_PROCESS | Immunity | -1.02481 | 0.43736 | 0.73890 | -0.94967 | 0.52826 | 0.56588 | -1.74586 | 0.01543 | 0.13135 |
| GOBP_ARGININE_METABOLIC_PROCESS | Metabolism and energy | -1.15059 | 0.28740 | 0.63473 | -1.09264 | 0.31672 | 0.41765 | 1.25931 | 0.17623 | 0.46700 |
| GOBP_POSITIVE_REGULATION_OF_CARBOHYDRATE_METABOLIC_PROCESS | Metabolism and energy | 0.94525 | 0.55951 | 0.79590 | 0.65831 | 0.95376 | 0.72077 | 1.02443 | 0.44481 | 0.70792 |
| GOBP_LIPID_LOCALIZATION | Metabolism and energy | -1.10606 | 0.20575 | 0.56555 | -1.20544 | 0.00962 | 0.03350 | 1.00116 | 0.50067 | 0.73858 |
| GOBP_GLYCOSPHINGOLIPID_BIOSYNTHETIC_PROCESS | Metabolism and energy | 1.07886 | 0.33566 | 0.67043 | 1.18757 | 0.22904 | 0.33689 | 1.11414 | 0.32085 | 0.61455 |
| GOBP_VASCULAR_ENDOTHELIAL_CELL_PROLIFERATION | ECM and metastasis | -0.87207 | 0.65026 | 0.83016 | 0.95163 | 0.55427 | 0.58197 | 0.62966 | 0.93044 | 0.87060 |
| GOBP_REGULATION_OF_LIPID_METABOLIC_PROCESS | Metabolism and energy | -1.11404 | 0.21043 | 0.56852 | -1.27239 | 0.00272 | 0.01257 | 0.88043 | 0.81190 | 0.86488 |
| GOBP_PHOSPHATIDYLINOSITOL_METABOLIC_PROCESS | Metabolism and energy | -0.95672 | 0.55890 | 0.79590 | 0.62109 | 0.99068 | 0.72077 | 0.50059 | 0.99989 | 0.87060 |
| GOBP_POSITIVE_REGULATION_OF_PROTEIN_POLYUBIQUITINATION | Immunity | 0.71975 | 0.82623 | 0.88692 | 0.91445 | 0.60317 | 0.60784 | 0.56373 | 0.96152 | 0.87060 |
| GOBP_RESPONSE_TO_MONOSACCHARIDE | Metabolism and energy | -0.98619 | 0.49735 | 0.77060 | 1.02023 | 0.45340 | 0.52165 | 0.97676 | 0.55335 | 0.76590 |
| GOBP_NEGATIVE_REGULATION_OF_LYMPHOCYTE_APOPTOTIC_PROCESS | Immunity | -1.68733 | 0.00524 | 0.06911 | -1.38106 | 0.05760 | 0.12703 | -1.13556 | 0.24131 | 0.53979 |
| GOBP_POSITIVE_REGULATION_OF_FATTY_ACID_BIOSYNTHETIC_PROCESS | Metabolism and energy | -1.17431 | 0.25877 | 0.61103 | 0.72458 | 0.84345 | 0.70759 | 0.73949 | 0.82522 | 0.86865 |
| GOBP_NEGATIVE_REGULATION_OF_TRANSFORMING_GROWTH_FACTOR_BETA_RECEPTOR_SIGNALING_PATHWAY | ECM and metastasis | 1.59507 | 0.00505 | 0.06836 | -1.25962 | 0.05702 | 0.12600 | 1.58117 | 0.00198 | 0.03757 |
| GOBP_PYRIDINE_NUCLEOTIDE_BIOSYNTHETIC_PROCESS | Genetic and epigenetic information | 1.08302 | 0.33859 | 0.67277 | -1.37660 | 0.07502 | 0.15325 | 1.04283 | 0.42212 | 0.69509 |
| GOBP_POSITIVE_REGULATION_OF_TYPE_I_INTERFERON_PRODUCTION | Immunity | 1.09265 | 0.29605 | 0.64096 | -1.58097 | 0.00552 | 0.02180 | -0.89898 | 0.66474 | 0.81342 |
| GOBP_CELL_CELL_ADHESION_MEDIATED_BY_CADHERIN | ECM and metastasis | -1.58221 | 0.01904 | 0.15887 | 1.19382 | 0.23051 | 0.33839 | 0.81571 | 0.74090 | 0.83944 |
| GOBP_CELL_CELL_JUNCTION_ASSEMBLY | ECM and metastasis | -1.40146 | 0.01982 | 0.16323 | 1.03934 | 0.41213 | 0.49306 | 1.28282 | 0.05592 | 0.26802 |
| GOBP_RESPONSE_TO_TUMOR_NECROSIS_FACTOR | Immunity | -1.05090 | 0.35323 | 0.68692 | -1.98905 | 0.00029 | 0.00231 | -1.22213 | 0.03519 | 0.20730 |
| GOBP_GRANULOCYTE_DIFFERENTIATION | Immunity | -0.95742 | 0.52992 | 0.78354 | -1.35488 | 0.06815 | 0.14366 | 1.14638 | 0.27768 | 0.57747 |
| GOBP_REGULATION_OF_AUTOPHAGY | Cell death | -0.87921 | 0.78959 | 0.87715 | -0.89199 | 0.92331 | 0.72077 | -1.32060 | 0.00141 | 0.02992 |
| GOBP_REGULATION_OF_TRANSLATIONAL_INITIATION | Metabolism and energy | 0.54007 | 0.99895 | 0.88692 | 0.46644 | 0.99932 | 0.72077 | -2.18536 | 0.00004 | 0.00345 |
| GOBP_PROTEIN_POLYUBIQUITINATION | Immunity | -0.60062 | 0.99987 | 0.88692 | 0.55140 | 0.99972 | 0.72077 | -1.15496 | 0.08019 | 0.32119 |
| GOBP_NUCLEOTIDE_TRANSPORT | Genetic and epigenetic information | -0.93816 | 0.56071 | 0.79653 | -0.67479 | 0.95945 | 0.72077 | -1.62300 | 0.01699 | 0.13769 |
| GOBP_POSITIVE_REGULATION_OF_PEPTIDASE_ACTIVITY | Metabolism and energy | 1.19579 | 0.09596 | 0.39410 | -2.04938 | 0.00012 | 0.00124 | -1.26231 | 0.03259 | 0.19757 |
| GOBP_POSITIVE_REGULATION_OF_LIPID_LOCALIZATION | Metabolism and energy | -0.95521 | 0.55213 | 0.79244 | -1.70812 | 0.00014 | 0.00142 | 1.15026 | 0.22372 | 0.52165 |
| GOBP_NEGATIVE_REGULATION_OF_INFLAMMATORY_RESPONSE_TO_ANTIGENIC_STIMULUS | Immunity | -0.50331 | 0.98951 | 0.88692 | 0.76568 | 0.80444 | 0.69460 | 0.80985 | 0.74738 | 0.84201 |
| GOBP_L_GLUTAMATE_TRANSMEMBRANE_TRANSPORT | Metabolism and energy | 0.83156 | 0.71209 | 0.85294 | 1.06906 | 0.38750 | 0.47370 | 1.06232 | 0.39435 | 0.67616 |
| GOBP_NEGATIVE_REGULATION_OF_OXIDATIVE_STRESS_INDUCED_CELL_DEATH | Cell death | -1.14124 | 0.26957 | 0.61914 | -0.89604 | 0.67012 | 0.64015 | -1.00695 | 0.42647 | 0.69760 |
| GOBP_SOMATIC_DIVERSIFICATION_OF_IMMUNOGLOBULINS | Immunity | -0.75154 | 0.87416 | 0.88692 | 0.47528 | 0.99851 | 0.72077 | -0.76861 | 0.90457 | 0.87060 |
| GOBP_AUTOPHAGOSOME_ORGANIZATION | Cell death | -0.79914 | 0.85128 | 0.88692 | -0.88270 | 0.80111 | 0.69341 | -1.04616 | 0.34078 | 0.63194 |
| GOBP_TIGHT_JUNCTION_ORGANIZATION | ECM and metastasis | -1.32581 | 0.07419 | 0.34842 | 0.98303 | 0.52570 | 0.56471 | 1.07703 | 0.35387 | 0.64115 |
| GOBP_POSITIVE_REGULATION_OF_EXTRINSIC_APOPTOTIC_SIGNALING_PATHWAY | Cell death | 0.90686 | 0.61705 | 0.81658 | -1.35106 | 0.05102 | 0.11603 | 0.84548 | 0.72594 | 0.83517 |
| GOBP_REGULATION_OF_MACROAUTOPHAGY | Cell death | -0.62241 | 0.99746 | 0.88692 | -0.73125 | 0.99422 | 0.72077 | -1.03798 | 0.34986 | 0.63933 |
| GOBP_REGULATION_OF_CELL_CELL_ADHESION_MEDIATED_BY_CADHERIN | ECM and metastasis | -1.63064 | 0.01399 | 0.13177 | 1.05577 | 0.40990 | 0.49131 | 0.73684 | 0.82670 | 0.86938 |
| GOBP_RESPONSE_TO_PURINE_CONTAINING_COMPOUND | Genetic and epigenetic information | 1.09891 | 0.24103 | 0.59635 | -1.25785 | 0.03454 | 0.08732 | -1.09782 | 0.22151 | 0.52058 |
| GOBP_REGULATION_OF_T_CELL_CHEMOTAXIS | Immunity | -1.00753 | 0.46354 | 0.75485 | 0.76303 | 0.79392 | 0.69048 | -1.18370 | 0.22921 | 0.52884 |
| GOBP_ACYLGLYCEROL_HOMEOSTASIS | Metabolism and energy | -1.05228 | 0.38995 | 0.71025 | 1.14395 | 0.27990 | 0.38473 | 1.05608 | 0.40010 | 0.68041 |
| GOBP_CHAPERONE_COFACTOR_DEPENDENT_PROTEIN_REFOLDING | Metabolism and energy | 0.81846 | 0.73833 | 0.86073 | 0.69742 | 0.88463 | 0.72044 | -1.28936 | 0.12144 | 0.39333 |
| GOBP_REGULATION_OF_DNA_BINDING_TRANSCRIPTION_FACTOR_ACTIVITY | Genetic and epigenetic information | -0.92905 | 0.67987 | 0.84067 | -1.16243 | 0.03086 | 0.08076 | 0.89291 | 0.81280 | 0.86488 |
| GOBP_POSITIVE_REGULATION_OF_LIPID_TRANSPORT | Metabolism and energy | -0.96686 | 0.52493 | 0.78007 | -1.42491 | 0.01146 | 0.03819 | 1.19977 | 0.17135 | 0.46252 |
| GOBP_CELL_JUNCTION_ASSEMBLY | ECM and metastasis | 1.35764 | 0.00215 | 0.03821 | 1.09909 | 0.18997 | 0.29578 | 1.40475 | 0.00024 | 0.00935 |
| GOBP_CELL_CELL_JUNCTION_ORGANIZATION | ECM and metastasis | -1.21899 | 0.10078 | 0.40326 | 0.85797 | 0.82114 | 0.70000 | 1.29733 | 0.03041 | 0.19147 |
| GOBP_REGULATION_OF_FATTY_ACID_TRANSPORT | Metabolism and energy | -0.78186 | 0.79124 | 0.87715 | -0.98953 | 0.47123 | 0.53117 | 1.36104 | 0.08293 | 0.32815 |
| GOBP_NEGATIVE_REGULATION_OF_TOLL_LIKE_RECEPTOR_SIGNALING_PATHWAY | Immunity | -1.01825 | 0.43838 | 0.74009 | -1.49983 | 0.02570 | 0.07048 | -0.96925 | 0.49443 | 0.73424 |
| GOBP_APICAL_JUNCTION_ASSEMBLY | ECM and metastasis | -1.47712 | 0.02097 | 0.17044 | 0.95672 | 0.57137 | 0.59128 | 1.03100 | 0.43365 | 0.70113 |
| GOBP_REGULATION_OF_VASCULAR_ENDOTHELIAL_GROWTH_FACTOR_RECEPTOR_SIGNALING_PATHWAY | ECM and metastasis | 1.35853 | 0.10717 | 0.41235 | 0.99024 | 0.50037 | 0.54923 | 1.09976 | 0.34539 | 0.63652 |
| GOBP_LIPID_HOMEOSTASIS | Metabolism and energy | -1.20175 | 0.13770 | 0.46306 | 0.71905 | 0.94865 | 0.72077 | 1.00985 | 0.47482 | 0.72645 |
| GOBP_NEGATIVE_REGULATION_OF_RESPONSE_TO_CYTOKINE_STIMULUS | Immunity | -1.00951 | 0.44716 | 0.74495 | -1.44615 | 0.01370 | 0.04377 | -1.08986 | 0.27314 | 0.57238 |
| GOBP_PHOSPHOLIPID_METABOLIC_PROCESS | Metabolism and energy | -1.06243 | 0.31140 | 0.65424 | -0.95861 | 0.66566 | 0.63902 | 0.70913 | 0.98893 | 0.87060 |
| GOBP_CELLULAR_MODIFIED_AMINO_ACID_CATABOLIC_PROCESS | Metabolism and energy | 1.34873 | 0.10827 | 0.41440 | -0.87476 | 0.68006 | 0.64364 | 0.87237 | 0.66661 | 0.81370 |
| GOBP_POSITIVE_REGULATION_OF_OXIDATIVE_STRESS_INDUCED_CELL_DEATH | Cell death | 1.30664 | 0.15487 | 0.48742 | -1.81001 | 0.00746 | 0.02766 | -1.21551 | 0.19465 | 0.48960 |
| GOBP_CELLULAR_RESPONSE_TO_CARBOHYDRATE_STIMULUS | Metabolism and energy | -1.02338 | 0.41816 | 0.72776 | 0.91816 | 0.67337 | 0.64131 | 0.98326 | 0.53005 | 0.75361 |
| GOBP_TYPE_I_INTERFERON_PRODUCTION | Immunity | 0.97593 | 0.50131 | 0.77188 | -1.66849 | 0.00023 | 0.00197 | -0.97857 | 0.50722 | 0.74122 |
| GOBP_GLUCOSE_IMPORT | Metabolism and energy | 1.15335 | 0.21059 | 0.56852 | 0.86001 | 0.72809 | 0.66447 | 1.03378 | 0.42948 | 0.69921 |
| GOBP_POSITIVE_REGULATION_OF_CELLULAR_CARBOHYDRATE_METABOLIC_PROCESS | Metabolism and energy | 1.12194 | 0.26300 | 0.61462 | -1.01558 | 0.42353 | 0.50205 | 0.96428 | 0.54246 | 0.76096 |
| GOBP_REGULATION_OF_MONOCYTE_DIFFERENTIATION | Immunity | -0.99693 | 0.47606 | 0.76212 | -1.78220 | 0.00938 | 0.03292 | 1.28808 | 0.15220 | 0.43795 |
| GOBP_DE_NOVO_PROTEIN_FOLDING | Metabolism and energy | 0.80065 | 0.78609 | 0.87612 | 0.71958 | 0.87324 | 0.71725 | -1.24297 | 0.14067 | 0.42233 |
| GOBP_REGULATION_OF_BICELLULAR_TIGHT_JUNCTION_ASSEMBLY | ECM and metastasis | -1.08584 | 0.36057 | 0.69224 | -1.13890 | 0.26250 | 0.36918 | 0.79797 | 0.75415 | 0.84431 |
| GOBP_UNSATURATED_FATTY_ACID_BIOSYNTHETIC_PROCESS | Metabolism and energy | -1.27015 | 0.13805 | 0.46306 | -1.41800 | 0.03362 | 0.08575 | 0.84451 | 0.72375 | 0.83517 |
| GOBP_REGULATION_OF_DNA_DAMAGE_RESPONSE_SIGNAL_TRANSDUCTION_BY_P53_CLASS_MEDIATOR | Genetic and epigenetic information | 1.00686 | 0.43512 | 0.73713 | -1.09250 | 0.29771 | 0.40159 | 0.60211 | 0.96148 | 0.87060 |
| GOBP_TRANSLATIONAL_INITIATION | Metabolism and energy | 0.80216 | 0.88866 | 0.88692 | 0.44384 | 0.99994 | 0.72077 | -2.13942 | 0.00002 | 0.00269 |
| GOBP_POSITIVE_REGULATION_OF_G1_S_TRANSITION_OF_MITOTIC_CELL_CYCLE | Cell cycle | -1.00180 | 0.46214 | 0.75453 | 0.80324 | 0.78025 | 0.68669 | 0.71090 | 0.88569 | 0.87060 |
| GOBP_POSITIVE_REGULATION_OF_CELL_JUNCTION_ASSEMBLY | ECM and metastasis | 1.09010 | 0.27137 | 0.61958 | 1.09924 | 0.30691 | 0.41006 | 1.69763 | 0.00016 | 0.00741 |
| GOBP_NEUTROPHIL_ACTIVATION_INVOLVED_IN_IMMUNE_RESPONSE | Immunity | -1.30241 | 0.14363 | 0.47352 | -2.34178 | 0.00005 | 0.00076 | -1.42455 | 0.07572 | 0.31233 |
| GOBP_TYROSINE_PHOSPHORYLATION_OF_STAT_PROTEIN | Immunity | -1.08629 | 0.32195 | 0.66175 | 0.79376 | 0.82958 | 0.70301 | 0.91278 | 0.64201 | 0.80463 |
| GOBP_B_CELL_APOPTOTIC_PROCESS | Immunity | -0.86098 | 0.66933 | 0.83639 | -1.16296 | 0.22508 | 0.33307 | -2.10433 | 0.00087 | 0.02206 |
| GOBP_ACTIVATION_OF_CYSTEINE_TYPE_ENDOPEPTIDASE_ACTIVITY | Cell death | 0.70000 | 0.85681 | 0.88692 | -1.43106 | 0.06909 | 0.14491 | -1.10512 | 0.30117 | 0.60023 |
| GOBP_REGULATION_OF_FATTY_ACID_OXIDATION | Metabolism and energy | -1.40493 | 0.06578 | 0.32549 | -0.95289 | 0.54215 | 0.57526 | 1.22430 | 0.18967 | 0.48536 |
| GOBP_RIBONUCLEOTIDE_CATABOLIC_PROCESS | Genetic and epigenetic information | -0.98311 | 0.49118 | 0.76857 | 0.64921 | 0.94244 | 0.72077 | 0.74211 | 0.85986 | 0.87060 |
| GOBP_NEGATIVE_REGULATION_OF_CHEMOKINE_PRODUCTION | Immunity | 0.99623 | 0.45196 | 0.74790 | -1.76001 | 0.00806 | 0.02940 | -0.99866 | 0.44095 | 0.70613 |
| GOBP_REGULATION_OF_PROTEIN_MATURATION | Metabolism and energy | 1.37034 | 0.05443 | 0.29362 | -1.56488 | 0.00446 | 0.01849 | 0.92808 | 0.61107 | 0.79249 |
| GOBP_EXTRINSIC_APOPTOTIC_SIGNALING_PATHWAY_VIA_DEATH_DOMAIN_RECEPTORS | Cell death | 1.32946 | 0.06515 | 0.32392 | -1.85201 | 0.00012 | 0.00130 | 0.87868 | 0.69966 | 0.82593 |
| GOBP_REGULATION_OF_INTEGRIN_ACTIVATION | ECM and metastasis | 0.95753 | 0.49979 | 0.77118 | -1.24093 | 0.17852 | 0.28395 | -1.14108 | 0.26834 | 0.56879 |
| GOBP_NEGATIVE_REGULATION_OF_GLUCOSE_TRANSMEMBRANE_TRANSPORT | Metabolism and energy | 1.07494 | 0.35841 | 0.69104 | 1.00324 | 0.48111 | 0.53758 | 0.94441 | 0.55758 | 0.76763 |
| GOBP_PHOSPHATIDYLGLYCEROL_METABOLIC_PROCESS | Metabolism and energy | -1.58998 | 0.01503 | 0.13723 | 0.64021 | 0.93510 | 0.72077 | 0.64831 | 0.93039 | 0.87060 |
| GOBP_GLYCEROLIPID_METABOLIC_PROCESS | Metabolism and energy | -1.06877 | 0.29640 | 0.64096 | -1.01542 | 0.42415 | 0.50255 | 0.72932 | 0.98313 | 0.87060 |
| GOBP_POSITIVE_REGULATION_OF_LIPID_CATABOLIC_PROCESS | Metabolism and energy | -1.00757 | 0.45727 | 0.75198 | 1.14811 | 0.28416 | 0.38872 | 1.14628 | 0.28674 | 0.58711 |
| GOBP_ESTABLISHMENT_OF_LYMPHOCYTE_POLARITY | Immunity | 0.72275 | 0.81897 | 0.88586 | -1.80955 | 0.00896 | 0.03193 | -0.91040 | 0.57002 | 0.77238 |
| GOBP_REGULATION_OF_TRANSCRIPTION_INVOLVED_IN_G1_S_TRANSITION_OF_MITOTIC_CELL_CYCLE | Cell cycle | -0.60633 | 0.94415 | 0.88692 | 1.17521 | 0.25796 | 0.36540 | -0.91673 | 0.57181 | 0.77324 |
| GOBP_CELL_CELL_ADHESION_VIA_PLASMA_MEMBRANE_ADHESION_MOLECULES | ECM and metastasis | 1.44208 | 0.00223 | 0.03932 | 0.97693 | 0.57225 | 0.59128 | 1.61207 | 0.00000 | 0.00074 |
| GOBP_GLYCEROPHOSPHOLIPID_METABOLIC_PROCESS | Metabolism and energy | -0.97793 | 0.52810 | 0.78307 | -0.87353 | 0.93761 | 0.72077 | 0.70856 | 0.98224 | 0.87060 |
| GOBP_POSITIVE_REGULATION_OF_LIPID_STORAGE | Metabolism and energy | -0.72529 | 0.84580 | 0.88692 | -1.89763 | 0.00253 | 0.01194 | 0.81490 | 0.73808 | 0.83838 |
| GOBP_NEGATIVE_REGULATION_OF_INTERLEUKIN_1_PRODUCTION | Immunity | -1.01705 | 0.43994 | 0.74070 | -1.28872 | 0.09987 | 0.18945 | 0.95218 | 0.55550 | 0.76637 |
| GOBP_POSITIVE_REGULATION_OF_FATTY_ACID_METABOLIC_PROCESS | Metabolism and energy | -1.01187 | 0.44757 | 0.74506 | -1.08280 | 0.31119 | 0.41301 | -0.86979 | 0.68658 | 0.82339 |
| GOBP_FATTY_ACID_METABOLIC_PROCESS | Metabolism and energy | -1.44768 | 0.00126 | 0.02603 | -0.98071 | 0.57669 | 0.59432 | 0.92063 | 0.73512 | 0.83705 |
| GOBP_NEGATIVE_REGULATION_OF_INNATE_IMMUNE_RESPONSE | Immunity | -0.88003 | 0.67785 | 0.84067 | -1.83746 | 0.00017 | 0.00157 | -1.01829 | 0.40818 | 0.68702 |
| GOBP_RESPONSE_TO_PLATELET_DERIVED_GROWTH_FACTOR | ECM and metastasis | 1.09046 | 0.33499 | 0.66950 | 0.75309 | 0.81356 | 0.69780 | 0.97785 | 0.51291 | 0.74380 |
| GOBP_REGULATION_OF_RESPONSE_TO_CYTOKINE_STIMULUS | Immunity | -1.05808 | 0.34859 | 0.68236 | -1.72483 | 0.00008 | 0.00097 | -1.30070 | 0.02519 | 0.17164 |
| GOBP_PHOSPHATIDYLCHOLINE_METABOLIC_PROCESS | Metabolism and energy | -1.10620 | 0.29613 | 0.64096 | -1.18823 | 0.12897 | 0.22697 | 0.96720 | 0.54502 | 0.76180 |
| GOBP_TRIGLYCERIDE_METABOLIC_PROCESS | Metabolism and energy | -1.36715 | 0.04665 | 0.27078 | -1.16986 | 0.12803 | 0.22624 | 0.62767 | 0.97915 | 0.87060 |
| GOBP_NUCLEOSIDE_PHOSPHATE_CATABOLIC_PROCESS | Genetic and epigenetic information | -0.94080 | 0.57327 | 0.79827 | 0.69319 | 0.93571 | 0.72077 | 0.76898 | 0.86091 | 0.87060 |
| GOBP_CELLULAR_CARBOHYDRATE_METABOLIC_PROCESS | Metabolism and energy | 1.24489 | 0.03392 | 0.22863 | -1.04410 | 0.29574 | 0.39990 | -0.75957 | 0.99666 | 0.87060 |
| GOBP_PYROPTOSIS | Cell death | -1.06804 | 0.37914 | 0.70211 | -1.95373 | 0.00162 | 0.00840 | -1.48446 | 0.05313 | 0.25953 |
| GOBP_ANTIGEN_PROCESSING_AND_PRESENTATION_OF_ENDOGENOUS_PEPTIDE_ANTIGEN | Immunity | -1.36945 | 0.10664 | 0.41227 | -2.25506 | 0.00026 | 0.00211 | -1.24737 | 0.19631 | 0.49058 |
| GOBP_SOMATIC_DIVERSIFICATION_OF_IMMUNE_RECEPTORS | Immunity | -0.68880 | 0.94938 | 0.88692 | 0.63433 | 0.96758 | 0.72077 | -0.63688 | 0.99281 | 0.87060 |
| GOBP_SNRNA_TRANSCRIPTION | Genetic and epigenetic information | -0.80111 | 0.74128 | 0.86073 | 0.80403 | 0.74788 | 0.67301 | -0.79605 | 0.75490 | 0.84452 |
| GOBP_LIPID_CATABOLIC_PROCESS | Metabolism and energy | -1.27412 | 0.03590 | 0.23598 | -0.92329 | 0.81351 | 0.69780 | 0.97483 | 0.57746 | 0.77629 |
| GOBP_FATTY_ACID_TRANSPORT | Metabolism and energy | -1.13630 | 0.24070 | 0.59629 | 0.84300 | 0.77951 | 0.68663 | 1.34801 | 0.04257 | 0.22933 |
| GOBP_ANTIGEN_PROCESSING_AND_PRESENTATION_OF_PEPTIDE_ANTIGEN_VIA_MHC_CLASS_I | Immunity | -1.23723 | 0.19582 | 0.55089 | -2.53867 | 0.00001 | 0.00035 | -1.36138 | 0.09878 | 0.35399 |
| GOBP_NEGATIVE_REGULATION_OF_RECEPTOR_SIGNALING_PATHWAY_VIA_STAT | Immunity | -0.94776 | 0.54264 | 0.78945 | 1.01119 | 0.47001 | 0.53062 | 1.11247 | 0.33054 | 0.62319 |
| GOBP_NEGATIVE_REGULATION_OF_INTERLEUKIN_1_BETA_PRODUCTION | Immunity | -1.18280 | 0.23610 | 0.59150 | -1.28289 | 0.11371 | 0.20778 | 0.69482 | 0.88686 | 0.87060 |
| GOBP_POSITIVE_REGULATION_OF_LIPID_METABOLIC_PROCESS | Metabolism and energy | -1.42539 | 0.01548 | 0.13975 | -1.20225 | 0.06126 | 0.13282 | 0.81234 | 0.84617 | 0.87060 |
| GOBP_REGULATION_OF_GLUTAMATE_SECRETION | Metabolism and energy | -1.28871 | 0.15504 | 0.48759 | 1.51771 | 0.02698 | 0.07300 | -1.35156 | 0.10790 | 0.36990 |
| GOBP_NUCLEOTIDE_SUGAR_METABOLIC_PROCESS | Genetic and epigenetic information | 1.49658 | 0.04322 | 0.25921 | -1.48243 | 0.03109 | 0.08100 | 0.69621 | 0.88966 | 0.87060 |
| GOBP_PURINE_NUCLEOTIDE_CATABOLIC_PROCESS | Genetic and epigenetic information | -1.07740 | 0.34934 | 0.68251 | 0.85601 | 0.71378 | 0.66033 | 0.74379 | 0.85697 | 0.87060 |
| GOBP_RESPONSE_TO_INTERLEUKIN_4 | Immunity | -0.92119 | 0.58549 | 0.80293 | -1.17531 | 0.19785 | 0.30393 | 0.69790 | 0.88810 | 0.87060 |
| GOBP_REGULATION_OF_PRODUCTION_OF_SMALL_RNA_INVOLVED_IN_GENE_SILENCING_BY_RNA | Genetic and epigenetic information | 0.85764 | 0.66351 | 0.83352 | -1.36550 | 0.07985 | 0.16058 | 0.49102 | 0.99087 | 0.87060 |
| GOBP_REGULATION_OF_LIPOPROTEIN_LIPASE_ACTIVITY | Metabolism and energy | -1.32787 | 0.12918 | 0.44916 | 0.81783 | 0.73047 | 0.66571 | -1.03131 | 0.39394 | 0.67564 |
| GOBP_NEGATIVE_REGULATION_OF_ANOIKIS | Cell death | 1.34768 | 0.13323 | 0.45625 | -1.66470 | 0.01944 | 0.05730 | 0.85537 | 0.67823 | 0.82041 |
| GOBP_POSITIVE_REGULATION_OF_CYSTEINE_TYPE_ENDOPEPTIDASE_ACTIVITY | Cell death | -0.80932 | 0.84807 | 0.88692 | -1.65439 | 0.00016 | 0.00151 | -1.55894 | 0.00190 | 0.03676 |
| GOBP_STEROID_METABOLIC_PROCESS | Metabolism and energy | -1.36085 | 0.01069 | 0.11150 | 0.55916 | 0.99987 | 0.72077 | 0.74612 | 0.96666 | 0.87060 |
| GOBP_REGULATION_OF_STEROID_METABOLIC_PROCESS | Metabolism and energy | -1.34972 | 0.05335 | 0.29151 | -0.95025 | 0.60497 | 0.60898 | -1.09516 | 0.24962 | 0.54724 |
| GOBP_MODIFIED_AMINO_ACID_TRANSPORT | Metabolism and energy | 0.88556 | 0.64531 | 0.82896 | 0.78184 | 0.80522 | 0.69490 | 1.38529 | 0.06203 | 0.28178 |
| GOBP_POSITIVE_REGULATION_OF_GLUCOSE_METABOLIC_PROCESS | Metabolism and energy | 1.13691 | 0.26127 | 0.61293 | 0.79295 | 0.78818 | 0.68850 | 0.92690 | 0.59399 | 0.78511 |
| GOBP_RESPONSE_TO_CHOLESTEROL | Metabolism and energy | -1.08683 | 0.35372 | 0.68737 | 0.42985 | 0.99747 | 0.72077 | 0.69860 | 0.87398 | 0.87060 |
| GOBP_AMINO_ACID_TRANSMEMBRANE_TRANSPORT | Metabolism and energy | 0.89548 | 0.69734 | 0.84709 | 1.18874 | 0.16909 | 0.27408 | 1.13097 | 0.25727 | 0.55753 |
| GOBP_TRANSLESION_SYNTHESIS | Genetic and epigenetic information | -0.46610 | 0.99478 | 0.88692 | 1.19531 | 0.23094 | 0.33873 | 0.53892 | 0.97861 | 0.87060 |
| GOBP_OLIGOSACCHARIDE_METABOLIC_PROCESS | Metabolism and energy | 0.73816 | 0.91122 | 0.88692 | 0.90000 | 0.65733 | 0.63529 | 0.88749 | 0.67056 | 0.81579 |
| GOBP_NEGATIVE_REGULATION_OF_LIPID_TRANSPORT | Metabolism and energy | -0.91149 | 0.59629 | 0.80705 | -0.84165 | 0.73438 | 0.66762 | 0.81942 | 0.73741 | 0.83777 |
| GOBP_POSITIVE_REGULATION_OF_SPROUTING_ANGIOGENESIS | ECM and metastasis | 0.90683 | 0.57783 | 0.79962 | 1.41290 | 0.06364 | 0.13687 | 0.97440 | 0.51732 | 0.74603 |
| GOBP_NEGATIVE_REGULATION_OF_RECEPTOR_SIGNALING_PATHWAY_VIA_JAK_STAT | Immunity | -0.83461 | 0.70158 | 0.84942 | 0.91759 | 0.60085 | 0.60625 | 1.01824 | 0.45708 | 0.71693 |
| GOBP_REGULATION_OF_LIPID_CATABOLIC_PROCESS | Metabolism and energy | -1.08748 | 0.32753 | 0.66505 | 0.72123 | 0.89560 | 0.72077 | 1.05912 | 0.38849 | 0.67146 |
| GOBP_L_ALPHA_AMINO_ACID_TRANSMEMBRANE_TRANSPORT | Metabolism and energy | 0.83614 | 0.79093 | 0.87715 | 1.13080 | 0.27042 | 0.37672 | 1.20663 | 0.17212 | 0.46339 |
| GOBP_NEGATIVE_REGULATION_OF_LIPID_METABOLIC_PROCESS | Metabolism and energy | -1.10766 | 0.28124 | 0.62789 | 0.57401 | 0.99278 | 0.72077 | 1.07383 | 0.35353 | 0.64072 |
| GOBP_POSITIVE_REGULATION_OF_INTERFERON_BETA_PRODUCTION | Immunity | 1.21014 | 0.18919 | 0.54215 | -1.96919 | 0.00042 | 0.00306 | 0.55036 | 0.98326 | 0.87060 |
| GOBP_REGULATION_OF_CYSTEINE_TYPE_ENDOPEPTIDASE_ACTIVITY | Cell death | 1.14420 | 0.13501 | 0.45889 | -1.80406 | 0.00022 | 0.00187 | -1.49680 | 0.00063 | 0.01732 |
| GOBP_NEGATIVE_REGULATION_OF_LIPID_BIOSYNTHETIC_PROCESS | Metabolism and energy | -0.92845 | 0.58373 | 0.80271 | 0.59327 | 0.97614 | 0.72077 | -1.10677 | 0.26169 | 0.56281 |
| GOBP_REGULATION_OF_RECEPTOR_SIGNALING_PATHWAY_VIA_STAT | Immunity | -1.17710 | 0.18732 | 0.54008 | 1.37139 | 0.02691 | 0.07294 | 1.31284 | 0.05970 | 0.27766 |
| GOBP_LIPID_MODIFICATION | Metabolism and energy | -1.06454 | 0.32724 | 0.66505 | -0.74808 | 0.99763 | 0.72077 | 0.55619 | 0.99963 | 0.87060 |
| GOBP_FATTY_ACID_BIOSYNTHETIC_PROCESS | Metabolism and energy | -1.43094 | 0.01420 | 0.13231 | 0.63505 | 0.98664 | 0.72077 | 0.95003 | 0.60351 | 0.78834 |
| GOBP_TRIGLYCERIDE_CATABOLIC_PROCESS | Metabolism and energy | -1.32161 | 0.11802 | 0.42993 | 0.56309 | 0.97293 | 0.72077 | 1.07812 | 0.37194 | 0.65782 |
| GOBP_REGULATION_OF_FATTY_ACID_METABOLIC_PROCESS | Metabolism and energy | -1.25473 | 0.11934 | 0.43183 | 0.80673 | 0.82055 | 0.69996 | 0.95060 | 0.57983 | 0.77722 |
| GOBP_REGULATION_OF_PEPTIDASE_ACTIVITY | Metabolism and energy | 1.42304 | 0.00065 | 0.01538 | -1.82915 | 0.00562 | 0.02211 | 1.01031 | 0.47076 | 0.72365 |
| GOBP_CELLULAR_MODIFIED_AMINO_ACID_METABOLIC_PROCESS | Metabolism and energy | 1.24775 | 0.06277 | 0.31859 | -1.30260 | 0.01379 | 0.04396 | 1.10802 | 0.26832 | 0.56879 |
| GOBP_ACTIVATION_OF_INNATE_IMMUNE_RESPONSE | Immunity | -1.08125 | 0.33953 | 0.67326 | -1.00100 | 0.45578 | 0.52229 | 0.83541 | 0.74704 | 0.84201 |
| GOBP_REGULATION_OF_STEROID_BIOSYNTHETIC_PROCESS | Metabolism and energy | -1.36751 | 0.05793 | 0.30449 | -0.99253 | 0.47892 | 0.53629 | -1.21170 | 0.12993 | 0.40652 |
| GOBP_AMINO_ACID_TRANSPORT | Metabolism and energy | 0.85872 | 0.82514 | 0.88692 | 1.28441 | 0.04944 | 0.11369 | 0.95454 | 0.59268 | 0.78511 |
| GOBP_PHOSPHOLIPID_TRANSPORT | Metabolism and energy | -1.02167 | 0.42347 | 0.73096 | 0.65602 | 0.96618 | 0.72077 | -1.12153 | 0.20602 | 0.50222 |
| GOBP_NEGATIVE_REGULATION_OF_EXTRINSIC_APOPTOTIC_SIGNALING_PATHWAY | Cell death | 1.60760 | 0.00423 | 0.05998 | -1.47908 | 0.00499 | 0.02020 | 1.19359 | 0.17384 | 0.46456 |
| GOBP_ONE_CARBON_COMPOUND_TRANSPORT | Genetic and epigenetic information | -1.91793 | 0.00040 | 0.01052 | -0.98316 | 0.47265 | 0.53186 | -2.34566 | 0.00009 | 0.00552 |
| GOBP_CYTOPLASMIC_TRANSLATION | Metabolism and energy | 0.62470 | 0.98965 | 0.88692 | 0.48186 | 0.99861 | 0.72077 | -1.56500 | 0.00972 | 0.10060 |
| GOBP_ACIDIC_AMINO_ACID_TRANSPORT | Metabolism and energy | 0.79060 | 0.85314 | 0.88692 | 1.23160 | 0.14780 | 0.24948 | 0.84802 | 0.73678 | 0.83751 |
| GOBP_CELLULAR_AMINO_ACID_CATABOLIC_PROCESS | Metabolism and energy | -1.34705 | 0.05083 | 0.28412 | 0.80605 | 0.83674 | 0.70537 | -0.84792 | 0.84178 | 0.87060 |
| GOBP_POSITIVE_REGULATION_OF_MIRNA_TRANSCRIPTION | Genetic and epigenetic information | -1.64416 | 0.00665 | 0.08302 | -1.53287 | 0.01403 | 0.04446 | -1.61757 | 0.01418 | 0.12522 |
| GOBP_GLYCEROPHOSPHOLIPID_CATABOLIC_PROCESS | Metabolism and energy | -1.66506 | 0.00731 | 0.08813 | -0.90489 | 0.63205 | 0.62157 | 0.74231 | 0.84030 | 0.87060 |
| GOBP_INTERLEUKIN_1_MEDIATED_SIGNALING_PATHWAY | Immunity | -1.08627 | 0.35443 | 0.68772 | -1.23530 | 0.16041 | 0.26487 | -2.18130 | 0.00042 | 0.01372 |
| GOBP_PURINE_NUCLEOTIDE_TRANSPORT | Genetic and epigenetic information | -0.83982 | 0.70419 | 0.85060 | -1.38625 | 0.06553 | 0.13996 | -1.68923 | 0.01578 | 0.13222 |
| GOBP_GLUTAMINE_FAMILY_AMINO_ACID_CATABOLIC_PROCESS | Metabolism and energy | -1.03933 | 0.41622 | 0.72624 | 1.56639 | 0.01502 | 0.04695 | 0.57034 | 0.96553 | 0.87060 |
| GOBP_NEGATIVE_REGULATION_OF_PROTEIN_MATURATION | Metabolism and energy | 1.32833 | 0.12387 | 0.44046 | -1.47113 | 0.04235 | 0.10155 | 0.62151 | 0.94050 | 0.87060 |
| GOBP_REGULATION_OF_PLASMA_LIPOPROTEIN_PARTICLE_LEVELS | Metabolism and energy | -0.91300 | 0.61967 | 0.81805 | -1.39528 | 0.02198 | 0.06274 | 0.79239 | 0.82129 | 0.86721 |
| GOBP_LONG_CHAIN_FATTY_ACYL_COA_BIOSYNTHETIC_PROCESS | Metabolism and energy | -0.61537 | 0.93512 | 0.88692 | 1.07598 | 0.38316 | 0.47085 | 0.63362 | 0.92156 | 0.87060 |
| GOBP_DNA_DOUBLE_STRAND_BREAK_PROCESSING | Genetic and epigenetic information | -0.64775 | 0.91318 | 0.88692 | 1.40233 | 0.06991 | 0.14593 | 0.73471 | 0.82909 | 0.86979 |
| GOBP_GLUTAMATE_SECRETION | Metabolism and energy | -0.78723 | 0.77314 | 0.87083 | 1.24757 | 0.17567 | 0.28120 | -1.20495 | 0.18913 | 0.48488 |
| GOBP_POSITIVE_REGULATION_OF_TYROSINE_PHOSPHORYLATION_OF_STAT_PROTEIN | Immunity | -0.99732 | 0.46819 | 0.75874 | 0.93853 | 0.59682 | 0.60477 | 0.95725 | 0.55827 | 0.76776 |
| GOBP_POSITIVE_REGULATION_OF_INSULIN_SECRETION_INVOLVED_IN_CELLULAR_RESPONSE_TO_GLUCOSE_STIMULUS | Metabolism and energy | 0.81975 | 0.72255 | 0.85522 | -0.76939 | 0.83690 | 0.70540 | 1.24153 | 0.18369 | 0.47862 |
| GOBP_CYTOPLASMIC_TRANSLATIONAL_INITIATION | Metabolism and energy | -0.62611 | 0.94944 | 0.88692 | -0.83617 | 0.75427 | 0.67626 | -1.73489 | 0.01045 | 0.10388 |
| GOBP_REGULATION_OF_FATTY_ACID_BIOSYNTHETIC_PROCESS | Metabolism and energy | -1.40137 | 0.06154 | 0.31361 | 0.88607 | 0.66508 | 0.63899 | 0.72401 | 0.87245 | 0.87060 |
| GOBP_POSITIVE_REGULATION_OF_NUCLEOTIDE_BIOSYNTHETIC_PROCESS | Genetic and epigenetic information | -0.95727 | 0.52848 | 0.78334 | -0.93456 | 0.55319 | 0.58127 | -1.09797 | 0.30769 | 0.60635 |
| GOBP_NADP_METABOLIC_PROCESS | Metabolism and energy | 0.83817 | 0.73434 | 0.86010 | -1.46385 | 0.02646 | 0.07204 | 0.51731 | 0.99220 | 0.87060 |
| GOBP_NEGATIVE_REGULATION_OF_CYSTEINE_TYPE_ENDOPEPTIDASE_ACTIVITY | Cell death | 1.25423 | 0.10689 | 0.41235 | -1.68893 | 0.00067 | 0.00432 | -1.17482 | 0.15513 | 0.44270 |
| GOBP_POSITIVE_REGULATION_OF_TRANSCRIPTION_BY_RNA_POLYMERASE_III | Genetic and epigenetic information | 0.69502 | 0.85419 | 0.88692 | 0.71910 | 0.84242 | 0.70738 | 0.74819 | 0.80907 | 0.86391 |
| GOBP_REGULATION_OF_MIRNA_TRANSCRIPTION | Genetic and epigenetic information | -1.59029 | 0.00852 | 0.09720 | -1.70040 | 0.00164 | 0.00845 | -1.34308 | 0.06145 | 0.28027 |
| GOBP_REGULATION_OF_PHOSPHOLIPID_METABOLIC_PROCESS | Metabolism and energy | -1.20403 | 0.21073 | 0.56864 | -1.04203 | 0.37867 | 0.46734 | -0.75149 | 0.86894 | 0.87060 |
| GOBP_POSTREPLICATION_REPAIR | Genetic and epigenetic information | -0.53872 | 0.98763 | 0.88692 | 1.22843 | 0.18295 | 0.28837 | 0.30382 | 1.00000 | 0.87060 |
| GOBP_NEGATIVE_REGULATION_OF_PROTEOLYSIS | Metabolism and energy | 1.36280 | 0.00505 | 0.06836 | -1.53558 | 0.00116 | 0.00657 | 1.09740 | 0.23941 | 0.53849 |
| GOBP_RESPONSE_TO_INTERFERON_BETA | Immunity | 1.45260 | 0.06556 | 0.32519 | -2.07714 | 0.00048 | 0.00333 | 1.08922 | 0.35924 | 0.64636 |
| GOBP_CELLULAR_RESPONSE_TO_INTERFERON_BETA | Immunity | -0.80771 | 0.73588 | 0.86010 | -1.36233 | 0.09502 | 0.18314 | 1.10128 | 0.34829 | 0.63853 |
| GOBP_ASPARTATE_FAMILY_AMINO_ACID_BIOSYNTHETIC_PROCESS | Metabolism and energy | -0.67140 | 0.89311 | 0.88692 | -0.64556 | 0.94252 | 0.72077 | 0.68388 | 0.88093 | 0.87060 |
| GOBP_POSITIVE_REGULATION_OF_PROTEIN_MATURATION | Metabolism and energy | 1.00049 | 0.44580 | 0.74489 | -1.44887 | 0.04692 | 0.10943 | 1.23146 | 0.19121 | 0.48707 |
| GOBP_AUTOPHAGY_OF_NUCLEUS | Cell death | -0.71686 | 0.83862 | 0.88692 | -1.37036 | 0.10095 | 0.19088 | -1.17004 | 0.24124 | 0.53979 |
| GOBP_NEUTRAL_LIPID_METABOLIC_PROCESS | Metabolism and energy | -1.30389 | 0.06630 | 0.32589 | -0.99750 | 0.47626 | 0.53424 | -0.90585 | 0.72598 | 0.83517 |
| GOBP_NEGATIVE_REGULATION_OF_PEPTIDASE_ACTIVITY | Metabolism and energy | 1.47655 | 0.00207 | 0.03710 | -1.66990 | 0.00032 | 0.00243 | 1.35114 | 0.00947 | 0.09887 |
| GOBP_REGULATION_OF_NUCLEOTIDE_BIOSYNTHETIC_PROCESS | Genetic and epigenetic information | -0.91497 | 0.59556 | 0.80693 | 0.67088 | 0.91315 | 0.72077 | -1.25014 | 0.14164 | 0.42442 |
| GOBP_FATTY_ACID_DERIVATIVE_BIOSYNTHETIC_PROCESS | Metabolism and energy | -1.14857 | 0.26004 | 0.61221 | 0.88559 | 0.66760 | 0.63987 | 0.76273 | 0.83088 | 0.87006 |
| GOBP_INTERFERON_BETA_PRODUCTION | Immunity | 1.04766 | 0.36471 | 0.69542 | -2.01350 | 0.00008 | 0.00096 | -0.80756 | 0.83693 | 0.87060 |
| GOBP_POSITIVE_REGULATION_OF_TRANSCRIPTION_REGULATORY_REGION_DNA_BINDING | Genetic and epigenetic information | -0.82944 | 0.70864 | 0.85153 | 1.12946 | 0.31198 | 0.41393 | 1.10479 | 0.34251 | 0.63383 |
| GOBP_HIGH_DENSITY_LIPOPROTEIN_PARTICLE_REMODELING | Metabolism and energy | -0.87966 | 0.63423 | 0.82623 | 0.93320 | 0.57728 | 0.59461 | 0.73588 | 0.82164 | 0.86721 |
| GOBP_BRANCHED_CHAIN_AMINO_ACID_METABOLIC_PROCESS | Metabolism and energy | -1.17997 | 0.24430 | 0.59886 | 0.56486 | 0.96971 | 0.72077 | -1.12213 | 0.26899 | 0.56879 |
| GOBP_MITOTIC_SISTER_CHROMATID_COHESION | Cell cycle | 0.43584 | 0.99791 | 0.88692 | 1.45783 | 0.04104 | 0.09890 | -0.80098 | 0.77324 | 0.85267 |
| GOBP_NEUTRAL_LIPID_BIOSYNTHETIC_PROCESS | Metabolism and energy | -1.07033 | 0.36094 | 0.69224 | -1.63243 | 0.00630 | 0.02421 | -1.12694 | 0.24442 | 0.54193 |
| GOBP_REGULATION_OF_CYTOPLASMIC_TRANSLATION | Metabolism and energy | 1.13223 | 0.28259 | 0.63003 | 0.84591 | 0.70489 | 0.65642 | -1.11763 | 0.27451 | 0.57338 |
| GOBP_GLYCEROLIPID_CATABOLIC_PROCESS | Metabolism and energy | -1.75974 | 0.00084 | 0.01911 | -0.73788 | 0.95451 | 0.72077 | 0.79838 | 0.80884 | 0.86391 |
| GOBP_NEGATIVE_REGULATION_OF_LIPID_CATABOLIC_PROCESS | Metabolism and energy | -1.34775 | 0.10725 | 0.41235 | 1.27500 | 0.15181 | 0.25432 | 1.21155 | 0.21511 | 0.51428 |
| GOBP_CYTOPLASMIC_PATTERN_RECOGNITION_RECEPTOR_SIGNALING_PATHWAY | Immunity | -0.72737 | 0.89948 | 0.88692 | -1.66758 | 0.00240 | 0.01142 | 0.55917 | 0.98923 | 0.87060 |
| GOBP_NUCLEOBASE_METABOLIC_PROCESS | Genetic and epigenetic information | 0.90355 | 0.61112 | 0.81519 | 1.19567 | 0.21421 | 0.32176 | -1.05036 | 0.35344 | 0.64072 |
| GOBP_AROMATIC_AMINO_ACID_FAMILY_METABOLIC_PROCESS | Metabolism and energy | -0.89508 | 0.62070 | 0.81851 | 0.75832 | 0.81581 | 0.69797 | -0.86564 | 0.67490 | 0.81885 |
| GOBP_GLUTAMATE_METABOLIC_PROCESS | Metabolism and energy | -1.16071 | 0.25866 | 0.61103 | 1.13068 | 0.30162 | 0.40509 | 0.60720 | 0.95490 | 0.87060 |
| GOBP_POSITIVE_REGULATION_OF_LEUKOCYTE_DEGRANULATION | Immunity | -1.00074 | 0.46831 | 0.75875 | -1.56372 | 0.02766 | 0.07420 | 0.63179 | 0.92886 | 0.87060 |
| GOBP_OLIGOSACCHARIDE_BIOSYNTHETIC_PROCESS | Metabolism and energy | -0.85789 | 0.67534 | 0.83911 | 0.72575 | 0.85052 | 0.71001 | 0.94358 | 0.56285 | 0.76847 |
| GOBP_POSITIVE_REGULATION_OF_LIPID_BIOSYNTHETIC_PROCESS | Metabolism and energy | -1.61660 | 0.00364 | 0.05362 | -0.89164 | 0.74749 | 0.67301 | -0.90528 | 0.68293 | 0.82131 |
| GOBP_GLUTAMINE_FAMILY_AMINO_ACID_METABOLIC_PROCESS | Metabolism and energy | -0.91229 | 0.62300 | 0.81990 | 0.96130 | 0.56395 | 0.58765 | -0.73205 | 0.95620 | 0.87060 |
| GOBP_TRICARBOXYLIC_ACID_CYCLE | Metabolism and energy | -1.06270 | 0.38290 | 0.70547 | 0.70677 | 0.86959 | 0.71613 | -1.09918 | 0.29683 | 0.59561 |
| GOBP_BRANCHED_CHAIN_AMINO_ACID_CATABOLIC_PROCESS | Metabolism and energy | -1.45305 | 0.05822 | 0.30500 | -0.42747 | 0.99962 | 0.72077 | -0.96456 | 0.49282 | 0.73342 |
| GOBP_TRANSLATIONAL_TERMINATION | Metabolism and energy | 1.20782 | 0.24105 | 0.59635 | 0.59964 | 0.93770 | 0.72077 | 0.52381 | 0.97539 | 0.87060 |
| GOBP_INTERFERON_GAMMA_MEDIATED_SIGNALING_PATHWAY | Immunity | -1.33021 | 0.11731 | 0.42908 | -1.56287 | 0.02486 | 0.06857 | -1.10951 | 0.28594 | 0.58638 |
| GOBP_SULFUR_AMINO_ACID_METABOLIC_PROCESS | Metabolism and energy | -0.81491 | 0.74168 | 0.86075 | -0.61643 | 0.97637 | 0.72077 | -0.94733 | 0.53107 | 0.75434 |
| GOBP_REGULATION_OF_INTRINSIC_APOPTOTIC_SIGNALING_PATHWAY_BY_P53_CLASS_MEDIATOR | Cell death | -1.13267 | 0.29780 | 0.64182 | 0.58953 | 0.95724 | 0.72077 | 0.66727 | 0.90455 | 0.87060 |
| GOBP_ACETYL_COA_BIOSYNTHETIC_PROCESS | Metabolism and energy | -1.23163 | 0.20671 | 0.56745 | 0.63983 | 0.91528 | 0.72077 | 0.70982 | 0.85221 | 0.87060 |
| GOBP_REGULATION_OF_PHOSPHOLIPID_BIOSYNTHETIC_PROCESS | Metabolism and energy | -0.48757 | 0.98776 | 0.88692 | -1.35174 | 0.10750 | 0.20004 | 1.17684 | 0.26217 | 0.56339 |
| GOBP_INTERLEUKIN_13_PRODUCTION | Immunity | -1.31191 | 0.13755 | 0.46306 | -1.31946 | 0.11691 | 0.21215 | 1.09142 | 0.36078 | 0.64801 |
| GOBP_NEUTRAL_LIPID_CATABOLIC_PROCESS | Metabolism and energy | -1.41894 | 0.05679 | 0.30074 | 0.79125 | 0.79185 | 0.68984 | 1.01968 | 0.45310 | 0.71313 |
| GOBP_ASPARTATE_FAMILY_AMINO_ACID_METABOLIC_PROCESS | Metabolism and energy | -1.04327 | 0.39921 | 0.71513 | 0.88484 | 0.66944 | 0.64015 | 0.66119 | 0.93274 | 0.87060 |
| GOBP_METHIONINE_METABOLIC_PROCESS | Metabolism and energy | -1.24615 | 0.19634 | 0.55137 | 0.76437 | 0.79241 | 0.69003 | -0.93201 | 0.53682 | 0.75679 |
| GOBP_REGULATION_OF_MACROPHAGE_DIFFERENTIATION | Immunity | 1.42153 | 0.08676 | 0.37550 | -2.00061 | 0.00123 | 0.00682 | 0.81494 | 0.73497 | 0.83705 |
| GOBP_NATURAL_KILLER_CELL_ACTIVATION_INVOLVED_IN_IMMUNE_RESPONSE | Immunity | -0.99079 | 0.48046 | 0.76283 | 1.68018 | 0.00314 | 0.01416 | 1.54102 | 0.01855 | 0.14265 |

Table S6.

TTT Score and TTT for each samples in LUAD-BT

| **id** | **TTT (months)** | **status** | **IL11RA** | **WIF1** | **PLAU** | **C19orf40** | **DAXX** | **JUN** | **WNT11** | **CACNA2D3** | **TTT score** | **Binary of TTT score (>-0.855 was high, <=-0.855 was low)** |
| --- | --- | --- | --- | --- | --- | --- | --- | --- | --- | --- | --- | --- |
| LUAD-BT18 | 67.25 | transformed | 1 | 1 | 1 | 0 | 0 | 1 | 1 | 1 | -2.91588 | low |
| LUAD-BT6 | 54.72 | transformed | 1 | 1 | 1 | 0 | 0 | 1 | 1 | 1 | -2.91588 | low |
| LUAD-BT9 | 63.90 | transformed | 1 | 1 | 1 | 0 | 0 | 1 | 1 | 1 | -2.91588 | low |
| LUAD-BT7 | 57.93 | transformed | 1 | 0 | 1 | 0 | 0 | 1 | 1 | 1 | -2.54881 | low |
| LUAD-BT10 | 46.07 | transformed | 0 | 1 | 1 | 0 | 1 | 1 | 0 | 1 | -2.20593 | low |
| LUAD-BT16 | 38.23 | transformed | 1 | 1 | 0 | 0 | 0 | 0 | 0 | 1 | -1.95730 | low |
| LUAD-BT2 | 36.00 | transformed | 0 | 1 | 0 | 0 | 0 | 1 | 1 | 1 | -1.64713 | low |
| LUAD-BT17 | 37.11 | transformed | 0 | 1 | 1 | 1 | 0 | 1 | 1 | 1 | -1.21095 | low |
| LUAD-BT1 | 28.98 | transformed | 1 | 1 | 0 | 1 | 0 | 0 | 1 | 1 | -0.93385 | low |
| LUAD-BT3 | 31.57 | transformed | 1 | 1 | 0 | 1 | 1 | 0 | 0 | 1 | -0.77520 | high |
| LUAD-BT15 | 32.79 | transformed | 1 | 1 | 1 | 1 | 1 | 1 | 0 | 0 | -0.56470 | high |
| LUAD-BT11 | 27.54 | transformed | 0 | 0 | 0 | 0 | 1 | 0 | 1 | 0 | -0.10172 | high |
| LUAD-BT14 | 21.61 | transformed | 1 | 0 | 1 | 1 | 1 | 0 | 0 | 0 | -0.08666 | high |
| LUAD-BT8 | 13.90 | transformed | 0 | 0 | 1 | 1 | 1 | 1 | 0 | 0 | 0.35369 | high |
| LUAD-BT4 | 11.05 | transformed | 0 | 0 | 1 | 1 | 1 | 0 | 0 | 0 | 0.46465 | high |
| LUAD-BT13 | 16.03 | transformed | 1 | 0 | 0 | 1 | 0 | 0 | 0 | 0 | 0.60232 | high |
| LUAD-BT12 | 5.57 | transformed | 0 | 0 | 0 | 1 | 1 | 1 | 0 | 0 | 1.07113 | high |
| LUAD-BT5 | 5.05 | transformed | 0 | 0 | 0 | 1 | 1 | 0 | 0 | 0 | 1.18209 | high |

Table S7.

OStrans Score and survival data for SCLC samples (SCLC-AT and SCLC-P).

| **patient id** | **Type** | **OStrans/OS (months)** | **status** | **BAIAP3** | **DDIT4** | **CDKN2A** | **FGF2** | **GADD45B** | **SGK2** | **TNFRSF10B** | **SHC4** | **FGF3** | **OStrans score** | **Binary of OStrans score (>-0.062 was high, <=-0.062 was low)** |
| --- | --- | --- | --- | --- | --- | --- | --- | --- | --- | --- | --- | --- | --- | --- |
| SCLC-AT13 | SCLC-AT | 28.89 | Censored | 0 | 1 | 1 | 1 | 0 | 0 | 0 | 0 | 0 | -0.77984 | low |
| SCLC-AT4 | SCLC-AT | 26.79 | Dead | 0 | 1 | 1 | 1 | 0 | 0 | 0 | 0 | 0 | -0.77984 | low |
| SCLC-AT14 | SCLC-AT | 65.38 | Dead | 0 | 1 | 1 | 1 | 0 | 0 | 0 | 0 | 0 | -0.77984 | low |
| SCLC-P20 | SCLC-P | 18.79 | Censored | 0 | 0 | 1 | 1 | 1 | 0 | 0 | 0 | 0 | -0.50581 | low |
| SCLC-AT10 | SCLC-AT | 11.25 | Censored | 0 | 1 | 1 | 0 | 1 | 0 | 0 | 0 | 0 | -0.50475 | low |
| SCLC-P16 | SCLC-P | 4.26 | Dead | 0 | 1 | 1 | 0 | 1 | 1 | 0 | 0 | 0 | -0.48261 | low |
| SCLC-AT6 | SCLC-AT | 19.84 | Dead | 0 | 1 | 0 | 1 | 0 | 0 | 0 | 0 | 0 | -0.38182 | low |
| SCLC-AT12 | SCLC-AT | 1.41 | Censored | 0 | 0 | 1 | 0 | 0 | 1 | 0 | 0 | 0 | -0.37588 | low |
| SCLC-AT9 | SCLC-AT | 18.92 | Dead | 0 | 0 | 1 | 1 | 1 | 0 | 1 | 0 | 0 | -0.31605 | low |
| SCLC-P5 | SCLC-P | 51.54 | Censored | 0 | 0 | 1 | 0 | 1 | 0 | 0 | 0 | 0 | -0.31437 | low |
| SCLC-P17 | SCLC-P | 38.33 | Censored | 0 | 0 | 1 | 0 | 1 | 0 | 0 | 0 | 0 | -0.31437 | low |
| SCLC-P8 | SCLC-P | 29.74 | Dead | 0 | 0 | 1 | 1 | 1 | 1 | 1 | 0 | 0 | -0.29391 | low |
| SCLC-AT3 | SCLC-AT | 14.16 | Censored | 0 | 0 | 1 | 0 | 0 | 1 | 0 | 1 | 0 | -0.27593 | low |
| SCLC-P4 | SCLC-P | 14.46 | Censored | 1 | 1 | 1 | 1 | 1 | 1 | 0 | 0 | 0 | -0.26885 | low |
| SCLC-AT8 | SCLC-AT | 11.70 | Censored | 0 | 1 | 0 | 1 | 0 | 0 | 0 | 0 | 1 | -0.24586 | low |
| SCLC-P13 | SCLC-P | 45.77 | Censored | 1 | 0 | 1 | 1 | 0 | 0 | 0 | 0 | 0 | -0.18426 | low |
| SCLC-AT21 | SCLC-AT | 15.90 | Dead | 0 | 0 | 1 | 1 | 0 | 1 | 1 | 1 | 1 | -0.14165 | low |
| SCLC-P1 | SCLC-P | 17.18 | Censored | 1 | 1 | 1 | 1 | 1 | 1 | 0 | 0 | 1 | -0.13290 | low |
| SCLC-P18 | SCLC-P | 11.21 | Dead | 0 | 0 | 1 | 0 | 1 | 0 | 1 | 0 | 0 | -0.12462 | low |
| SCLC-P15 | SCLC-P | 19.28 | Censored | 1 | 0 | 1 | 1 | 1 | 0 | 0 | 0 | 0 | -0.10061 | low |
| SCLC-P9 | SCLC-P | 46.33 | Censored | 1 | 0 | 1 | 1 | 1 | 1 | 0 | 0 | 0 | -0.07847 | low |
| SCLC-AT15 | SCLC-AT | 1.97 | Censored | 1 | 1 | 1 | 1 | 0 | 1 | 1 | 1 | 0 | -0.06279 | low |
| SCLC-AT20 | SCLC-AT | 13.08 | Censored | 1 | 0 | 1 | 1 | 1 | 0 | 0 | 0 | 1 | 0.03534 | high |
| SCLC-P3 | SCLC-P | 3.31 | Censored | 1 | 0 | 1 | 0 | 1 | 0 | 0 | 0 | 0 | 0.09082 | high |
| SCLC-AT7 | SCLC-AT | 15.21 | Dead | 1 | 0 | 1 | 1 | 1 | 1 | 1 | 0 | 0 | 0.11129 | high |
| SCLC-AT1 | SCLC-AT | 16.26 | Dead | 0 | 1 | 0 | 0 | 1 | 0 | 0 | 1 | 1 | 0.12917 | high |
| SCLC-P7 | SCLC-P | 15.80 | Dead | 1 | 0 | 1 | 1 | 1 | 0 | 0 | 1 | 1 | 0.13529 | high |
| SCLC-P14 | SCLC-P | 18.03 | Censored | 1 | 0 | 1 | 1 | 1 | 0 | 1 | 1 | 0 | 0.18910 | high |
| SCLC-AT18 | SCLC-AT | 13.02 | Dead | 0 | 1 | 0 | 0 | 1 | 0 | 1 | 0 | 1 | 0.21898 | high |
| SCLC-P10 | SCLC-P | 0.95 | Censored | 1 | 1 | 0 | 1 | 1 | 1 | 1 | 0 | 0 | 0.31892 | high |
| SCLC-AT19 | SCLC-AT | 14.26 | Dead | 1 | 0 | 0 | 1 | 0 | 1 | 0 | 1 | 0 | 0.33585 | high |
| SCLC-P6 | SCLC-P | 32.95 | Dead | 1 | 1 | 1 | 0 | 1 | 1 | 1 | 1 | 1 | 0.34825 | high |
| SCLC-P12 | SCLC-P | 12.52 | Dead | 1 | 0 | 0 | 0 | 1 | 0 | 0 | 0 | 0 | 0.48884 | high |
| SCLC-P2 | SCLC-P | 47.74 | Censored | 1 | 0 | 1 | 0 | 1 | 1 | 1 | 1 | 1 | 0.53863 | high |
| SCLC-P19 | SCLC-P | 33.93 | Dead | 1 | 0 | 1 | 0 | 1 | 1 | 1 | 1 | 1 | 0.53863 | high |
| SCLC-AT16 | SCLC-AT | 11.21 | Censored | 1 | 0 | 0 | 1 | 1 | 1 | 1 | 0 | 1 | 0.64526 | high |
| SCLC-AT11 | SCLC-AT | 4.59 | Dead | 1 | 0 | 0 | 0 | 1 | 1 | 0 | 1 | 1 | 0.74689 | high |
| SCLC-P11 | SCLC-P | 45.64 | Censored | 1 | 0 | 0 | 0 | 1 | 1 | 1 | 1 | 0 | 0.80069 | high |
| SCLC-AT5 | SCLC-AT | 13.34 | Dead | 1 | 0 | 0 | 0 | 1 | 0 | 1 | 1 | 1 | 0.91451 | high |
| SCLC-AT2 | SCLC-AT | 9.57 | Dead | 1 | 0 | 0 | 0 | 1 | 1 | 1 | 1 | 1 | 0.93665 | high |
| SCLC-AT17 | SCLC-AT | 1.15 | Dead | 1 | 0 | 0 | 0 | 1 | 1 | 1 | 1 | 1 | 0.93665 | high |
